# Supplementary material for: Herpes ICP8 protein stimulates homologous recombination in human cells
Source: PLoS One. 2018 Aug 15;13(8):e0200955. doi: 10.1371/journal.pone.0200955 (PMC6093641; doi:10.1371/journal.pone.0200955)
Supplement: S1 Fig — Orthologous protein sequences were detected in sequenced genomes via reiterative use of PSIBLAST against the non-redundant protein sequence (nr) database with a cut off of 0.005 until convergence was reached. Query sequences: NP_040616.1 (Lambda exo); NP_059594.1 (phage P22 Abc2); NP_415865.1 (Rac prophage RecE); NP_040617.1 (lambda Beta); NP_415865.1 (Rac prophage RecT); YP_009137104.1 (Human alphaherpesvirus 1 ICP8); NP_613196.1 (Mamestra configurata nucleopolyhedrovirus A LEF-3); NP_059596.1 (phage P22 ERF); CAA86623.1 (Saccharomyces cerevisiae Rad52). Numbers reflect the number of unique species in which viral SynExo subunit genes were found in integrated viruses in their genomes or in sequenced viruses specific for these organisms. The numbers are color-coded to show the distribution of different SynExo protein families. Examples of some SynExo complexes are listed in S1 Table. (PDF) [file pone.0200955.s001.pdf]

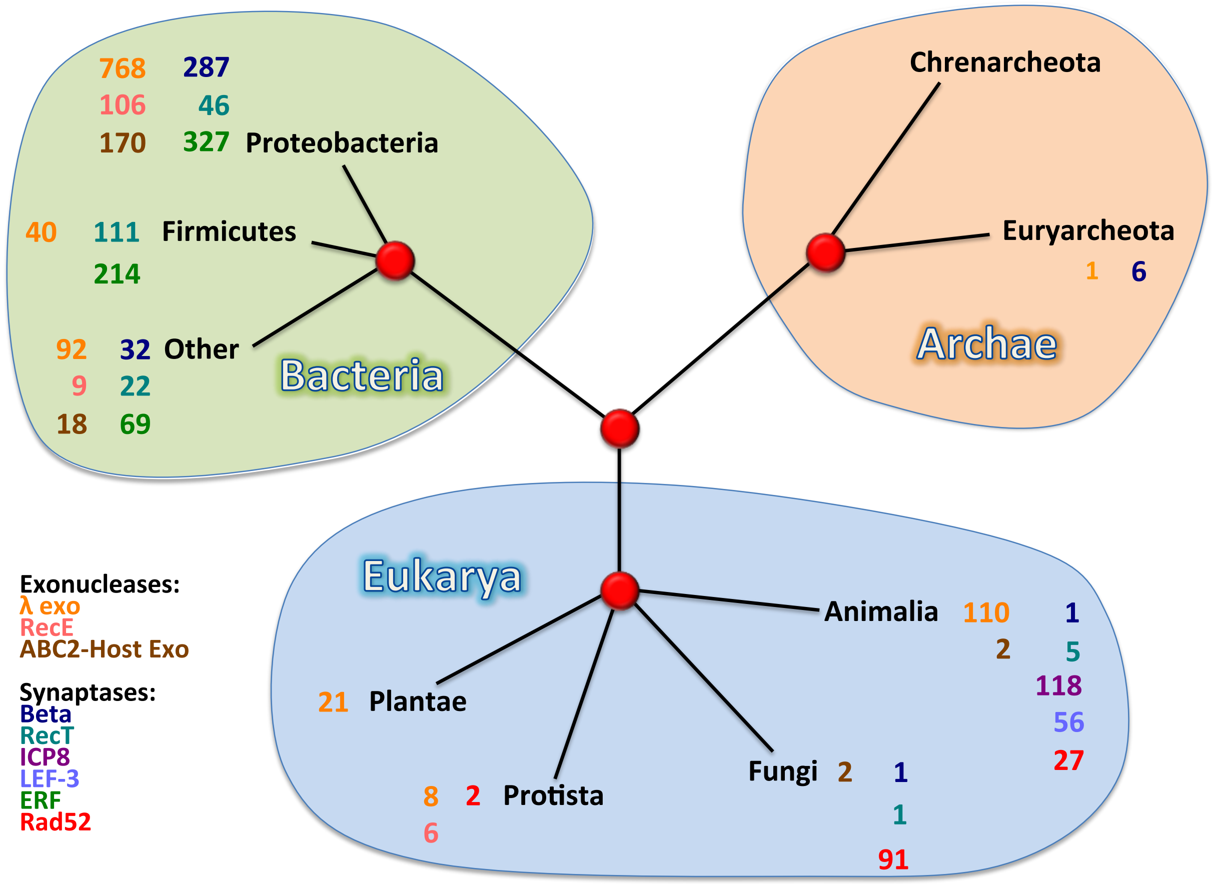

S1 Fig. SynExo genes are found in dsDNA viruses that infect Bacteria, Archae and Eukarya.

Orthologous protein sequences were detected in sequenced genomes via reiterative use of PSIBLAST against the non-redundant protein sequence (nr) database with a cut off of 0.005 until convergence was reached. Query sequences: NP_040616.1 (Lambda exo); NP_059594.1 (phage P22 Abc2); NP_415865.1 (Rac prophage RecE); NP_040617.1 (lambda Beta); NP_415865.1 (Rac prophage RecT); YP_009137104.1 (Human alphaherpesvirus 1 ICP8); NP_613196.1 (*Mamestra configurata* nucleopolyhedrovirus A LEF-3); NP_059596.1 (phage P22 ERF); CAA86623.1 (*Saccharomyces cerevisiae* Rad52). Numbers reflect the number of unique species in which viral SynExo subunit genes were found in integrated viruses in their genomes or in sequenced viruses specific for these organisms. The numbers are color-coded to show the distribution of different SynExo protein families. Examples of some SynExo complexes are listed in S8 Table.
