# Supplementary material for: Herpes ICP8 protein stimulates homologous recombination in human cells
Source: PLoS One. 2018 Aug 15;13(8):e0200955. doi: 10.1371/journal.pone.0200955 (PMC6093641; doi:10.1371/journal.pone.0200955)
Supplement: S2 Fig — A) Oligos for fluorescent protein engineering in human cells. Oligos that introduce a change in the target gene were used to evaluate recombineering and oligos that retained the target sequence were used as a “selfing” control. The labels next to the oligos refer to oligo numbers in S2 Table, the amino acid encoded at position 203 in the oligo, the strand identity of the oligo sequence with respect to the direction of transcription across the target gene, and the oligo length in nucleotides. The strand specificity of the oligo is notated as sense “s” or antisense “as” relative to the eGFPY203 coding sequence. B) Mismatches produced in recombination intermediates during annealing of oligo 85 (top) and oligo 84 (bottom) to the complementary strand of the Yellow gene target sequence. The oligos introduce the sequence for threonine at position 203, which changes the fluorescence spectral properties from Yellow to Green. There is a four nucleotide mismatch when targeting the Yellow gene with these oligos. (PDF) [file pone.0200955.s002.pdf]

A

```
                      Sense yellow target and sense oligos

    P  V  L  L  P  D  N  H  Y  L  S  Y  Q  S  A  L  S  K  D  P  N
5′ CCCGTGCTGCTGCCCGACAACCACTACCTGAGCTACCAGTCCGCCCTGAGCAAAGACCCCAACGA 3′  Yellow target, s
5′ CCCGTGCTGCTGCCCGACAACCACTACCTGTCCACCCAGTCCGCCCTGAGCAAAGACCCCAACGA 3′  84. T203 4s/65
5′ CCCGTGCTGCTGCCCGACAACCACTACCTGAGCTACCAGTCCGCCCTGAGCAAAGACCCCAACGA 3′ 130. Y203  s/65
        5′ GCTGCTGCCCGACAACCACTACCTGTCCACCCAGTCCGCCCTGAGCAAAGACCCC 3′       82. T203 4s/55
             5′ TGCCCGACAACCACTACCTGTCCACCCAGTCCGCCCTGAGCAAAG 3′            80. T203 4s/45
                  5′ GACAACCACTACCTGTCCACCCAGTCCGCCCTGAG 3′                 78. T203 4s/35
                    5′ CAACCACTACCTGAGCTACCAGTCCGCCCTGAGC 3′                70. Y203  s/34

                   Antisense yellow target and antisense oligos

3′ GGGCACGACGACGGGCTGTTGGTGATGGACTCGATGGTCAGGCGGGACTCGTTTCTGGGGTTGCT 5′ Yellow target, as
3′ GGGCACGACGACGGGCTGTTGGTGATGGACAGGTGGGTCAGGCGGGACTCGTTTCTGGGGTTGCT 5′  85. T203 4as/65
3′ GGGCACGACGACGGGCTGTTGGTGATGGACTCGATGGTCAGGCGGGACTCGTTTCTGGGGTTGCT 5′ 131. Y203  as/65
        3′ CGACGACGGGCTGTTGGTGATGGACAGGTGGGTCAGGCGGGACTCGTTTCTGGGG 5′       83. T203 4as/55
             3′ ACGGGCTGTTGGTGATGGACAGGTGGGTCAGGCGGGACTCGTTTC 5′            81. T203 4as/45
                  3′ CTGTTGGTGATGGACAGGTGGGTCAGGCGGGACTC 5′                 79. T203 4as/35
                    3′ GTTGGTGATGGACTCGATGGTCAGGCGGGACTCG 5′                71. Y203  as/34
```

B

```
             Mismatch in oligo and Yellow chromosomal target hybridization

5′ CCCGTGCTGCTGCCCGACAACCACTACCTGAGCTACCAGTCCGCCCTGAGCAAAGACCCCAACGA 3′Yellow target  s
   ||||||||||||||||||||||||||||||  |  ||||||||||||||||||||||||||||||
3′ GGGCACGACGACGGGCTGTTGGTGATGGACAGGTGGGTCAGGCGGGACTCGTTTCTGGGGTTGCT 5′Oligo 85 T203 as

3′ GGGCACGACGACGGGCTGTTGGTGATGGACTCGATGGTCAGGCGGGACTCGTTTCTGGGGTTGCT 5′Yellow target as
   ||||||||||||||||||||||||||||||  |  ||||||||||||||||||||||||||||||
5′ CCCGTGCTGCTGCCCGACAACCACTACCTGTCCACCCAGTCCGCCCTGAGCAAAGACCCCAACGA 3′Oligo 84 T203  s
```

S2 Fig. Recombineering substrates and targets.

A) Oligos for fluorescent protein engineering in human cells. Oligos that introduce a change in the target gene were used to evaluate recombineering and oligos that retained the target sequence were used as a “selfing” control. The labels next to the oligos refer to oligo numbers in S9 Table, the amino acid encoded at position 203 in the oligo, the strand identity of the oligo sequence with respect to the direction of transcription across the target gene, and the oligo length in nucleotides. The strand specificity of the oligo is notated as sense “s” or antisense “as” relative to the *eGFP*^{Y203} coding sequence. B) Mismatches produced in recombination intermediates during annealing of oligo 85 (top) and oligo 84 (bottom) to the complementary strand of the Yellow gene target sequence. The oligos introduce the sequence for threonine at position 203, which changes the fluorescence spectral properties from Yellow to Green. There is a four nucleotide mismatch when targeting the Yellow gene with these oligos.
