# Supplementary material for: Herpes ICP8 protein stimulates homologous recombination in human cells
Source: PLoS One. 2018 Aug 15;13(8):e0200955. doi: 10.1371/journal.pone.0200955 (PMC6093641; doi:10.1371/journal.pone.0200955)
Supplement: S3 Fig — Synaptase genes were fused to a red fluorescent gene, E2-Crimson (Strack et al. 2009) through a P2A linker (Szymczak-Workman et al. 2012) in a single open reading frame. The P2A linker causes ribosome skipping to produce equimolar amounts of the upstream and downstream protein products. The P2A peptide leaves a proline residue at the N-terminal end (Nt) of the C-terminal (Ct) protein and an 18 amino acid peptide at the Ct of the Nt protein. Previous reports have shown that these synaptases are moderately defective when fused to reporter genes (Taylor et al. 2003; Poteete 2011). Since we didn’t know if any of these additions might affect the recombination activity of the proteins, E2-Crimson was cloned either upstream or downstream of the synaptases in separate lentiviral constructs. (PDF) [file pone.0200955.s003.pdf]

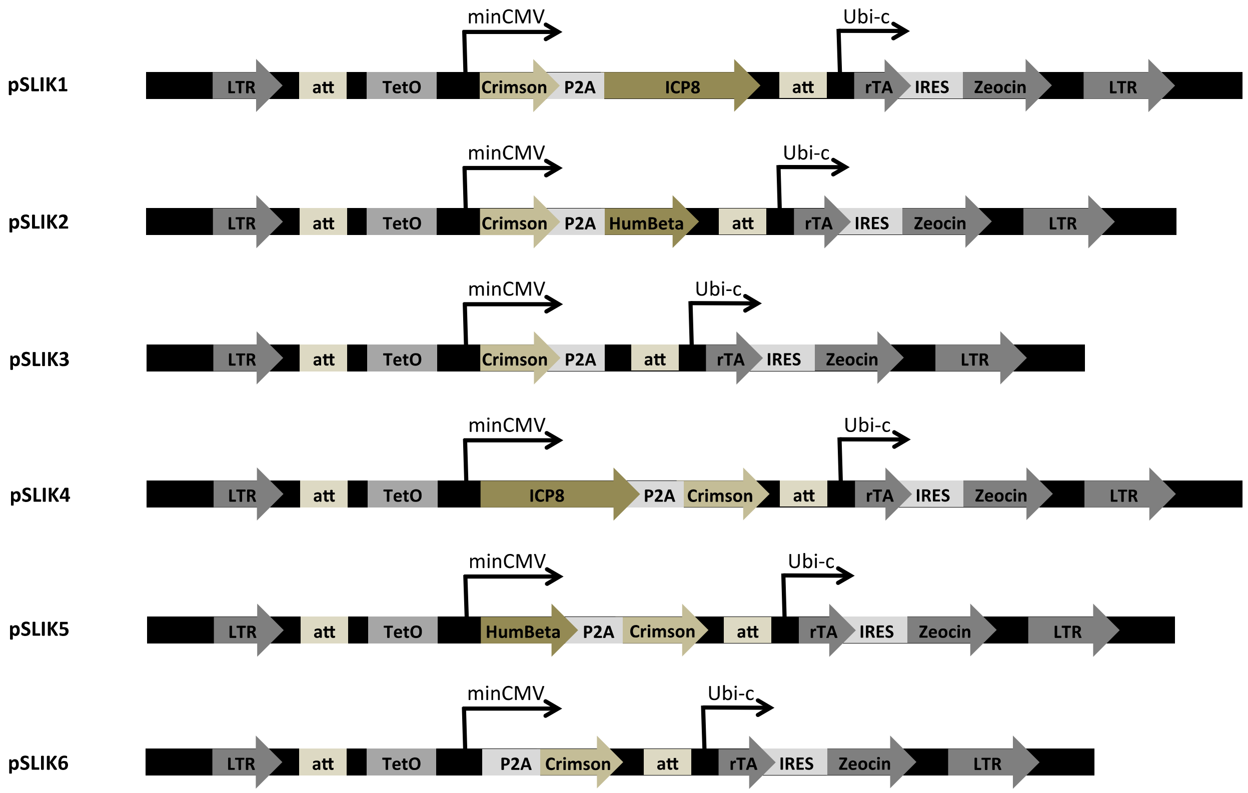

S3 Fig. Scheme for lentiviral plasmids encoding doxycycline-inducible synaptases.

Synaptase genes were fused to a red fluorescent gene, E2-Crimson (Strack et al. 2009) through a P2A linker (Szymczak-Workman et al. 2012) in a single open reading frame. The P2A linker causes ribosome skipping to produce equimolar amounts of the upstream and downstream protein products. The P2A peptide leaves a proline residue at the N-terminal end (Nt) of the C-terminal (Ct) protein and an 18 amino acid peptide at the Ct of the Nt protein. Previous reports have shown that these synaptases are moderately defective when fused to reporter genes (Taylor et al. 2003; Poteete 2011). Since we didn't know if any of these additions might affect the recombination activity of the proteins, E2-Crimson was cloned either upstream or downstream of the synaptases in separate lentiviral constructs. Maps and DNA sequences for plasmids constructed in this study are provided in S13 DNA Sequences.
