# Supplementary material for: Herpes ICP8 protein stimulates homologous recombination in human cells
Source: PLoS One. 2018 Aug 15;13(8):e0200955. doi: 10.1371/journal.pone.0200955 (PMC6093641; doi:10.1371/journal.pone.0200955)
Supplement: S4 Fig — Expression of viral synaptases and the Crimson reporter from pSLIK plasmids was validated in 293T cells. 293T cells were transiently transfected with each pSLIK plasmid and synaptase expression was induced with 1 μg/ml doxycycline in the media for 48 hours. ICP8 and HumBeta were detected by immunocytochemistry using anti-ICP8 (Abcam, ab20193) and anti-HA antibodies (Abcam, ab9110), respectively. Briefly, 293T cells were seeded onto poly-L-Lysine (Sigma) coated coverslips in 6 well plates in media. When cells were ready for imaging, cells adhering to coverslips were washed 3 times with PBS and then fixed in 4% paraformaldehyde in PBS pH 7.4 for 15 min at room temperature. Cells were washed 3 times with PBS and permeabilized with 0.25% Triton X-100 for 10 min. Cells were washed again and blocked with 1% BSA, 0.3 M glycine in PBST for 30 min. Cells were incubated with the primary antibody in 1% BSA in PBST in a humidified chamber overnight at 4°C. Cells were washed 3 times with PBS and incubated with the secondary antibody (which were labeled by Alexa Fluor) in 1% BSA for 1 hour at room temperature in the dark. Cells were washed and incubated with 0.5 μg/ml DAPI for 10 min. Cells were washed, mounted with Prolong antifade or Vectashield (Vector Laboratories). Cells were viewed with a Nikon Diaphot equipped with a Retiga 1300 camera. A Nikon 20X objective was used. Images were collected and analysed using IP-Lab software package. ICP8 and HumBeta are coloured green, E2-Crimson is coloured Red and DAPI is coloured blue. (PDF) [file pone.0200955.s004.pdf]

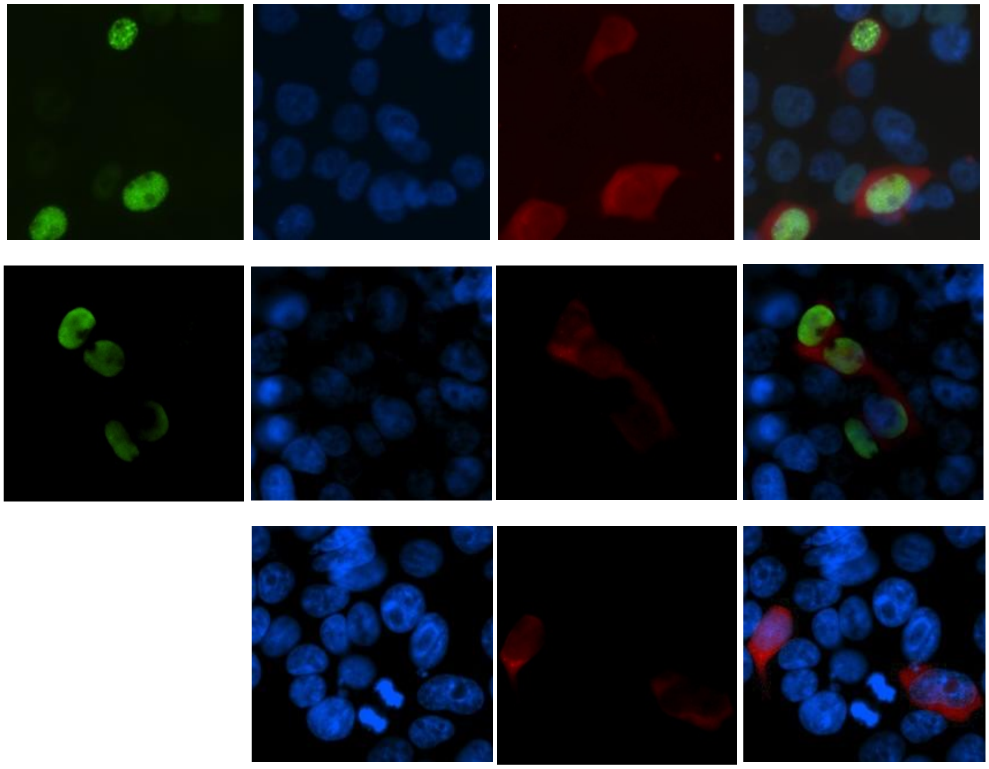

ICP8-P2A/Crimson

pSLIK1

HumBeta-P2A/Crimson

pSLIK2

Crimson-P2A

pSLIK6

**S4 Fig. ICP8 and HumBeta synaptases localize to the nucleus.**

Expression of viral synaptases and the Crimson reporter from pSLIK plasmids was validated in 293T cells. 293T cells were transiently transfected with each pSLIK plasmid and synaptase expression was induced with 1 µg/ml doxycycline in the media for 48 hours. ICP8 and HumBeta were detected by immunocytochemistry using anti-ICP8 (Abcam, ab20193) and anti-HA antibodies (Abcam, ab9110), respectively. Briefly, 293T cells were seeded onto poly-L-Lysine (Sigma) coated coverslips in 6 well plates in media. When cells were ready for imaging, cells adhering to coverslips were washed 3 times with PBS and then fixed in 4 % paraformaldehyde in PBS pH 7.4 for 15 min at room temperature. Cells were washed 3 times with PBS and permeabilized with 0.25 % Triton X-100 for 10 min. Cells were washed again and blocked with 1 % BSA, 0.3 M glycine in PBST for 30 min. Cells were incubated with the primary antibody in 1 % BSA in PBST in a humidified chamber overnight at 4 °C. Cells were washed 3 times with PBS and incubated with the secondary antibody (which were labeled by Alexa Fluor) in 1 % BSA for 1 hour at room temperature in the dark. Cells were washed and incubated with 0.5 µg/ml DAPI for 10 min. Cells were washed, mounted with Prolong antifade or Vectashield (Vector Laboratories). Cells were viewed with a Nikon Diaphot equipped with a Retiga 1300 camera. A Nikon 20X objective was used. Images were collected and analysed using IP-Lab software package. ICP8 and HumBeta are coloured green, E2-Crimson is coloured Red and DAPI is coloured blue.
