# Supplementary material for: Herpes ICP8 protein stimulates homologous recombination in human cells
Source: PLoS One. 2018 Aug 15;13(8):e0200955. doi: 10.1371/journal.pone.0200955 (PMC6093641; doi:10.1371/journal.pone.0200955)
Supplement: S5 Fig — 293T cells transfected with lentiviral vectors in the presence and absence of doxycycline (1 μg/ml) were collected 24 hours after transfection and analysed by Western blot as described by Abcam. ICP8 was detected with primary Herpes Virus I ICP8 Major DNA Binding Protein antibody (Abcam, ab20193) mouse monoclonal IGg1 and goat anti-mouse IGg1 secondary HRP labelled antibody [sc-2064] (Santa Cruz). HumBeta was detected with primary rabbit polyclonal anti-HA antibody (Abcam, ab9110) and goat anti-rabbit IgG secondary HRP labelled antibody [sc-2064] (Santa Cruz). Membranes were washed three times in TBST and incubated with the ECL Plus Western Blotting Detection System (from GE Healthcare RPN2132) for 5 min or until the bands glowed visibly. Films were exposed to membranes for varying amounts of time and then developed. Films were scanned and analysed using Photoshop or Image J software. A) ICP8 expression from pSLIK1 and pSLIK4 co-migrates with ICP8 expressed from the pCMV-ICP8 control. B) HumBeta expression from pSLIK2 and pSLIK5. The table indicates the masses of predicted protein products and observed outcomes in these Western Blots. All fusion proteins demonstrated >99% ribosome skipping efficiency, separating reporter fluorescent proteins from synaptases (trace amounts of fusion proteins were only evident when films were greatly overexposed, data not shown). (PDF) [file pone.0200955.s005.pdf]

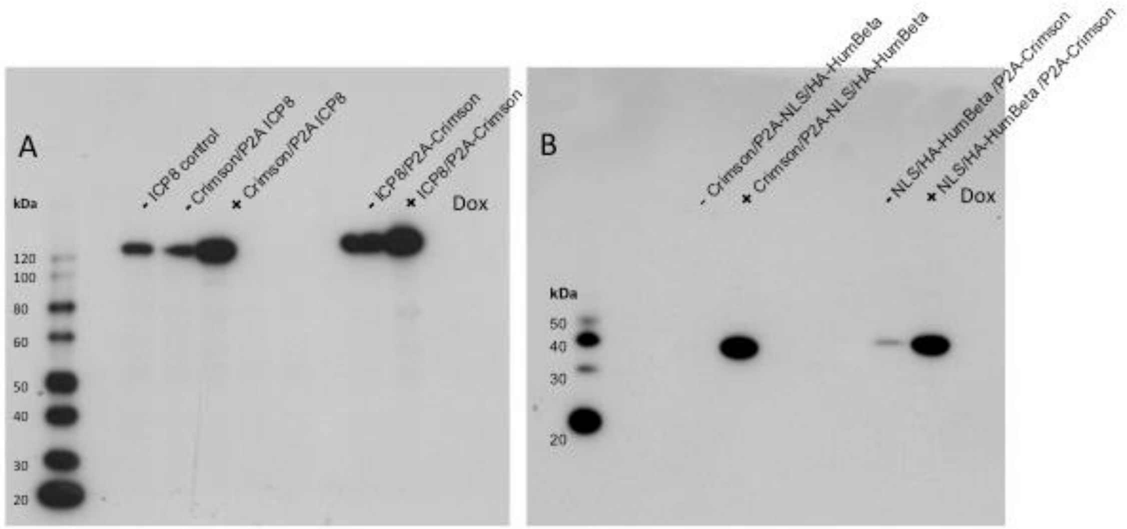

Gene	Possible proteins	Molecular Weight	Observed?
Crimson/P2A-ICP8 (pSLIK1)	Crimson-P2A-ICP8 (fusion protein)	157 kDa	No
	PH-ICP8	136 kDa	Yes
ICP8/P2A-Crimson (pSLIK4)	ICP8-P2A-Crimson (fusion protein)	156 kDa	No
	ICP8-P2A	137 kDa	Yes
Crimson/P2A-NLS/HA-HumBeta (pSLIK2)	Crimson/P2A-NLS/HA-HumBeta (fusion protein)	65 kDa	No
	PH-NLS/HA-HumBeta	36 kDa	Yes
NLS/HA-HumBeta/P2A-Crimson (pSLIK5)	NLS/HA-HumBeta/P2A-Crimson (fusion protein)	65 kDa	No
	NLS/HA-HumBeta/P2A	38 kDa	Yes

S5 Fig. ICP8 and HumBeta expression from pSLIK plasmids in transiently transfected 293T cells.

293T cells transfected with lentiviral vectors in the presence and absence of doxycycline (1 µg/ml) were collected 24 hours after transfection and analysed by Western blot as described by Abcam. ICP8 was detected with primary Herpes Virus I ICP8 Major DNA Binding Protein antibody (Abcam, ab20193) mouse monoclonal IGg_1 and goat anti-mouse IGg_1 secondary HRP labelled antibody [sc-2064] (Santa Cruz). HumBeta was detected with primary rabbit polyclonal anti-HA antibody (Abcam, ab9110) and goat anti-rabbit IgG secondary HRP labelled antibody [sc-2064] (Santa Cruz). Membranes were washed three times in TBST and incubated with the ECL Plus Western Blotting Detection System (from GE Healthcare RPN2132) for 5 min or until the bands glowed visibly. Films were exposed to membranes for varying amounts of time and then developed. Films were scanned and analysed using Photoshop or Image J software. A) ICP8 expression from pSLIK1 and pSLIK4 co-migrates with ICP8 expressed from the pCMV-ICP8 control. B) HumBeta expression from pSLIK2 and pSLIK5. The table indicates the masses of predicted protein products and observed outcomes in these Western Blots. All fusion proteins demonstrated >99% ribosome skipping efficiency, separating reporter fluorescent proteins from synaptases (trace amounts of fusion proteins were only evident when films were greatly overexposed, data not shown).
