# Supplementary material for: Herpes ICP8 protein stimulates homologous recombination in human cells
Source: PLoS One. 2018 Aug 15;13(8):e0200955. doi: 10.1371/journal.pone.0200955 (PMC6093641; doi:10.1371/journal.pone.0200955)
Supplement: S6 Fig — This figure includes recombineering data from all pSLIK cell lines. Recombineering reporter cell lines 293T-Yellow-pSLIK cells were incubated with 1 μg/ml doxycycline to induce synaptase expression and seeded in 24 well plates at 5,000 cells/cm2. The next day, cells were transfected with 50 nM of oligo 85. Fluorescent cells were quantified by flow cytometry and recombination efficiency was calculated by dividing the number of Green cells by the total number of fluorescent cells counted (typically ~50,000 cells per trial). The background of green cells in “no oligo” control experiments (never exceeding 0.02%) was subtracted from recombination frequencies and plotted. Data were evaluated by Fisher’s Exact Test of significance; *** indicates P<0.005 and **** indicates P<0.001. n = 3 and error bars reflect SEM. (PDF) [file pone.0200955.s006.pdf]

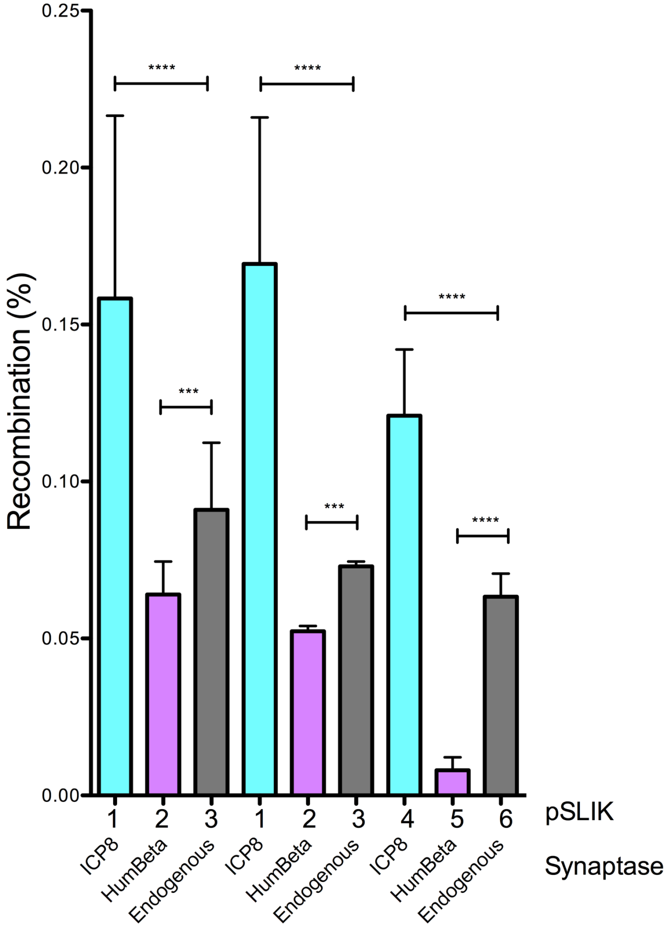

S6 Figure. Gene targeting is stimulated by HHV1 ICP8 and inhibited by phage λ Beta in human cells. This figure includes recombineering data from all pSLIK cell lines. Recombineering reporter cell lines 293T-Yellow-pSLIK cells were incubated with 1 μg/ml doxycycline to induce synaptase expression and seeded in 24 well plates at 5,000 cells/cm2. The next day, cells were transfected with 50 nM of oligo 85. Fluorescent cells were quantified by flow cytometry and recombination efficiency was calculated by dividing the number of Green cells by the total number of fluorescent cells counted (typically ~50,000 cells per trial). The background of green cells in "no oligo" control experiments (never exceeding 0.02%) was subtracted from recombination frequencies and plotted. Data were evaluated by Fisher's Exact Test of significance; *** indicates P<0.005 and **** indicates P<0.001. n=3 and error bars reflect SEM.
