# Supplementary material for: Herpes ICP8 protein stimulates homologous recombination in human cells
Source: PLoS One. 2018 Aug 15;13(8):e0200955. doi: 10.1371/journal.pone.0200955 (PMC6093641; doi:10.1371/journal.pone.0200955)
Supplement: S7 Fig — Recombinant green fluorescent cells from experiment 82 in Fig 2F were enriched by cell sorting. Genomic DNA was isolated and the target gene was amplified by PCR using Taq 2X Master Mix (NEB). For each allele-specific PCR reaction, one of the primers in the pair was complementary at the 3’ end to the template sequence of one of two compared alleles, to specifically amplify that allele sequence. As a control, the same primer pair was used with the other allele’s template. Green primers correspond to oligos 45 and 75 and Yellow primers correspond to oligos 45 and 74. The annealing temperature was optimized to 70.8°C to make amplification selective for each sequence. Reactions were performed as recommended (NEB). Allele-specific PCR shows evidence of genuine Green recombinant allele sequences among non-recombinant Yellow genes in a mixed population. Experimental details for this experiment can be found in S1 Extended Methods under "Determination of recombinant genotype". (PDF) [file pone.0200955.s007.pdf]

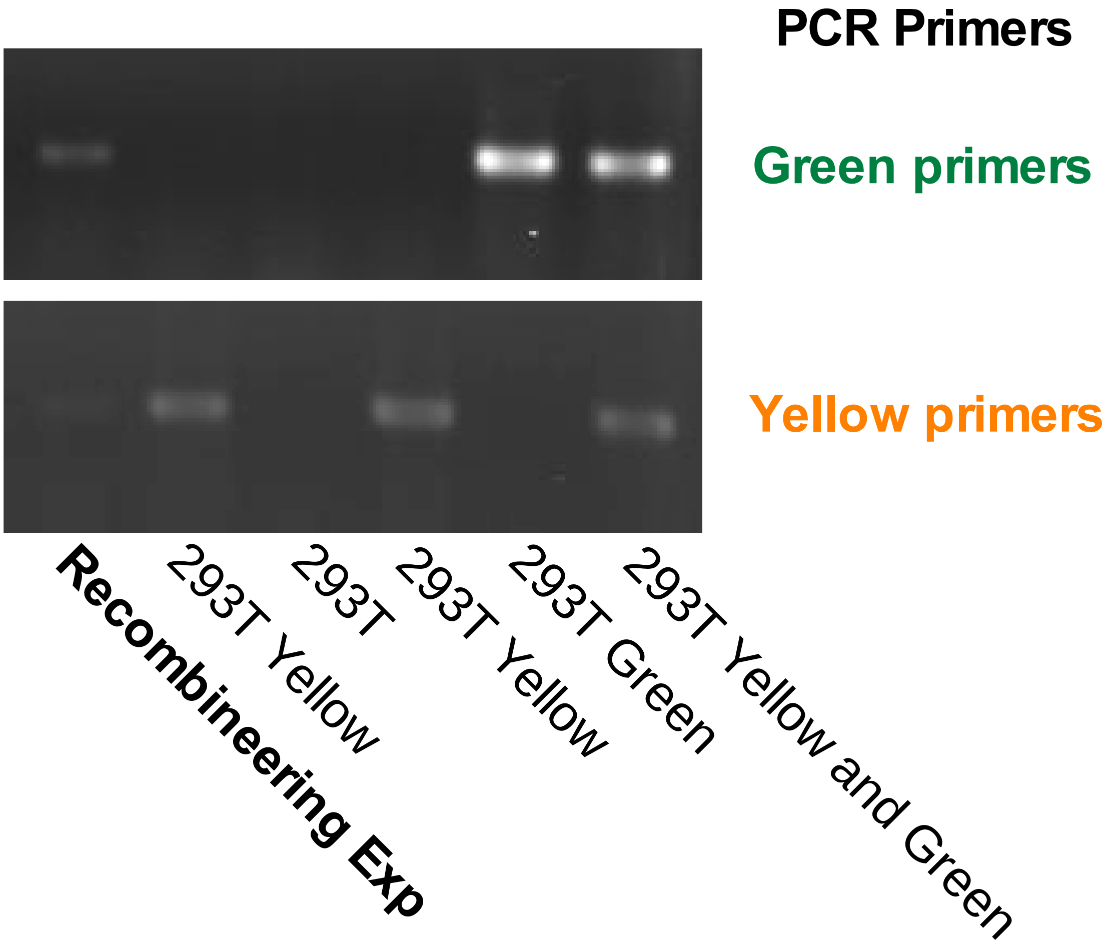

S7 Fig. Recombinant Green genotype detection by allele-specific PCR.
Recombinant green fluorescent cells from experiment 82 in Fig. 2F were enriched by cell sorting. Genomic DNA was isolated and the target gene was amplified by PCR using Taq 2X Master Mix (NEB). For each allele-specific PCR reaction, one of the primers in the pair was complementary at the 3' end to the template sequence of one of two compared alleles, to specifically amplify that allele sequence. As a control, the same primer pair was used with the other allele's template. Green primers correspond to oligos 45 and 75 and Yellow primers correspond to oligos 45 and 74. The annealing temperature was optimized to 70.8°C to make amplification selective for each sequence. Reactions were performed as recommended (NEB). Allele-specific PCR shows evidence of genuine Green recombinant allele sequences among non-recombinant Yellow genes in a mixed population. Experimental details for this experiment can be found in S11 Extended Methods under "Determination of recombinant genotype".
