# Supplementary material for: Herpes ICP8 protein stimulates homologous recombination in human cells
Source: PLoS One. 2018 Aug 15;13(8):e0200955. doi: 10.1371/journal.pone.0200955 (PMC6093641; doi:10.1371/journal.pone.0200955)
Supplement: S1 Table — (PDF) [file pone.0200955.s008.pdf]

S1 Table. Examples of SynExo Functional Modules

Host	Virus	Exonuclease	Synaptase	Reference
E. coli	λ	λ Exo	β	(Cassuto et al. 1971)
E. coli	Rac	RecE	RecT	(Kusano et al. 1994)
B. subtilis	SPP1	Chu	35	(Vellani & Myers 2003; Datta et al. 2008)
M. smegmatis	Che9c	gp60	gp61	(van Kessel & Hatfull 2007)
P. syringae	Rac-like	RecEPsy	RecTPsy	(Swingle et al. 2010)
Winged insects	AcMNPV	Alkaline Exo	LEF-3	(Mikhailov et al. 2003)
H. sapiens	HSV-1	UL12	ICP8	(Reuven et al. 2004; Schumacher et al. 2012)
