# Supplementary material for: Herpes ICP8 protein stimulates homologous recombination in human cells
Source: PLoS One. 2018 Aug 15;13(8):e0200955. doi: 10.1371/journal.pone.0200955 (PMC6093641; doi:10.1371/journal.pone.0200955)
Supplement: S2 Table — (PDF) [file pone.0200955.s009.pdf]

S2 Table. Oligonucleotides used in this study

#	Name	Sequence (5’ to 3’)	Function/Description
**44**	EGFP_N_out	cgtcgccgtccagctcgaccag	To sequence from eGFP 5’ end towards 5’ UTR.
**45**	EGFP_forward	gagcaagggcgaggagctgttc	To sequence eGFP from 5’ end and also used for allele-specific PCR.
**46**	EGFP_reverse	gtccatgccgagagtgatcccg	To sequence eGFP from 3’ end and also used for allele-specific PCR.
**61**	T7_Fw	agatctcgatcccgcgaaattaatac	Oligo to amplify from the pET28a MCS (BglII, T7 promoter, Lac operator, Shine-Dalgrano, His_6, T7 tag).
**62**	T7_Rv	cgcgacccatttgctgtccac	Oligo to amplify from the pET28a MCS (BglII, T7 promoter, Lac operator, Shine-Dalgrano, His_6, T7 tag).
**68**	H67_eGFP	cgtgaccaccctgacccacggcgtgcagtgcttc	Oligo to change eGFP to BFP by site-directed mutagenesis.
**69**	H67_eGFP_R	gaagcactgcacgccgtgggtcagggtggtcacg	Oligo to change eGFP to BFP by site-directed mutagenesis.
**70**	Y204_eGFP	caaccactacctgagctaccagtccgccctgagc	Oligo to change eGFP to YFP by site-directed mutagenesis.
**71**	Y204_eGFP_R	gctcagggcggactggtagctcaggtagtggttg	Oligo to change eGFP to YFP by site-directed mutagenesis.

**74**	3T204eGFP_R	tttgctcagggcggactgggt	Allele-specific PCR to detect "Green".
**75**	3Y204eGFP_R	tttgctcagggcggactggta	Allele-specific PCR to detect "Yellow".
**78**	Y203T-4s/35	gacaaccactacctgtccacccagtccgccctgag	For Recombineering 203T (yellow to green).
**79**	Y203T-4as/35	ctcagggcggactgggtggacaggtagtggttgtc	For Recombineering 203T (yellow to green).
**80**	Y203T-4s/45	tgcccgacaaccactacctgtccacccagtccgccc tgagcaaag	For Recombineering 203T (yellow to green).
**81**	Y203T-4as/45	ctttgctcagggcggactgggtggacaggtagtggtt gtcgggca	For Recombineering 203T (yellow to green).
**82**	Y203T-4s/55	gctgctgcccgacaaccactacctgtccacccagtc cgccctgagcaaagacccc	For Recombineering 203T (yellow to green).
**83**	Y203T-4as/55	ggggtctttgctcagggcggactgggtggacaggta gtggttgtcgggcagcagc	For Recombineering 203T (yellow to green).
**84**	Y203T-4s/65	cccgtgctgctgcccgacaaccactacctgtccacc cagtccgccctgagcaaagaccccaacga	For Recombineering 203T (yellow to green).
**85**	Y203T-4as/65	tcgttggggtctttgctcagggcggactgggtggaca ggtagtggttgtcgggcagcagcacggg	For Recombineering 203T (yellow to green).
**130**	Yellow s/65	cccgtgctgctgcccgacaaccactacctgagctac cagtccgccctgagcaaagaccccaacga	For selfing 203Y to 203Y (yellow to yellow) to control for sequence-specificity of Recombineering.
**131**	Yellow.as/65	tcgttggggtctttgctcagggcggactggtagctcag gtagtggttgtcgggcagcagcacggg	For selfing 203Y to 203Y (yellow to yellow) to control for sequence-specificity of Recombineering.

**190**	pSLIK-att-f	cagggacagcagagatccag	Sequencing of pSLIK, upstream of *att* site, directed toward inserts.
**191**	pSLIK-att-r	gccagatcttgggtgggttaat	Sequencing of pSLIK, downstream of *att* site, directed toward inserts.
**192**	Crimson5-r1	gtgccctcgtagggcttg	Sequencing of pSLIK-Crimson, 5' end of Crimson, directed out.
**193**	Crimson3-r2	tggaacaggtggtggcgg	Sequencing of pSLIK-Crimson, 3' end of Crimson, directed toward its 5' end.
**194**	Crimson5-f1	cactgagaacgtcatcaagcc	Sequencing of pSLIK-Crimson, 5' end of Crimson, directed toward its 3' end.
**195**	HumBeta-r1	ggttcctgtccttcctgtagat	Sequencing of HumBeta, directed out toward sequences 5' to HumBeta.
**196**	HumBeta3-r2	aaacggcggcgaccttc	Sequencing of HumBeta, 3' end, directed toward its 5’ end.
**197**	HumBeta5-f1	atggtgcctcccaagaagaa	Sequencing of HumBeta, 5' end, toward its 3' end.
**198**	HumBeta3-f2	gctgcacatgcaggatctac	Sequencing of HumBeta, directed out toward sequences 3' to HumBeta.
**203**	7-ICP8-Flexi7	aggagcgatcgccatggagacaaagcccaagac g	Forward primer to amplify ICP8 and ICP8-GFP from pCMV-ICP8 and pCMV-ICP8-GFP and ligate to pFN22K

**204**	8-ICP8-Flexi8	gtcggtttaaaccagcatatccaacgtcaggtctc	Reverse primer to amplify ICP8 from pCMV-ICP8 and ligate to pFN22K
**205**	9-ICP8-GFP-Flexi9	gtcggtttaaaccttgtacagctcgtccatgcc	Reverse primer to amplify ICP8-GFP from pCMV-ICP8-GFP and ligate to pFN22K
**214**	214_AluI_eGFP	gctcgccgaccactacca	To amplify Yellow/Green gene between AluI sites
**215**	215_eGFP_AluI	gctcgtccatgccgaga	To amplify Yellow/Green gene between AluI sites
**216**	216_AluI_eGFP_seq	gaccactaccagcagaac	To sequence Yellow/Green gene between AluI sites
