# Supplementary material for: Herpes ICP8 protein stimulates homologous recombination in human cells
Source: PLoS One. 2018 Aug 15;13(8):e0200955. doi: 10.1371/journal.pone.0200955 (PMC6093641; doi:10.1371/journal.pone.0200955)
Supplement: S1 Additional References — (PDF) [file pone.0200955.s010.pdf]

S1 Additional References

Cassuto, E. et al., 1971. Role of exonuclease and beta protein of phage lambda in genetic recombination. V. Recombination of lambda DNA in vitro. *Proc Natl Acad Sci U S A*, 68(7), pp.1639–1643.

Datta, S. et al., 2008. Identification and analysis of recombineering functions from Gram-negative and Gram-positive bacteria and their phages. *Proceedings of the National Academy of Sciences*, 105(5), pp.1626–1631.

Kalderon, D. et al., 1984. A short amino acid sequence able to specify nuclear location. *Cell*, 39(3 Pt 2), pp.499–509.

Kusano, K. et al., 1994. Involvement of RecE exonuclease and RecT annealing protein in DNA double-strand break repair by homologous recombination. *Gene*, 138(1-2), pp.17–25.

Lopes, A. et al., 2010. Detection of novel recombinases in bacteriophage genomes unveils Rad52, Rad51 and Gp2.5 remote homologs. *Nucleic acids research*, 38(12), pp.3952–3962.

McDonald, J.H., 2014. *Handbook of Biological Statistics* 3rd ed., Baltimore, Maryland: Sparky House Publishing. Available at: http://www.biostathandbook.com.

Mikhailov, V.S., Okano, K. & Rohrmann, G.F., 2003. Baculovirus alkaline nuclease possesses a 5“-->3” exonuclease activity and associates with the DNA-binding protein LEF-3. *Journal of virology*, 77(4), pp.2436–2444.

Ploquin, M. et al., 2008. Functional and structural basis for a bacteriophage homolog of human RAD52. *Current biology : CB*, 18(15), pp.1142–1146.

Pluta, K. et al., 2005. Tight control of transgene expression by lentivirus vectors containing second-generation tetracycline-responsive promoters. *The journal of gene medicine*, 7(6), pp.803–817.

Poteete, A.R., 2011. Recombination phenotypes of Escherichia coli greA mutants. *BMC molecular biology*, 12, p.12.

Reuven, N.B., Antoku, S. & Weller, S.K., 2004. The UL12.5 gene product of herpes simplex virus type 1 exhibits nuclease and strand exchange activities but does not localize to the nucleus. *Journal of virology*, 78(9), pp.4599–4608.

Schumacher, A.J. et al., 2012. The HSV-1 exonuclease, UL12, stimulates recombination by a single strand annealing mechanism. *PLoS pathogens*, 8(8), p.e1002862.

Shin, K.-J. et al., 2006. A single lentiviral vector platform for microRNA-based conditional RNA interference and coordinated transgene expression. *Proc Natl Acad Sci U S A*, 103(37), pp.13759–13764.

Strack, R.L. et al., 2009. A rapidly maturing far-red derivative of DsRed-Express2 for whole-cell labeling. *Biochemistry*, 48(35), pp.8279–8281.

Swingle, B. et al., 2010. Recombineering using RecTE from Pseudomonas syringae. *Applied and environmental microbiology*, 76(15), pp.4960–4968.

Szymczak-Workman, A.L., Vignali, K.M. & Vignali, D.A.A., 2012. Design and construction of 2A peptide-linked multicistronic vectors. *Cold Spring Harbor Protocols*, 2012(2), pp.199–204.

Taylor, T.J. & Knipe, D.M., 2003. C-terminal region of herpes simplex virus ICP8 protein needed for intranuclear localization. *Virology*, 309(2), pp.219–231.

Taylor, T.J. et al., 2003. Herpes simplex virus replication compartments can form by coalescence of smaller compartments. *Virology*, 309(2), pp.232–247.

Valledor, M. et al., 2012. Fluorescent protein engineering by in vivo site-directed mutagenesis. *IUBMB life*, 64(8), pp.684–689.

van Kessel, J.C. & Hatfull, G.F., 2007. Recombineering in Mycobacterium tuberculosis. *Nature Methods*, 4(2), pp.147–152. Available at: http://www.nature.com/nmeth/journal/v4/n2/abs/nmeth996.html.

Vellani, T.S. & Myers, R.S., 2003. Bacteriophage SPP1 Chu is an alkaline exonuclease in the SynExo family of viral two-component recombinases. *J Bacteriol*, 185(8), pp.2465–2474. Available at: http://www.ncbi.nlm.nih.gov/pmc/articles/PMC152610/.

White, M.F., 2011. Homologous recombination in the archaea: the means justify the ends. *Biochemical Society transactions*, 39(1), pp.15–19.
