# Supplementary material for: Herpes ICP8 protein stimulates homologous recombination in human cells
Source: PLoS One. 2018 Aug 15;13(8):e0200955. doi: 10.1371/journal.pone.0200955 (PMC6093641; doi:10.1371/journal.pone.0200955)
Supplement: S1 Extended Methods — (PDF) [file pone.0200955.s011.pdf]

S1 Extended Methods

Cell culture conditions

293T cells were grown in Dulbecco's modified Eagle's medium (DMEM)-high glucose medium with 5% fetal bovine serum (FBS) and antibiotics–antimycotics (50 U/mL penicillin G, 2.5 µg/mL amphotericin B, and 10 I.U./mL streptomycin) at 37 °C in 5 % atmospheric CO_2. The pSLIK 293T-Yellow cell lines were grown as described for 293T except using 5-10% Tet-free FBS (Clontech, 631106) in the presence of 100 µg/ml zeocin (Zeo).

Plasmids

pCMV-ICP8 was kindly provided by Dr. David Knipe at Harvard. ICP8 from pCMV-ICP8 (Taylor & Knipe 2003) and synthetic Beta synaptase genes were individually cloned into the pSLIK-Zeo lentivirus vectors (Shin et al. 2006). The synaptase genes were fused to the E2-Crimson fluorescent protein gene (Strack et al. 2009) through a P2A linker (Szymczak-Workman et al. 2012) to follow synaptase expression without tethering E2-Crimson to the synaptase protein (S3 Figure). The coding sequence of ICP8 from pCMV-ICP8 was used to produce pSLIK1 (E2-Crimson N-terminus) and pSLIK4 (E2-Crimson C-terminus). The bacteriophage lambda Beta gene was redesigned to be better expressed in human cells and fused to a nuclear localization signal (NLS) (Kalderon et al. 1984) and an HA epitope tag. The resulting "humanized" Beta (hereafter referred to as HumBeta) coding sequence was used to create pSLIK2 (E2-Crimson N-terminus) and pSLIK5 (E2-Crimson C-terminus). Isogenic synaptase-free control vectors were also built to evaluate recombination using endogenous functions: pSLIK3 for E2-Crimson N-terminus and pSLIK6 for E2-Crimson C-terminus.

Construction of pSLIK lentiviral plasmids for doxycycline-inducible expression of viral synaptases.

pSLIK lentiviral plasmid constructions 1, 2, 4 and 5 were created by fusing viral synaptase genes (ICP8 and Beta) to the red fluorescent protein gene E2-Crimson (Strack et al. 2009) through a P2A linker (Szymczak-Workman et al. 2012) in single open reading frames (S3 Figure). The synaptase-P2A/ Crimson fusions and "no synaptase" controls (pSLIK3 and 6) were cloned downstream of a minimal CMV promoter controlled by a Tet ON operator (Pluta et al. 2005) and spliced into a pSLIK-Zeo backbone ((Shin et al. 2006); Addgene plasmid 25736). Following construction of six pSLIK plasmids (N- and C-terminal fusions for each synaptase plus controls), lentiviral plasmids were expanded in *E. coli* Stbl3 cells (Life Technologies) and endotoxin-free DNA purified for each before production of lentiviruses in 293T cells. The ICP8 and HumBeta synaptase genes were individually cloned into the pSLIK-Zeo lentivirus vector for high transduction efficiency, selection and inducibility (Shin et al. 2006). The pSLIK-Zeo lentivirus expresses the rTA transactivator from a ubiquitin promoter. The synaptases were placed under the expression of a minimal cytomegalovirus (CMV) promoter controlled by rTA transactivator acting at multiple TetO operators (Pluta et al. 2005) to direct their inducible transcription in the presence of

doxycycline and the rTA transactivator. Since the rTA transactivator is expressed constitutively in *cis* to the cloned genes, expression of the synaptases is only limited by the diffusion rate of doxycycline from the growth medium into the nucleus, facilitating time-course experiments. Maps and DNA sequences are in S13 DNA Sequences.

N-terminal Crimson/P2A fusion plasmid construction details

Crimson/P2A-ICP8 (pSLIK1)

ICP8 was amplified from pCMV-ICP8 (Taylor & Knipe 2003) using a primer that introduced an AsiSI site at the 5' end of ICP8 to put it in frame with the HaloTag® (Promega) and with a second primer containing a PmeI site engineered at the 3' end of ICP8 which adds one more codon before the TAA stop (primers: ICP8-Flexi7 and ICP8-Flexi8). The product was digested with a mix of AsiSI and PmeI and ligated to pFN22K (Promega) also cut with AsiSI and PmeI. The ligation reaction removes the toxic barnase gene. Kanamycin resistant (Kan^R) clones were selected and screened for inserts. One sequence-verified clone was named pFN22K::ICP8.

E2-Crimson was amplified from pTEC19 (created by Lalita Ramakrishnan, Addgene plasmid 30178) with primers 145 and 146. One ng of the 742 bp PCR product was used as the template for a second PCR using phosphorylated primers 147 and 148 to incorporate the P2A (Szymczak-Workman et al. 2012) sequence and to phosphorylate the 5’ ends to make them suitable for ligation. The final PCR product of 795 bp was purified by gel extraction and was named “Product N”. pFN24K (Promega) was cut with BsaAI, treated with alkaline phosphatase, and the 2477 bp vector backbone was purified by gel extraction. 50 ng of the dephosphorylated vector was ligated to 100 ng “Product N”. Kanamycin resistant clones were selected, screened for red fluorescence by microscopy, and validated by AsiSI restriction analysis. One clone named pFN24K::Crimson/P2A expresses E2-Crimson from the phage T7 promoter and can release a 779 bp insert when cleaved with AsiSI.

To fuse Crimson/P2A to the N-terminus of ICP8, Crimson/P2A was released from pFN24K-Crimson/P2A by AsiSI restriction and gel purified. pFN22K::ICP8 was cleaved by AsiSI and treated with alkaline phosphatase. Dephosphorylated pFN22K::ICP8/AsiSI and Crimson/P2A/AsiSI were ligated, Kan^R clones were selected, and correct clones identified by digestion with BglII and EcoRI. One clone was named pFN22K-Crimson/P2A-ICP8 and can release a 4376 bp Crimson/P2A-ICP8 insert when cleaved with BglII and EcoRI.

To create the Gateway (Life Technologies) entry vector for Crimson/P2A-ICP8, Crimson/P2A-ICP8 was released from pFN22K-Crimson/P2A-ICP8 with BglII and EcoRI-HF and the 4376 bp product was ligated to pENTR2B/TREPitt (a generous gift of Jakob Reiser) that had been previously cleaved by BamHI and EcoRI-HF. Kan^R clones were selected, and correct clones identified by digestion with EcoRI and AsiSI. One 7061 bp Gateway entry clone was named pENTR2B/TREPitt::Crimson/P2A-ICP8 and used to transfer Crimson/P2A-ICP8 to pSLIK-Zeo ((Shin et al. 2006); Addgene plasmid 25736) using the Gateway

LR clonase II reaction (Life Technologies). The products of site-specific recombination were used to transform NEB 5α (New England Biolabs) to Amp^R and clones were screened by restriction digestion with EcoRI. One 16133 bp recombinant lentiviral plasmid was named pSLIK/TREPitt::Crimson/P2A-ICP8 and subsequently renamed pSLIK1 for brevity. pSLIK1 was used to transform Stbl3 chemically competent cells (Life Technologies) for higher yield of the lentivirus vector than from NEB5α. The pSLIK1 lentivirus vector was isolated by endotoxin-free maxiprep (Qiagen) and submitted for sequencing to Genewiz. The sequence so obtained confirmed the entire sequence of the insert between the recombined *att* site in the pSLIK-Zeo vector.

Crimson/P2A-NLS/HA-HumBeta (pSLIK2)

"Humanized" Beta (HumBeta) was designed *in silico* using codon optimization software (Genewiz) and additional sequence analysis to modify the enterobacteria phage λ Beta gene (*bet*; NCBI gi: 9626243) to include an N-terminal Nuclear Localization Signal (NLS) sequence from the SV40 large T antigen (http://www.uniprot.org/uniprot/P03070), an Influenza virus hemagglutinin epitope (HA) tag (http://en.wikipedia.org/wiki/HA-tag) and then a linker (GGGGGSGGGGGSGGGS) to reduce steric hindrance of Beta protein. The resulting predicted fusion protein sequence is:

MVPPKKKRKVEDPKYPYDVPDYAGGGGGSGGGGGSGGGSMSTALATLAGKLAERVGMDSVDPQELIT
TLRQTAFKGDASDAQFIALLIVANQYGLNPWTKEIYAFPDKQNGIVPVVGVDGWSRIINENQQFDGMDFE
QDNESCTCRIYRKDRNHPICVTEWMDECRREPFKTREGREITGPWQSHPKRMLRHKAMIQCARLAFGF
AGIYDKDEAERIVENTAYTAERQPERDITPVNDETMQEINTLLIALDKTWDDDLLPLCSQIFRRDIRASSELT
QAEAVKALGFLKQKAAEQKVAAV.

Restriction sites were added to the end of the sequence to facilitate cloning. Genewiz synthesized the DNA and ligated it into plasmid pUC57-Kan to form pUC57-Kan::NLS/HA-HumBeta. The entire plasmid sequence was verified by Genewiz. NLS/HA-HumBeta was released from pUC57-Kan::NLS/HA-HumBeta by cleavage with HindIII and EcoRI and ligated to pUC19(delta-KpnI) that had been previously digested with HindIII and EcoRI and treated with alkaline phosphatase. Amp^R clones were validated with EcoRI digestion and one that produced a 3560 linear product was named pUC19::NLS/HA-HumBeta. This plasmid places expression of HumBeta under control of P_{Lac}/ O_{Lac}/LacI/IPTG, a property that was used in screens for E2-Crimson fusions to HumBeta.

To fuse Crimson/P2A to the N-terminus of HumBeta, Crimson/P2A was released from pFN24K-Crimson/P2A by PciI restriction and gel purified. pUC19ΔKpnI::NLS/HA-HumBeta was linearized with NcoI and treated with alkaline phosphatase. Dephosphorylated pUC19ΔKpnI::NLS/HA-HumBeta/NcoI and Crimson/P2A/ PciI were ligated, Amp^R clones were selected, and correct clones identified by digestion with BglII and EcoRI. One clone was named pUC19::Crimson/P2A-NLS/HA-HumBeta and can release a 1673 bp insert when cleaved with BglII and EcoRI. NEB5α cultures containing pUC19::Crimson/P2A-NLS/HA-HumBeta appear blue under standard fluorescent lab room lighting,

reflecting the absorbance spectrum of the strongly expressed E2-Crimson fluorescent protein, and are highly fluorescent when colonies are viewed on a Dark Reader (Clare Chemical) imaging box.

To create the entry vector for Crimson/P2A-NLS/HA-HumBeta, Crimson/P2A-NLS/HA-HumBeta was released from pUC19::Crimson/P2A-NLS/HA-HumBeta with BglII and EcoRI-HF and the 1673 bp product was ligated to pENTR2B/TREPitt that had been previously cleaved by BamHI and EcoRI-HF. Kan^R clones were selected, and correct clones identified by digestion with EcoRI. One 4358 bp entry clone was named pENTR2B/TREPitt::Crimson/P2A-NLS/HA-HumBeta, used to transfer Crimson/P2A-NLS/HA-HumBeta to pSLIK-Zeo, and identified as described above. One 13617 bp recombinant lentiviral plasmid was named pSLIK/TREPitt:: Crimson/P2A-NLS/HA-HumBeta and subsequently renamed pSLIK2 for brevity. pSLIK2 was used to transform Stbl3 cells, isolated by endotoxin-free maxiprep (Qiagen) and submitted for sequencing to Genewiz. The sequence so obtained confirmed the entire sequence of the insert between the recombined *att* site in the pSLIK-Zeo vector.

Crimson/P2A-Control (pSLIK3)

As a no synaptase control, the 795 bp “Product N” described above was used as template for PCR with phosphorylated primers 145 and 149 to create the 784 bp “Product N-control” and then purified by gel extraction. pFN24K was cut with BsaAI, treated with alkaline phosphatase, and the 2477 bp product purified by gel extraction. Dephosphorylated pFN24K/BsaAI and PCR “Product N-control” were ligated and used to transform NEB5α cells. Kan^R clones were screened for red fluorescence by microscopy and validated by BglII and EcoRI restriction analysis. One plasmid isolate named pFN24K::Crimson/P2A-Control is 3261 bp, expresses E2-Crimson from the T7 promoter, and can release a 768 bp insert when cleaved with with BglII and EcoRI.

To create the entry vector for Crimson/P2A-Control, Crimson/P2A-Control was released from pFN24K::Crimson/P2A-Control with BglII and EcoRI-HF and the 768 bp product was ligated to pENTR2B/TREPitt that had been previously cleaved by BamHI and EcoRI-HF. Kan^R clones were selected, and correct clones identified by digestion with EcoRI and independently by digestion with AsiSI and NcoI. One 3453 bp entry clone was named pENTR2B/TREPitt::Crimson/P2A-Control, used to transfer Crimson/P2A-NLS/HA-Control to pSLIK-Zeo, and identified as described above. One 12712 bp recombinant lentiviral plasmid was named pSLIK/TREPitt:: Crimson/P2A-Control and subsequently renamed pSLIK3 for brevity. pSLIK3 was used to transform Stbl3 cells, isolated by endotoxin-free maxiprep (Qiagen) and submitted for sequencing to Genewiz. The sequence so obtained confirmed the entire sequence of the insert between the recombined *att* site in the pSLIK-Zeo vector.

C-terminal P2A/Crimson fusion plasmid construction details

ICP8-P2A/Crimson (pSLIK4)

E2-Crimson was amplified from pTEC19 with primers 150 and 151. One ng of the resulting PCR product (717 bp) was used as template for a second PCR using phosphorylated primers 152 and 151 to incorporate the P2A (Szymczak-Workman et al. 2012) sequence and to phosphorylate the 5' ends to make them suitable for ligation. The final PCR product of 746 bp was purified by gel extraction and was named "Product C". pFN22K::ICP8 was cut with PmeI, treated with alkaline phosphatase, and ligated to "Product C". Kan^R clones were screened by BamHI and by fluorescence when cells were treated with IPTG. One 8158 bp plasmid was named pFN22K::ICP8-P2A/Crimson. To create the entry vector for ICP8-P2A/Crimson, pFN22K::ICP8-P2A/Crimson was cut with AsiSI. The AsiSI sticky ends were made blunt by a fill-in reaction with T4 DNA Polymerase. The plasmid was then cut with EcoRI-HF to release the ICP8-P2A/Crimson insert of 4348 bp, which was then gel purified. Likewise, pENTR2B/TREPitt was cut with BamHI and the ends were blunted as described above and then cut with EcoRI-HF, treated with alkaline phosphatase, and the 2685 bp product was gel purified. Dephosphorylated pENTR2B/TREPitt/BamHI/Blunted/EcoRI and the pFN22K::ICP8-P2A/Crimson/AsiSI/Blunted/EcoRI 4348 bp insert were ligated, used to transform NEB5α to Kan^R, and clones were confirmed by EcoRI and PstI restriction analysis. One 7037 bp entry clone was named pENTR2B/TREPitt::ICP8-P2A/Crimson, used to transfer ICP8-P2A/Crimson to pSLIK-Zeo, and identified as described above. One 16297 bp recombinant lentiviral plasmid was named pSLIK/TREPitt::ICP8-P2A/Crimson and subsequently renamed pSLIK4 for brevity. pSLIK4 was used to transform Stbl3 cells, isolated by endotoxin-free maxiprep (Qiagen) and submitted for sequencing to Genewiz. The sequence so obtained confirmed the entire sequence of the insert between the recombined *att* site in the pSLIK-Zeo vector.

NLS/HA-HumBeta-P2A/Crimson (pSLIK5)

pUC19::NLS/HA-HumBeta was cut with PmeI, treated with alkaline phosphatase, ligated to "Product C", and used to transform NEB5α cells. Amp^R clones were screened by SnaBI and EcoRI and by fluorescence when cells were treated with IPTG. One 4306 bp plasmid was named pUC19::NLS/HA-HumBeta-P2A/Crimson and can release a 1663 bp insert when cleaved with SnaBI and EcoRI. To create the entry vector for NLS/HA-HumBeta-P2A/Crimson, NLS/HA-HumBeta-P2A/Crimson was released from pUC19ΔKpnI-Kan::NLS/HA-HumBeta-P2A/Crimson with SnaBI and EcoRI and the 1663 bp product was ligated to dephosphorylated pENTR2B/TREPitt/BamHI/Blunted/EcoRI as described above. Kan^R NEB5α clones were selected, and correct clones identified by digestion with EcoRI and PstI. One 4358 bp entry clone named pENTR2B/TREPitt::NLS/HA-HumBeta-P2A/Crimson was used to transfer NLS/HA-HumBeta-P2A/Crimson to pSLIK-Zeo, and identified as described above. One 13612 bp recombinant lentiviral plasmid was named pSLIK/TREPitt::NLS/HA-HumBeta-P2A/Crimson and subsequently renamed pSLIK5 for brevity. pSLIK5 was used to transform Stbl3 cells, isolated by endotoxin-free maxiprep

(Qiagen) and submitted for sequencing to Genewiz. The sequence so obtained confirmed the entire sequence of the insert between the recombined *att* site in the pSLIK-Zeo vector.

P2A/Crimson-Control (pSLIK6)

As a no synaptase control, a P2A/Crimson-Control sequence was amplified from "Product C" with phosphorylated primers 153 and 154. The resulting PCR product of 787 bp was purified by gel extraction and named "Product C-Control". pFN24K was cut with BsaAI, treated with alkaline phosphatase, the 2477 bp product ligated to "Product C-Control" and used to transform NEB5α cells. Kan^R clones were confirmed for red fluorescence by microscopy and by BglII and EcoRI restriction analysis. A confirmed plasmid of 3264 bp was named pFN24K::P2A/Crimson-Control and can release a 771 bp insert when cleaved with BglII and EcoRI.

The 771 bp P2A/Crimson-Control insert was released from pFN24K::P2A/Crimson-Control with BglII and EcoRI-HF and ligated to dephosphorylated pENTR2B/TREPitt/BamHI/Blunted/EcoRI as described above. Kan^R NEB5α clones were selected, and correct clones identified by digestion with EcoRI and PstI. One 3456 bp entry clone named pENTR2B/TREPitt::P2A/Crimson-Control was used to transfer NLS/HA-HumBeta-P2A/Crimson to pSLIK-Zeo, and identified as described above. One 12715 bp recombinant lentiviral plasmid was named pSLIK/TREPitt::P2A/Crimson-Control and subsequently renamed pSLIK6 for brevity. pSLIK6 was used to transform Stbl3 cells, isolated by endotoxin-free maxiprep (Qiagen) and submitted for sequencing to Genewiz. The sequence so obtained confirmed the entire sequence of the insert between the recombined *att* site in the pSLIK-Zeo vector.

Lentiviral transduction protocol

293T-Yellow recombineering reporter cell lines were created by transducing 293T cells with pDual-eGFP(Y203) as previously described (Valledor et al. 2012). Briefly, 2 x 10^6 293T cells were plated on 10 cm^2 dishes. The next day, 4 µg of pDual-eGFP(Y203), 4 µg of pHR'8.2ΔR and 0.4 µg of pCMV-VSV-G were mixed with 24 µl of Fugene 6 and 400 µl DMEM, incubated for 15-30 minutes (min) at room temperature, and then added to the 293T cells. Cells were incubated overnight in a BL2+ incubator at 37 °C in 5% atmospheric CO_2. 24 hours later the medium was changed. The following day, media containing transducing particles were collected, filtered with 0.45 µm syringe filters, treated with DNase I for 30 min at 37 °C, and then added to growing 293T cells. Cells were incubated with transducing particles overnight. Transduction efficiency was determined to be 93% as assessed by flow cytometry. The pool of transduced cells were then expanded, named 293T-Yellow, and used for recombineering assays.

For Inducible Synaptase-Crimson cell lines, 293T-Yellow cells were transduced with pSLIK-derived lentiviral particles prepared as described above. Cells were incubated with transducing particles for 8 hours in a BL2+ incubator at 37 °C in 5% atmospheric CO_2 to minimize multiple lentiviral insertions. Pools of transduced cells were selected by culturing the cells in 100 µg/ml Zeo for a week.

Doxycycline-inducible expression of viral synaptases from pSLIK lentiviral vectors

Treating cells bearing pSLIK vectors with 1 μg/ml doxycycline induced both ICP8 and HumBeta expression (S4 Figure). In the pSLIK vectors, expression of the Zeocin resistance gene (Zeo) is coupled to expression of the doxycycline-activated rTA transactivator. Selection for Zeo^R enriches for cells that express synaptase genes. In titrating [Zeo] in media, it was evident that levels of the Crimson reporter (stoichiometrically expressed with synaptase) was positively correlated with Zeo concentration (data not shown). An advantage of the lentiviral system was that by using the P2A/Crimson reporter, it was possible to evaluate how many cells expressed the synaptases before and after induction without having to estimate from parallel experiments.

Fluorescence spectroscopy

Fluorescence spectra shown in Fig 2 panel B were collected from protein extracts from *E. coli* Rosetta-gami™2 cells (Novagen) transformed with plasmids pDual-eGFP and pDual-eGFP(Y203) independently. Protein expression was induced with 1 mM IPTG. Fluorescent proteins were purified and their fluorescence spectra evaluated as described in (Valledor et al. 2012) and presented in S12 Data.

Flow cytometry analysis

Cells were harvested, washed and resuspended in 0.5 ml PBS. Cells were vortexed and filtered just before analysis using the BD Accuri flow cytometer. 50,0000 cells were evaluated by flow for evidence of recombination. Green recombinants were distinguished from Yellow parental cells using narrow bandpass filters (510 ± 15 nm and 540 ± 10 nm) and a differential angular distribution that allowed quantification of each cell without compensation. Dark, Green and Yellow gates were set using untransduced 293T, 293T-eGFP and 293T-Yellow cells respectively (Fig 2 panels C-E). Green fluorescence from cells expressing GFP was quantified using a 530 ± 15 nm filter. Cells expressing E2-Crimson were quantified using a 675 ± 25 filter. Data from the Accuri were analysed using BD Accuri C6 Analysis software, Excel and GraphPad PRISM. Green recombinants were enriched by sorting with the BD LSR-Fortessa-HTS cytometer using a 488 nm blue laser and narrow bandpass filters (510 ± 10 nm for eGFP and 550 ± 15 nm for $eGFP^{Y204}$) combined with dichroic mirrors (Q495lpxr_11.25 and Q525lpxr_11.25, respectively). Data were processed using Facs Diva 6.1.3 and FlowJo software (Tree Star). The geometric mean fluorescence intensity values were used to compare fluorescence between different samples.

Determination of recombinant genotype

Gene conversion rates in recombination experiments were measured phenotypically by using flow cytometry to enumerate Green (putative recombinant) and Yellow (nonrecombinant) fluorescent cells. Since recombinant cells appeared at a low frequency within the culture (S6 Figure), it was necessary to enrich for the recombinant population for genotyping studies. Cells from recombination experiment 82 (Fig 2 panel F, pSLIK1 + Dox + oligo85) were sorted for Green fluorescence, enriching the Green population from 0.1 % to 39 %. To verify that the predicted recombinant genotype was evident in phenotypically recombinant cells, two methods were employed: allele specific PCR (S7 Figure) and

sequencing of the target region (Fig 4 panel B). Allele-specific PCR was performed using genomic DNA from sorted cells with primers 45 and 74 to uniquely detect the Green allele and 45 and 75 to uniquely detect the Yellow allele at an annealing temperature of 70.8°C. For sequencing the target region, genomic DNA from sorted cells was extracted and PCR amplified using oligos 214 and 215. Since cell sorting enriched the recombinants to 39% of the total population, there was still a large amount of cells bearing a Yellow allele along with Green. As acquisition of the Green allele destroyed an AluI site present in the nonrecombinant parental Yellow allele, PCR products treated with AluI were resolved by agarose gel electrophoresis and the non-cut band was excised from the gel and purified. Gel purified restricted PCR product was sent for sequencing (GeneWiz) using oligo 216.

Statistical methods

Multiple independent gene targeting experiments were performed to address specific questions. In general, 50,000 cells were interrogated by flow cytometry for their fluorescence phenotype. In these experiments, n was typically 3 but occasionally much higher (*e.g.* in Fig 3, n=12). To assess significance, Fisher's Exact Test was performed with pairwise comparisons of Green and Yellow cell numbers between conditions using an application found here: http://www.langsrud.com/fisher.htm. When the minority population exceeded 1000 cells, Chi-square tests of independence were employed using the chiind.xls Excel spreadsheet calculator (McDonald 2014). When compared, both approaches provided concordant conclusions of level of significance. On occasion, Student's T-tests and one-way ANOVA were also evaluated (in GraphPad PRISM) and were concordant, however the data were heteroscedastic as the variances in the ICP8 samples were much greater than in the HumBeta and Endogenous samples. This is consistent with the observation that 1) viability of ICP8 expressing lines was lower than the HumBeta and control lines; 2) the number of Crimson (and therefore ICP8) expressing cells was at least 10x less than those of HumBeta and Endogenous lines. Heteroscedasticity does not effect Fisher and chi-square, so Fisher's test was the preferred method. Two-tail P values were reported, but examination of the one-tailed values provided insight into the tendency of recombination to be stimulated (ICP8) or inhibited (HumBeta).
