# Supplementary material for: Herpes ICP8 protein stimulates homologous recombination in human cells
Source: PLoS One. 2018 Aug 15;13(8):e0200955. doi: 10.1371/journal.pone.0200955 (PMC6093641; doi:10.1371/journal.pone.0200955)
Supplement: S1 DNA Sequences — (PDF) [file pone.0200955.s013.pdf]

Plasmids used in construction of pSLIK1

>pFN24K::Crimson/P2A

AKA pFN24K::Product N.

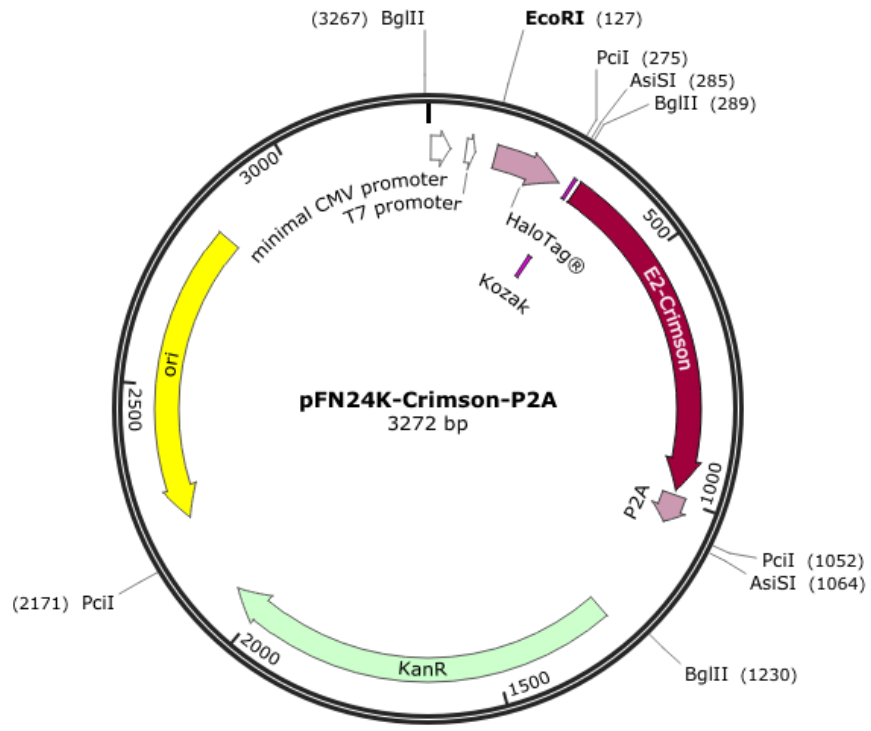

>pFN24K::Crimson/P2A

ATGGGCGGTAGGCGTGTACGGTGGGAGGTCTATATAAGCAGAGCTGGTTTAG

TGAACCGTCAGATCACTAGAAGCTTAATACGACTCACTATAGGGTACGATTT

AGGTGACACTATAGAATAAGGAGAATTCATCATGGCAGAAATCGGTACTGGC

TTTCCATTCGACCCCCATTATGTGGAAGTCCTGGGCGAGCGCATGCACTACGT

CGATGTTGGTCCGCGCGATGGCACCCCTGTGCTGTTCCTGCACGGTAACCCGA

CCTCCTCCTACAACATGTGCGATCGCAGATCTAGGAGGTCCGCCACCATGGA

TAGCACTGAGAACGTCATCAAGCCCTTCATGCGCTTCAAGGTGCACATGGAG

GGCTCCGTGAACGGCCACGAGTTCGAGATCGAGGGCGTGGGCGAGGGCAAG

CCCTACGAGGGCACCCAGACCGCCAAGCTGCAAGTGACCAAGGGCGGCCCC

CTGCCCTTCGCCTGGGACATCCTGTCCCCCCAGTTCTTCTACGGCTCCAAGGC

GTACATCAAGCACCCCGCCGACATCCCCGACTACCTCAAGCAGTCCTTCCCC

GAGGGCTTCAAGTGGGAGCGCGTGATGAACTTCGAGGACGGCGGCGTGGTG

ACCGTGACCCAGGACTCCTCCCTGCAGGACGGCACCCTCATCTACCACGTGA

AGTTCATCGGCGTGAACTTCCCCTCCGACGGCCCCGTAATGCAGAAGAAGAC

TCTGGGCTGGGAGCCCTCCACTGAGCGCAACTACCCCCGCGACGGCGTGCTG

AAGGGCGAGAACCACATGGCGCTGAAGCTGAAGGGCGGCGGCCACTACCTG

TGTGAGTTCAAGTCCATCTACATGGCCAAGAAGCCCGTGAAGCTGCCCGGCT

ACCACTACGTGGACTACAAGCTCGACATCACCTCCCACAACGAGGACTACAC

CGTGGTGGAGCAGTACGAGCGCGCCGAGGCCCGCCACCACCTGTTCCAGGGA

TCCGGAGCCACGAACTTCTCTCTGTTAAAGCAAGCAGGAGACGTGGAAGAAA

ACCCCGGTCCTCACATGTCTGCGATCGCTGTGTTCCGCTTCCTTTAGCAGCCC

TTGCGCCCTGAGTGCTTGCGGCAGCGTGAGCTTCAAAAGAATTGCCAGCTGG

GGCGCCCTCTGGTAAGGTTGGGAAGCCCTGCAAAGTAAACTGGATGGCTTTC

TTGCCGCCAAGGATCTGATGGCGCAGGGGATCAAGATCTGATCAAGAGACAG

GATGACGGTCGTTTCGCATGCTTGAACAAGATGGATTGCACGCAGGTTCTCC

GGCCGCTTGGGTGGAGAGGCTATTCGGCTATGACTGGGCACAACAGACAATC

GGCTGCTCTGATGCCGCCGTGTTCCGGCTGTCAGCGCAGGGGCGCCCGGTTCT

TTTTGTCAAGACCGACCTGTCCGGTGCCCTGAATGAACTGCAGGACGAGGCA

GCGCGGCTATCGTGGCTGGCCACGACGGGCGTTCCTTGCGCAGCTGTGCTCG

ACGTTGTCACTGAAGCGGGAAGGGACTGGCTGCTATTGGGCGAAGTGCCGGG

GCAGGATCTCCTGTCATCTCACCTTGCTCCTGCCGAGAAAGTATCCATCATGG

CTGATGCAATGCGGCGGCTGCATACGCTTGATCCGGCTACCTGCCCATTCGAC

CACCAAGCGAAACATCGCATCGAGCGAGCACGCACTCGGATGGAAGCCGGT

CTTGTCGATCAGGATGATCTGGACGAAGAGCATCAGGGGCTCGCGCCAGCCG

AACTGTTCGCCAGGCTCAAGGCGCGTATGCCGGATGGTGAGGATCTCGTCGT

GACTCATGGCGATGCCTGCTTGCCGAATATCATGGTGGAAAATGGCCGCTTTT

CTGGATTCATCGACTGTGGCCGGCTGGGTGTGGCGGACCGCTATCAGGACAT

AGCGTTGGCTACCCGTGATATTGCTGAAGAGCTTGGCGGCGAATGGGCTGAC

CGCTTCCTCGTGCTTTACGGTATCGCCGCTCCCGATTCGCAGCGCATCGCCTT

CTATCGCCTTCTTGACGAGTTCTTCTGAGCGGGACTCTGGGGTTCGAAATGAC

CGACCAAGCGACGCCCAACCGGTATCAGCTCACTCAAAGGCGGTAATACGGT

TATCCACAGAATCAGGGGATAACGCAGGAAAGAACATGTGAGCAAAAGGCC

AGCAAAAGGCCAGGAACCGTAAAAAGGCCGCGTTGCTGGCGTTTTTCCATAG

GCTCCGCCCCCCTGACGAGCATCACAAAAATCGACGCTCAAGTCAGAGGTGG

CGAAACCCGACAGGACTATAAAGATACCAGGCGTTTCCCCCTGGAAGCTCCC

TCGTGCGCTCTCCTGTTCCGACCCTGCCGCTTACCGGATACCTGTCCGCCTTTC

TCCCTTCGGGAAGCGTGGCGCTTTCTCATAGCTCACGCTGTAGGTATCTCAGT

TCGGTGTAGGTCGTTCGCTCCAAGCTGGGCTGTGTGCACGAACCCCCCGTTCA

GCCCGACCGCTGCGCCTTATCCGGTAACTATCGTCTTGAGTCCAACCCGGTAA

GACACGACTTATCGCCACTGGCAGCAGCCACTGGTAACAGGATTAGCAGAGC

GAGGTATGTAGGCGGTGCTACAGAGTTCTTGAAGTGGTGGCCTAACTACGGC

TACACTAGAAGGACAGTATTTGGTATCTGCGCTCTGCTGAAGCCAGTTACCTT

CGGAAAAAGAGTTGGTAGCTCTTGATCCGGCAAACAAACCACCGCTGGTAGC

GGTGGTTTTTTTGTTTGCAAGCAGCAGATTACGCGCAGAAAAAAAGGATTTC

AAGAAGATCCTTTGATCTTTTCTACGGGGTCTGACGCTCAGTGGAACGAAAA

CTCACGTTAAGGGATTTTGGTCATGAGATTATCAAAAAGGATCTTCACCTAGA

TCCTTTTATAGTCCGGAAATACAGGAACGCACGCTGGATGGCCCTTCGCTGG

GATGGTGAAACCATGAAAAATGGCAGCTTCAGTGGATTAAGTGGGGGTAATG

TGGCCTGTACCCTCTGGTTGCATAGGTATTCATACGGTTAAAATTTATCAGGC

GCGATTGCGGCAGTTTTTCGGGTGGTTTGTTGCCATTTTTACCTGTCTGCTGCC

GTGATCGCGCTGAACGCGTTTTAGCGGTGCGTACAATTAAGGGATTATGGTA

AATCCACTTACTGTCTGCCCTCGTAGCCATCGAGATAAACCGCAGTACTCCGG

CCACGATGCGTCCGGCGTAGAGGATCGAGATCT

>pFN22K-Crimson/P2A-ICP8

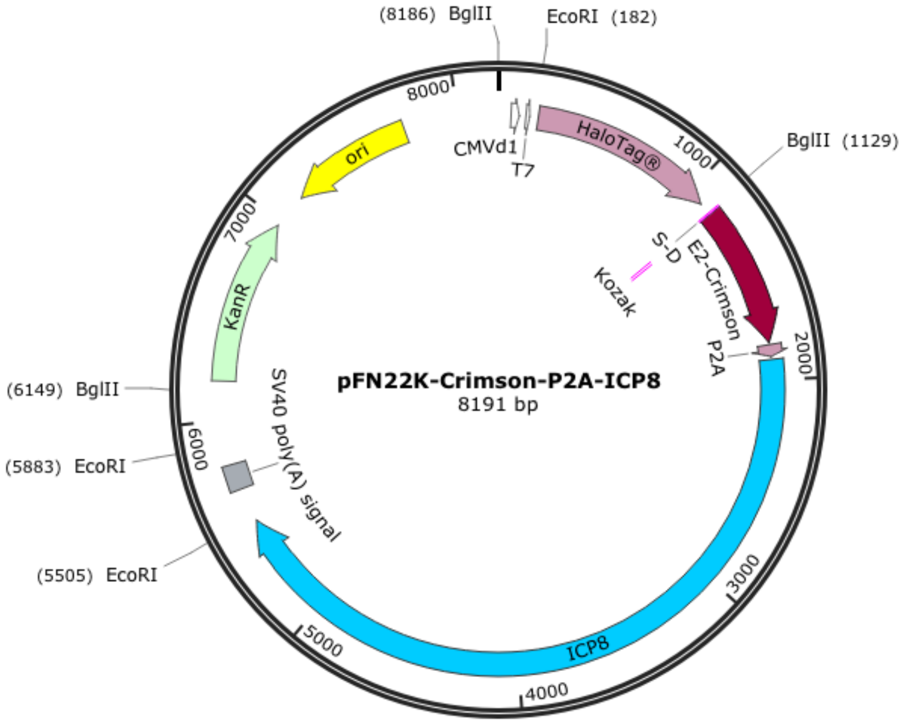

>pFN22K-Crimson/P2A-ICP8

CCAAAATCAACGGGACTTTCCAAAATGTCGTAATAACCCCGCCCCGTTGACG

CAAATGGGCGGTAGGCGTGTACGGTGGGAGGTCTATATAAGCAGAGCTGGTT

TAGTGAACCGTCAGATCACTAGAAGCTTAATACGACTCACTATAGGGTACGA

TTTAGGTGACACTATAGAATAAGGAGAATTCATCATGGCAGAAATCGGTACT

GGCTTTCCATTCGACCCCCATTATGTGGAAGTCCTGGGCGAGCGCATGCACTA

CGTCGATGTTGGTCCGCGCGATGGCACCCCTGTGCTGTTCCTGCACGGTAACC

CGACCTCCTCCTACGTGTGGCGCAACATCATCCCGCATGTTGCACCGACCCAT

CGCTGCATTGCTCCAGACCTGATCGGTATGGGCAAATCCGACAAACCAGACC

TGGGTTATTTCTTCGACGACCACGTCCGCTTCATGGATGCCTTCATCGAAGCC

CTGGGTCTGGAAGAGGTCGTCCTGGTCATTCACGACTGGGGCTCCGCTCTGG

GTTTCCACTGGGCCAAGCGCAATCCAGAGCGCGTCAAAGGTATTGCATTTAT

GGAGTTCATCCGCCCTATCCCGACCTGGGACGAATGGCCAGAATTTGCCCGC

GAGACCTTCCAGGCCTTCCGCACCACCGACGTCGGCCGCAAGCTGATCATCG

ATCAGAACGTTTTTATCGAGGGTACGCTGCCGATGGGTGTCGTCCGCCCGCTG

ACTGAAGTCGAGATGGACCATTACCGCGAGCCGTTCCTGAATCCTGTTGACC

GCGAGCCACTGTGGCGCTTCCCAAACGAGCTGCCAATCGCCGGTGAGCCAGC

GAACATCGTCGCGCTGGTCGAAGAATACATGGACTGGCTGCACCAGTCCCCT

GTCCCGAAGCTGCTGTTCTGGGGCACCCCAGGCGTTCTGATCCCACCGGCCG

AAGCCGCTCGCCTGGCCAAAAGCCTGCCTAACTGCAAGGCTGTGGACATCGG

CCCGGGTCTGAATCTGCTGCAAGAAGACAACCCGGACCTGATCGGCAGCGAG

ATCGCGCGCTGGCTGTCGACGCTCGAGATTTCCGGCGAGCCAACCACTGAGG

ATCTGTACTTTCAGAGCGATAACGCGATCGCAGATCTAGGAGGTCCGCCACC

ATGGATAGCACTGAGAACGTCATCAAGCCCTTCATGCGCTTCAAGGTGCACA

TGGAGGGCTCCGTGAACGGCCACGAGTTCGAGATCGAGGGCGTGGGCGAGG

GCAAGCCCTACGAGGGCACCCAGACCGCCAAGCTGCAAGTGACCAAGGGCG

GCCCCCTGCCCTTCGCCTGGGACATCCTGTCCCCCCAGTTCTTCTACGGCTCC

AAGGCGTACATCAAGCACCCCGCCGACATCCCCGACTACCTCAAGCAGTCCT

TCCCCGAGGGCTTCAAGTGGGAGCGCGTGATGAACTTCGAGGACGGCGGCGT

GGTGACCGTGACCCAGGACTCCTCCCTGCAGGACGGCACCCTCATCTACCAC

GTGAAGTTCATCGGCGTGAACTTCCCCTCCGACGGCCCCGTAATGCAGAAGA

AGACTCTGGGCTGGGAGCCCTCCACTGAGCGCAACTACCCCCGCGACGGCGT

GCTGAAGGGCGAGAACCACATGGCGCTGAAGCTGAAGGGCGGCGGCCACTA

CCTGTGTGAGTTCAAGTCCATCTACATGGCCAAGAAGCCCGTGAAGCTGCCC

GGCTACCACTACGTGGACTACAAGCTCGACATCACCTCCCACAACGAGGACT

ACACCGTGGTGGAGCAGTACGAGCGCGCCGAGGCCCGCCACCACCTGTTCCA

GGGATCCGGAGCCACGAACTTCTCTCTGTTAAAGCAAGCAGGAGACGTGGAA

GAAAACCCCGGTCCTCACATGTCTGCGATCGCCATGGAGACAAAGCCCAAGA

CGGCAACCACCATCAAGGTCCCCCCCGGGCCCCTGGGATACGTGTACGCTCG

CGCGTGTCCGTCCGAAGGCATCGAGCTTCTGGCGTTACTGTCGGCGCGCAGC

GGCGATGCCGACGTCGCCGTGGCGCCCCTGGTCGTGGGCCTGACCGTGGAGA

GCGGCTTTGAGGCCAACGTAGCCGTGGTCGTGGGTTCTCGCACGACGGGGCT

CGGGGGTACCGCGGTGTCCCTGAAACTGACGCCATCGCACTACAGCTCGTCC

GTGTACGTCTTTCACGGCGGCCGGCACCTGGACCCCAGCACCCAGGCCCCAA

ACCTGACGCGACTCTGCGAGCGGGCACGCCGCCATTTTGGCTTTTCGGACTAC

ACCCCCCGGCCCGGCGACCTCAAACACGAGACGACGGGGGAGGCGCTGTGT

GAGCGCCTCGGCCTGGACCCGGACCGCGCCCTCCTGTATCTGGTCGTTACCG

AGGGCTTCAAGGAGGCCGTGTGCATCAACAACACCTTTCTGCACCTGGGAGG

CTCGGACAAGGTAACCATAGGCGGGGCGGAGGTGCACCGCATACCCGTGTAT

CCGTTGCAGCTGTTCATGCCGGATTTTAGCCGGGTCATCGCCGAGCCGTTCAA

CGCCAACCACCGATCGATCGGGGAGAATTTTACCTACCCGCTTCCGTTTTTA

ACCGCCCCCTCAACCGCCTCCTGTTCGAGGCGGTCGTGGGACCCGCCGCCGT

GGCACTGCGATGCCGAAACGTGGACGCCGTGGCCCGCGCGGCCGCCCACCTG

GCGTTTGACGAAAACCACGAGGGCGCCGCCCTCCCCGCCGACATTACGTTCA

CGGCCTTCGAAGCCAGCCAGGGTAAGACCCCGCGGGGTGGGCGCGACGGCG

GCGGCAAGGGCCCGGCGGGCGGGTTCGAACAGCGCCTGGCCTCCGTCATGGC

CGGAGACGCCGCCCTGGCCCTCGAGTCTATCGTGTCGATGGCCGTCTTCGAC

GAGCCGCCCACCGACATCTCCGCGTGGCCGCTGTGCGAGGGCCAGGACACGG

CCGCGGCCCGCGCCAACGCCGTCGGGGCGTACCTGGCGCGCGCCGCGGGACT

CGTGGGGGCCATGGTATTTAGCACCAACTCGGCCCTCCATCTCACCGAGGTG

GACGACGCCGGTCCGGCGGACCCAAAGGACCACAGCAAACCCTCCTTTTACC

GCTTCTTCCTCGTGCCCGGGACCCACGTGGCGGCCAACCCACAGGTGGACCG

CGAGGGACACGTGGTGCCCGGGTTCGAGGGTCGGCCCACCGCGCCCCTCGTC

GGCGGAACCCAGGAATTTGCCGGCGAGCACCTGGCCATGCTGTGTGGGTTTT

CCCCGGCGCTGCTGGCCAAGATGCTGTTTTACCTGGAGCGCTGCGACGGCGG

CGTGATCGTCGGGCGCCAGGAGATGGACGTGTTTCGATACGTCGCGGACTCC

AACCAGACCGACGTGCCCTGCAACCTGTGCACCTTCGACACGCGCCACGCCT

GCGTACACACGACGCTCATGCGCCTCCGGGCGCGCCATCCCAAGTTCGCCAG

CGCCGCCCGCGGAGCCATCGGCGTCTTCGGGACCATGAACAGCATGTACAGC

GACTGCGACGTGCTGGGAAACTACGCCGCCTTCTCGGCCCTGAAGCGCGCGG

ACGGATCCGAGACCGCCCGGACCATCATGCAGGAGACGTACCGCGCGGCGA

CCGAGCGCGTCATGGCCGAACTCGAGACCCTGCAGTACGTGGACCAGGCGGT

CCCCACGGCCATGGGGCGGCTGGAGACCATCATCACCAACCGCGAGGCCCTG

CATACGGTGGTGAACAACGTCAGGCAGGTCGTGGACCGCGAGGTGGAGCAG

CTGATGCGCAACCTGGTGGAGGGGAGGAACTTCAAGTTTCGCGACGGTCTGG

GCGAGGCCAACCACGCCATGTCCCTGACGCTGGACCCGTACGCGTGCGGGCC

ATGCCCCCTGCTTCAGCTTCTCGGGCGGCGATCCAACCTCGCCGTGTATCAGG

ACCTGGCCCTGAGCCAGTGCCACGGGGTGTTCGCCGGGCAGTCGGTCGAGGG

GCGCAACTTTCGCAATCAATTCCAACCGGTGCTGCGGCGGCGCGTGATGGAC

ATGTTTAACAACGGGTTTCTGTCGGCCAAAACGCTGACGGTCGCGCTCTCGG

AGGGGGCGGCTATCTGCGCCCCCAGCCTAACGGCCGGCCAGACGGCCCCCGC

CGAGAGCAGCTTCGAGGGCGACGTTGCCCGCGTGACCCTGGGGTTTCCCAAG

GAGCTGCGCGTCAAGAGCCGCGTGTTGTTCGCGGGCGCGAGCGCCAACGCGT

CCGAGGCCGCCAAGGCGCGGGTCGCCAGCCTCCAGAGCGCCTACCAGAAGC

CCGACAAGCGCGTGGACATCCTCCTCGGACCGCTGGGCTTTCTGCTGAAGCA

GTTCCACGCGGCCATCTTCCCCAACGGCAAGCCCCCGGGGTCCAACCAGCCG

AACCCGCAGTGGTTCTGGACGGCCCTCCAACGCAACCAGCTTCCCGCCCGGC

TCCTGTCGCGCGAGGACATCGAGACCATCGCGTTCATTAAAAAGTTTTCCCTG

GACTACGGCGCGATAAACTTTATTAACCTGGCCCCCAACAACGTGAGCGAGC

TGGCGATGTACTACATGGCAAACCAGATTCTGCGGTACTGCGATCACTCGAC

ATACTTCATCAACACCCTCACGGCCATCATCGCGGGGTCCCGCCGTCCCCCA

GCGTGCAGGCGGCGGCCGCGTGGTCCGCGCAGGGCGGGGCGGGCCTGGAGG

CCGGGGCCCGCGCGCTGATGGACGCCGTGGACGCGCATCCGGGCGCGTGGAC

GTCCATGTTCGCCAGCTGCAACCTGCTGCGGCCCGTCATGGCGGCGCGCCCC

ATGGTCGTGTTGGGGTTGAGCATCAGCAAATACTACGGCATGGCCGGCAACG

ACCGTGTGTTTCAGGCCGGGAACTGGGCCAGCCTGATGGGCGGCAAAAACGC

GTGCCCGCTCCTTATTTTTGACCGCACCCGCAAGTTCGTCCTGGCCTGTCCCC

GGGCCGGGTTTGTGTGCGCGGCCTCGAACCTCGGCGGCGGAGCGCACGAAAG

CTCGCTGTGCGAGCAGCTCCGGGGCATTATCTCCGAGGGCGGGGCGGCCGTC

GCCAGTAGCGTGTTCGTGGCGACCGTGAAAAGCCTGGGGCCCCGCACCCAGC

AGCTGCAGATCGAGGACTGGCTGGCGCTCCTGGAGGACGAGTACCTAAGCGA

GGAGATGATGGAGCTGACCGCGCGTGCCCTGGAGCGCGGCAACGGCGAGTG

GTCGACGGACGCGGCCCTGGAGGTGGCGCACGAGGCCGAGGCCCTAGTCAG

CCAACTCGGCAACGCCGGGGAGGTGTTTAACTTTGGGGATTTTGGCTGCGAG

GACGACAACGCGACGCCGTTCGGCGGCCCGGGGGCCCCGGGACCGGCATTTG

CCGGCCGCAAACGGGCGTTCCACGGGGATGACCCGTTTGGGGAGGGGCCCCC

CGACAAAAAGGGAGACCTGACGTTGGATATGCTGGTTTAAACGAATTCGGGC

TCGGTACCCGGGGATCCTCTAGAGTCGACCTGCAGGCATGCAAGCTGATCCG

GCTGCTAACAAAGCCCGAAAGGAAGCTGAGTTGGCTGCTGCCACCGCTGAGC

AATAACTAGCATAACCCCTTGGGGCGGCCGCTTCGAGCAGACATGATAAGAT

ACATTGATGAGTTTGGACAAACCACAACTAGAATGCAGTGAAAAAAATGCTT

TATTTGTGAAATTTGTGATGCTATTGCTTTATTTGTAACCATTATAAGCTGCAA

TAAACAAGTTAACAACAACAATTGCATTCATTTTATGTTTCAGGTTCAGGGGG

AGATGTGGGAGGTTTTTTAAAGCAAGTAAAACCTCTACAAATGTGGTAAAAT

CGAATTCTAATGGATCCTCTTTGCGCTTGCGTTTTCCCTTGTCCAGATAGCCCA

GTAGCTGACATTCATCCGGGGTCAGCACCGTTTCTGCGGACTGGCTTTCTACG

TGTTCCGCTTCCTTTAGCAGCCCTTGCGCCCTGAGTGCTTGCGGCAGCGTGAG

CTTCAAAAGAATTGCCAGCTGGGGCGCCCTCTGGTAAGGTTGGGAAGCCCTG

CAAAGTAAACTGGATGGCTTTCTTGCCGCCAAGGATCTGATGGCGCAGGGGA

TCAAGATCTGATCAAGAGACAGGATGACGGTCGTTTCGCATGCTTGAACAAG

ATGGATTGCACGCAGGTTCTCCGGCCGCTTGGGTGGAGAGGCTATTCGGCTA

TGACTGGGCACAACAGACAATCGGCTGCTCTGATGCCGCCGTGTTCCGGCTG

TCAGCGCAGGGGCGCCCGGTTCTTTTTGTCAAGACCGACCTGTCCGGTGCCCT

GAATGAACTGCAGGACGAGGCAGCGCGGCTATCGTGGCTGGCCACGACGGG

CGTTCCTTGCGCAGCTGTGCTCGACGTTGTCACTGAAGCGGGAAGGGACTGG

CTGCTATTGGGCGAAGTGCCGGGGCAGGATCTCCTGTCATCTCACCTTGCTCC

TGCCGAGAAAGTATCCATCATGGCTGATGCAATGCGGCGGCTGCATACGCTT

GATCCGGCTACCTGCCCATTCGACCACCAAGCGAAACATCGCATCGAGCGAG

CACGCACTCGGATGGAAGCCGGTCTTGTCGATCAGGATGATCTGGACGAAGA

GCATCAGGGGCTCGCGCCAGCCGAACTGTTCGCCAGGCTCAAGGCGCGTATG

CCGGATGGTGAGGATCTCGTCGTGACTCATGGCGATGCCTGCTTGCCGAATAT

CATGGTGGAAAATGGCCGCTTTTCTGGATTCATCGACTGTGGCCGGCTGGGTG

TGGCGGACCGCTATCAGGACATAGCGTTGGCTACCCGTGATATTGCTGAAGA

GCTTGGCGGCGAATGGGCTGACCGCTTCCTCGTGCTTTACGGTATCGCCGCTC

CCGATTCGCAGCGCATCGCCTTCTATCGCCTTCTTGACGAGTTCTTCTGAGCG

GGACTCTGGGGTTCGAAATGACCGACCAAGCGACGCCCAACCGGTATCAGCT

CACTCAAAGGCGGTAATACGGTTATCCACAGAATCAGGGGATAACGCAGGA

AAGAACATGTGAGCAAAAGGCCAGCAAAAGGCCAGGAACCGTAAAAAGGCC

GCGTTGCTGGCGTTTTTCCATAGGCTCCGCCCCCCTGACGAGCATCACAAAAA

TCGACGCTCAAGTCAGAGGTGGCGAAACCCGACAGGACTATAAAGATACCA

GGCGTTTCCCCCTGGAAGCTCCCTCGTGCGCTCTCCTGTTCCGACCCTGCCGC

TTACCGGATACCTGTCCGCCTTTCTCCCTTCGGGAAGCGTGGCGCTTTCTCAT

AGCTCACGCTGTAGGTATCTCAGTTCGGTGTAGGTCGTTCGCTCCAAGCTGGG

CTGTGTGCACGAACCCCCCGTTCAGCCCGACCGCTGCGCCTTATCCGGTAACT

ATCGTCTTGAGTCCAACCCGGTAAGACACGACTTATCGCCACTGGCAGCAGC

CACTGGTAACAGGATTAGCAGAGCGAGGTATGTAGGCGGTGCTACAGAGTTC

TTGAAGTGGTGGCCTAACTACGGCTACACTAGAAGGACAGTATTTGGTATCT

GCGCTCTGCTGAAGCCAGTTACCTTCGGAAAAAGAGTTGGTAGCTCTTGATCC

GGCAAACAAACCACCGCTGGTAGCGGTGGTTTTTTTGTTTGCAAGCAGCAGA

TTACGCGCAGAAAAAAAGGATTTCAAGAAGATCCTTTGATCTTTTCTACGGG

GTCTGACGCTCAGTGGAACGAAAACTCACGTTAAGGGATTTTGGTCATGAGA

TTATCAAAAAGGATCTTCACCTAGATCCTTTTATAGTCCGGAAATACAGGAAC

GCACGCTGGATGGCCCTTCGCTGGGATGGTGAAACCATGAAAAATGGCAGCT

TCAGTGGATTAAGTGGGGGTAATGTGGCCTGTACCCTCTGGTTGCATAGGTAT

TCATACGGTTAAAATTTATCAGGCGCGATTGCGGCAGTTTTTCGGGTGGTTTG

TTGCCATTTTTACCTGTCTGCTGCCGTGATCGCGCTGAACGCGTTTTAGCGGT

GCGTACAATTAAGGGATTATGGTAAATCCACTTACTGTCTGCCCTCGTAGCCA

TCGAGATAAACCGCAGTACTCCGGCCACGATGCGTCCGGCGTAGAGGATCGA

GATCT

>pENTR/TREPitt::Crimson/P2A-ICP8

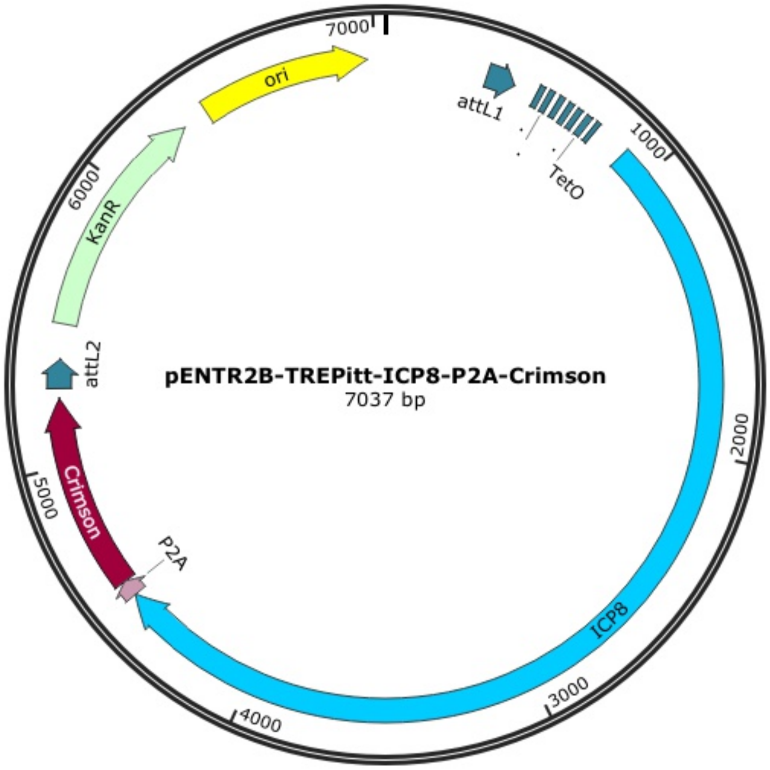

> pENTR/TREPitt::Crimson/P2A-ICP8

CTTTCCTGCGTTATCCCCTGATTCTGTGGATAACCGTATTACCGCTAGCATGG

ATCTCGGGGACGTCTAACTACTAAGCGAGAGTAGGGAACTGCCAGGCATCAA

ATAAAACGAAAGGCTCAGTCGGAAGACTGGGCCTTTCGTTTTATCTGTTGTTT

GTCGGTGAACGCTCTCCTGAGTAGGACAAATCCGCCGGGAGCGGATTTGAAC

GTTGTGAAGCAACGGCCCGGAGGGTGGCGGGCAGGACGCCCGCCATAAACT

GCCAGGCATCAAACTAAGCAGAAGGCCATCCTGACGGATGGCCTTTTTGCGT

TTCTACAAACTCTTCCTGTTAGTTAGTTACTTAAGCTCGGGCCCCAAATAATG

ATTTTATTTTGACTGATAGTGACCTGTTCGTTGCAACAAATTGATAAGCAATG

CTTTTTTATAATGCCAACTTTGTACAAAAAAGCAGGCTGGCGCCGGAACCAA

TTCAgatatcAAGTGCCACCTGACGTCTCCCTATCAGTGATAGAGAAGTCGACAC

GTCTCGAGCTCCCTATCAGTGATAGAGAAGGTACGTCTAGAACGTCTCCCTAT

CAGTGATAGAGAAGTCGACACGTCTCGAGCTCCCTATCAGTGATAGAGAAGG

TACGTCTAGAACGTCTCCCTATCAGTGATAGAGAAGTCGACACGTCTCGAGC

TCCCTATCAGTGATAGAGAAGGTACGTCTAGAACGTCTCCCTATCAGTGATA

GAGAAGTCGACACGTCTCGAGCTCCCTATCAGTGATAGAGAAGGTACCCCCT

ATATAAGCAGAGCTCGTTTAGTGAACCGTCAGATCGCCTGGAGACGCCATCC

ACGCTGTTTTGACCTCCATAGAAGACACCGGGACCGATCCAGCCTGGATCTA

GGAGGTCCGCCACCATGGATAGCACTGAGAACGTCATCAAGCCCTTCATGCG

CTTCAAGGTGCACATGGAGGGCTCCGTGAACGGCCACGAGTTCGAGATCGAG

GGCGTGGGCGAGGGCAAGCCCTACGAGGGCACCCAGACCGCCAAGCTGCAA

GTGACCAAGGGCGGCCCCCTGCCCTTCGCCTGGGACATCCTGTCCCCCCAGTT

CTTCTACGGCTCCAAGGCGTACATCAAGCACCCCGCCGACATCCCCGACTAC

CTCAAGCAGTCCTTCCCCGAGGGCTTCAAGTGGGAGCGCGTGATGAACTTCG

AGGACGGCGGCGTGGTGACCGTGACCCAGGACTCCTCCCTGCAGGACGGCAC

CCTCATCTACCACGTGAAGTTCATCGGCGTGAACTTCCCCTCCGACGGCCCCG

TAATGCAGAAGAAGACTCTGGGCTGGGAGCCCTCCACTGAGCGCAACTACCC

CCGCGACGGCGTGCTGAAGGGCGAGAACCACATGGCGCTGAAGCTGAAGGG

CGGCGGCCACTACCTGTGTGAGTTCAAGTCCATCTACATGGCCAAGAAGCCC

GTGAAGCTGCCCGGCTACCACTACGTGGACTACAAGCTCGACATCACCTCCC

ACAACGAGGACTACACCGTGGTGGAGCAGTACGAGCGCGCCGAGGCCCGCC

ACCACCTGTTCCAGGGATCCGGAGCCACGAACTTCTCTCTGTTAAAGCAAGC

AGGAGACGTGGAAGAAAACCCCGGTCCTCACATGTCTGCGATCGCCATGGAG

ACAAAGCCCAAGACGGCAACCACCATCAAGGTCCCCCCCGGGCCCCTGGGAT

ACGTGTACGCTCGCGCGTGTCCGTCCGAAGGCATCGAGCTTCTGGCGTTACTG
TCGGCGCGCAGCGGCGATGCCGACGTCGCCGTGGCGCCCCTGGTCGTGGGCC
TGACCGTGGAGAGCGGCTTTGAGGCCAACGTAGCCGTGGTCGTGGGTTCTCG
CACGACGGGGCTCGGGGGTACCGCGGTGTCCCTGAAACTGACGCCATCGCAC
TACAGCTCGTCCGTGTACGTCTTTCACGGCGGCCGGCACCTGGACCCCAGCA
CCCAGGCCCCAAACCTGACGCGACTCTGCGAGCGGGCACGCCGCCATTTTGG
CTTTTCGGACTACACCCCCCGGCCCGGCGACCTCAAACACGAGACGACGGGG
GAGGCGCTGTGTGAGCGCCTCGGCCTGGACCCGGACCGCGCCCTCCTGTATC
TGGTCGTTACCGAGGGCTTCAAGGAGGCCGTGTGCATCAACAACACCTTTCT
GCACCTGGGAGGCTCGGACAAGGTAACCATAGGCGGGGCGGAGGTGCACCG
CATACCCGTGTATCCGTTGCAGCTGTTCATGCCGGATTTTAGCCGGGTCATCG
CCGAGCCGTTCAACGCCAACCACCGATCGATCGGGGAGAATTTTACCTACCC
GCTTCCGTTTTTTAACCGCCCCCTCAACCGCCTCCTGTTCGAGGCGGTCGTGG
GACCCGCCGCCGTGGCACTGCGATGCCGAAACGTGGACGCCGTGGCCCGCGC
GGCCGCCCACCTGGCGTTTGACGAAAACCACGAGGGCGCCGCCCTCCCCGCC
GACATTACGTTCACGGCCTTCGAAGCCAGCCAGGGTAAGACCCCGCGGGGTG
GGCGCGACGGCGGCGGCAAGGGCCCGGCGGGCGGGTTCGAACAGCGCCTGG
CCTCCGTCATGGCCGGAGACGCCGCCCTGGCCCTCGAGTCTATCGTGTCGATG
GCCGTCTTCGACGAGCCGCCCACCGACATCTCCGCGTGGCCGCTGTGCGAGG
GCCAGGACACGGCCGCGGCCCGCGCCAACGCCGTCGGGGCGTACCTGGCGC
GCGCCGCGGGACTCGTGGGGGCCATGGTATTTAGCACCAACTCGGCCCTCCA
TCTCACCGAGGTGGACGACGCCGGTCCGGCGGACCCAAAGGACCACAGCAA
ACCCTCCTTTTACCGCTTCTTCCTCGTGCCCGGGACCCACGTGGCGGCCAACC

CACAGGTGGACCGCGAGGGACACGTGGTGCCCGGGTTCGAGGGTCGGCCCA

CCGCGCCCCTCGTCGGCGGAACCCAGGAATTTGCCGGCGAGCACCTGGCCAT

GCTGTGTGGGTTTTCCCCGGCGCTGCTGGCCAAGATGCTGTTTTACCTGGAGC

GCTGCGACGGCGGCGTGATCGTCGGGCGCCAGGAGATGGACGTGTTTCGATA

CGTCGCGGACTCCAACCAGACCGACGTGCCCTGCAACCTGTGCACCTTCGAC

ACGCGCCACGCCTGCGTACACACGACGCTCATGCGCCTCCGGGCGCGCCATC

CCAAGTTCGCCAGCGCCGCCCGCGGAGCCATCGGCGTCTTCGGGACCATGAA

CAGCATGTACAGCGACTGCGACGTGCTGGGAAACTACGCCGCCTTCTCGGCC

CTGAAGCGCGCGGACGGATCCGAGACCGCCCGGACCATCATGCAGGAGACG

TACCGCGCGGCGACCGAGCGCGTCATGGCCGAACTCGAGACCCTGCAGTACG

TGGACCAGGCGGTCCCCACGGCCATGGGGCGGCTGGAGACCATCATCACCAA

CCGCGAGGCCCTGCATACGGTGGTGAACAACGTCAGGCAGGTCGTGGACCGC

GAGGTGGAGCAGCTGATGCGCAACCTGGTGGAGGGGAGGAACTTCAAGTTTC

GCGACGGTCTGGGCGAGGCCAACCACGCCATGTCCCTGACGCTGGACCCGTA

CGCGTGCGGGCCATGCCCCCTGCTTCAGCTTCTCGGGCGGCGATCCAACCTCG

CCGTGTATCAGGACCTGGCCCTGAGCCAGTGCCACGGGGTGTTCGCCGGGCA

GTCGGTCGAGGGGCGCAACTTTCGCAATCAATTCCAACCGGTGCTGCGGCGG

CGCGTGATGGACATGTTTAACAACGGGTTTCTGTCGGCCAAAACGCTGACGG

TCGCGCTCTCGGAGGGGGCGGCTATCTGCGCCCCCAGCCTAACGGCCGGCCA

GACGGCCCCCGCCGAGAGCAGCTTCGAGGGCGACGTTGCCCGCGTGACCCTG

GGGTTTCCCAAGGAGCTGCGCGTCAAGAGCCGCGTGTTGTTCGCGGGCGCGA

GCGCCAACGCGTCCGAGGCCGCCAAGGCGCGGGTCGCCAGCCTCCAGAGCG

CCTACCAGAAGCCCGACAAGCGCGTGGACATCCTCCTCGGACCGCTGGGCTT

TCTGCTGAAGCAGTTCCACGCGGCCATCTTCCCCAACGGCAAGCCCCGGGG

TCCAACCAGCCGAACCCGCAGTGGTTCTGGACGGCCCTCCAACGCAACCAGC

TTCCCGCCCGGCTCCTGTCGCGCGAGGACATCGAGACCATCGCGTTCATTAA

AAAGTTTTCCCTGGACTACGGCGCGATAAACTTTATTAACCTGGCCCCCAACA

ACGTGAGCGAGCTGGCGATGTACTACATGGCAAACCAGATTCTGCGGTACTG

CGATCACTCGACATACTTCATCAACACCCTCACGGCCATCATCGCGGGGTCCC

GCCGTCCCCCCAGCGTGCAGGCGGCGGCCGCGTGGTCCGCGCAGGGCGGGGC

GGGCCTGGAGGCCGGGGCCCGCGCGCTGATGGACGCCGTGGACGCGCATCC

GGGCGCGTGGACGTCCATGTTCGCCAGCTGCAACCTGCTGCGGCCCGTCATG

GCGGCGCGCCCCATGGTCGTGTTGGGGTTGAGCATCAGCAAATACTACGGCA

TGGCCGGCAACGACCGTGTGTTTCAGGCCGGGAACTGGGCCAGCCTGATGGG

CGGCAAAAACGCGTGCCCGCTCCTTATTTTGACCGCACCCGCAAGTTCGTCC

TGGCCTGTCCCCGGGCCGGGTTTGTGTGCGCGGCCTCGAACCTCGGCGGCGG

AGCGCACGAAAGCTCGCTGTGCGAGCAGCTCCGGGGCATTATCTCCGAGGGC

GGGGCGGCCGTCGCCAGTAGCGTGTTCGTGGCGACCGTGAAAAGCCTGGGGC

CCCGCACCCAGCAGCTGCAGATCGAGGACTGGCTGGCGCTCCTGGAGGACGA

GTACCTAAGCGAGGAGATGATGGAGCTGACCGCGCGTGCCCTGGAGCGCGG

CAACGGCGAGTGGTCGACGGACGCGGCCCTGGAGGTGGCGCACGAGGCCGA

GGCCCTAGTCAGCCAACTCGGCAACGCCGGGGAGGTGTTTAACTTTGGGGAT

TTTGGCTGCGAGGACGACAACGCGACGCCGTTCGGCGGCCCGGGGGCCCCGG

GACCGGCATTTGCCGGCCGCAAACGGGCGTTCCACGGGGATGACCCGTTTGG

GGAGGGGCCCCCCGACAAAAAGGGAGACCTGACGTTGGATATGCTGGTTTAA

ACGAATTCGCGGCCGCACTCGAGATATCTAGACCCAGCTTTCTTGTACAAAGT

TGGCATTATAAGAAAGCATTGCTTATCAATTTGTTGCAACGAACAGGTCACTA
TCAGTCAAAATAAAATCATTATTTGCCATCCAGCTGCAGCTCTGGCCCGTGTC
TCAAAATCTCTGATGTTACATTGCACAAGATAAAAATATATCATCATGAACA
ATAAAACTGTCTGCTTACATAAACAGTAATACAAGGGGTGTTATGAGCCATA
TTCAACGGGAAACGTCGAGGCCGCGATTAAATTCCAACATGGATGCTGATTT
ATATGGGTATAAATGGGCTCGCGATAATGTCGGGCAATCAGGTGCGACAATC
TATCGCTTGTATGGGAAGCCCGATGCGCCAGAGTTGTTTCTGAAACATGGCA
AAGGTAGCGTTGCCAATGATGTTACAGATGAGATGGTCAGACTAAACTGGCT
GACGGAATTTATGCCTCTTCCGACCATCAAGCATTTTATCCGTACTCCTGATG
ATGCATGGTTACTCACCACTGCGATCCCCGGAAAAACAGCATTCCAGGTATT
AGAAGAATATCCTGATTCAGGTGAAAATATTGTTGATGCGCTGGCAGTGTTC
CTGCGCCGGTTGCATTCGATTCCTGTTTGTAATTGTCCTTTTAACAGCGATCGC
GTATTTCGTCTCGCTCAGGCGCAATCACGAATGAATAACGGTTTGGTTGATGC
GAGTGATTTTGATGACGAGCGTAATGGCTGGCCTGTTGAACAAGTCTGGAAA
GAAATGCATAAACTTTTGCCATTCTCACCGGATTCAGTCGTCACTCATGGTGA
TTTCTCACTTGATAACCTTATTTTTGACGAGGGGAAATTAATAGGTTGTATTG
ATGTTGGACGAGTCGGAATCGCAGACCGATACCAGGATCTTGCCATCCTATG
GAACTGCCTCGGTGAGTTTTCTCCTTCATTACAGAAACGGCTTTTTCAAAAAT
ATGGTATTGATAATCCTGATATGAATAAATTGCAGTTTCATTTGATGCTCGAT
GAGTTTTTCTAATCAGAATTGGTTAATTGGTTGTAACATTATTCAGATTGGGC
CCCGTTCCACTGAGCGTCAGACCCCGTAGAAAAGATCAAAGGATCTTCTTGA
GATCCTTTTTTTCTGCGCGTAATCTGCTGCTTGCAAACAAAAAAACCACCGCT
ACCAGCGGTGGTTTGTTTGCCGGATCAAGAGCTACCAACTCTTTTTCCGAAGG

TAACTGGCTTCAGCAGAGCGCAGATACCAAATACTGTTCTTCTAGTGTAGCCG

TAGTTAGGCCACCACTTCAAGAACTCTGTAGCACCGCCTACATACCTCGCTCT

GCTAATCCTGTTACCAGTGGCTGCTGCCAGTGGCGATAAGTCGTGTCTTACCG

GGTTGGACTCAAGACGATAGTTACCGGATAAGGCGCAGCGGTCGGGCTGAAC

GGGGGGTTCGTGCACACAGCCCAGCTTGGAGCGAACGACCTACACCGAACTG

AGATACCTACAGCGTGAGCTATGAGAAAGCGCCACGCTTCCCGAAGGGAGA

AAGGCGGACAGGTATCCGGTAAGCGGCAGGGTCGGAACAGGAGAGCGCACG

AGGGAGCTTCCAGGGGGAAACGCCTGGTATCTTTATAGTCCTGTCGGGTTTCG

CCACCTCTGACTTGAGCGTCGATTTTTGTGATGCTCGTCAGGGGGGCGGAGCC

TATGGAAAAACGCCAGCAACGCGGCCTTTTTACGGTTCCTGGCCTTTTGCTGG

CCTTTTGCTCACATGTT

>pSLIK/TREPitt::Crimson/P2A-ICP8

AKA 1.pSLIK-Zeo/TREPitt::Crimson/P2A-ICP8 or pSLIK1. Sequence confirmed from 2607-7842 bp.

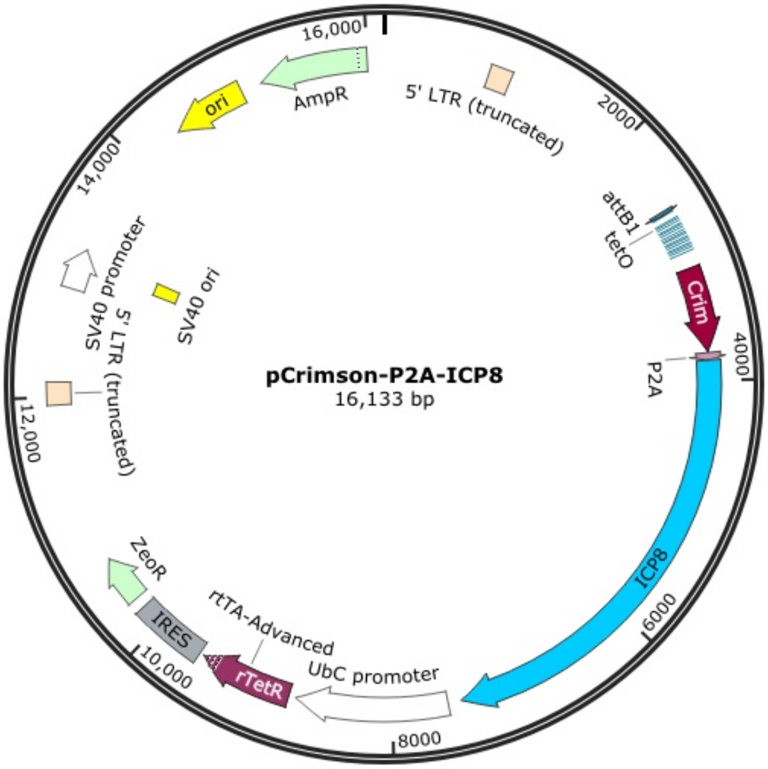

>1.pSLIK-Zeo/TREPitt::Crimson/P2A-ICP8

GTCGACGGATCGGGAGATCTCCCGATCCCCTATGGTGCACTCTCAGTACAAT

CTGCTCTGATGCCGCATAGTTAAGCCAGTATCTGCTCCCTGCTTGTGTGTTGG

AGGTCGCTGAGTAGTGCGCGAGCAAAATTTAAGCTACAACAAGGCAAGGCTT

GACCGACAATTGCATGAAGAATCTGCTTAGGGTTAGGCGTTTTGCGCTGCTTC

GCGATGTACGGGCCAGATATACGCGTTGACATTGATTATTGACTAGTTATTAA

TAGTAATCAATTACGGGGTCATTAGTTCATAGCCCATATATGGAGTTCCGCGT

TACATAACTTACGGTAAATGGCCCGCCTGGCTGACCGCCCAACGACCCCCGC

CCATTGACGTCAATAATGACGTATGTTCCCATAGTAACGCCAATAGGGACTTT

CCATTGACGTCAATGGGTGGAGTATTTACGGTAAACTGCCCACTTGGCAGTA

CATCAAGTGTATCATATGCCAAGTACGCCCCCTATTGACGTCAATGACGGTA

AATGGCCCGCCTGGCATTATGCCCAGTACATGACCTTATGGGACTTTCCTACT

TGGCAGTACATCTACGTATTAGTCATCGCTATTACCATGGTGATGCGGTTTTG

GCAGTACATCAATGGGCGTGGATAGCGGTTTGACTCACGGGGATTTCCAAGT

CTCCACCCCATTGACGTCAATGGGAGTTTGTTTTGGCACCAAAATCAACGGG

ACTTTCCAAAATGTCGTAACAACTCCGCCCCATTGACGCAAATGGGCGGTAG

GCGTGTACGGTGGGAGGTCTATATAAGCAGCGCGTTTTGCCTGTACTGGGTCT

CTCTGGTTAGACCAGATCTGAGCCTGGGAGCTCTCTGGCTAACTAGGGAACC

CACTGCTTAAGCCTCAATAAAGCTTGCCTTGAGTGCTTCAAGTAGTGTGTGCC

CGTCTGTTGTGTGACTCTGGTAACTAGAGATCCCTCAGACCCTTTTAGTCAGT

GTGGAAAATCTCTAGCAGTGGCGCCCGAACAGGGACTTGAAAGCGAAAGGG

AAACCAGAGGAGCTCTCTCGACGCAGGACTCGGCTTGCTGAAGCGCGCACGG

CAAGAGGCGAGGGGCGGCGACTGGTGAGTACGCCAAAAATTTTGACTAGCG

GAGGCTAGAAGGAGAGAGATGGGTGCGAGAGCGTCAGTATTAAGCGGGGGA

GAATTAGATCGCGATGGGAAAAAATTCGGTTAAGGCCAGGGGGAAAGAAAA

AATATAAATTAAAACATATAGTATGGGCAAGCAGGGAGCTAGAACGATTCGC

AGTTAATCCTGGCCTGTTAGAAACATCAGAAGGCTGTAGACAAATACTGGGA

CAGCTACAACCATCCCTTCAGACAGGATCAGAAGAACTTAGATCATTATATA

ATACAGTAGCAACCCTCTATTGTGTGCATCAAAGGATAGAGATAAAAGACAC

CAAGGAAGCTTTAGACAAGATAGAGGAAGAGCAAAACAAAAGTAAGACCAC

CGCACAGCAAGCGGCCGCTGATCTTCAGACCTGGAGGAGGAGATATGAGGG

ACAATTGGAGAAGTGAATTATATAAATATAAAGTAGTAAAAATTGAACCATT

AGGAGTAGCACCCACCAAGGCAAAGAGAAGAGTGGTGCAGAGAGAAAAAA
GAGCAGTGGGAATAGGAGCTTTGTTCCTTGGGTTCTTGGGAGCAGCAGGAAG
CACTATGGGCGCAGCGTCAATGACGCTGACGGTACAGGCCAGACAATTATTG
TCTGGTATAGTGCAGCAGCAGAACAATTTGCTGAGGGCTATTGAGGCGCAAC
AGCATCTGTTGCAACTCACAGTCTGGGGCATCAAGCAGCTCCAGGCAAGAAT
CCTGGCTGTGGAAAGATACCTAAAGGATCAACAGCTCCTGGGGATTTGGGGT
TGCTCTGGAAAACTCATTTGCACCACTGCTGTGCCTTGGAATGCTAGTTGGAG
TAATAAATCTCTGGAACAGATTTGGAATCACACGACCTGGATGGAGTGGGAC
AGAGAAATTAACAATTACACAAGCTTAATACACTCCTTAATTGAAGAATCGC
AAAACCAGCAAGAAAAGAATGAACAAGAATTATTGGAATTAGATAAATGGG
CAAGTTTGTGGAATTGGTTTAACATAACAAATTGGCTGTGGTATATAAAATTA
TTCATAATGATAGTAGGAGGCTTGGTAGGTTTAAGAATAGTTTTTGCTGTACT
TTCTATAGTGAATAGAGTTAGGCAGGGATATTCACCATTATCGTTTCAGACCC
ACCTCCCAACCCCGAGGGGACCCGACAGGCCCGAAGGAATAGAAGAAGAAG
GTGGAGAGAGAGACAGAGACAGATCCATTCGATTAGTGAACGGATCGGCAC
TGCGTGCGCCAATTCTGCAGACAAATGGCAGTATTCATCCACAATTTTAAAA
GAAAAGGGGGGATTGGGGGGTACAGTGCAGGGGAAAGAATAGTAGACATAA
TAGCAACAGACATACAAACTAAAGAATTACAAAAACAAATTACAAAAATTC
AAAATTTTCGGGTTTATTACAGGGACAGCAGAGATCCAGTTTGGTTAATTCTA
GACAGTTATCAACAAGTTTGTACAAAAAAGCAGGCTGGCGCCGGAACCAAG
GCGCCGGAACCAATTCAGATATCAAGTGCCACCTGACGTCTCCCTATCAGTG
ATAGAGAAGTCGACACGTCTCGAGCTCCCTATCAGTGATAGAGAAGGTACGT
CTAGAACGTCTCCCTATCAGTGATAGAGAAGTCGACACGTCTCGAGCTCCCT

ATCAGTGATAGAGAAGGTACGTCTAGAACGTCTCCCTATCAGTGATAGAGAA

GTCGACACGTCTCGAGCTCCCTATCAGTGATAGAGAAGGTACGTCTAGAACG

TCTCCCTATCAGTGATAGAGAAGTCGACACGTCTCGAGCTCCCTATCAGTGAT

AGAGAAGGTACCCCCTATATAAGCAGAGCTCGTTTAGTGAACCGTCAGATCG

CCTGGAGACGCCATCCACGCTGTTTTGACCTCCATAGAAGACACCGGGACCG

ATCCAGCCTGGATCTAGGAGGTCCGCCACCATGGATAGCACTGAGAACGTCA

TCAAGCCCTTCATGCGCTTCAAGGTGCACATGGAGGGCTCCGTGAACGGCCA

CGAGTTCGAGATCGAGGGCGTGGGCGAGGGCAAGCCCTACGAGGGCACCCA

GACCGCCAAGCTGCAAGTGACCAAGGGCGGCCCCCTGCCCTTCGCCTGGGAC

ATCCTGTCCCCCCAGTTCTTCTACGGCTCCAAGGCGTACATCAAGCACCCCGC

CGACATCCCCGACTACCTCAAGCAGTCCTTCCCCGAGGGCTTCAAGTGGGAG

CGCGTGATGAACTTCGAGGACGGCGGCGTGGTGACCGTGACCCAGGACTCCT

CCCTGCAGGACGGCACCCTCATCTACCACGTGAAGTTCATCGGCGTGAACTT

CCCCTCCGACGGCCCCGTAATGCAGAAGAAGACTCTGGGCTGGGAGCCCTCC

ACTGAGCGCAACTACCCCCGCGACGGCGTGCTGAAGGGCGAGAACCACATG

GCGCTGAAGCTGAAGGGCGGCGGCCACTACCTGTGTGAGTTCAAGTCCATCT

ACATGGCCAAGAAGCCCGTGAAGCTGCCCGGCTACCACTACGTGGACTACAA

GCTCGACATCACCTCCCACAACGAGGACTACACCGTGGTGGAGCAGTACGAG

CGCGCCGAGGCCCGCCACCACCTGTTCCAGGGATCCGGAGCCACGAACTTCT

CTCTGTTAAAGCAAGCAGGAGACGTGGAAGAAAACCCCGGTCCTCACATGTC

TGCGATCGCCATGGAGACAAAGCCCAAGACGGCAACCACCATCAAGGTCCCC

CCCGGGCCCCTGGGATACGTGTACGCTCGCGCGTGTCCGTCCGAAGGCATCG

AGCTTCTGGCGTTACTGTCGGCGCGCAGCGGCGATGCCGACGTCGCCGTGGC

GCCCCTGGTCGTGGGCCTGACCGTGGAGAGCGGCTTTGAGGCCAACGTAGCC

GTGGTCGTGGGTTCTCGCACGACGGGGCTCGGGGGTACCGCGGTGTCCCTGA

AACTGACGCCATCGCACTACAGCTCGTCCGTGTACGTCTTTCACGGCGGCCG

GCACCTGGACCCCAGCACCCAGGCCCCAAACCTGACGCGACTCTGCGAGCGG

GCACGCCGCCATTTTGGCTTTTCGGACTACACCCCCCGGCCCGGCGACCTCAA

ACACGAGACGACGGGGGAGGCGCTGTGTGAGCGCCTCGGCCTGGACCCGGA

CCGCGCCCTCCTGTATCTGGTCGTTACCGAGGGCTTCAAGGAGGCCGTGTGC

ATCAACAACACCTTTCTGCACCTGGGAGGCTCGGACAAGGTAACCATAGGCG

GGGCGGAGGTGCACCGCATACCCGTGTATCCGTTGCAGCTGTTCATGCCGGA

TTTTAGCCGGGTCATCGCCGAGCCGTTCAACGCCAACCACCGATCGATCGGG

GAGAATTTTACCTACCCGCTTCCGTTTTTAACCGCCCCCTCAACCGCCTCCT

GTTCGAGGCGGTCGTGGGACCCGCCGCCGTGGCACTGCGATGCCGAAACGTG

GACGCCGTGGCCCGCGCGGCCGCCCACCTGGCGTTTGACGAAAACCACGAGG

GCGCCGCCCTCCCCGCCGACATTACGTTCACGGCCTTCGAAGCCAGCCAGGG

TAAGACCCCGCGGGGTGGGCGCGACGGCGGCGGCAAGGGCCCGGCGGGCGG

GTTCGAACAGCGCCTGGCCTCCGTCATGGCCGGAGACGCCGCCCTGGCCCTC

GAGTCTATCGTGTCGATGGCCGTCTTCGACGAGCCGCCCACCGACATCTCCGC

GTGGCCGCTGTGCGAGGGCCAGGACACGGCCGCGGCCCGCGCCAACGCCGTC

GGGGCGTACCTGGCGCGCGCCGCGGGACTCGTGGGGGCCATGGTATTTAGCA

CCAACTCGGCCCTCCATCTCACCGAGGTGGACGACGCCGGTCCGGCGGACCC

AAAGGACCACAGCAAACCCTCCTTTTACCGCTTCTTCCTCGTGCCCGGGACCC

ACGTGGCGGCCAACCCACAGGTGGACCGCGAGGGACACGTGGTGCCCGGGT

TCGAGGGTCGGCCCACCGCGCCCCTCGTCGGCGGAACCCAGGAATTTGCCGG

CGAGCACCTGGCCATGCTGTGTGGGTTTTCCCCGGCGCTGCTGGCCAAGATG
CTGTTTTACCTGGAGCGCTGCGACGGCGGCGTGATCGTCGGGCGCCAGGAGA
TGGACGTGTTTCGATACGTCGCGGACTCCAACCAGACCGACGTGCCCTGCAA
CCTGTGCACCTTCGACACGCGCCACGCCTGCGTACACACGACGCTCATGCGC
CTCCGGGCGCGCCATCCCAAGTTCGCCAGCGCCGCCCGCGGAGCCATCGGCG
TCTTCGGGACCATGAACAGCATGTACAGCGACTGCGACGTGCTGGGAAACTA
CGCCGCCTTCTCGGCCCTGAAGCGCGCGGACGGATCCGAGACCGCCCGGACC
ATCATGCAGGAGACGTACCGCGCGGCGACCGAGCGCGTCATGGCCGAACTCG
AGACCCTGCAGTACGTGGACCAGGCGGTCCCCACGGCCATGGGGCGGCTGGA
GACCATCATCACCAACCGCGAGGCCCTGCATACGGTGGTGAACAACGTCAGG
CAGGTCGTGGACCGCGAGGTGGAGCAGCTGATGCGCAACCTGGTGGAGGGG
AGGAACTTCAAGTTTCGCGACGGTCTGGGCGAGGCCAACCACGCCATGTCCC
TGACGCTGGACCCGTACGCGTGCGGGCCATGCCCCCTGCTTCAGCTTCTCGGG
CGGCGATCCAACCTCGCCGTGTATCAGGACCTGGCCCTGAGCCAGTGCCACG
GGGTGTTCGCCGGGCAGTCGGTCGAGGGGCGCAACTTTCGCAATCAATTCCA
ACCGGTGCTGCGGCGGCGCGTGATGGACATGTTTAACAACGGGTTTCTGTCG
GCCAAAACGCTGACGGTCGCGCTCTCGGAGGGGGCGGCTATCTGCGCCCCCA
GCCTAACGGCCGGCCAGACGGCCCCCGCCGAGAGCAGCTTCGAGGGCGACG
TTGCCCGCGTGACCCTGGGGTTTCCCAAGGAGCTGCGCGTCAAGAGCCGCGT
GTTGTTCGCGGGCGCGAGCGCCAACGCGTCCGAGGCCGCCAAGGCGCGGGTC
GCCAGCCTCCAGAGCGCCTACCAGAAGCCCGACAAGCGCGTGGACATCCTCC
TCGGACCGCTGGGCTTTCTGCTGAAGCAGTTCCACGCGGCCATCTTCCCCAAC
GGCAAGCCCCCGGGGTCCAACCAGCCGAACCCGCAGTGGTTCTGGACGGCCC

TCCAACGCAACCAGCTTCCCGCCCGGCTCCTGTCGCGCGAGGACATCGAGAC
CATCGCGTTCATTAAAAAGTTTTCCCTGGACTACGGCGCGATAAACTTTATTA
ACCTGGCCCCCAACAACGTGAGCGAGCTGGCGATGTACTACATGGCAAACCA
GATTCTGCGGTACTGCGATCACTCGACATACTTCATCAACACCCTCACGGCCA
TCATCGCGGGGTCCCGCCGTCCCCCCAGCGTGCAGGCGGCGGCCGCGTGGTC
CGCGCAGGGCGGGGCGGGCCTGGAGGCCGGGGCCCGCGCGCTGATGGACGC
CGTGGACGCGCATCCGGGCGCGTGGACGTCCATGTTCGCCAGCTGCAACCTG
CTGCGGCCCGTCATGGCGGCGCGCCCCATGGTCGTGTTGGGGTTGAGCATCA
GCAAATACTACGGCATGGCCGGCAACGACCGTGTGTTTCAGGCCGGGAACTG
GGCCAGCCTGATGGGCGGCAAAAACGCGTGCCCGCTCCTTATTTTTGACCGC
ACCCGCAAGTTCGTCCTGGCCTGTCCCCGGGCCGGGTTTGTGTGCGCGGCCTC
GAACCTCGGCGGCGGAGCGCACGAAAGCTCGCTGTGCGAGCAGCTCCGGGG
CATTATCTCCGAGGGCGGGGCGGCCGTCGCCAGTAGCGTGTTCGTGGCGACC
GTGAAAAGCCTGGGGCCCCGCACCCAGCAGCTGCAGATCGAGGACTGGCTG
GCGCTCCTGGAGGACGAGTACCTAAGCGAGGAGATGATGGAGCTGACCGCG
CGTGCCCTGGAGCGCGGCAACGGCGAGTGGTCGACGGACGCGGCCCTGGAG
GTGGCGCACGAGGCCGAGGCCCTAGTCAGCCAACTCGGCAACGCCGGGGAG
GTGTTTAACTTTGGGGATTTTGGCTGCGAGGACGACAACGCGACGCCGTTCG
GCGGCCCGGGGGCCCCGGGACCGGCATTTGCCGGCCGCAAACGGGCGTTCCA
CGGGGATGACCCGTTTGGGGAGGGGCCCCCCGACAAAAAGGGAGACCTGAC
GTTGGATATGCTGGTTTAAACGAATTCGCGGCCGCACTCGAGATATCTAGAT
AGCTTTCTTGTACAAAGTGGTTGATAACATCTCGAGCCATAAAGATGGTTAAT
TAACCCACCCAAGATCTGGCCTCCGCGCCGGGTTTTGGCGCCTCCCGCGGGC

GCCCCCCTCCTCACGGCGAGCGCTGCCACGTCAGACGAAGGGCGCAGCGAGC

GTCCTGATCCTTCCGCCCGGACGCTCAGGACAGCGGCCCGCTGCTCATAAGA

CTCGGCCTTAGAACCCCAGTATCAGCAGAAGGACATTTTAGGACGGGACTTG

GGTGACTCTAGGGCACTGGTTTTCTTTCCAGAGAGCGGAACAGGCGAGGAAA

AGTAGTCCCTTCTCGGCGATTCTGCGGAGGGATCTCCGTGGGGCGGTGAACG

CCGATGATTATATAAGGACGCGCCGGGTGTGGCACAGCTAGTTCCGTCGCAG

CCGGGATTTGGGTCGCGGTTCTTGTTTGTGGATCGCTGTGATCGTCACTTGGT

GAGTAGCGGGCTGCTGGGCTGGCCGGGGCTTTCGTGGCCGCCGGGCCGCTCG

GTGGGACGGAAGCGTGTGGAGAGACCGCCAAGGGCTGTAGTCTGGGTCCGC

GAGCAAGGTTGCCCTGAACTGGGGGTTGGGGGGAGCGCAGCAAAATGGCGG

CTGTTCCCGAGTCTTGAATGGAAGACGCTTGTGAGGCGGGCTGTGAGGTCGT

TGAAACAAGGTGGGGGGCATGGTGGGCGGCAAGAACCCAAGGTCTTGAGGC

CTTCGCTAATGCGGGAAAGCTCTTATTCGGGTGAGATGGGCTGGGGCACCAT

CTGGGGACCCTGACGTGAAGTTTGTCACTGACTGGAGAACTCGGTTTGTCGTC

TGTTGCGGGGGCGGCAGTTATGGCGGTGCCGTTGGGCAGTGCACCCGTACCT

TTGGGAGCGCGCGCCCTCGTCGTGTCGTGACGTCACCCGTTCTGTTGGCTTAT

AATGCAGGGTGGGGCCACCTGCCGGTAGGTGTGCGGTAGGCTTTTCTCCGTC

GCAGGACGCAGGGTTCGGGCCTAGGGTAGGCTCTCCTGAATCGACAGGCGCC

GGACCTCTGGTGAGGGGAGGGATAAGTGAGGCGTCAGTTTCTTTGGTCGGTT

TTATGTACCTATCTTCTTAAGTAGCTGAAGCTCCGGTTTTGAACTATGCGCTC

GGGGTTGGCGAGTGTGTTTTGTGAAGTTTTTTAGGCACCTTTTGAAATGTAAT

CATTTGGGTCAATATGTAATTTTCAGTGTTAGACTAGTAAATTGTCCGCTAAA

TTCTGGCCGTTTTTGGCTTTTTTGTTAGACGAAGCTTGGTACCGAGCTCGGAT

CTCCACCCCGTACCGGTCCTGCAGTCGAATTCACCATGTCTAGACTGGACAA
GAGCAAAGTCATAAACGGAGCTCTGGAATTACTCAATGGTGTCGGTATCGAA
GGCCTGACGACAAGGAAACTCGCTCAAAAGCTGGGAGTTGAGCAGCCTACCC
TGTACTGGCACGTGAAGAACAAGCGGGCCCTGCTCGATGCCCTGCCAATCGA
GATGCTGGACAGGCATCATACCCACTTCTGCCCCCTGGAAGGCGAGTCATGG
CAAGACTTTCTGCGGAACAACGCCAAGTCATACCGCTGTGCTCTCCTCTCACA
TCGCGACGGGGCTAAAGTGCATCTCGGCACCCGCCCAACAGAGAAACAGTAC
GAAACCCTGGAAAATCAGCTCGCGTTCCTGTGTCAGCAAGGCTTCTCCCTGG
AGAACGCACTGTACGCTCTGTCCGCCGTGGGCCACTTTACACTGGGCTGCGT
ATTGGAGGAACAGGAGCATCAAGTAGCAAAAGAGGAAAGAGAGACACCTAC
CACCGATTCTATGCCCCCACTTCTGAGACAAGCAATTGAGCTGTTCGACCGGC
AGGGAGCCGAACCTGCCTTCCTTTTCGGCCTGGAACTAATCATATGTGGCCTG
GAGAAACAGCTAAAGTGCGAAAGCGGCGGGCCGACCGACGCCCTTGACGAT
TTTGACTTAGACATGCTCCCAGCCGATGCCCTTGACGACTTTGACCTTGATAT
GCTGCCTGCTGACGCTCTTGACGATTTTGACCTTGACATGCTCCCCGGGTAAC
TAAGTAAGGATCCGCGGCCGCACTAGAGGAATTCCGCCCCTCTCCCTCCCCC
CCCCCTAACGTTACTGGCCGAAGCCGCTTGGAATAAGGCCGGTGTGTGTTTGT
CTATATGTTATTTTCCACCATATTGCCGTCTTTTGGCAATGTGAGGGCCCGGA
AACCTGGCCCTGTCTTCTTGACGAGCATTCCTAGGGGTCTTTCCCCTCTCGCC
AAAGGAATGCAAGGTCTGTTGAATGTCGTGAAGGAAGCAGTTCCTCTGGAAG
CTTCTTGAAGACAAACAACGTCTGTAGCGACCCTTTGCAGGCAGCGGAACCC
CCCACCTGGCGACAGGTGCCTCTGCGGCCAAAAGCCACGTGTATAAGATACA
CCTGCAAAGGCGGCACAACCCCAGTGCCACGTTGTGAGTTGGATAGTTGTGG

AAAGAGTCAAATGGCTCTCCTCAAGCGTAGTCAACAAGGGGCTGAAGGATGC
CCAGAAGGTACCCCATTGTATGGGAATCTGATCTGGGGCCTCGGTGCACATG
CTTTACATGTGTTTAGTCGAGGTTAAAAAAACGTCTAGGCCCCCCGAACCAC
GGGGACGTGGTTTTCCTTTGAAAAACACGATGATAAGCTTACCGGTACGCGT
GATGTGTTGACAATTAATCATCGGCATAGTATATCGGCATAGTATAATACGA
CAAGGTGAGGAACTAAACCATGGCCAAGTTGACCAGTGCCGTTCCGGTGCTC
ACCGCGCGCGACGTCGCCGGAGCGGTCGAGTTCTGGACCGACCGGCTCGGGT
TCTCCCGGGACTTCGTGGAGGACGACTTCGCCGGTGTGGTCCGGGACGACGT
GACCCTGTTCATCAGCGCGGTCCAGGACCAGGTGGTGCCGGACAACACCCTG
GCCTGGGTGTGGGTGCGCGGCCTGGACGAGCTGTACGCCGAGTGGTCGGAGG
TCGTGTCCACGAACTTCCGGGACGCCTCCGGGCCGGCCATGACCGAGATCGG
CGAGCAGCCGTGGGGGCGGGAGTTCGCCCTGCGCGACCCGGCCGGCAACTGC
GTGCACTTCGTGGCCGAGGAGCAGGACTGACACATCTGTACAAGTAAAGCGG
CCGCGACTCTAGATCATAATCAGCCATACCACATTTGTAGAGGTTTTACTTGC
TTTAAAAAACCTCCCACACCTCCCCCTGAACCTGAAACATAAAATGAATGCA
ATTGTTGTTGTTTAGTCCCTCCCAATTCGATATCAAGCTTATCGATAATCAAC
CTCTGGATTACAAAATTTGTGAAAGATTGACTGGTATTCTTAACTATGTTGCT
CCTTTTACGCTATGTGGATACGCTGCTTTAATGCCTTTGTATCATGCTATTGCT
TCCCGTATGGCTTTCATTTTCTCCTCCTTGTATAAATCCTGGTTGCTGTCTCTTT
ATGAGGAGTTGTGGCCCGTTGTCAGGCAACGTGGCGTGGTGTGCACTGTGTTT
GCTGACGCAACCCCCACTGGTTGGGGCATTGCCACCACCTGTCAGCTCCTTTC
CGGGACTTTCGCTTTCCCCCTCCCTATTGCCACGGCGGAACTCATCGCCGCCT
GCCTTGCCCGCTGCTGGACAGGGGCTCGGCTGTTGGGCACTGACAATTCCGT

GGTGTTGTCGGGGAAATCATCGTCCTTTCCTTGGCTGCTCGCCTGTGTTGCCA
CCTGGATTCTGCGCGGGACGTCCTTCTGCTACGTCCCTTCGGCCCTCAATCCA
GCGGACCTTCCTTCCCGCGGCCTGCTGCCGGCTCTGCGGCCTCTTCCGCGTCT
TCGCCTTCGCCCTCAGACGAGTCGGATCTCCCTTTGGGCCGCCTCCCCGCATC
GATACCGTCGACCTCGATCGAGACCTAGAAAAACATGGAGCAATCACAAGTA
GCAATACAGCAGCTACCAATGCTGATTGTGCCTGGCTAGAAGCACAAGAGGA
GGAGGAGGTGGGTTTTCCAGTCACACCTCAGGTACCTTTAAGACCAATGACT
TACAAGGCAGCTGTAGATCTTAGCCACTTTTTAAAAGAAAAGGGGGGACTGG
AAGGGCTAATTCACTCCCAACGAAGACAAGATATCCTTGATCTGTGGATCTA
CCACACACAAGGCTACTTCCCTGATTGGCAGAACTACACACCAGGGCCAGGG
ATCAGATATCCACTGACCTTTGGATGGTGCTACAAGCTAGTACCAGTTGAGC
AAGAGAAGGTAGAAGAAGCCAATGAAGGAGAGAACACCCGCTTGTTACACC
CTGTGAGCCTGCATGGGATGGATGACCCGGAGAGAGAAGTATTAGAGTGGA
GGTTTGACAGCCGCCTAGCATTTCATCACATGGCCCGAGAGCTGCATCCGGA
CTGTACTGGGTCTCTCTGGTTAGACCAGATCTGAGCCTGGGAGCTCTCTGGCT
AACTAGGGAACCCACTGCTTAAGCCTCAATAAAGCTTGCCTTGAGTGCTTCA
AGTAGTGTGTGCCCGTCTGTTGTGTGACTCTGGTAACTAGAGATCCCTCAGAC
CCTTTTAGTCAGTGTGGAAAATCTCTAGCAGGGCCCGTTTAAACCCGCTGATC
AGCCTCGACTGTGCCTTCTAGTTGCCAGCCATCTGTTGTTTGCCCCTCCCCCGT
GCCTTCCTTGACCCTGGAAGGTGCCACTCCCACTGTCCTTTCCTAATAAAATG
AGGAAATTGCATCGCATTGTCTGAGTAGGTGTCATTCTATTCTGGGGGGTGGG
GTGGGGCAGGACAGCAAGGGGGAGGATTGGGAAGACAATAGCAGGCATGCT
GGGGATGCGGTGGGCTCTATGGCTTCTGAGGCGGAAAGAACCAGCTGGGGCT

CTAGGGGGTATCCCCACGCGCCCTGTAGCGGCGCATTAAGCGCGGCGGGTGT

GGTGGTTACGCGCAGCGTGACCGCTACACTTGCCAGCGCCCTAGCGCCCGCT

CCTTTCGCTTTCTTCCCTTCCTTTCTCGCCACGTTCGCCGGCTTTCCCCGTCAA

GCTCTAAATCGGGGGCTCCCTTTAGGGTTCCGATTTAGTGCTTTACGGCACCT

CGACCCCAAAAAACTTGATTAGGGTGATGGTTCACGTAGTGGGCCATCGCCC

TGATAGACGGTTTTTCGCCCTTTGACGTTGGAGTCCACGTTCTTTAATAGTGG

ACTCTTGTTCCAAACTGGAACAACACTCAACCCTATCTCGGTCTATTCTTTTG

ATTTATAAGGGATTTTGCCGATTTCGGCCTATTGGTTAAAAAATGAGCTGATT

TAACAAAAATTTAACGCGAATTAATTCTGTGGAATGTGTGTCAGTTAGGGTGT

GGAAAGTCCCCAGGCTCCCCAGCAGGCAGAAGTATGCAAAGCATGCATCTCA

ATTAGTCAGCAACCAGGTGTGGAAAGTCCCCAGGCTCCCCAGCAGGCAGAAG

TATGCAAAGCATGCATCTCAATTAGTCAGCAACCATAGTCCCGCCCCTAACTC

CGCCCATCCCGCCCCTAACTCCGCCCAGTTCCGCCCATTCTCCGCCCCATGGC

TGACTAATTTTTTTTATTTATGCAGAGGCCGAGGCCGCCTCTGCCTCTGAGCT

ATTCCAGAAGTAGTGAGGAGGCTTTTTTGGAGGCCTAGGCTTTTGCAAAAAG

CTCCCGGGAGCTTGTATATCCATTTTCGGATCTGATCAGCACGTGTTGACAAT

TAATCATCGGCATAGTATATCGGCATAGTATAATACGACAAGGTGAGGAACT

AAACCATGGCCAAGTTGACCAGTGCCGTTCCGGTGCTCACCGCGCGCGACGT

CGCCGGAGCGGTCGAGTTCTGGACCGACCGGCTCGGGTTCTCCCGGGACTTC

GTGGAGGACGACTTCGCCGGTGTGGTCCGGGACGACGTGACCCTGTTCATCA

GCGCGGTCCAGGACCAGGTGGTGCCGGACAACACCCTGGCCTGGGTGTGGGT

GCGCGGCCTGGACGAGCTGTACGCCGAGTGGTCGGAGGTCGTGTCCACGAAC

TTCCGGGACGCCTCCGGGCCGGCCATGACCGAGATCGGCGAGCAGCCGTGGG

GGCGGGAGTTCGCCCTGCGCGACCCGGCCGGCAACTGCGTGCACTTCGTGGC

CGAGGAGCAGGACTGACACGTGCTACGAGATTTCGATTCCACCGCCGCCTTC

TATGAAAGGTTGGGCTTCGGAATCGTTTTCCGGGACGCCGGCTGGATGATCCT

CCAGCGCGGGGATCTCATGCTGGAGTTCTTCGCCCACCCCAACTTGTTTATTG

CAGCTTATAATGGTTACAAATAAAGCAATAGCATCACAAATTTCACAAATAA

AGCATTTTTTTCACTGCATTCTAGTTGTGGTTTGTCCAAACTCATCAATGTATC

TTATCATGTCTGTATACCGTCGACCTCTAGCTAGAGCTTGGCGTAATCATGGT

CATAGCTGTTTCCTGTGTGAAATTGTTATCCGCTCACAATTCCACACAACATA

CGAGCCGGAAGCATAAAGTGTAAAGCCTGGGGTGCCTAATGAGTGAGCTAAC

TCACATTAATTGCGTTGCGCTCACTGCCCGCTTTCCAGTCGGGAAACCTGTCG

TGCCAGCTGCATTAATGAATCGGCCAACGCGCGGGGAGAGGCGGTTTGCGTA

TTGGGCGCTCTTCCGCTTCCTCGCTCACTGACTCGCTGCGCTCGGTCGTTCGG

CTGCGGCGAGCGGTATCAGCTCACTCAAAGGCGGTAATACGGTTATCCACAG

AATCAGGGGATAACGCAGGAAAGAACATGTGAGCAAAAGGCCAGCAAAAGG

CCAGGAACCGTAAAAAGGCCGCGTTGCTGGCGTTTTTCCATAGGCTCCGCCC

CCCTGACGAGCATCACAAAAATCGACGCTCAAGTCAGAGGTGGCGAAACCC

GACAGGACTATAAAGATACCAGGCGTTTCCCCCTGGAAGCTCCCTCGTGCGC

TCTCCTGTTCCGACCCTGCCGCTTACCGGATACCTGTCCGCCTTTCTCCCTTCG

GGAAGCGTGGCGCTTTCTCATAGCTCACGCTGTAGGTATCTCAGTTCGGTGTA

GGTCGTTCGCTCCAAGCTGGGCTGTGTGCACGAACCCCCCGTTCAGCCCGAC

CGCTGCGCCTTATCCGGTAACTATCGTCTTGAGTCCAACCCGGTAAGACACG

ACTTATCGCCACTGGCAGCAGCCACTGGTAACAGGATTAGCAGAGCGAGGTA

TGTAGGCGGTGCTACAGAGTTCTTGAAGTGGTGGCCTAACTACGGCTACACT

AGAAGAACAGTATTTGGTATCTGCGCTCTGCTGAAGCCAGTTACCTTCGGAA
AAAGAGTTGGTAGCTCTTGATCCGGCAAACAAACCACCGCTGGTAGCGGTGG
TTTTTTTGTTTGCAAGCAGCAGATTACGCGCAGAAAAAAAGGATCTCAAGAA
GATCCTTTGATCTTTTCTACGGGGTCTGACGCTCAGTGGAACGAAAACTCACG
TTAAGGGATTTTGGTCATGAGATTATCAAAAAGGATCTTCACCTAGATCCTTT
TAAATTAAAAATGAAGTTTTAAATCAATCTAAAGTATATATGAGTAAACTTG
GTCTGACAGTTACCAATGCTTAATCAGTGAGGCACCTATCTCAGCGATCTGTC
TATTTCGTTCATCCATAGTTGCCTGACTCCCCGTCGTGTAGATAACTACGATA
CGGGAGGGCTTACCATCTGGCCCCAGTGCTGCAATGATACCGCGAGACCCAC
GCTCACCGGCTCCAGATTTATCAGCAATAAACCAGCCAGCCGGAAGGGCCGA
GCGCAGAAGTGGTCCTGCAACTTTATCCGCCTCCATCCAGTCTATTAATTGTT
GCCGGGAAGCTAGAGTAAGTAGTTCGCCAGTTAATAGTTTGCGCAACGTTGT
TGCCATTGCTACAGGCATCGTGGTGTCACGCTCGTCGTTTGGTATGGCTTCAT
TCAGCTCCGGTTCCCAACGATCAAGGCGAGTTACATGATCCCCCATGTTGTGC
AAAAAAGCGGTTAGCTCCTTCGGTCCTCCGATCGTTGTCAGAAGTAAGTTGG
CCGCAGTGTTATCACTCATGGTTATGGCAGCACTGCATAATTCTCTTACTGTC
ATGCCATCCGTAAGATGCTTTTCTGTGACTGGTGAGTACTCAACCAAGTCATT
CTGAGAATAGTGTATGCGGCGACCGAGTTGCTCTTGCCCGGCGTCAATACGG
GATAATACCGCGCCACATAGCAGAACTTTAAAAGTGCTCATCATTGGAAAAC
GTTCTTCGGGGCGAAAACTCTCAAGGATCTTACCGCTGTTGAGATCCAGTTCG
ATGTAACCCACTCGTGCACCCAACTGATCTTCAGCATCTTTTACTTTCACCAG
CGTTTCTGGGTGAGCAAAAACAGGAAGGCAAAATGCCGCAAAAAAGGGAAT
AAGGGCGACACGGAAATGTTGAATACTCATACTCTTCCTTTTTCAATATTATT

GAAGCATTTATCAGGGTTATTGTCTCATGAGCGGATACATATTTGAATGTATT

TAGAAAAATAAACAAATAGGGGTTCCGCGCACATTTCCCCGAAAAGTGCCAC

CTGAC

Plasmids used in construction of pSLIK2

>pUC19::Crimson/P2A-NLS/HA-HumBeta

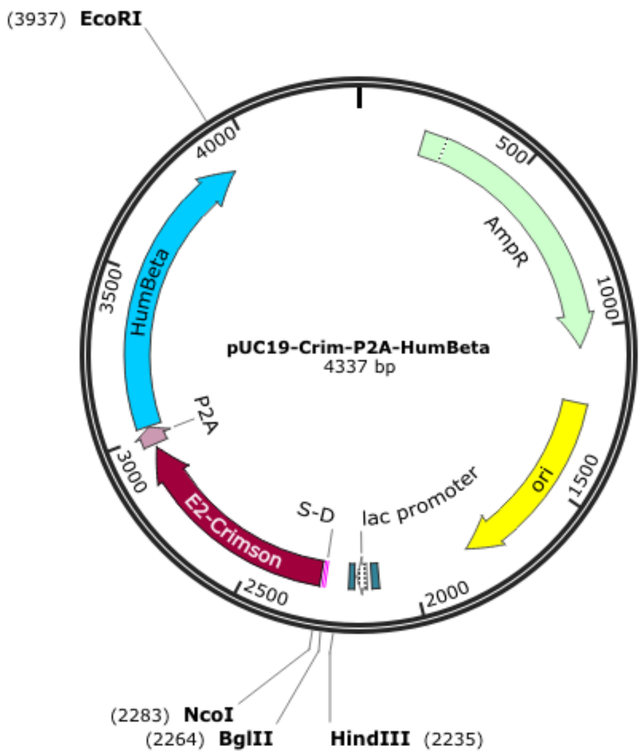

>pUC19::Crimson/P2A-NLS/HA-HumBeta

GACGAAAGGGCCTCGTGATACGCCTATTTTTATAGGTTAATGTCATGATAATA

ATGGTTTCTTAGACGTCAGGTGGCACTTTTCGGGGAAATGTGCGCGGAACCC

CTATTTGTTTATTTTTCTAAATACATTCAAATATGTATCCGCTCATGAGACAAT

AACCCTGATAAATGCTTCAATAATATTGAAAAAGGAAGAGTATGAGTATTCA

ACATTTCCGTGTCGCCCTTATTCCCTTTTTTGCGGCATTTTGCCTTCCTGTTTTT

GCTCACCCAGAAACGCTGGTGAAAGTAAAAGATGCTGAAGATCAGTTGGGTG

CACGAGTGGGTTACATCGAACTGGATCTCAACAGCGGTAAGATCCTTGAGAG

TTTTCGCCCCGAAGAACGTTTTCCAATGATGAGCACTTTTAAAGTTCTGCTAT

GTGGCGCGGTATTATCCCGTATTGACGCCGGGCAAGAGCAACTCGGTCGCCG

CATACACTATTCTCAGAATGACTTGGTTGAGTACTCACCAGTCACAGAAAAG

CATCTTACGGATGGCATGACAGTAAGAGAATTATGCAGTGCTGCCATAACCA

TGAGTGATAACACTGCGGCCAACTTACTTCTGACAACGATCGGAGGACCGAA

GGAGCTAACCGCTTTTTTGCACAACATGGGGGATCATGTAACTCGCCTTGATC

GTTGGGAACCGGAGCTGAATGAAGCCATACCAAACGACGAGCGTGACACCA

CGATGCCTGTAGCAATGGCAACAACGTTGCGCAAACTATTAACTGGCGAACT

ACTTACTCTAGCTTCCCGGCAACAATTAATAGACTGGATGGAGGCGGATAAA

GTTGCAGGACCACTTCTGCGCTCGGCCCTTCCGGCTGGCTGGTTTATTGCTGA

TAAATCTGGAGCCGGTGAGCGTGGGTCTCGCGGTATCATTGCAGCACTGGGG

CCAGATGGTAAGCCCTCCCGTATCGTAGTTATCTACACGACGGGGAGTCAGG

CAACTATGGATGAACGAAATAGACAGATCGCTGAGATAGGTGCCTCACTGAT

TAAGCATTGGTAACTGTCAGACCAAGTTTACTCATATATACTTTAGATTGATT

TAAAACTTCATTTTTAATTTAAAAGGATCTAGGTGAAGATCCTTTTTGATAAT

CTCATGACCAAAATCCCTTAACGTGAGTTTTCGTTCCACTGAGCGTCAGACCC

CGTAGAAAAGATCAAAGGATCTTCTTGAGATCCTTTTTTTCTGCGCGTAATCT

GCTGCTTGCAAACAAAAAAACCACCGCTACCAGCGGTGGTTTGTTTGCCGGA

TCAAGAGCTACCAACTCTTTTTCCGAAGGTAACTGGCTTCAGCAGAGCGCAG

ATACCAAATACTGTTCTTCTAGTGTAGCCGTAGTTAGGCCACCACTTCAAGAA

CTCTGTAGCACCGCCTACATACCTCGCTCTGCTAATCCTGTTACCAGTGGCTG

CTGCCAGTGGCGATAAGTCGTGTCTTACCGGGTTGGACTCAAGACGATAGTT

ACCGGATAAGGCGCAGCGGTCGGGCTGAACGGGGGGTTCGTGCACACAGCC

CAGCTTGGAGCGAACGACCTACACCGAACTGAGATACCTACAGCGTGAGCTA

TGAGAAAGCGCCACGCTTCCCGAAGGGAGAAAGGCGGACAGGTATCCGGTA

AGCGGCAGGGTCGGAACAGGAGAGCGCACGAGGGAGCTTCCAGGGGGAAAC

GCCTGGTATCTTTATAGTCCTGTCGGGTTTCGCCACCTCTGACTTGAGCGTCG

ATTTTTGTGATGCTCGTCAGGGGGGCGGAGCCTATGGAAAAACGCCAGCAAC

GCGGCCTTTTTACGGTTCCTGGCCTTTTGCTGGCCTTTTGCTCACATGTTCTTT

CCTGCGTTATCCCCTGATTCTGTGGATAACCGTATTACCGCCTTTGAGTGAGC

TGATACCGCTCGCCGCAGCCGAACGACCGAGCGCAGCGAGTCAGTGAGCGA

GGAAGCGGAAGAGCGCCCAATACGCAAACCGCCTCTCCCCGCGCGTTGGCCG

ATTCATTAATGCAGCTGGCACGACAGGTTTCCCGACTGGAAAGCGGGCAGTG

AGCGCAACGCAATTAATGTGAGTTAGCTCACTCATTAGGCACCCCAGGCTTT

ACACTTTATGCTTCCGGCTCGTATGTTGTGTGGAATTGTGAGCGGATAACAAT

TTCACACAGGAAACAGCTATGACCATGATTACGCCAAGCTTTACGTACCACC

ATGTGCGATCGCAGATCTAGGAGGTCCGCCACCATGGATAGCACTGAGAACG

TCATCAAGCCCTTCATGCGCTTCAAGGTGCACATGGAGGGCTCCGTGAACGG

CCACGAGTTCGAGATCGAGGGCGTGGGCGAGGGCAAGCCCTACGAGGGCAC

CCAGACCGCCAAGCTGCAAGTGACCAAGGGCGGCCCCCTGCCCTTCGCCTGG

GACATCCTGTCCCCCCAGTTCTTCTACGGCTCCAAGGCGTACATCAAGCACCC

CGCCGACATCCCCGACTACCTCAAGCAGTCCTTCCCCGAGGGCTTCAAGTGG

GAGCGCGTGATGAACTTCGAGGACGGCGGCGTGGTGACCGTGACCCAGGACT

CCTCCCTGCAGGACGGCACCCTCATCTACCACGTGAAGTTCATCGGCGTGAA

CTTCCCCTCCGACGGCCCCGTAATGCAGAAGAAGACTCTGGGCTGGGAGCCC

TCCACTGAGCGCAACTACCCCCGCGACGGCGTGCTGAAGGGCGAGAACCACA

TGGCGCTGAAGCTGAAGGGCGGCGGCCACTACCTGTGTGAGTTCAAGTCCAT

CTACATGGCCAAGAAGCCCGTGAAGCTGCCCGGCTACCACTACGTGGACTAC

AAGCTCGACATCACCTCCCACAACGAGGACTACACCGTGGTGGAGCAGTACG

AGCGCGCCGAGGCCCGCCACCACCTGTTCCAGGGATCCGGAGCCACGAACTT

CTCTCTGTTAAAGCAAGCAGGAGACGTGGAAGAAAACCCCGGTCCTCACATG

GTGCCTCCCAAGAAGAAGAGGAAGGTCGAGGACCCCAAGTACCCCTATGAC

GTGCCCGATTACGCTGGCGGAGGCGGAGGCTCCGGCGGAGGAGGAGGATCC

GGAGGAGGCAGCATGTCCACAGCTCTCGCCACCCTGGCCGGAAAACTGGCCG

AGAGAGTGGGCATGGACTCCGTGGATCCCCAGGAGCTGATCACCACACTGAG

GCAAACCGCCTTCAAGGGAGATGCTTCCGACGCCCAATTCATCGCCCTCCTC

ATCGTGGCCAACCAGTACGGCCTGAACCCTTGGACCAAGGAGATTTACGCCT

TCCCCGACAAGCAGAATGGCATTGTCCCTGTGGTCGGCGTGGACGGCTGGAG

CAGAATCATTAACGAGAACCAGCAGTTTGACGGCATGGACTTCGAGCAGGAC

AACGAGAGCTGCACATGCAGGATCTACAGGAAGGACAGGAACCATCCTATCT

GCGTCACCGAGTGGATGGATGAGTGCAGAAGAGAACCCTTCAAGACCAGAG

AGGGCAGAGAGATCACAGGACCCTGGCAGTCCCATCCTAAGAGAATGCTCA

GACACAAGGCCATGATCCAGTGTGCCAGGCTCGCTTTTGGCTTCGCCGGCAT

CTATGATAAGGACGAAGCCGAGAGGATCGTGGAAAATACCGCCTACACCGCT

GAAAGGCAGCCCGAAAGGGACATTACACCCGTGAACGACGAGACCATGCAG

GAGATCAATACCCTGCTCATTGCCCTGGACAAAACCTGGGACGATGACCTCC

TCCCCCTCTGCAGCCAAATCTTCAGGAGGGACATCAGGGCCAGCTCCGAACT

CACACAAGCCGAAGCTGTGAAGGCCCTCGGATTCCTCAAACAGAAGGCCGCT
GAGCAGAAGGTCGCCGCCGTTTAAACGAATTCACTGGCCGTCGTTTTACAAC
GTCGTGACTGGGAAAACCCTGGCGTTACCCAACTTAATCGCCTTGCAGCACA
TCCCCCTTTCGCCAGCTGGCGTAATAGCGAAGAGGCCCGCACCGATCGCCCT
TCCCAACAGTTGCGCAGCCTGAATGGCGAATGGCGCCTGATGCGGTATTTTCT
CCTTACGCATCTGTGCGGTATTTCACACCGCATATGGTGCACTCTCAGTACAA
TCTGCTCTGATGCCGCATAGTTAAGCCAGCCCCGACACCCGCCAACACCCGC
TGACGCGCCCTGACGGGCTTGTCTGCTCCCGGCATCCGCTTACAGACAAGCT
GTGACCGTCTCCGGGAGCTGCATGTGTCAGAGGTTTTCACCGTCATCACCGAA
ACGCGCGA

>pENTR2B/TREPitt::Crimson/P2A-NLS/HA-HumBeta

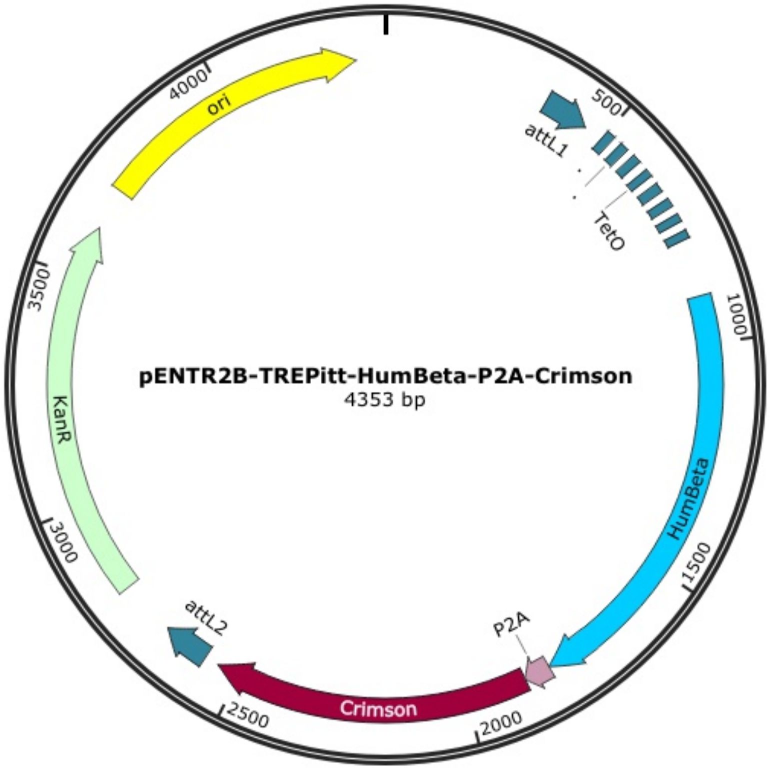

>pENTR2B/TREPitt::Crimson/P2A-NLS/HA-HumBeta

CTTTCCTGCGTTATCCCCTGATTCTGTGGATAACCGTATTACCGCTAGCATGG

ATCTCGGGGACGTCTAACTACTAAGCGAGAGTAGGGAACTGCCAGGCATCAA

ATAAAACGAAAGGCTCAGTCGGAAGACTGGGCCTTTCGTTTTATCTGTTGTTT

GTCGGTGAACGCTCTCCTGAGTAGGACAAATCCGCCGGGAGCGGATTTGAAC

GTTGTGAAGCAACGGCCCGGAGGGTGGCGGGCAGGACGCCCGCCATAAACT

GCCAGGCATCAAACTAAGCAGAAGGCCATCCTGACGGATGGCCTTTTTGCGT

TTCTACAAACTCTTCCTGTTAGTTAGTTACTTAAGCTCGGGCCCCAAATAATG

ATTTTATTTTGACTGATAGTGACCTGTTCGTTGCAACAAATTGATAAGCAATG

CTTTTTTATAATGCCAACTTTGTACAAAAAAGCAGGCTGGCGCCGGAACCAA

TTCAgatatcAAGTGCCACCTGACGTCTCCCTATCAGTGATAGAGAAGTCGACAC

GTCTCGAGCTCCCTATCAGTGATAGAGAAGGTACGTCTAGAACGTCTCCCTAT

CAGTGATAGAGAAGTCGACACGTCTCGAGCTCCCTATCAGTGATAGAGAAGG

TACGTCTAGAACGTCTCCCTATCAGTGATAGAGAAGTCGACACGTCTCGAGC

TCCCTATCAGTGATAGAGAAGGTACGTCTAGAACGTCTCCCTATCAGTGATA

GAGAAGTCGACACGTCTCGAGCTCCCTATCAGTGATAGAGAAGGTACCCCCT

ATATAAGCAGAGCTCGTTTAGTGAACCGTCAGATCGCCTGGAGACGCCATCC

ACGCTGTTTTGACCTCCATAGAAGACACCGGGACCGATCCAGCCTGGATCTA

GGAGGTCCGCCACCATGGATAGCACTGAGAACGTCATCAAGCCCTTCATGCG

CTTCAAGGTGCACATGGAGGGCTCCGTGAACGGCCACGAGTTCGAGATCGAG

GGCGTGGGCGAGGGCAAGCCCTACGAGGGCACCCAGACCGCCAAGCTGCAA

GTGACCAAGGGCGGCCCCCTGCCCTTCGCCTGGGACATCCTGTCCCCCCAGTT

CTTCTACGGCTCCAAGGCGTACATCAAGCACCCCGCCGACATCCCCGACTAC

CTCAAGCAGTCCTTCCCCGAGGGCTTCAAGTGGGAGCGCGTGATGAACTTCG

AGGACGGCGGCGTGGTGACCGTGACCCAGGACTCCTCCCTGCAGGACGGCAC

CCTCATCTACCACGTGAAGTTCATCGGCGTGAACTTCCCCTCCGACGGCCCCG

TAATGCAGAAGAAGACTCTGGGCTGGGAGCCCTCCACTGAGCGCAACTACCC

CCGCGACGGCGTGCTGAAGGGCGAGAACCACATGGCGCTGAAGCTGAAGGG

CGGCGGCCACTACCTGTGTGAGTTCAAGTCCATCTACATGGCCAAGAAGCCC

GTGAAGCTGCCCGGCTACCACTACGTGGACTACAAGCTCGACATCACCTCCC

ACAACGAGGACTACACCGTGGTGGAGCAGTACGAGCGCGCCGAGGCCCGCC

ACCACCTGTTCCAGGGATCCGGAGCCACGAACTTCTCTCTGTTAAAGCAAGC

AGGAGACGTGGAAGAAAACCCCGGTCCTCACATGGTGCCTCCCAAGAAGAA

GAGGAAGGTCGAGGACCCCAAGTACCCCTATGACGTGCCCGATTACGCTGGC

GGAGGCGGAGGCTCCGGCGGAGGAGGAGGATCCGGAGGAGGCAGCATGTCC

ACAGCTCTCGCCACCCTGGCCGGAAAACTGGCCGAGAGAGTGGGCATGGACT

CCGTGGATCCCCAGGAGCTGATCACCACACTGAGGCAAACCGCCTTCAAGGG

AGATGCTTCCGACGCCCAATTCATCGCCCTCCTCATCGTGGCCAACCAGTACG

GCCTGAACCCTTGGACCAAGGAGATTTACGCCTTCCCCGACAAGCAGAATGG

CATTGTCCCTGTGGTCGGCGTGGACGGCTGGAGCAGAATCATTAACGAGAAC

CAGCAGTTTGACGGCATGGACTTCGAGCAGGACAACGAGAGCTGCACATGCA

GGATCTACAGGAAGGACAGGAACCATCCTATCTGCGTCACCGAGTGGATGGA

TGAGTGCAGAAGAGAACCCTTCAAGACCAGAGAGGGCAGAGAGATCACAGG

ACCCTGGCAGTCCCATCCTAAGAGAATGCTCAGACACAAGGCCATGATCCAG

TGTGCCAGGCTCGCTTTTGGCTTCGCCGGCATCTATGATAAGGACGAAGCCG

AGAGGATCGTGGAAAATACCGCCTACACCGCTGAAAGGCAGCCCGAAAGGG

ACATTACACCCGTGAACGACGAGACCATGCAGGAGATCAATACCCTGCTCAT

TGCCCTGGACAAAACCTGGGACGATGACCTCCTCCCCCTCTGCAGCCAAATC

TTCAGGAGGGACATCAGGGCCAGCTCCGAACTCACACAAGCCGAAGCTGTGA

AGGCCCTCGGATTCCTCAAACAGAAGGCCGCTGAGCAGAAGGTCGCCGCCGT

TTAAACGAATTCGCGGCCGCACTCGAGATATCTAGACCCAGCTTTCTTGTACA

AAGTTGGCATTATAAGAAAGCATTGCTTATCAATTTGTTGCAACGAACAGGT

CACTATCAGTCAAAATAAAATCATTATTTGCCATCCAGCTGCAGCTCTGGCCC

GTGTCTCAAAATCTCTGATGTTACATTGCACAAGATAAAAATATATCATCATG

AACAATAAAACTGTCTGCTTACATAAACAGTAATACAAGGGGTGTTATGAGC

CATATTCAACGGGAAACGTCGAGGCCGCGATTAAATTCCAACATGGATGCTG

ATTTATATGGGTATAAATGGGCTCGCGATAATGTCGGGCAATCAGGTGCGAC

AATCTATCGCTTGTATGGGAAGCCCGATGCGCCAGAGTTGTTTCTGAAACATG

GCAAAGGTAGCGTTGCCAATGATGTTACAGATGAGATGGTCAGACTAAACTG

GCTGACGGAATTTATGCCTCTTCCGACCATCAAGCATTTTATCCGTACTCCTG

ATGATGCATGGTTACTCACCACTGCGATCCCCGGAAAAACAGCATTCCAGGT

ATTAGAAGAATATCCTGATTCAGGTGAAAATATTGTTGATGCGCTGGCAGTG

TTCCTGCGCCGGTTGCATTCGATTCCTGTTTGTAATTGTCCTTTTAACAGCGAT

CGCGTATTTCGTCTCGCTCAGGCGCAATCACGAATGAATAACGGTTTGGTTGA

TGCGAGTGATTTTGATGACGAGCGTAATGGCTGGCCTGTTGAACAAGTCTGG

AAAGAAATGCATAAACTTTTGCCATTCTCACCGGATTCAGTCGTCACTCATGG

TGATTTCTCACTTGATAACCTTATTTTTGACGAGGGGAAATTAATAGGTTGTA

TTGATGTTGGACGAGTCGGAATCGCAGACCGATACCAGGATCTTGCCATCCT

ATGGAACTGCCTCGGTGAGTTTTCTCCTTCATTACAGAAACGGCTTTTTCAAA

AATATGGTATTGATAATCCTGATATGAATAAATTGCAGTTTCATTTGATGCTC
GATGAGTTTTTCTAATCAGAATTGGTTAATTGGTTGTAACATTATTCAGATTG
GGCCCCGTTCCACTGAGCGTCAGACCCCGTAGAAAAGATCAAAGGATCTTCT
TGAGATCCTTTTTTTCTGCGCGTAATCTGCTGCTTGCAAACAAAAAAACCACC
GCTACCAGCGGTGGTTTGTTTGCCGGATCAAGAGCTACCAACTCTTTTTCCGA
AGGTAACTGGCTTCAGCAGAGCGCAGATACCAAATACTGTTCTTCTAGTGTA
GCCGTAGTTAGGCCACCACTTCAAGAACTCTGTAGCACCGCCTACATACCTC
GCTCTGCTAATCCTGTTACCAGTGGCTGCTGCCAGTGGCGATAAGTCGTGTCT
TACCGGGTTGGACTCAAGACGATAGTTACCGGATAAGGCGCAGCGGTCGGGC
TGAACGGGGGGTTCGTGCACACAGCCCAGCTTGGAGCGAACGACCTACACCG
AACTGAGATACCTACAGCGTGAGCTATGAGAAAGCGCCACGCTTCCCGAAGG
GAGAAAGGCGGACAGGTATCCGGTAAGCGGCAGGGTCGGAACAGGAGAGCG
CACGAGGGAGCTTCCAGGGGGAAACGCCTGGTATCTTTATAGTCCTGTCGGG
TTTCGCCACCTCTGACTTGAGCGTCGATTTTTGTGATGCTCGTCAGGGGGGCG
GAGCCTATGGAAAAACGCCAGCAACGCGGCCTTTTTACGGTTCCTGGCCTTTT
GCTGGCCTTTTGCTCACATGTT

>pSLIK/TREPitt::Crimson/P2A-NLS/HA-HumBeta

AKS pSLIK-Zeo-TREPitt-Crimson-P2A-HumBeta or pSLIK2. Sequence verified from 2633 to 4868 bp.

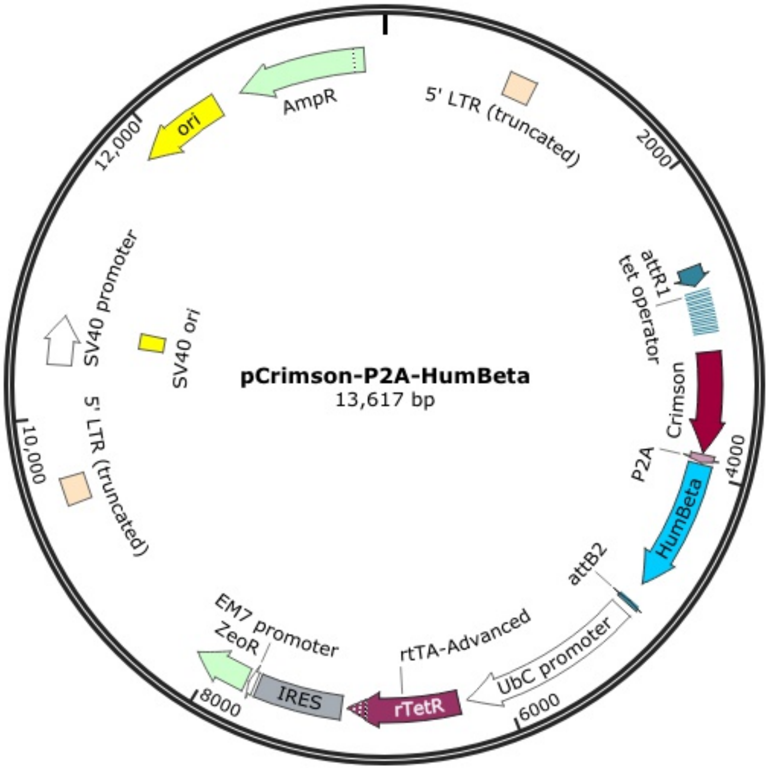

>2.pSLIK-Zeo/TREPitt::Crimson/P2A-HumBeta

GTCGACGGATCGGGAGATCTCCCGATCCCCTATGGTGCACTCTCAGTACAAT

CTGCTCTGATGCCGCATAGTTAAGCCAGTATCTGCTCCCTGCTTGTGTGTTGG

AGGTCGCTGAGTAGTGCGCGAGCAAAATTTAAGCTACAACAAGGCAAGGCTT

GACCGACAATTGCATGAAGAATCTGCTTAGGGTTAGGCGTTTTGCGCTGCTTC

GCGATGTACGGGCCAGATATACGCGTTGACATTGATTATTGACTAGTTATTAA

TAGTAATCAATTACGGGGTCATTAGTTCATAGCCCATATATGGAGTTCCGCGT

TACATAACTTACGGTAAATGGCCCGCCTGGCTGACCGCCCAACGACCCCCGC

CCATTGACGTCAATAATGACGTATGTTCCCATAGTAACGCCAATAGGGACTTT

CCATTGACGTCAATGGGTGGAGTATTTACGGTAAACTGCCCACTTGGCAGTA
CATCAAGTGTATCATATGCCAAGTACGCCCCCTATTGACGTCAATGACGGTA
AATGGCCCGCCTGGCATTATGCCCAGTACATGACCTTATGGGACTTTCCTACT
TGGCAGTACATCTACGTATTAGTCATCGCTATTACCATGGTGATGCGGTTTTG
GCAGTACATCAATGGGCGTGGATAGCGGTTTGACTCACGGGGATTTCCAAGT
CTCCACCCCATTGACGTCAATGGGAGTTTGTTTTGGCACCAAAATCAACGGG
ACTTTCCAAAATGTCGTAACAACTCCGCCCCATTGACGCAAATGGGCGGTAG
GCGTGTACGGTGGGAGGTCTATATAAGCAGCGTTTTGCCTGTACTGGGTCT
CTCTGGTTAGACCAGATCTGAGCCTGGGAGCTCTCTGGCTAACTAGGGAACC
CACTGCTTAAGCCTCAATAAAGCTTGCCTTGAGTGCTTCAAGTAGTGTGTGCC
CGTCTGTTGTGTGACTCTGGTAACTAGAGATCCCTCAGACCCTTTTAGTCAGT
GTGGAAAATCTCTAGCAGTGGCGCCCGAACAGGGACTTGAAAGCGAAAGGG
AAACCAGAGGAGCTCTCTCGACGCAGGACTCGGCTTGCTGAAGCGCGCACGG
CAAGAGGCGAGGGGCGGCGACTGGTGAGTACGCCAAAAATTTTGACTAGCG
GAGGCTAGAAGGAGAGAGATGGGTGCGAGAGCGTCAGTATTAAGCGGGGGA
GAATTAGATCGCGATGGGAAAAAATTCGGTTAAGGCCAGGGGGAAAGAAAA
AATATAAATTAAAACATATAGTATGGGCAAGCAGGGAGCTAGAACGATTCGC
AGTTAATCCTGGCCTGTTAGAAACATCAGAAGGCTGTAGACAAATACTGGGA
CAGCTACAACCATCCCTTCAGACAGGATCAGAAGAACTTAGATCATTATATA
ATACAGTAGCAACCCTCTATTGTGTGCATCAAAGGATAGAGATAAAAGACAC
CAAGGAAGCTTTAGACAAGATAGAGGAAGAGCAAAACAAAAGTAAGACCAC
CGCACAGCAAGCGGCCGCTGATCTTCAGACCTGGAGGAGGAGATATGAGGG
ACAATTGGAGAAGTGAATTATATAAATATAAAGTAGTAAAAATTGAACCATT

AGGAGTAGCACCCACCAAGGCAAAGAGAAGAGTGGTGCAGAGAGAAAAAA
GAGCAGTGGGAATAGGAGCTTTGTTCCTTGGGTTCTTGGGAGCAGCAGGAAG
CACTATGGGCGCAGCGTCAATGACGCTGACGGTACAGGCCAGACAATTATTG
TCTGGTATAGTGCAGCAGCAGAACAATTTGCTGAGGGCTATTGAGGCGCAAC
AGCATCTGTTGCAACTCACAGTCTGGGGCATCAAGCAGCTCCAGGCAAGAAT
CCTGGCTGTGGAAAGATACCTAAAGGATCAACAGCTCCTGGGGATTTGGGGT
TGCTCTGGAAAACTCATTTGCACCACTGCTGTGCCTTGGAATGCTAGTTGGAG
TAATAAATCTCTGGAACAGATTTGGAATCACACGACCTGGATGGAGTGGGAC
AGAGAAATTAACAATTACACAAGCTTAATACACTCCTTAATTGAAGAATCGC
AAAACCAGCAAGAAAAGAATGAACAAGAATTATTGGAATTAGATAAATGGG
CAAGTTTGTGGAATTGGTTTAACATAACAAATTGGCTGTGGTATATAAAATTA
TTCATAATGATAGTAGGAGGCTTGGTAGGTTTAAGAATAGTTTTTGCTGTACT
TTCTATAGTGAATAGAGTTAGGCAGGGATATTCACCATTATCGTTTCAGACCC
ACCTCCCAACCCCGAGGGGACCCGACAGGCCCGAAGGAATAGAAGAAGAAG
GTGGAGAGAGAGACAGAGACAGATCCATTCGATTAGTGAACGGATCGGCAC
TGCGTGCGCCAATTCTGCAGACAAATGGCAGTATTCATCCACAATTTTAAAA
GAAAAGGGGGGATTGGGGGGTACAGTGCAGGGGAAAGAATAGTAGACATAA
TAGCAACAGACATACAAACTAAAGAATTACAAAAACAAATTACAAAAATTC
AAAATTTTCGGGTTTATTACAGGGACAGCAGAGATCCAGTTTGGTTAATTCTA
GACAGTTATCAACAAGTTTGTACAAAAAAGCTGAACGAGAAACGTAAAATG
ATATAAATATCAATATATTAAATTAGATTTTGCATAAAAAACAGACTACATA
ATACTGTAAAACACAACATATCCAGTCACTATGGGCGCCGGAACCAATTCAG
ATATCAAGTGCCACCTGACGTCTCCCTATCAGTGATAGAGAAGTCGACACGT

CTCGAGCTCCCTATCAGTGATAGAGAAGGTACGTCTAGAACGTCTCCCTATCA
GTGATAGAGAAGTCGACACGTCTCGAGCTCCCTATCAGTGATAGAGAAGGTA
CGTCTAGAACGTCTCCCTATCAGTGATAGAGAAGTCGACACGTCTCGAGCTC
CCTATCAGTGATAGAGAAGGTACGTCTAGAACGTCTCCCTATCAGTGATAGA
GAAGTCGACACGTCTCGAGCTCCCTATCAGTGATAGAGAAGGTACCCCCTAT
ATAAGCAGAGCTCGTTTAGTGAACCGTCAGATCGCCTGGAGACGCCATCCAC
GCTGTTTTGACCTCCATAGAAGACACCGGGACCGATCCAGCCTGGATCTAGG
AGGTCCGCCACCATGGATAGCACTGAGAACGTCATCAAGCCCTTCATGCGCT
TCAAGGTGCACATGGAGGGCTCCGTGAACGGCCACGAGTTCGAGATCGAGGG
CGTGGGCGAGGGCAAGCCCTACGAGGGCACCCAGACCGCCAAGCTGCAAGT
GACCAAGGGCGGCCCCCTGCCCTTCGCCTGGGACATCCTGTCCCCCCAGTTCT
TCTACGGCTCCAAGGCGTACATCAAGCACCCCGCCGACATCCCCGACTACCT
CAAGCAGTCCTTCCCCGAGGGCTTCAAGTGGGAGCGCGTGATGAACTTCGAG
GACGGCGGCGTGGTGACCGTGACCCAGGACTCCTCCCTGCAGGACGGCACCC
TCATCTACCACGTGAAGTTCATCGGCGTGAACTTCCCCTCCGACGGCCCCGTA
ATGCAGAAGAAGACTCTGGGCTGGGAGCCCTCCACTGAGCGCAACTACCCCC
GCGACGGCGTGCTGAAGGGCGAGAACCACATGGCGCTGAAGCTGAAGGGCG
GCGGCCACTACCTGTGTGAGTTCAAGTCCATCTACATGGCCAAGAAGCCCGT
GAAGCTGCCCGGCTACCACTACGTGGACTACAAGCTCGACATCACCTCCCAC
AACGAGGACTACACCGTGGTGGAGCAGTACGAGCGCGCCGAGGCCCGCCAC
CACCTGTTCCAGGGATCCGGAGCCACGAACTTCTCTCTGTTAAAGCAAGCAG
GAGACGTGGAAGAAAACCCCGGTCCTCACATGGTGCCTCCCAAGAAGAAGA
GGAAGGTCGAGGACCCCAAGTACCCCTATGACGTGCCCGATTACGCTGGCGG

AGGCGGAGGCTCCGGCGGAGGAGGAGGATCCGGAGGAGGCAGCATGTCCAC

AGCTCTCGCCACCCTGGCCGGAAAACTGGCCGAGAGAGTGGGCATGGACTCC

GTGGATCCCCAGGAGCTGATCACCACACTGAGGCAAACCGCCTTCAAGGGAG

ATGCTTCCGACGCCCAATTCATCGCCCTCCTCATCGTGGCCAACCAGTACGGC

CTGAACCCTTGGACCAAGGAGATTTACGCCTTCCCCGACAAGCAGAATGGCA

TTGTCCCTGTGGTCGGCGTGGACGGCTGGAGCAGAATCATTAACGAGAACCA

GCAGTTTGACGGCATGGACTTCGAGCAGGACAACGAGAGCTGCACATGCAGG

ATCTACAGGAAGGACAGGAACCATCCTATCTGCGTCACCGAGTGGATGGATG

AGTGCAGAAGAGAACCCTTCAAGACCAGAGAGGGCAGAGAGATCACAGGAC

CCTGGCAGTCCCATCCTAAGAGAATGCTCAGACACAAGGCCATGATCCAGTG

TGCCAGGCTCGCTTTTGGCTTCGCCGGCATCTATGATAAGGACGAAGCCGAG

AGGATCGTGGAAAATACCGCCTACACCGCTGAAAGGCAGCCCGAAAGGGAC

ATTACACCCGTGAACGACGAGACCATGCAGGAGATCAATACCCTGCTCATTG

CCCTGGACAAAACCTGGGACGATGACCTCCTCCCCCTCTGCAGCCAAATCTTC

AGGAGGGACATCAGGGCCAGCTCCGAACTCACACAAGCCGAAGCTGTGAAG

GCCCTCGGATTCCTCAAACAGAAGGCCGCTGAGCAGAAGGTCGCCGCCGTTT

AAACGAATTCGCGGCCGCACTCGAGATATCTAGATAGTGACTGGATATGTTG

TGTTTTACAGTATTATGTAGTCTGTTTTTTATGCAAAATCTAATTTAATATATT

GATATTTATATCATTTTACGTTTCTCGTTCAGCTTTCTTGTACAAAGTGGTTGA

TAACATCTCGAGCCATAAAGATGGTTAATTAACCCACCCAAGATCTGGCCTC

CGCGCCGGGTTTTGGCGCCTCCCGCGGGCGCCCCCCTCCTCACGGCGAGCGC

TGCCACGTCAGACGAAGGGCGCAGCGAGCGTCCTGATCCTTCCGCCCGGACG

CTCAGGACAGCGGCCCGCTGCTCATAAGACTCGGCCTTAGAACCCCAGTATC

AGCAGAAGGACATTTTAGGACGGGACTTGGGTGACTCTAGGGCACTGGTTTT

CTTTCCAGAGAGCGGAACAGGCGAGGAAAAGTAGTCCCTTCTCGGCGATTCT

GCGGAGGGATCTCCGTGGGGCGGTGAACGCCGATGATTATATAAGGACGCGC

CGGGTGTGGCACAGCTAGTTCCGTCGCAGCCGGGATTTGGGTCGCGGTTCTTG

TTTGTGGATCGCTGTGATCGTCACTTGGTGAGTAGCGGGCTGCTGGGCTGGCC

GGGGCTTTCGTGGCCGCCGGGCCGCTCGGTGGGACGGAAGCGTGTGGAGAGA

CCGCCAAGGGCTGTAGTCTGGGTCCGCGAGCAAGGTTGCCCTGAACTGGGGG

TTGGGGGGAGCGCAGCAAAATGGCGGCTGTTCCCGAGTCTTGAATGGAAGAC

GCTTGTGAGGCGGGCTGTGAGGTCGTTGAAACAAGGTGGGGGGCATGGTGGG

CGGCAAGAACCCAAGGTCTTGAGGCCTTCGCTAATGCGGGAAAGCTCTTATT

CGGGTGAGATGGGCTGGGGCACCATCTGGGGACCCTGACGTGAAGTTTGTCA

CTGACTGGAGAACTCGGTTTGTCGTCTGTTGCGGGGGCGGCAGTTATGGCGG

TGCCGTTGGGCAGTGCACCCGTACCTTTGGGAGCGCGCGCCCTCGTCGTGTCG

TGACGTCACCCGTTCTGTTGGCTTATAATGCAGGGTGGGGCCACCTGCCGGTA

GGTGTGCGGTAGGCTTTTCTCCGTCGCAGGACGCAGGGTTCGGGCCTAGGGT

AGGCTCTCCTGAATCGACAGGCGCCGGACCTCTGGTGAGGGGAGGGATAAGT

GAGGCGTCAGTTTCTTTGGTCGGTTTTATGTACCTATCTTCTTAAGTAGCTGA

AGCTCCGGTTTTGAACTATGCGCTCGGGGTTGGCGAGTGTGTTTTGTGAAGTT

TTTTAGGCACCTTTTGAAATGTAATCATTTGGGTCAATATGTAATTTTCAGTGT

TAGACTAGTAAATTGTCCGCTAAATTCTGGCCGTTTTTGGCTTTTTTGTTAGAC

GAAGCTTGGTACCGAGCTCGGATCTCCACCCCGTACCGGTCCTGCAGTCGAA

TTCACCATGTCTAGACTGGACAAGAGCAAAGTCATAAACGGAGCTCTGGAAT

TACTCAATGGTGTCGGTATCGAAGGCCTGACGACAAGGAAACTCGCTCAAAA

GCTGGGAGTTGAGCAGCCTACCCTGTACTGGCACGTGAAGAACAAGCGGGCC
CTGCTCGATGCCCTGCCAATCGAGATGCTGGACAGGCATCATACCCACTTCTG
CCCCCTGGAAGGCGAGTCATGGCAAGACTTTCTGCGGAACAACGCCAAGTCA
TACCGCTGTGCTCTCCTCTCACATCGCGACGGGGCTAAAGTGCATCTCGGCAC
CCGCCCAACAGAGAAACAGTACGAAACCCTGGAAAATCAGCTCGCGTTCCTG
TGTCAGCAAGGCTTCTCCCTGGAGAACGCACTGTACGCTCTGTCCGCCGTGG
GCCACTTTACACTGGGCTGCGTATTGGAGGAACAGGAGCATCAAGTAGCAAA
AGAGGAAAGAGAGACACCTACCACCGATTCTATGCCCCCACTTCTGAGACAA
GCAATTGAGCTGTTCGACCGGCAGGGAGCCGAACCTGCCTTCCTTTTCGGCCT
GGAACTAATCATATGTGGCCTGGAGAAACAGCTAAAGTGCGAAAGCGGCGG
GCCGACCGACGCCCTTGACGATTTTGACTTAGACATGCTCCCAGCCGATGCCC
TTGACGACTTTGACCTTGATATGCTGCCTGCTGACGCTCTTGACGATTTTGAC
CTTGACATGCTCCCCGGGTAACTAAGTAAGGATCCGCGGCCGCACTAGAGGA
ATTCCGCCCCTCTCCCTCCCCCCCCCCTAACGTTACTGGCCGAAGCCGCTTGG
AATAAGGCCGGTGTGTGTTTGTCTATATGTTATTTTCCACCATATTGCCGTCTT
TTGGCAATGTGAGGGCCCGGAAACCTGGCCCTGTCTTCTTGACGAGCATTCCT
AGGGGTCTTTCCCCTCTCGCCAAAGGAATGCAAGGTCTGTTGAATGTCGTGA
AGGAAGCAGTTCCTCTGGAAGCTTCTTGAAGACAAACAACGTCTGTAGCGAC
CCTTTGCAGGCAGCGGAACCCCCCACCTGGCGACAGGTGCCTCTGCGGCCAA
AAGCCACGTGTATAAGATACACCTGCAAAGGCGGCACAACCCCAGTGCCACG
TTGTGAGTTGGATAGTTGTGGAAAGAGTCAAATGGCTCTCCTCAAGCGTAGT
CAACAAGGGGCTGAAGGATGCCCAGAAGGTACCCCATTGTATGGGAATCTGA
TCTGGGGCCTCGGTGCACATGCTTTACATGTGTTTAGTCGAGGTTAAAAAAC

GTCTAGGCCCCCCGAACCACGGGGACGTGGTTTTCCTTTGAAAAACACGATG
ATAAGCTTACCGGTACGCGTGATGTGTTGACAATTAATCATCGGCATAGTATA
TCGGCATAGTATAATACGACAAGGTGAGGAACTAAACCATGGCCAAGTTGAC
CAGTGCCGTTCCGGTGCTCACCGCGCGCGACGTCGCCGGAGCGGTCGAGTTC
TGGACCGACCGGCTCGGGTTCTCCCGGGACTTCGTGGAGGACGACTTCGCCG
GTGTGGTCCGGGACGACGTGACCCTGTTCATCAGCGCGGTCCAGGACCAGGT
GGTGCCGGACAACACCCTGGCCTGGGTGTGGGTGCGCGGCCTGGACGAGCTG
TACGCCGAGTGGTCGGAGGTCGTGTCCACGAACTTCCGGGACGCCTCCGGGC
CGGCCATGACCGAGATCGGCGAGCAGCCGTGGGGGCGGGAGTTCGCCCTGC
GCGACCCGGCCGGCAACTGCGTGCACTTCGTGGCCGAGGAGCAGGACTGACA
CATCTGTACAAGTAAAGCGGCCGCGACTCTAGATCATAATCAGCCATACCAC
ATTTGTAGAGGTTTTACTTGCTTTAAAAAACCTCCCACACCTCCCCCTGAACC
TGAAACATAAAATGAATGCAATTGTTGTTGTTTAGTCCCTCCCAATTCGATAT
CAAGCTTATCGATAATCAACCTCTGGATTACAAAATTTGTGAAAGATTGACTG
GTATTCTTAACTATGTTGCTCCTTTTACGCTATGTGGATACGCTGCTTTAATGC
CTTTGTATCATGCTATTGCTTCCCGTATGGCTTTCATTTTCTCCTCCTTGTATA
AATCCTGGTTGCTGTCTCTTTATGAGGAGTTGTGGCCCGTTGTCAGGCAACGT
GGCGTGGTGTGCACTGTGTTTGCTGACGCAACCCCCACTGGTTGGGGCATTGC
CACCACCTGTCAGCTCCTTTCCGGGACTTTCGCTTTCCCCCTCCCTATTGCCAC
GGCGGAACTCATCGCCGCCTGCCTTGCCCGCTGCTGGACAGGGGCTCGGCTG
TTGGGCACTGACAATTCCGTGGTGTTGTCGGGGAAATCATCGTCCTTTCCTTG
GCTGCTCGCCTGTGTTGCCACCTGGATTCTGCGCGGGACGTCCTTCTGCTACG
TCCCTTCGGCCCTCAATCCAGCGGACCTTCCTTCCCGCGGCCTGCTGCCGGCT

CTGCGGCCTCTTCCGCGTCTTCGCCTTCGCCCTCAGACGAGTCGGATCTCCCT

TTGGGCCGCCTCCCCGCATCGATACCGTCGACCTCGATCGAGACCTAGAAAA

ACATGGAGCAATCACAAGTAGCAATACAGCAGCTACCAATGCTGATTGTGCC

TGGCTAGAAGCACAAGAGGAGGAGGAGGTGGGTTTTCCAGTCACACCTCAGG

TACCTTTAAGACCAATGACTTACAAGGCAGCTGTAGATCTTAGCCACTTTTTA

AAAGAAAAGGGGGGACTGGAAGGGCTAATTCACTCCCAACGAAGACAAGAT

ATCCTTGATCTGTGGATCTACCACACACAAGGCTACTTCCCTGATTGGCAGAA

CTACACACCAGGGCCAGGGATCAGATATCCACTGACCTTTGGATGGTGCTAC

AAGCTAGTACCAGTTGAGCAAGAGAAGGTAGAAGAAGCCAATGAAGGAGAG

AACACCCGCTTGTTACACCCTGTGAGCCTGCATGGGATGGATGACCCGGAGA

GAGAAGTATTAGAGTGGAGGTTTGACAGCCGCCTAGCATTTCATCACATGGC

CCGAGAGCTGCATCCGGACTGTACTGGGTCTCTCTGGTTAGACCAGATCTGA

GCCTGGGAGCTCTCTGGCTAACTAGGGAACCCACTGCTTAAGCCTCAATAAA

GCTTGCCTTGAGTGCTTCAAGTAGTGTGTGCCCGTCTGTTGTGTGACTCTGGT

AACTAGAGATCCCTCAGACCCTTTTAGTCAGTGTGGAAAATCTCTAGCAGGG

CCCGTTTAAACCCGCTGATCAGCCTCGACTGTGCCTTCTAGTTGCCAGCCATC

TGTTGTTTGCCCCTCCCCCGTGCCTTCCTTGACCCTGGAAGGTGCCACTCCCA

CTGTCCTTTCCTAATAAAATGAGGAAATTGCATCGCATTGTCTGAGTAGGTGT

CATTCTATTCTGGGGGGTGGGGTGGGGCAGGACAGCAAGGGGGAGGATTGG

GAAGACAATAGCAGGCATGCTGGGGATGCGGTGGGCTCTATGGCTTCTGAGG

CGGAAAGAACCAGCTGGGGCTCTAGGGGGTATCCCCACGCGCCCTGTAGCGG

CGCATTAAGCGCGGCGGGTGTGGTGGTTACGCGCAGCGTGACCGCTACACTT

GCCAGCGCCCTAGCGCCCGCTCCTTTCGCTTTCTTCCCTTCCTTTCTCGCCACG

TTCGCCGGCTTTCCCCGTCAAGCTCTAAATCGGGGGCTCCCTTTAGGGTTCCG

ATTTAGTGCTTTACGGCACCTCGACCCCAAAAAACTTGATTAGGGTGATGGTT

CACGTAGTGGGCCATCGCCCTGATAGACGGTTTTTCGCCCTTTGACGTTGGAG

TCCACGTTCTTTAATAGTGGACTCTTGTTCCAAACTGGAACAACACTCAACCC

TATCTCGGTCTATTCTTTTGATTTATAAGGGATTTTGCCGATTTCGGCCTATTG

GTTAAAAAATGAGCTGATTTAACAAAAATTTAACGCGAATTAATTCTGTGGA

ATGTGTGTCAGTTAGGGTGTGGAAAGTCCCCAGGCTCCCCAGCAGGCAGAAG

TATGCAAAGCATGCATCTCAATTAGTCAGCAACCAGGTGTGGAAAGTCCCCA

GGCTCCCCAGCAGGCAGAAGTATGCAAAGCATGCATCTCAATTAGTCAGCAA

CCATAGTCCCGCCCCTAACTCCGCCCATCCCGCCCCTAACTCCGCCCAGTTCC

GCCCATTCTCCGCCCCATGGCTGACTAATTTTTTTTATTTATGCAGAGGCCGA

GGCCGCCTCTGCCTCTGAGCTATTCCAGAAGTAGTGAGGAGGCTTTTTTGGAG

GCCTAGGCTTTTGCAAAAAGCTCCCGGGAGCTTGTATATCCATTTTCGGATCT

GATCAGCACGTGTTGACAATTAATCATCGGCATAGTATATCGGCATAGTATA

ATACGACAAGGTGAGGAACTAAACCATGGCCAAGTTGACCAGTGCCGTTCCG

GTGCTCACCGCGCGCGACGTCGCCGGAGCGGTCGAGTTCTGGACCGACCGGC

TCGGGTTCTCCCGGGACTTCGTGGAGGACGACTTCGCCGGTGTGGTCCGGGA

CGACGTGACCCTGTTCATCAGCGCGGTCCAGGACCAGGTGGTGCCGGACAAC

ACCCTGGCCTGGGTGTGGGTGCGCGGCCTGGACGAGCTGTACGCCGAGTGGT

CGGAGGTCGTGTCCACGAACTTCCGGGACGCCTCCGGGCCGGCCATGACCGA

GATCGGCGAGCAGCCGTGGGGGCGGGAGTTCGCCCTGCGCGACCCGGCCGG

CAACTGCGTGCACTTCGTGGCCGAGGAGCAGGACTGACACGTGCTACGAGAT

TTCGATTCCACCGCCGCCTTCTATGAAAGGTTGGGCTTCGGAATCGTTTTCCG

GGACGCCGGCTGGATGATCCTCCAGCGCGGGGATCTCATGCTGGAGTTCTTC

GCCCACCCCAACTTGTTTATTGCAGCTTATAATGGTTACAAATAAAGCAATAG

CATCACAAATTTCACAAATAAAGCATTTTTTTCACTGCATTCTAGTTGTGGTTT

GTCCAAACTCATCAATGTATCTTATCATGTCTGTATACCGTCGACCTCTAGCT

AGAGCTTGGCGTAATCATGGTCATAGCTGTTTCCTGTGTGAAATTGTTATCCG

CTCACAATTCCACACAACATACGAGCCGGAAGCATAAAGTGTAAAGCCTGGG

GTGCCTAATGAGTGAGCTAACTCACATTAATTGCGTTGCGCTCACTGCCCGCT

TTCCAGTCGGGAAACCTGTCGTGCCAGCTGCATTAATGAATCGGCCAACGCG

CGGGGAGAGGCGGTTTGCGTATTGGGCGCTCTTCCGCTTCCTCGCTCACTGAC

TCGCTGCGCTCGGTCGTTCGGCTGCGGCGAGCGGTATCAGCTCACTCAAAGG

CGGTAATACGGTTATCCACAGAATCAGGGGATAACGCAGGAAAGAACATGT

GAGCAAAAGGCCAGCAAAAGGCCAGGAACCGTAAAAAGGCCGCGTTGCTGG

CGTTTTTCCATAGGCTCCGCCCCCCTGACGAGCATCACAAAAATCGACGCTCA

AGTCAGAGGTGGCGAAACCCGACAGGACTATAAAGATACCAGGCGTTTCCCC

CTGGAAGCTCCCTCGTGCGCTCTCCTGTTCCGACCCTGCCGCTTACCGGATAC

CTGTCCGCCTTTCTCCCTTCGGGAAGCGTGGCGCTTTCTCATAGCTCACGCTG

TAGGTATCTCAGTTCGGTGTAGGTCGTTCGCTCCAAGCTGGGCTGTGTGCACG

AACCCCCCGTTCAGCCCGACCGCTGCGCCTTATCCGGTAACTATCGTCTTGAG

TCCAACCCGGTAAGACACGACTTATCGCCACTGGCAGCAGCCACTGGTAACA

GGATTAGCAGAGCGAGGTATGTAGGCGGTGCTACAGAGTTCTTGAAGTGGTG

GCCTAACTACGGCTACACTAGAAGAACAGTATTTGGTATCTGCGCTCTGCTGA

AGCCAGTTACCTTCGGAAAAAGAGTTGGTAGCTCTTGATCCGGCAAACAAAC

CACCGCTGGTAGCGGTGGTTTTTTTGTTTGCAAGCAGCAGATTACGCGCAGAA

AAAAAGGATCTCAAGAAGATCCTTTGATCTTTTCTACGGGGTCTGACGCTCAG

TGGAACGAAAACTCACGTTAAGGGATTTTGGTCATGAGATTATCAAAAAGGA

TCTTCACCTAGATCCTTTTAAATTAAAAATGAAGTTTTAAATCAATCTAAAGT

ATATATGAGTAAACTTGGTCTGACAGTTACCAATGCTTAATCAGTGAGGCAC

CTATCTCAGCGATCTGTCTATTTCGTTCATCCATAGTTGCCTGACTCCCCGTCG

TGTAGATAACTACGATACGGGAGGGCTTACCATCTGGCCCCAGTGCTGCAAT

GATACCGCGAGACCCACGCTCACCGGCTCCAGATTTATCAGCAATAAACCAG

CCAGCCGGAAGGGCCGAGCGCAGAAGTGGTCCTGCAACTTTATCCGCCTCCA

TCCAGTCTATTAATTGTTGCCGGGAAGCTAGAGTAAGTAGTTCGCCAGTTAAT

AGTTTGCGCAACGTTGTTGCCATTGCTACAGGCATCGTGGTGTCACGCTCGTC

GTTTGGTATGGCTTCATTCAGCTCCGGTTCCCAACGATCAAGGCGAGTTACAT

GATCCCCCATGTTGTGCAAAAAAGCGGTTAGCTCCTTCGGTCCTCCGATCGTT

GTCAGAAGTAAGTTGGCCGCAGTGTTATCACTCATGGTTATGGCAGCACTGC

ATAATTCTCTTACTGTCATGCCATCCGTAAGATGCTTTTCTGTGACTGGTGAG

TACTCAACCAAGTCATTCTGAGAATAGTGTATGCGGCGACCGAGTTGCTCTTG

CCCGGCGTCAATACGGGATAATACCGCGCCACATAGCAGAACTTTAAAAGTG

CTCATCATTGGAAAACGTTCTTCGGGGCGAAAACTCTCAAGGATCTTACCGCT

GTTGAGATCCAGTTCGATGTAACCCACTCGTGCACCCAACTGATCTTCAGCAT

CTTTTACTTTCACCAGCGTTTCTGGGTGAGCAAAAACAGGAAGGCAAAATGC

CGCAAAAAAGGGAATAAGGGCGACACGGAAATGTTGAATACTCATACTCTTC

CTTTTTCAATATTATTGAAGCATTTATCAGGGTTATTGTCTCATGAGCGGATA

CATATTTGAATGTATTTAGAAAAATAAACAAATAGGGGTTCCGCGCACATTT

CCCCGAAAAGTGCCACCTGAC

Plasmids used in construction of pSLIK3

>pFN24K::Crimson/P2A-Control

AKA pFN24K::Product N-Control.

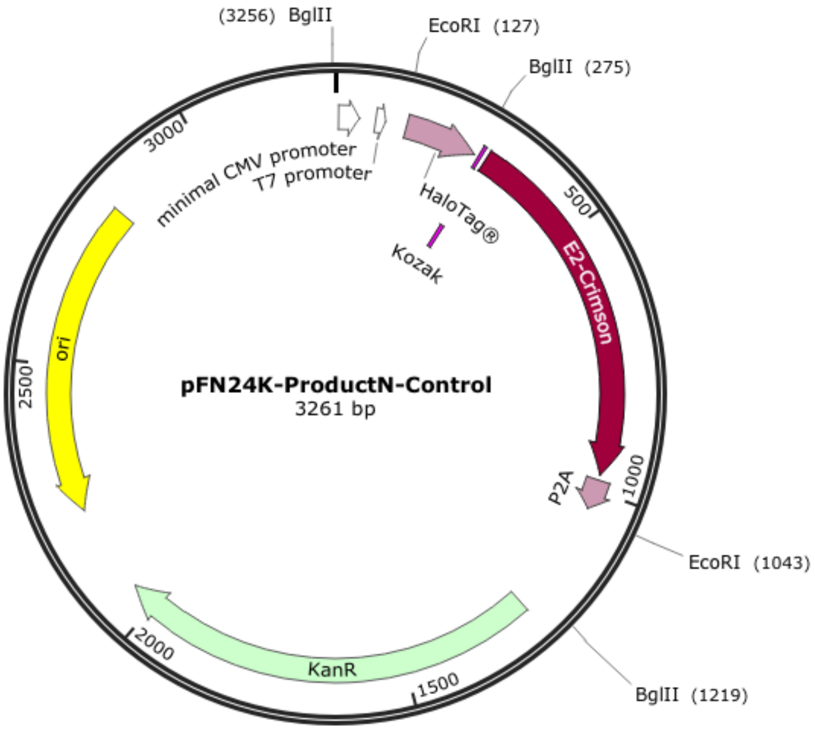

>pFN24K::Crimson/P2A-Control

ATGGGCGGTAGGCGTGTACGGTGGGAGGTCTATATAAGCAGAGCTGGTTTAG

TGAACCGTCAGATCACTAGAAGCTTAATACGACTCACTATAGGGTACGATTT

AGGTGACACTATAGAATAAGGAGAATTCATCATGGCAGAAATCGGTACTGGC

TTTCCATTCGACCCCCATTATGTGGAAGTCCTGGGCGAGCGCATGCACTACGT

CGATGTTGGTCCGCGCGATGGCACCCCTGTGCTGTTCCTGCACGGTAACCCGA

CCTCCTCCTACCAGATCTAGGAGGTCCGCCACCATGGATAGCACTGAGAACG

TCATCAAGCCCTTCATGCGCTTCAAGGTGCACATGGAGGGCTCCGTGAACGG

CCACGAGTTCGAGATCGAGGGCGTGGGCGAGGGCAAGCCCTACGAGGGCAC
CCAGACCGCCAAGCTGCAAGTGACCAAGGGCGGCCCCCTGCCCTTCGCCTGG
GACATCCTGTCCCCCCAGTTCTTCTACGGCTCCAAGGCGTACATCAAGCACCC
CGCCGACATCCCCGACTACCTCAAGCAGTCCTTCCCCGAGGGCTTCAAGTGG
GAGCGCGTGATGAACTTCGAGGACGGCGGCGTGGTGACCGTGACCCAGGACT
CCTCCCTGCAGGACGGCACCCTCATCTACCACGTGAAGTTCATCGGCGTGAA
CTTCCCCTCCGACGGCCCCGTAATGCAGAAGAAGACTCTGGGCTGGGAGCCC
TCCACTGAGCGCAACTACCCCCGCGACGGCGTGCTGAAGGGCGAGAACCACA
TGGCGCTGAAGCTGAAGGGCGGCGGCCACTACCTGTGTGAGTTCAAGTCCAT
CTACATGGCCAAGAAGCCCGTGAAGCTGCCCGGCTACCACTACGTGGACTAC
AAGCTCGACATCACCTCCCACAACGAGGACTACACCGTGGTGGAGCAGTACG
AGCGCGCCGAGGCCCGCCACCACCTGTTCCAGGGATCCGGAGCCACGAACTT
CTCTCTGTTAAAGCAAGCAGGAGACGTGGAAGAAAACCCCGGTCCTCACTGA
GAATTCGCGGCCGCAGTGTTCCGCTTCCTTTAGCAGCCCTTGCGCCCTGAGTG
CTTGCGGCAGCGTGAGCTTCAAAAGAATTGCCAGCTGGGGCGCCCTCTGGTA
AGGTTGGGAAGCCCTGCAAAGTAAACTGGATGGCTTTCTTGCCGCCAAGGAT
CTGATGGCGCAGGGGATCAAGATCTGATCAAGAGACAGGATGACGGTCGTTT
CGCATGCTTGAACAAGATGGATTGCACGCAGGTTCTCCGGCCGCTTGGGTGG
AGAGGCTATTCGGCTATGACTGGGCACAACAGACAATCGGCTGCTCTGATGC
CGCCGTGTTCCGGCTGTCAGCGCAGGGGCGCCCGGTTCTTTTTGTCAAGACCG
ACCTGTCCGGTGCCCTGAATGAACTGCAGGACGAGGCAGCGCGGCTATCGTG
GCTGGCCACGACGGGCGTTCCTTGCGCAGCTGTGCTCGACGTTGTCACTGAA
GCGGGAAGGGACTGGCTGCTATTGGGCGAAGTGCCGGGGCAGGATCTCCTGT

CATCTCACCTTGCTCCTGCCGAGAAAGTATCCATCATGGCTGATGCAATGCGG

CGGCTGCATACGCTTGATCCGGCTACCTGCCCATTCGACCACCAAGCGAAAC

ATCGCATCGAGCGAGCACGCACTCGGATGGAAGCCGGTCTTGTCGATCAGGA

TGATCTGGACGAAGAGCATCAGGGGCTCGCGCCAGCCGAACTGTTCGCCAGG

CTCAAGGCGCGTATGCCCGGATGGTGAGGATCTCGTCGTGACTCATGGCGATG

CCTGCTTGCCGAATATCATGGTGGAAAATGGCCGCTTTTCTGGATTCATCGAC

TGTGGCCGGCTGGGTGTGGCGGACCGCTATCAGGACATAGCGTTGGCTACCC

GTGATATTGCTGAAGAGCTTGGCGGCGAATGGGCTGACCGCTTCCTCGTGCTT

TACGGTATCGCCGCTCCCGATTCGCAGCGCATCGCCTTCTATCGCCTTCTTGA

CGAGTTCTTCTGAGCGGGACTCTGGGGTTCGAAATGACCGACCAAGCGACGC

CCAACCGGTATCAGCTCACTCAAAGGCGGTAATACGGTTATCCACAGAATCA

GGGGATAACGCAGGAAAGAACATGTGAGCAAAAGGCCAGCAAAAGGCCAGG

AACCGTAAAAAGGCCGCGTTGCTGGCGTTTTTCCATAGGCTCCGCCCCCCTGA

CGAGCATCACAAAAATCGACGCTCAAGTCAGAGGTGGCGAAACCCGACAGG

ACTATAAAGATACCAGGCGTTTCCCCCTGGAAGCTCCCTCGTGCGCTCTCCTG

TTCCGACCCTGCCGCTTACCGGATACCTGTCCGCCTTTCTCCCTTCGGGAAGC

GTGGCGCTTTCTCATAGCTCACGCTGTAGGTATCTCAGTTCGGTGTAGGTCGT

TCGCTCCAAGCTGGGCTGTGTGCACGAACCCCCCGTTCAGCCCGACCGCTGC

GCCTTATCCGGTAACTATCGTCTTGAGTCCAACCCGGTAAGACACGACTTATC

GCCACTGGCAGCAGCCACTGGTAACAGGATTAGCAGAGCGAGGTATGTAGGC

GGTGCTACAGAGTTCTTGAAGTGGTGGCCTAACTACGGCTACACTAGAAGGA

CAGTATTTGGTATCTGCGCTCTGCTGAAGCCAGTTACCTTCGGAAAAAGAGTT

GGTAGCTCTTGATCCGGCAAACAAACCACCGCTGGTAGCGGTGGTTTTTTTGT

TTGCAAGCAGCAGATTACGCGCAGAAAAAAAGGATTTCAAGAAGATCCTTTG
ATCTTTTCTACGGGGTCTGACGCTCAGTGGAACGAAAACTCACGTTAAGGGA
TTTTGGTCATGAGATTATCAAAAAGGATCTTCACCTAGATCCTTTTATAGTCC
GGAAATACAGGAACGCACGCTGGATGGCCCTTCGCTGGGATGGTGAAACCAT
GAAAAATGGCAGCTTCAGTGGATTAAGTGGGGGTAATGTGGCCTGTACCCTC
TGGTTGCATAGGTATTCATACGGTTAAAATTTATCAGGCGCGATTGCGGCAGT
TTTTCGGGTGGTTTGTTGCCATTTTTACCTGTCTGCTGCCGTGATCGCGCTGAA
CGCGTTTTAGCGGTGCGTACAATTAAGGGATTATGGTAAATCCACTTACTGTC
TGCCCTCGTAGCCATCGAGATAAACCGCAGTACTCCGGCCACGATGCGTCCG
GCGTAGAGGATCGAGATCT

>pENTR2B/TREPitt::Crimson/P2A-Control

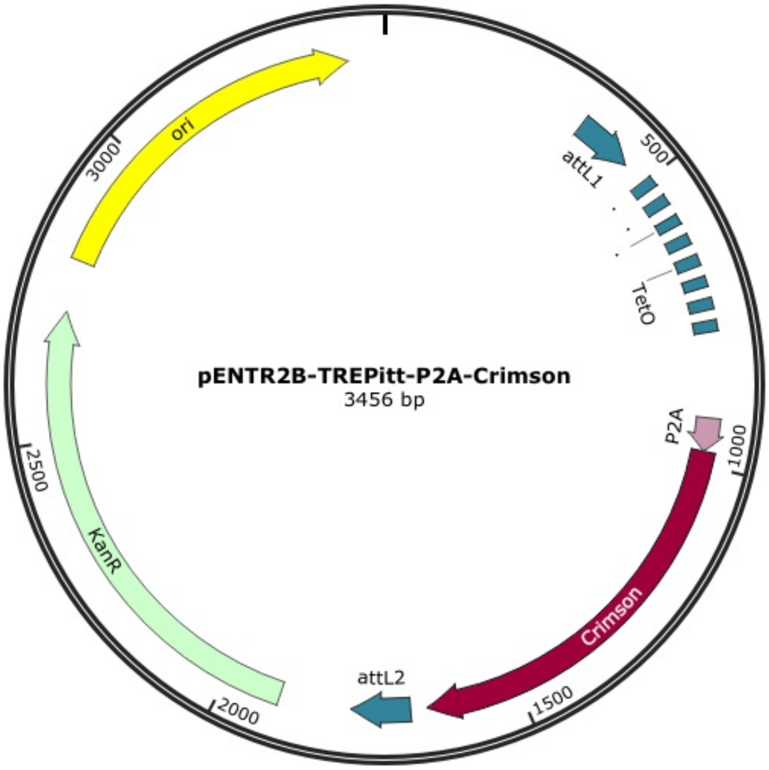

>pENTR2B/TREPitt::Crimson/P2A-Control

CTTTCCTGCGTTATCCCCTGATTCTGTGGATAACCGTATTACCGCTAGCATGG

ATCTCGGGGACGTCTAACTACTAAGCGAGAGTAGGGAACTGCCAGGCATCAA

ATAAAACGAAAGGCTCAGTCGGAAGACTGGGCCTTTCGTTTTATCTGTTGTTT

GTCGGTGAACGCTCTCCTGAGTAGGACAAATCCGCCGGGAGCGGATTTGAAC

GTTGTGAAGCAACGGCCCGGAGGGTGGCGGGCAGGACGCCCGCCATAAACT

GCCAGGCATCAAACTAAGCAGAAGGCCATCCTGACGGATGGCCTTTTTGCGT

TTCTACAAACTCTTCCTGTTAGTTAGTTACTTAAGCTCGGGCCCCAAATAATG

ATTTTATTTTGACTGATAGTGACCTGTTCGTTGCAACAAATTGATAAGCAATG

CTTTTTTATAATGCCAACTTTGTACAAAAAAGCAGGCTGGCGCCGGAACCAA

TTCAgatatcAAGTGCCACCTGACGTCTCCCTATCAGTGATAGAGAAGTCGACAC

GTCTCGAGCTCCCTATCAGTGATAGAGAAGGTACGTCTAGAACGTCTCCCTAT

CAGTGATAGAGAAGTCGACACGTCTCGAGCTCCCTATCAGTGATAGAGAAGG

TACGTCTAGAACGTCTCCCTATCAGTGATAGAGAAGTCGACACGTCTCGAGC

TCCCTATCAGTGATAGAGAAGGTACGTCTAGAACGTCTCCCTATCAGTGATA

GAGAAGTCGACACGTCTCGAGCTCCCTATCAGTGATAGAGAAGGTACCCCCT

ATATAAGCAGAGCTCGTTTAGTGAACCGTCAGATCGCCTGGAGACGCCATCC

ACGCTGTTTTGACCTCCATAGAAGACACCGGGACCGATCCAGCCTGGATCTA

GGAGGTCCGCCACCATGGATAGCACTGAGAACGTCATCAAGCCCTTCATGCG

CTTCAAGGTGCACATGGAGGGCTCCGTGAACGGCCACGAGTTCGAGATCGAG

GGCGTGGGCGAGGGCAAGCCCTACGAGGGCACCCAGACCGCCAAGCTGCAA

GTGACCAAGGGCGGCCCCCTGCCCTTCGCCTGGGACATCCTGTCCCCCCAGTT

CTTCTACGGCTCCAAGGCGTACATCAAGCACCCCGCCGACATCCCCGACTAC

CTCAAGCAGTCCTTCCCCGAGGGCTTCAAGTGGGAGCGCGTGATGAACTTCG
AGGACGGCGGCGTGGTGACCGTGACCCAGGACTCCTCCCTGCAGGACGGCAC
CCTCATCTACCACGTGAAGTTCATCGGCGTGAACTTCCCCTCCGACGGCCCCG
TAATGCAGAAGAAGACTCTGGGCTGGGAGCCCTCCACTGAGCGCAACTACCC
CCGCGACGGCGTGCTGAAGGGCGAGAACCACATGGCGCTGAAGCTGAAGGG
CGGCGGCCACTACCTGTGTGAGTTCAAGTCCATCTACATGGCCAAGAAGCCC
GTGAAGCTGCCCGGCTACCACTACGTGGACTACAAGCTCGACATCACCTCCC
ACAACGAGGACTACACCGTGGTGGAGCAGTACGAGCGCGCCGAGGCCCGCC
ACCACCTGTTCCAGGGATCCGGAGCCACGAACTTCTCTCTGTTAAAGCAAGC
AGGAGACGTGGAAGAAAACCCCGGTCCTcACtgaGAATTCGCGGCCGCACTCG
AGATATCTAGACCCAGCTTTCTTGTACAAAGTTGGCATTATAAGAAAGCATTG
CTTATCAATTTGTTGCAACGAACAGGTCACTATCAGTCAAAATAAAATCATTA
TTTGCCATCCAGCTGCAGCTCTGGCCCGTGTCTCAAAATCTCTGATGTTACAT
TGCACAAGATAAAAATATATCATCATGAACAATAAAACTGTCTGCTTACATA
AACAGTAATACAAGGGGTGTTATGAGCCATATTCAACGGGAAACGTCGAGGC
CGCGATTAAATTCCAACATGGATGCTGATTTATATGGGTATAAATGGGCTCGC
GATAATGTCGGGCAATCAGGTGCGACAATCTATCGCTTGTATGGGAAGCCCG
ATGCGCCAGAGTTGTTTCTGAAACATGGCAAAGGTAGCGTTGCCAATGATGT
TACAGATGAGATGGTCAGACTAAACTGGCTGACGGAATTTATGCCTCTTCCG
ACCATCAAGCATTTTATCCGTACTCCTGATGATGCATGGTTACTCACCACTGC
GATCCCCGGAAAAACAGCATTCCAGGTATTAGAAGAATATCCTGATTCAGGT
GAAAATATTGTTGATGCGCTGGCAGTGTTCCTGCGCCGGTTGCATTCGATTCC
TGTTTGTAATTGTCCTTTTAACAGCGATCGCGTATTTCGTCTCGCTCAGGCGC

AATCACGAATGAATAACGGTTTGGTTGATGCGAGTGATTTTGATGACGAGCG

TAATGGCTGGCCTGTTGAACAAGTCTGGAAAGAAATGCATAAACTTTTGCCA

TTCTCACCGGATTCAGTCGTCACTCATGGTGATTTCTCACTTGATAACCTTATT

TTTGACGAGGGGAAATTAATAGGTTGTATTGATGTTGGACGAGTCGGAATCG

CAGACCGATACCAGGATCTTGCCATCCTATGGAACTGCCTCGGTGAGTTTTCT

CCTTCATTACAGAAACGGCTTTTTCAAAAATATGGTATTGATAATCCTGATAT

GAATAAATTGCAGTTTCATTTGATGCTCGATGAGTTTTTCTAATCAGAATTGG

TTAATTGGTTGTAACATTATTCAGATTGGGCCCCGTTCCACTGAGCGTCAGAC

CCCGTAGAAAAGATCAAAGGATCTTCTTGAGATCCTTTTTTTCTGCGCGTAAT

CTGCTGCTTGCAAACAAAAAAACCACCGCTACCAGCGGTGGTTTGTTTGCCG

GATCAAGAGCTACCAACTCTTTTTCCGAAGGTAACTGGCTTCAGCAGAGCGC

AGATACCAAATACTGTTCTTCTAGTGTAGCCGTAGTTAGGCCACCACTTCAAG

AACTCTGTAGCACCGCCTACATACCTCGCTCTGCTAATCCTGTTACCAGTGGC

TGCTGCCAGTGGCGATAAGTCGTGTCTTACCGGGTTGGACTCAAGACGATAG

TTACCGGATAAGGCGCAGCGGTCGGGCTGAACGGGGGGTTCGTGCACACAGC

CCAGCTTGGAGCGAACGACCTACACCGAACTGAGATACCTACAGCGTGAGCT

ATGAGAAAGCGCCACGCTTCCCGAAGGGAGAAAGGCGGACAGGTATCCGGT

AAGCGGCAGGGTCGGAACAGGAGAGCGCACGAGGGAGCTTCCAGGGGGAAA

CGCCTGGTATCTTTATAGTCCTGTCGGGTTTCGCCACCTCTGACTTGAGCGTC

GATTTTTGTGATGCTCGTCAGGGGGGCGGAGCCTATGGAAAAACGCCAGCAA

CGCGGCCTTTTTACGGTTCCTGGCCTTTTGCTGGCCTTTTGCTCACATGTT

>pSLIK/TREPitt::Crimson/P2A

Sequence verified from 2770 to 3963 bp.

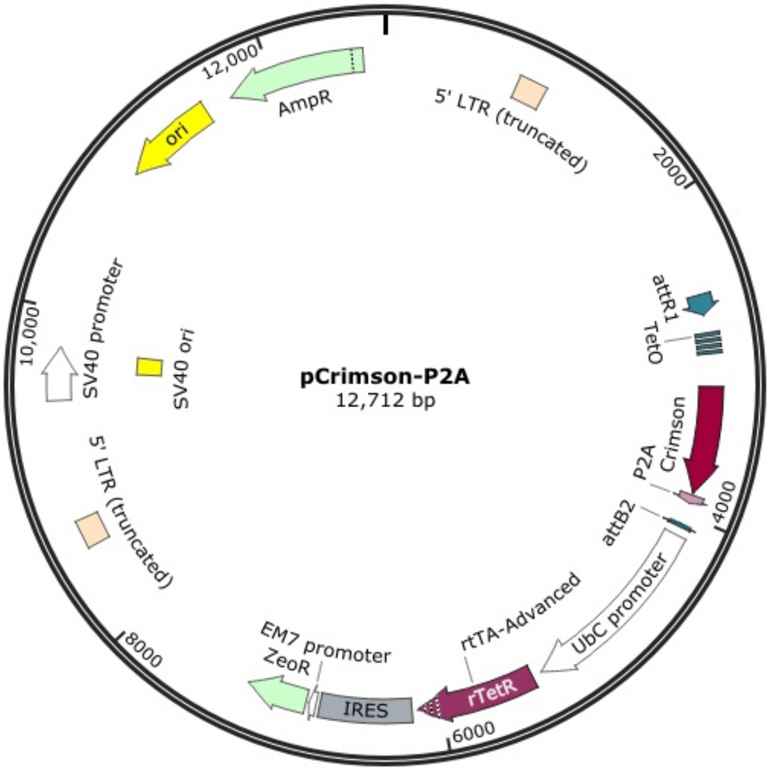

>3.pSLIK-Zeo/TREPitt::Crimson/P2A-Control

GTCGACGGATCGGGAGATCTCCCGATCCCCTATGGTGCACTCTCAGTACAAT

CTGCTCTGATGCCGCATAGTTAAGCCAGTATCTGCTCCCTGCTTGTGTGTTGG

AGGTCGCTGAGTAGTGCGCGAGCAAAATTTAAGCTACAACAAGGCAAGGCTT

GACCGACAATTGCATGAAGAATCTGCTTAGGGTTAGGCGTTTTGCGCTGCTTC

GCGATGTACGGGCCAGATATACGCGTTGACATTGATTATTGACTAGTTATTAA

TAGTAATCAATTACGGGGTCATTAGTTCATAGCCCATATATGGAGTTCCGCGT

TACATAACTTACGGTAAATGGCCCGCCTGGCTGACCGCCCAACGACCCCCGC

CCATTGACGTCAATAATGACGTATGTTCCCATAGTAACGCCAATAGGGACTTT

CCATTGACGTCAATGGGTGGAGTATTTACGGTAAACTGCCCACTTGGCAGTA

CATCAAGTGTATCATATGCCAAGTACGCCCCCTATTGACGTCAATGACGGTA
AATGGCCCGCCTGGCATTATGCCCAGTACATGACCTTATGGGACTTTCCTACT
TGGCAGTACATCTACGTATTAGTCATCGCTATTACCATGGTGATGCGGTTTTG
GCAGTACATCAATGGGCGTGGATAGCGGTTTGACTCACGGGGATTTCCAAGT
CTCCACCCCATTGACGTCAATGGGAGTTTGTTTTGGCACCAAAATCAACGGG
ACTTTCCAAAATGTCGTAACAACTCCGCCCCATTGACGCAAATGGGCGGTAG
GCGTGTACGGTGGGAGGTCTATATAAGCAGCGCGTTTTGCCTGTACTGGGTCT
CTCTGGTTAGACCAGATCTGAGCCTGGGAGCTCTCTGGCTAACTAGGGAACC
CACTGCTTAAGCCTCAATAAAGCTTGCCTTGAGTGCTTCAAGTAGTGTGTGCC
CGTCTGTTGTGTGACTCTGGTAACTAGAGATCCCTCAGACCCTTTTAGTCAGT
GTGGAAAATCTCTAGCAGTGGCGCCCGAACAGGGACTTGAAAGCGAAAGGG
AAACCAGAGGAGCTCTCTCGACGCAGGACTCGGCTTGCTGAAGCGCGCACGG
CAAGAGGCGAGGGGCGGCGACTGGTGAGTACGCCAAAAATTTTGACTAGCG
GAGGCTAGAAGGAGAGAGATGGGTGCGAGAGCGTCAGTATTAAGCGGGGGA
GAATTAGATCGCGATGGGAAAAAATTCGGTTAAGGCCAGGGGGAAAGAAAA
AATATAAATTAAAACATATAGTATGGGCAAGCAGGGAGCTAGAACGATTCGC
AGTTAATCCTGGCCTGTTAGAAACATCAGAAGGCTGTAGACAAATACTGGGA
CAGCTACAACCATCCCTTCAGACAGGATCAGAAGAACTTAGATCATTATATA
ATACAGTAGCAACCCTCTATTGTGTGCATCAAAGGATAGAGATAAAAGACAC
CAAGGAAGCTTTAGACAAGATAGAGGAAGAGCAAAACAAAAGTAAGACCAC
CGCACAGCAAGCGGCCGCTGATCTTCAGACCTGGAGGAGGAGATATGAGGG
ACAATTGGAGAAGTGAATTATATAAATATAAAGTAGTAAAAATTGAACCATT
AGGAGTAGCACCCACCAAGGCAAAGAGAAGAGTGGTGCAGAGAGAAAAAA

GAGCAGTGGGAATAGGAGCTTTGTTCCTTGGGTTCTTGGGAGCAGCAGGAAG

CACTATGGGCGCAGCGTCAATGACGCTGACGGTACAGGCCAGACAATTATTG

TCTGGTATAGTGCAGCAGCAGAACAATTTGCTGAGGGCTATTGAGGCGCAAC

AGCATCTGTTGCAACTCACAGTCTGGGGCATCAAGCAGCTCCAGGCAAGAAT

CCTGGCTGTGGAAAGATACCTAAAGGATCAACAGCTCCTGGGGATTTGGGGT

TGCTCTGGAAAACTCATTTGCACCACTGCTGTGCCTTGGAATGCTAGTTGGAG

TAATAAATCTCTGGAACAGATTTGGAATCACACGACCTGGATGGAGTGGGAC

AGAGAAATTAACAATTACACAAGCTTAATACACTCCTTAATTGAAGAATCGC

AAAACCAGCAAGAAAAGAATGAACAAGAATTATTGGAATTAGATAAATGGG

CAAGTTTGTGGAATTGGTTTAACATAACAAATTGGCTGTGGTATATAAAATTA

TTCATAATGATAGTAGGAGGCTTGGTAGGTTTAAGAATAGTTTTTGCTGTACT

TTCTATAGTGAATAGAGTTAGGCAGGGATATTCACCATTATCGTTTCAGACCC

ACCTCCCAACCCCGAGGGGACCCGACAGGCCCGAAGGAATAGAAGAAGAAG

GTGGAGAGAGAGACAGAGACAGATCCATTCGATTAGTGAACGGATCGGCAC

TGCGTGCGCCAATTCTGCAGACAAATGGCAGTATTCATCCACAATTTTAAAA

GAAAAGGGGGGATTGGGGGGTACAGTGCAGGGGAAAGAATAGTAGACATAA

TAGCAACAGACATACAAACTAAAGAATTACAAAAACAAATTACAAAAATTC

AAAATTTTCGGGTTTATTACAGGGACAGCAGAGATCCAGTTTGGTTAATTCTA

GACAGTTATCAACAAGTTTGTACAAAAAAGCTGAACGAGAAACGTAAAATG

ATATAAATATCAATATATTAAATTAGATTTTGCATAAAAAACAGACTACATA

ATACTGTAAAACACAACATATCCAGTCACTATGGGCGCCGGAACCAATTCAga

tatcAAGTGCCACCTGACGTCTCCCTATCAGTGATAGAGAAGTCGACACGTCTC

GAGCTCCCTATCAGTGATAGAGAAGGTACGTCTAGAACGTCTCCCTATCAGT

GATAGAGAAGTCGACACGTCTCGAGCTCCCTATCAGTGATAGAGAAGGTACG

TCTAGAACGTCTCCCTATCAGTGATAGAGAAGTCGACACGTCTCGAGCTCCCT

ATCAGTGATAGAGAAGGTACGTCTAGAACGTCTCCCTATCAGTGATAGAGAA

GTCGACACGTCTCGAGCTCCCTATCAGTGATAGAGAAGGTACCCCCTATATA

AGCAGAGCTCGTTTAGTGAACCGTCAGATCGCCTGGAGACGCCATCCACGCT

GTTTTGACCTCCATAGAAGACACCGGGACCGATCCAGCCTGGATCTAGGAGG

TCCGCCACCATGGATAGCACTGAGAACGTCATCAAGCCCTTCATGCGCTTCA

AGGTGCACATGGAGGGCTCCGTGAACGGCCACGAGTTCGAGATCGAGGGCGT

GGGCGAGGGCAAGCCCTACGAGGGCACCCAGACCGCCAAGCTGCAAGTGAC

CAAGGGCGGCCCCCTGCCCTTCGCCTGGGACATCCTGTCCCCCCAGTTCTTCT

ACGGCTCCAAGGCGTACATCAAGCACCCCGCCGACATCCCCGACTACCTCAA

GCAGTCCTTCCCCGAGGGCTTCAAGTGGGAGCGCGTGATGAACTTCGAGGAC

GGCGGCGTGGTGACCGTGACCCAGGACTCCTCCCTGCAGGACGGCACCCTCA

TCTACCACGTGAAGTTCATCGGCGTGAACTTCCCCTCCGACGGCCCCGTAATG

CAGAAGAAGACTCTGGGCTGGGAGCCCTCCACTGAGCGCAACTACCCCCGCG

ACGGCGTGCTGAAGGGCGAGAACCACATGGCGCTGAAGCTGAAGGGCGGCG

GCCACTACCTGTGTGAGTTCAAGTCCATCTACATGGCCAAGAAGCCCGTGAA

GCTGCCCGGCTACCACTACGTGGACTACAAGCTCGACATCACCTCCCACAAC

GAGGACTACACCGTGGTGGAGCAGTACGAGCGCGCCGAGGCCCGCCACCAC

CTGTTCCAGGGATCCGGAGCCACGAACTTCTCTCTGTTAAAGCAAGCAGGAG

ACGTGGAAGAAAACCCCGGTCCTcACtgaGAATTCGCGGCCGCACTCGAGATA

TCTAGATAGTGACTGGATATGTTGTGTTTTACAGTATTATGTAGTCTGTTTTTT

ATGCAAAATCTAATTTAATATATTGATATTTATATCATTTTACGTTTCTCGTTC

AGCTTTCTTGTACAAAGTGGTTGATAACATCTCGAGCCATAAAGATGGTTAAT

TAACCCACCCAAGATCTGGCCTCCGCGCCGGGTTTTGGCGCCTCCCGCGGGC

GCCCCCCTCCTCACGGCGAGCGCTGCCACGTCAGACGAAGGGCGCAGCGAGC

GTCCTGATCCTTCCGCCCGGACGCTCAGGACAGCGGCCCGCTGCTCATAAGA

CTCGGCCTTAGAACCCCAGTATCAGCAGAAGGACATTTTAGGACGGGACTTG

GGTGACTCTAGGGCACTGGTTTTCTTTCCAGAGAGCGGAACAGGCGAGGAAA

AGTAGTCCCTTCTCGGCGATTCTGCGGAGGGATCTCCGTGGGGCGGTGAACG

CCGATGATTATATAAGGACGCGCCGGGTGTGGCACAGCTAGTTCCGTCGCAG

CCGGGATTTGGGTCGCGGTTCTTGTTTGTGGATCGCTGTGATCGTCACTTGGT

GAGTAGCGGGCTGCTGGGCTGGCCGGGGCTTTCGTGGCCGCCGGGCCGCTCG

GTGGGACGGAAGCGTGTGGAGAGACCGCCAAGGGCTGTAGTCTGGGTCCGC

GAGCAAGGTTGCCCTGAACTGGGGGTTGGGGGGAGCGCAGCAAAATGGCGG

CTGTTCCCGAGTCTTGAATGGAAGACGCTTGTGAGGCGGGCTGTGAGGTCGT

TGAAACAAGGTGGGGGGCATGGTGGGCGGCAAGAACCCAAGGTCTTGAGGC

CTTCGCTAATGCGGGAAAGCTCTTATTCGGGTGAGATGGGCTGGGGCACCAT

CTGGGGACCCTGACGTGAAGTTTGTCACTGACTGGAGAACTCGGTTTGTCGTC

TGTTGCGGGGGCGGCAGTTATGGCGGTGCCGTTGGGCAGTGCACCCGTACCT

TTGGGAGCGCGCGCCCTCGTCGTGTCGTGACGTCACCCGTTCTGTTGGCTTAT

AATGCAGGGTGGGGCCACCTGCCGGTAGGTGTGCGGTAGGCTTTTCTCCGTC

GCAGGACGCAGGGTTCGGGCCTAGGGTAGGCTCTCCTGAATCGACAGGCGCC

GGACCTCTGGTGAGGGGAGGGATAAGTGAGGCGTCAGTTTCTTTGGTCGGTT

TTATGTACCTATCTTCTTAAGTAGCTGAAGCTCCGGTTTTGAACTATGCGCTC

GGGGTTGGCGAGTGTGTTTTGTGAAGTTTTTTAGGCACCTTTTGAAATGTAAT

CATTTGGGTCAATATGTAATTTTCAGTGTTAGACTAGTAAATTGTCCGCTAAA

TTCTGGCCGTTTTTGGCTTTTTTGTTAGACGAAGCTTGGTACCGAGCTCGGAT

CTCCACCCCGTACCGGTCCTGCAGTCGAATTCACCATGTCTAGACTGGACAA

GAGCAAAGTCATAAACGGAGCTCTGGAATTACTCAATGGTGTCGGTATCGAA

GGCCTGACGACAAGGAAACTCGCTCAAAAGCTGGGAGTTGAGCAGCCTACCC

TGTACTGGCACGTGAAGAACAAGCGGGCCCTGCTCGATGCCCTGCCAATCGA

GATGCTGGACAGGCATCATACCCACTTCTGCCCCCTGGAAGGCGAGTCATGG

CAAGACTTTCTGCGGAACAACGCCAAGTCATACCGCTGTGCTCTCCTCTCACA

TCGCGACGGGGCTAAAGTGCATCTCGGCACCCGCCCAACAGAGAAACAGTAC

GAAACCCTGGAAAATCAGCTCGCGTTCCTGTGTCAGCAAGGCTTCTCCCTGG

AGAACGCACTGTACGCTCTGTCCGCCGTGGGCCACTTTACACTGGGCTGCGT

ATTGGAGGAACAGGAGCATCAAGTAGCAAAAGAGGAAAGAGAGACACCTAC

CACCGATTCTATGCCCCCACTTCTGAGACAAGCAATTGAGCTGTTCGACCGGC

AGGGAGCCGAACCTGCCTTCCTTTTCGGCCTGGAACTAATCATATGTGGCCTG

GAGAAACAGCTAAAGTGCGAAAGCGGCGGGCCGACCGACGCCCTTGACGAT

TTTGACTTAGACATGCTCCCAGCCGATGCCCTTGACGACTTTGACCTTGATAT

GCTGCCTGCTGACGCTCTTGACGATTTTGACCTTGACATGCTCCCCGGGTAAC

TAAGTAAGGATCCGCGGCCGCACTAGAGGAATTCCGCCCCTCTCCCTCCCCC

CCCCCTAACGTTACTGGCCGAAGCCGCTTGGAATAAGGCCGGTGTGTGTTTGT

CTATATGTTATTTTCCACCATATTGCCGTCTTTTGGCAATGTGAGGGCCCGGA

AACCTGGCCCTGTCTTCTTGACGAGCATTCCTAGGGGTCTTTCCCCTCTCGCC

AAAGGAATGCAAGGTCTGTTGAATGTCGTGAAGGAAGCAGTTCCTCTGGAAG

CTTCTTGAAGACAAACAACGTCTGTAGCGACCCTTTGCAGGCAGCGGAACCC

CCCACCTGGCGACAGGTGCCTCTGCGGCCAAAAGCCACGTGTATAAGATACA
CCTGCAAAGGCGGCACAACCCCAGTGCCACGTTGTGAGTTGGATAGTTGTGG
AAAGAGTCAAATGGCTCTCCTCAAGCGTAGTCAACAAGGGGCTGAAGGATGC
CCAGAAGGTACCCCATTGTATGGGAATCTGATCTGGGGCCTCGGTGCACATG
CTTTACATGTGTTTAGTCGAGGTTAAAAAAACGTCTAGGCCCCCCGAACCAC
GGGGACGTGGTTTTCCTTTGAAAAACACGATGATAAGCTTACCGGTACGCGT
GATGTGTTGACAATTAATCATCGGCATAGTATATCGGCATAGTATAATACGA
CAAGGTGAGGAACTAAACCATGGCCAAGTTGACCAGTGCCGTTCCGGTGCTC
ACCGCGCGCGACGTCGCCGGAGCGGTCGAGTTCTGGACCGACCGGCTCGGGT
TCTCCCGGGACTTCGTGGAGGACGACTTCGCCGGTGTGGTCCGGGACGACGT
GACCCTGTTCATCAGCGCGGTCCAGGACCAGGTGGTGCCGGACAACACCCTG
GCCTGGGTGTGGGTGCGCGGCCTGGACGAGCTGTACGCCGAGTGGTCGGAGG
TCGTGTCCACGAACTTCCGGGACGCCTCCGGGCCGGCCATGACCGAGATCGG
CGAGCAGCCGTGGGGGCGGGAGTTCGCCCTGCGCGACCCGGCCGGCAACTGC
GTGCACTTCGTGGCCGAGGAGCAGGACTGACACATCTGTACAAGTAAAGCGG
CCGCGACTCTAGATCATAATCAGCCATACCACATTTGTAGAGGTTTTACTTGC
TTTAAAAAACCTCCCACACCTCCCCCTGAACCTGAAACATAAAATGAATGCA
ATTGTTGTTGTTTAGTCCCTCCCAATTCGATATCAAGCTTATCGATAATCAAC
CTCTGGATTACAAAATTTGTGAAAGATTGACTGGTATTCTTAACTATGTTGCT
CCTTTTACGCTATGTGGATACGCTGCTTTAATGCCTTTGTATCATGCTATTGCT
TCCCGTATGGCTTTCATTTTCTCCTCCTTGTATAAATCCTGGTTGCTGTCTCTTT
ATGAGGAGTTGTGGCCCGTTGTCAGGCAACGTGGCGTGGTGTGCACTGTGTTT
GCTGACGCAACCCCCACTGGTTGGGGCATTGCCACCACCTGTCAGCTCCTTTC

CGGGACTTTCGCTTTCCCCCTCCCTATTGCCACGGCGGAACTCATCGCCGCCT

GCCTTGCCCGCTGCTGGACAGGGGCTCGGCTGTTGGGCACTGACAATTCCGT

GGTGTTGTCGGGGAAATCATCGTCCTTTCCTTGGCTGCTCGCCTGTGTTGCCA

CCTGGATTCTGCGCGGGACGTCCTTCTGCTACGTCCCTTCGGCCCTCAATCCA

GCGGACCTTCCTTCCCGCGGCCTGCTGCCGGCTCTGCGGCCTCTTCCGCGTCT

TCGCCTTCGCCCTCAGACGAGTCGGATCTCCCTTTGGGCCGCCTCCCCGCATC

GATACCGTCGACCTCGATCGAGACCTAGAAAAACATGGAGCAATCACAAGTA

GCAATACAGCAGCTACCAATGCTGATTGTGCCTGGCTAGAAGCACAAGAGGA

GGAGGAGGTGGGTTTTCCAGTCACACCTCAGGTACCTTTAAGACCAATGACT

TACAAGGCAGCTGTAGATCTTAGCCACTTTTTAAAAGAAAAGGGGGGACTGG

AAGGGCTAATTCACTCCCAACGAAGACAAGATATCCTTGATCTGTGGATCTA

CCACACACAAGGCTACTTCCCTGATTGGCAGAACTACACACCAGGGCCAGGG

ATCAGATATCCACTGACCTTTGGATGGTGCTACAAGCTAGTACCAGTTGAGC

AAGAGAAGGTAGAAGAAGCCAATGAAGGAGAGAACACCCGCTTGTTACACC

CTGTGAGCCTGCATGGGATGGATGACCCGGAGAGAGAAGTATTAGAGTGGA

GGTTTGACAGCCGCCTAGCATTTCATCACATGGCCCGAGAGCTGCATCCGGA

CTGTACTGGGTCTCTCTGGTTAGACCAGATCTGAGCCTGGGAGCTCTCTGGCT

AACTAGGGAACCCACTGCTTAAGCCTCAATAAAGCTTGCCTTGAGTGCTTCA

AGTAGTGTGTGCCCGTCTGTTGTGTGACTCTGGTAACTAGAGATCCCTCAGAC

CCTTTTAGTCAGTGTGGAAAATCTCTAGCAGGGCCCGTTTAAACCCGCTGATC

AGCCTCGACTGTGCCTTCTAGTTGCCAGCCATCTGTTGTTTGCCCCTCCCCCGT

GCCTTCCTTGACCCTGGAAGGTGCCACTCCCACTGTCCTTTCCTAATAAAATG

AGGAAATTGCATCGCATTGTCTGAGTAGGTGTCATTCTATTCTGGGGGGTGGG

GTGGGGCAGGACAGCAAGGGGGAGGATTGGGAAGACAATAGCAGGCATGCT

GGGGATGCGGTGGGCTCTATGGCTTCTGAGGCGGAAAGAACCAGCTGGGGCT

CTAGGGGGTATCCCCACGCGCCCTGTAGCGGCGCATTAAGCGCGGCGGGTGT

GGTGGTTACGCGCAGCGTGACCGCTACACTTGCCAGCGCCCTAGCGCCCGCT

CCTTTCGCTTTCTTCCCTTCCTTTCTCGCCACGTTCGCCGGCTTTCCCCGTCAA

GCTCTAAATCGGGGGCTCCCTTTAGGGTTCCGATTTAGTGCTTTACGGCACCT

CGACCCCAAAAAACTTGATTAGGGTGATGGTTCACGTAGTGGGCCATCGCCC

TGATAGACGGTTTTTCGCCCTTTGACGTTGGAGTCCACGTTCTTTAATAGTGG

ACTCTTGTTCCAAACTGGAACAACACTCAACCCTATCTCGGTCTATTCTTTTG

ATTTATAAGGGATTTTGCCGATTTCGGCCTATTGGTTAAAAAATGAGCTGATT

TAACAAAAATTTAACGCGAATTAATTCTGTGGAATGTGTGTCAGTTAGGGTGT

GGAAAGTCCCCAGGCTCCCCAGCAGGCAGAAGTATGCAAAGCATGCATCTCA

ATTAGTCAGCAACCAGGTGTGGAAAGTCCCCAGGCTCCCCAGCAGGCAGAAG

TATGCAAAGCATGCATCTCAATTAGTCAGCAACCATAGTCCCGCCCCTAACTC

CGCCCATCCCGCCCCTAACTCCGCCCAGTTCCGCCCATTCTCCGCCCCATGGC

TGACTAATTTTTTTTATTTATGCAGAGGCCGAGGCCGCCTCTGCCTCTGAGCT

ATTCCAGAAGTAGTGAGGAGGCTTTTTTGGAGGCCTAGGCTTTTGCAAAAAG

CTCCCGGGAGCTTGTATATCCATTTTCGGATCTGATCAGCACGTGTTGACAAT

TAATCATCGGCATAGTATATCGGCATAGTATAATACGACAAGGTGAGGAACT

AAACCATGGCCAAGTTGACCAGTGCCGTTCCGGTGCTCACCGCGCGCGACGT

CGCCGGAGCGGTCGAGTTCTGGACCGACCGGCTCGGGTTCTCCCGGGACTTC

GTGGAGGACGACTTCGCCGGTGTGGTCCGGGACGACGTGACCCTGTTCATCA

GCGCGGTCCAGGACCAGGTGGTGCCGGACAACACCCTGGCCTGGGTGTGGGT

GCGCGGCCTGGACGAGCTGTACGCCGAGTGGTCGGAGGTCGTGTCCACGAAC

TTCCGGGACGCCTCCGGGCCGGCCATGACCGAGATCGGCGAGCAGCCGTGGG

GGCGGGAGTTCGCCCTGCGCGACCCGGCCGGCAACTGCGTGCACTTCGTGGC

CGAGGAGCAGGACTGACACGTGCTACGAGATTTCGATTCCACCGCCGCCTTC

TATGAAAGGTTGGGCTTCGGAATCGTTTTCCGGGACGCCGGCTGGATGATCCT

CCAGCGCGGGGATCTCATGCTGGAGTTCTTCGCCCACCCCAACTTGTTTATTG

CAGCTTATAATGGTTACAAATAAAGCAATAGCATCACAAATTTCACAAATAA

AGCATTTTTTTCACTGCATTCTAGTTGTGGTTTGTCCAAACTCATCAATGTATC

TTATCATGTCTGTATACCGTCGACCTCTAGCTAGAGCTTGGCGTAATCATGGT

CATAGCTGTTTCCTGTGTGAAATTGTTATCCGCTCACAATTCCACACAACATA

CGAGCCGGAAGCATAAAGTGTAAAGCCTGGGGTGCCTAATGAGTGAGCTAAC

TCACATTAATTGCGTTGCGCTCACTGCCCGCTTTCCAGTCGGGAAACCTGTCG

TGCCAGCTGCATTAATGAATCGGCCAACGCGCGGGGAGAGGCGGTTTGCGTA

TTGGGCGCTCTTCCGCTTCCTCGCTCACTGACTCGCTGCGCTCGGTCGTTCGG

CTGCGGCGAGCGGTATCAGCTCACTCAAAGGCGGTAATACGGTTATCCACAG

AATCAGGGGATAACGCAGGAAAGAACATGTGAGCAAAAGGCCAGCAAAAGG

CCAGGAACCGTAAAAAGGCCGCGTTGCTGGCGTTTTTCCATAGGCTCCGCCC

CCCTGACGAGCATCACAAAAATCGACGCTCAAGTCAGAGGTGGCGAAACCC

GACAGGACTATAAAGATACCAGGCGTTTCCCCCTGGAAGCTCCCTCGTGCGC

TCTCCTGTTCCGACCCTGCCGCTTACCGGATACCTGTCCGCCTTTCTCCCTTCG

GGAAGCGTGGCGCTTTCTCATAGCTCACGCTGTAGGTATCTCAGTTCGGTGTA

GGTCGTTCGCTCCAAGCTGGGCTGTGTGCACGAACCCCCCGTTCAGCCCGAC

CGCTGCGCCTTATCCGGTAACTATCGTCTTGAGTCCAACCCGGTAAGACACG

ACTTATCGCCACTGGCAGCAGCCACTGGTAACAGGATTAGCAGAGCGAGGTA

TGTAGGCGGTGCTACAGAGTTCTTGAAGTGGTGGCCTAACTACGGCTACACT

AGAAGAACAGTATTTGGTATCTGCGCTCTGCTGAAGCCAGTTACCTTCGGAA

AAAGAGTTGGTAGCTCTTGATCCGGCAAACAAACCACCGCTGGTAGCGGTGG

TTTTTTTGTTTGCAAGCAGCAGATTACGCGCAGAAAAAAAGGATCTCAAGAA

GATCCTTTGATCTTTTCTACGGGGTCTGACGCTCAGTGGAACGAAAACTCACG

TTAAGGGATTTTGGTCATGAGATTATCAAAAAGGATCTTCACCTAGATCCTTT

TAAATTAAAAATGAAGTTTTAAATCAATCTAAAGTATATATGAGTAAACTTG

GTCTGACAGTTACCAATGCTTAATCAGTGAGGCACCTATCTCAGCGATCTGTC

TATTTCGTTCATCCATAGTTGCCTGACTCCCCGTCGTGTAGATAACTACGATA

CGGGAGGGCTTACCATCTGGCCCCAGTGCTGCAATGATACCGCGAGACCCAC

GCTCACCGGCTCCAGATTTATCAGCAATAAACCAGCCAGCCGGAAGGGCCGA

GCGCAGAAGTGGTCCTGCAACTTTATCCGCCTCCATCCAGTCTATTAATTGTT

GCCGGGAAGCTAGAGTAAGTAGTTCGCCAGTTAATAGTTTGCGCAACGTTGT

TGCCATTGCTACAGGCATCGTGGTGTCACGCTCGTCGTTTGGTATGGCTTCAT

TCAGCTCCGGTTCCCAACGATCAAGGCGAGTTACATGATCCCCCATGTTGTGC

AAAAAAGCGGTTAGCTCCTTCGGTCCTCCGATCGTTGTCAGAAGTAAGTTGG

CCGCAGTGTTATCACTCATGGTTATGGCAGCACTGCATAATTCTCTTACTGTC

ATGCCATCCGTAAGATGCTTTTCTGTGACTGGTGAGTACTCAACCAAGTCATT

CTGAGAATAGTGTATGCGGCGACCGAGTTGCTCTTGCCCGGCGTCAATACGG

GATAATACCGCGCCACATAGCAGAACTTTAAAAGTGCTCATCATTGGAAAAC

GTTCTTCGGGGCGAAAACTCTCAAGGATCTTACCGCTGTTGAGATCCAGTTCG

ATGTAACCCACTCGTGCACCCAACTGATCTTCAGCATCTTTTACTTTCACCAG

CGTTTCTGGGTGAGCAAAAACAGGAAGGCAAAATGCCGCAAAAAAGGGAAT

AAGGGCGACACGGAAATGTTGAATACTCATACTCTTCCTTTTTCAATATTATT

GAAGCATTTATCAGGGTTATTGTCTCATGAGCGGATACATATTTGAATGTATT

TAGAAAAATAAACAAATAGGGGTTCCGCGCACATTTCCCCGAAAAGTGCCAC

CTGAC

Plasmids used in construction of pSLIK4

>pFN22K::ICP8-P2A/Crimson

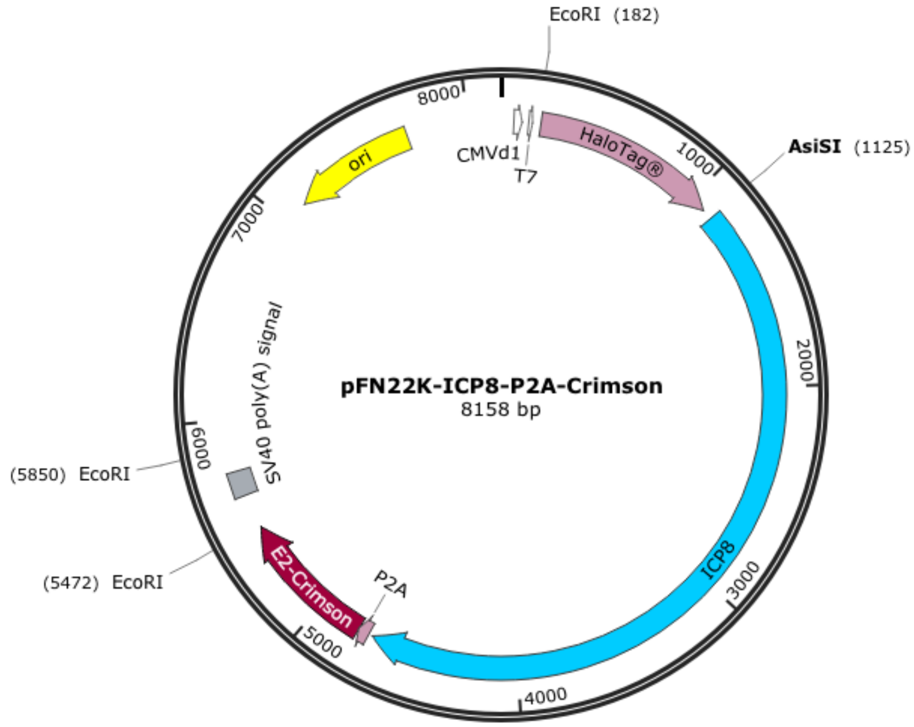

>pFN22K::ICP8-P2A/Crimson

CCAAAATCAACGGGACTTTCCAAAATGTCGTAATAACCCCGCCCCGTTGACG

CAAATGGGCGGTAGGCGTGTACGGTGGGAGGTCTATATAAGCAGAGCTGGTT

TAGTGAACCGTCAGATCACTAGAAGCTTAATACGACTCACTATAGGGTACGA

TTTAGGTGACACTATAGAATAAGGAGAATTCATCATGGCAGAAATCGGTACT

GGCTTTCCATTCGACCCCCATTATGTGGAAGTCCTGGGCGAGCGCATGCACTA

CGTCGATGTTGGTCCGCGCGATGGCACCCCTGTGCTGTTCCTGCACGGTAACC

CGACCTCCTCCTACGTGTGGCGCAACATCATCCCGCATGTTGCACCGACCCAT

CGCTGCATTGCTCCAGACCTGATCGGTATGGGCAAATCCGACAAACCAGACC

TGGGTTATTTCTTCGACGACCACGTCCGCTTCATGGATGCCTTCATCGAAGCC

CTGGGTCTGGAAGAGGTCGTCCTGGTCATTCACGACTGGGGCTCCGCTCTGG

GTTTCCACTGGGCCAAGCGCAATCCAGAGCGCGTCAAAGGTATTGCATTTAT

GGAGTTCATCCGCCCTATCCCGACCTGGGACGAATGGCCAGAATTTGCCCGC

GAGACCTTCCAGGCCTTCCGCACCACCGACGTCGGCCGCAAGCTGATCATCG

ATCAGAACGTTTTTATCGAGGGTACGCTGCCGATGGGTGTCGTCCGCCCGCTG

ACTGAAGTCGAGATGGACCATTACCGCGAGCCGTTCCTGAATCCTGTTGACC

GCGAGCCACTGTGGCGCTTCCCAAACGAGCTGCCAATCGCCGGTGAGCCAGC

GAACATCGTCGCGCTGGTCGAAGAATACATGGACTGGCTGCACCAGTCCCCT

GTCCCGAAGCTGCTGTTCTGGGGCACCCCAGGCGTTCTGATCCCACCGGCCG

AAGCCGCTCGCCTGGCCAAAAGCCTGCCTAACTGCAAGGCTGTGGACATCGG

CCCGGGTCTGAATCTGCTGCAAGAAGACAACCCGGACCTGATCGGCAGCGAG

ATCGCGCGCTGGCTGTCGACGCTCGAGATTTCCGGCGAGCCAACCACTGAGG

ATCTGTACTTTCAGAGCGATAACGCGATCGCCATGGAGACAAAGCCCAAGAC

GGCAACCACCATCAAGGTCCCCCCCGGGCCCCTGGGATACGTGTACGCTCGC

GCGTGTCCGTCCGAAGGCATCGAGCTTCTGGCGTTACTGTCGGCGCGCAGCG

GCGATGCCGACGTCGCCGTGGCGCCCCTGGTCGTGGGCCTGACCGTGGAGAG

CGGCTTTGAGGCCAACGTAGCCGTGGTCGTGGGTTCTCGCACGACGGGGCTC

GGGGGTACCGCGGTGTCCCTGAAACTGACGCCATCGCACTACAGCTCGTCCG

TGTACGTCTTTCACGGCGGCCGGCACCTGGACCCCAGCACCCAGGCCCCAAA
CCTGACGCGACTCTGCGAGCGGGCACGCCGCCATTTTGGCTTTTCGGACTACA
CCCCCCGGCCCGGCGACCTCAAACACGAGACGACGGGGGAGGCGCTGTGTG
AGCGCCTCGGCCTGGACCCGGACCGCGCCCTCCTGTATCTGGTCGTTACCGA
GGGCTTCAAGGAGGCCGTGTGCATCAACAACACCTTTCTGCACCTGGGAGGC
TCGGACAAGGTAACCATAGGCGGGGCGGAGGTGCACCGCATACCCGTGTATC
CGTTGCAGCTGTTCATGCCGGATTTTAGCCGGGTCATCGCCGAGCCGTTCAAC
GCCAACCACCGATCGATCGGGGAGAATTTTACCTACCCGCTTCCGTTTTTAA
CCGCCCCCTCAACCGCCTCCTGTTCGAGGCGGTCGTGGGACCCGCCGCCGTG
GCACTGCGATGCCGAAACGTGGACGCCGTGGCCCGCGCGGCCGCCCACCTGG
CGTTTGACGAAAACCACGAGGGCGCCGCCCTCCCCGCCGACATTACGTTCAC
GGCCTTCGAAGCCAGCCAGGGTAAGACCCCGCGGGGTGGGCGCGACGGCGG
CGGCAAGGGCCCGGCGGGCGGGTTCGAACAGCGCCTGGCCTCCGTCATGGCC
GGAGACGCCGCCCTGGCCCTCGAGTCTATCGTGTCGATGGCCGTCTTCGACG
AGCCGCCCACCGACATCTCCGCGTGGCCGCTGTGCGAGGGCCAGGACACGGC
CGCGGCCCGCGCCAACGCCGTCGGGGCGTACCTGGCGCGCGCCGCGGGACTC
GTGGGGGCCATGGTATTTAGCACCAACTCGGCCCTCCATCTCACCGAGGTGG
ACGACGCCGGTCCGGCGGACCCAAAGGACCACAGCAAACCCTCCTTTTACCG
CTTCTTCCTCGTGCCCGGGACCCACGTGGCGGCCAACCCACAGGTGGACCGC
GAGGGACACGTGGTGCCCGGGTTCGAGGGTCGGCCCACCGCGCCCCTCGTCG
GCGGAACCCAGGAATTTGCCGGCGAGCACCTGGCCATGCTGTGTGGGTTTTC
CCCGGCGCTGCTGGCCAAGATGCTGTTTTACCTGGAGCGCTGCGACGGCGGC
GTGATCGTCGGGCGCCAGGAGATGGACGTGTTTCGATACGTCGCGGACTCCA

ACCAGACCGACGTGCCCTGCAACCTGTGCACCTTCGACACGCGCCACGCCTG

CGTACACACGACGCTCATGCGCCTCCGGGCGCGCCATCCCAAGTTCGCCAGC

GCCGCCCGCGGAGCCATCGGCGTCTTCGGGACCATGAACAGCATGTACAGCG

ACTGCGACGTGCTGGGAAACTACGCCGCCTTCTCGGCCCTGAAGCGCGCGGA

CGGATCCGAGACCGCCCGGACCATCATGCAGGAGACGTACCGCGCGGCGAC

CGAGCGCGTCATGGCCGAACTCGAGACCCTGCAGTACGTGGACCAGGCGGTC

CCCACGGCCATGGGGCGGCTGGAGACCATCATCACCAACCGCGAGGCCCTGC

ATACGGTGGTGAACAACGTCAGGCAGGTCGTGGACCGCGAGGTGGAGCAGC

TGATGCGCAACCTGGTGGAGGGGAGGAACTTCAAGTTTCGCGACGGTCTGGG

CGAGGCCAACCACGCCATGTCCCTGACGCTGGACCCGTACGCGTGCGGGCCA

TGCCCCCTGCTTCAGCTTCTCGGGCGGCGATCCAACCTCGCCGTGTATCAGGA

CCTGGCCCTGAGCCAGTGCCACGGGGTGTTCGCCGGGCAGTCGGTCGAGGGG

CGCAACTTTCGCAATCAATTCCAACCGGTGCTGCGGCGGCGCGTGATGGACA

TGTTTAACAACGGGTTTCTGTCGGCCAAAACGCTGACGGTCGCGCTCTCGGA

GGGGGCGGCTATCTGCGCCCCCAGCCTAACGGCCGGCCAGACGGCCCCCGCC

GAGAGCAGCTTCGAGGGCGACGTTGCCCGCGTGACCCTGGGGTTTCCCAAGG

AGCTGCGCGTCAAGAGCCGCGTGTTGTTCGCGGGCGCGAGCGCCAACGCGTC

CGAGGCCGCCAAGGCGCGGGTCGCCAGCCTCCAGAGCGCCTACCAGAAGCC

CGACAAGCGCGTGGACATCCTCCTCGGACCGCTGGGCTTTCTGCTGAAGCAG

TTCCACGCGGCCATCTTCCCCAACGGCAAGCCCCCGGGGTCCAACCAGCCGA

ACCCGCAGTGGTTCTGGACGGCCCTCCAACGCAACCAGCTTCCCGCCCGGCT

CCTGTCGCGCGAGGACATCGAGACCATCGCGTTCATTAAAAAGTTTTCCCTGG

ACTACGGCGCGATAAACTTTATTAACCTGGCCCCCAACAACGTGAGCGAGCT

GGCGATGTACTACATGGCAAACCAGATTCTGCGGTACTGCGATCACTCGACA

TACTTCATCAACACCCTCACGGCCATCATCGCGGGGTCCCGCCGTCCCCCCAG

CGTGCAGGCGGCGGCCGCGTGGTCCGCGCAGGGCGGGGCGGGCCTGGAGGC

CGGGGCCCGCGCGCTGATGGACGCCGTGGACGCGCATCCGGGCGCGTGGAC

GTCCATGTTCGCCAGCTGCAACCTGCTGCGGCCCGTCATGGCGGCGCGCCCC

ATGGTCGTGTTGGGGTTGAGCATCAGCAAATACTACGGCATGGCCGGCAACG

ACCGTGTGTTTCAGGCCGGGAACTGGGCCAGCCTGATGGGCGGCAAAAACGC

GTGCCCGCTCCTTATTTTTGACCGCACCCGCAAGTTCGTCCTGGCCTGTCCCC

GGGCCGGGTTTGTGTGCGCGGCCTCGAACCTCGGCGGCGGAGCGCACGAAAG

CTCGCTGTGCGAGCAGCTCCGGGGCATTATCTCCGAGGGCGGGGCGGCCGTC

GCCAGTAGCGTGTTCGTGGCGACCGTGAAAAGCCTGGGGCCCCGCACCCAGC

AGCTGCAGATCGAGGACTGGCTGGCGCTCCTGGAGGACGAGTACCTAAGCGA

GGAGATGATGGAGCTGACCGCGCGTGCCCTGGAGCGCGGCAACGGCGAGTG

GTCGACGGACGCGGCCCTGGAGGTGGCGCACGAGGCCGAGGCCCTAGTCAG

CCAACTCGGCAACGCCGGGGAGGTGTTTAACTTTGGGGATTTTGGCTGCGAG

GACGACAACGCGACGCCGTTCGGCGGCCCGGGGGCCCCGGACCGGCATTTG

CCGGCCGCAAACGGGCGTTCCACGGGGATGACCCGTTTGGGGAGGGGCCCCC

CGACAAAAAGGGAGACCTGACGTTGGATATGCTGGTTTCCGGATCCGGAGCC

ACGAACTTCTCTCTGTTAAAGCAAGCAGGAGACGTGGAAGAAAACCCCGGTC

CTATGGATAGCACTGAGAACGTCATCAAGCCCTTCATGCGCTTCAAGGTGCA

CATGGAGGGCTCCGTGAACGGCCACGAGTTCGAGATCGAGGGCGTGGGCGA

GGGCAAGCCCTACGAGGGCACCCAGACCGCCAAGCTGCAAGTGACCAAGGG

CGGCCCCCTGCCCTTCGCCTGGGACATCCTGTCCCCCCAGTTCTTCTACGGCT

CCAAGGCGTACATCAAGCACCCCGCCGACATCCCCGACTACCTCAAGCAGTC

CTTCCCCGAGGGCTTCAAGTGGGAGCGCGTGATGAACTTCGAGGACGGCGGC

GTGGTGACCGTGACCCAGGACTCCTCCCTGCAGGACGGCACCCTCATCTACC

ACGTGAAGTTCATCGGCGTGAACTTCCCCTCCGACGGCCCCGTAATGCAGAA

GAAGACTCTGGGCTGGGAGCCCTCCACTGAGCGCAACTACCCCCGCGACGGC

GTGCTGAAGGGCGAGAACCACATGGCGCTGAAGCTGAAGGGCGGCGGCCAC

TACCTGTGTGAGTTCAAGTCCATCTACATGGCCAAGAAGCCCGTGAAGCTGC

CCGGCTACCACTACGTGGACTACAAGCTCGACATCACCTCCCACAACGAGGA

CTACACCGTGGTGGAGCAGTACGAGCGCGCCGAGGCCCGCCACCACCTGTTC

CAGTAGAAACGAATTCGGGCTCGGTACCCGGGGATCCTCTAGAGTCGACCTG

CAGGCATGCAAGCTGATCCGGCTGCTAACAAAGCCCGAAAGGAAGCTGAGTT

GGCTGCTGCCACCGCTGAGCAATAACTAGCATAACCCCTTGGGGCGGCCGCT

TCGAGCAGACATGATAAGATACATTGATGAGTTTGGACAAACCACAACTAGA

ATGCAGTGAAAAAAATGCTTTATTTGTGAAATTTGTGATGCTATTGCTTTATT

TGTAACCATTATAAGCTGCAATAAACAAGTTAACAACAACAATTGCATTCAT

TTTATGTTTCAGGTTCAGGGGGAGATGTGGGAGGTTTTTTAAGCAAGTAAAA

CCTCTACAAATGTGGTAAAATCGAATTCTAATGGATCCTCTTTGCGCTTGCGT

TTTCCCTTGTCCAGATAGCCCAGTAGCTGACATTCATCCGGGGTCAGCACCGT

TTCTGCGGACTGGCTTTCTACGTGTTCCGCTTCCTTTAGCAGCCCTTGCGCCCT

GAGTGCTTGCGGCAGCGTGAGCTTCAAAAGAATTGCCAGCTGGGGCGCCCTC

TGGTAAGGTTGGGAAGCCCTGCAAAGTAAACTGGATGGCTTTCTTGCCGCCA

AGGATCTGATGGCGCAGGGGATCAAGATCTGATCAAGAGACAGGATGACGG

TCGTTTCGCATGCTTGAACAAGATGGATTGCACGCAGGTTCTCCGGCCGCTTG

GGTGGAGAGGCTATTCGGCTATGACTGGGCACAACAGACAATCGGCTGCTCT

GATGCCGCCGTGTTCCGGCTGTCAGCGCAGGGGCGCCCGGTTCTTTTTGTCAA

GACCGACCTGTCCGGTGCCCTGAATGAACTGCAGGACGAGGCAGCGCGGCTA

TCGTGGCTGGCCACGACGGGCGTTCCTTGCGCAGCTGTGCTCGACGTTGTCAC

TGAAGCGGGAAGGGACTGGCTGCTATTGGGCGAAGTGCCGGGGCAGGATCTC

CTGTCATCTCACCTTGCTCCTGCCGAGAAAGTATCCATCATGGCTGATGCAAT

GCGGCGGCTGCATACGCTTGATCCGGCTACCTGCCCATTCGACCACCAAGCG

AAACATCGCATCGAGCGAGCACGCACTCGGATGGAAGCCGGTCTTGTCGATC

AGGATGATCTGGACGAAGAGCATCAGGGGCTCGCGCCAGCCGAACTGTTCGC

CAGGCTCAAGGCGCGTATGCCGGATGGTGAGGATCTCGTCGTGACTCATGGC

GATGCCTGCTTGCCGAATATCATGGTGGAAAATGGCCGCTTTTCTGGATTCAT

CGACTGTGGCCGGCTGGGTGTGGCGGACCGCTATCAGGACATAGCGTTGGCT

ACCCGTGATATTGCTGAAGAGCTTGGCGGCGAATGGGCTGACCGCTTCCTCG

TGCTTTACGGTATCGCCGCTCCCGATTCGCAGCGCATCGCCTTCTATCGCCTT

CTTGACGAGTTCTTCTGAGCGGGACTCTGGGGTTCGAAATGACCGACCAAGC

GACGCCCAACCGGTATCAGCTCACTCAAAGGCGGTAATACGGTTATCCACAG

AATCAGGGGATAACGCAGGAAAGAACATGTGAGCAAAAGGCCAGCAAAAGG

CCAGGAACCGTAAAAAGGCCGCGTTGCTGGCGTTTTTCCATAGGCTCCGCCC

CCCTGACGAGCATCACAAAAATCGACGCTCAAGTCAGAGGTGGCGAAACCC

GACAGGACTATAAAGATACCAGGCGTTTCCCCCTGGAAGCTCCCTCGTGCGC

TCTCCTGTTCCGACCCTGCCGCTTACCGGATACCTGTCCGCCTTTCTCCCTTCG

GGAAGCGTGGCGCTTTCTCATAGCTCACGCTGTAGGTATCTCAGTTCGGTGTA

GGTCGTTCGCTCCAAGCTGGGCTGTGTGCACGAACCCCCCGTTCAGCCCGAC

CGCTGCGCCTTATCCGGTAACTATCGTCTTGAGTCCAACCCGGTAAGACACG

ACTTATCGCCACTGGCAGCAGCCACTGGTAACAGGATTAGCAGAGCGAGGTA

TGTAGGCGGTGCTACAGAGTTCTTGAAGTGGTGGCCTAACTACGGCTACACT

AGAAGGACAGTATTTGGTATCTGCGCTCTGCTGAAGCCAGTTACCTTCGGAA

AAAGAGTTGGTAGCTCTTGATCCGGCAAACAAACCACCGCTGGTAGCGGTGG

TTTTTTTGTTTGCAAGCAGCAGATTACGCGCAGAAAAAAAGGATTTCAAGAA

GATCCTTTGATCTTTTCTACGGGGTCTGACGCTCAGTGGAACGAAAACTCACG

TTAAGGGATTTTGGTCATGAGATTATCAAAAAGGATCTTCACCTAGATCCTTT

TATAGTCCGGAAATACAGGAACGCACGCTGGATGGCCCTTCGCTGGGATGGT

GAAACCATGAAAAATGGCAGCTTCAGTGGATTAAGTGGGGGTAATGTGGCCT

GTACCCTCTGGTTGCATAGGTATTCATACGGTTAAAATTTATCAGGCGCGATT

GCGGCAGTTTTTCGGGTGGTTTGTTGCCATTTTTACCTGTCTGCTGCCGTGATC

GCGCTGAACGCGTTTTAGCGGTGCGTACAATTAAGGGATTATGGTAAATCCA

CTTACTGTCTGCCCTCGTAGCCATCGAGATAAACCGCAGTACTCCGGCCACG

ATGCGTCCGGCGTAGAGGATCGAGATCT

>pENTR2B/TREPitt::ICP8-P2A/Crimson

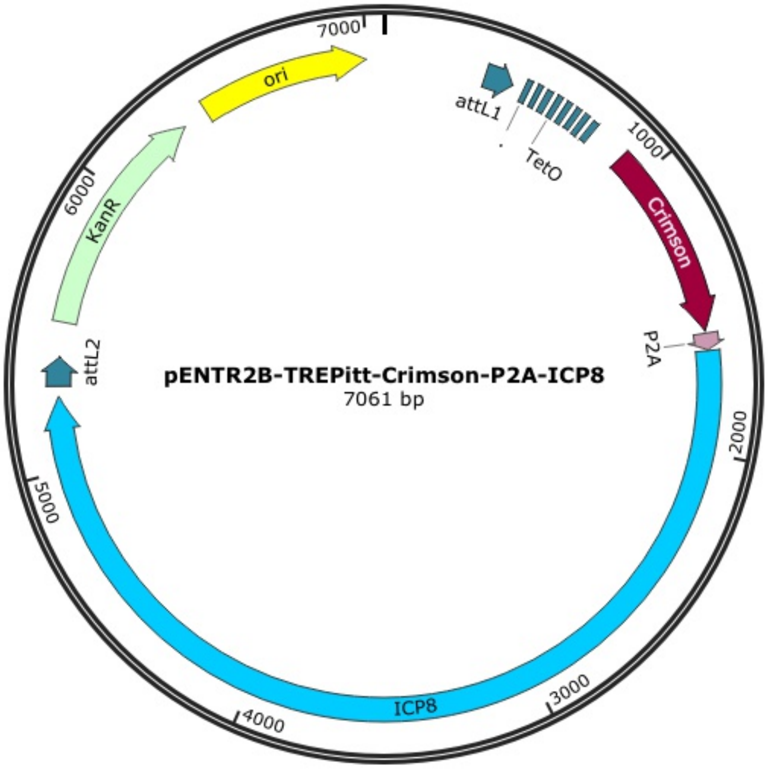

>pENTR2B/TREPitt::ICP8-P2A/Crimson

CTTTCCTGCGTTATCCCCTGATTCTGTGGATAACCGTATTACCGCTAGCATGG

ATCTCGGGGACGTCTAACTACTAAGCGAGAGTAGGGAACTGCCAGGCATCAA

ATAAAACGAAAGGCTCAGTCGGAAGACTGGGCCTTTCGTTTTATCTGTTGTTT

GTCGGTGAACGCTCTCCTGAGTAGGACAAATCCGCCGGGAGCGGATTTGAAC

GTTGTGAAGCAACGGCCCGGAGGGTGGCGGGCAGGACGCCCGCCATAAACT

GCCAGGCATCAAACTAAGCAGAAGGCCATCCTGACGGATGGCCTTTTTGCGT

TTCTACAAACTCTTCCTGTTAGTTAGTTACTTAAGCTCGGGCCCCAAATAATG

ATTTTATTTTGACTGATAGTGACCTGTTCGTTGCAACAAATTGATAAGCAATG

CTTTTTTATAATGCCAACTTTGTACAAAAAAGCAGGCTGGCGCCGGAACCAA

TTCAGATATCAAGTGCCACCTGACGTCTCCCTATCAGTGATAGAGAAGTCGA

CACGTCTCGAGCTCCCTATCAGTGATAGAGAAGGTACGTCTAGAACGTCTCC
CTATCAGTGATAGAGAAGTCGACACGTCTCGAGCTCCCTATCAGTGATAGAG
AAGGTACGTCTAGAACGTCTCCCTATCAGTGATAGAGAAGTCGACACGTCTC
GAGCTCCCTATCAGTGATAGAGAAGGTACGTCTAGAACGTCTCCCTATCAGT
GATAGAGAAGTCGACACGTCTCGAGCTCCCTATCAGTGATAGAGAAGGTACC
CCCTATATAAGCAGAGCTCGTTTAGTGAACCGTCAGATCGCCTGGAGACGCC
ATCCACGCTGTTTTGACCTCCATAGAAGACACCGGGACCGATCCAGCCTGGA
TCTCGCCATGGAGACAAAGCCCAAGACGGCAACCACCATCAAGGTCCCCCCC
GGGCCCCTGGGATACGTGTACGCTCGCGCGTGTCCGTCCGAAGGCATCGAGC
TTCTGGCGTTACTGTCGGCGCGCAGCGGCGATGCCGACGTCGCCGTGGCGCC
CCTGGTCGTGGGCCTGACCGTGGAGAGCGGCTTTGAGGCCAACGTAGCCGTG
GTCGTGGGTTCTCGCACGACGGGGCTCGGGGGTACCGCGGTGTCCCTGAAAC
TGACGCCATCGCACTACAGCTCGTCCGTGTACGTCTTTCACGGCGGCCGGCAC
CTGGACCCCAGCACCCAGGCCCCAAACCTGACGCGACTCTGCGAGCGGGCAC
GCCGCCATTTTGGCTTTTCGGACTACACCCCCCGGCCCGGCGACCTCAAACAC
GAGACGACGGGGGAGGCGCTGTGTGAGCGCCTCGGCCTGGACCCGGACCGC
GCCCTCCTGTATCTGGTCGTTACCGAGGGCTTCAAGGAGGCCGTGTGCATCAA
CAACACCTTTCTGCACCTGGGAGGCTCGGACAAGGTAACCATAGGCGGGGCG
GAGGTGCACCGCATACCCGTGTATCCGTTGCAGCTGTTCATGCCGGATTTTAG
CCGGGTCATCGCCGAGCCGTTCAACGCCAACCACCGATCGATCGGGGAGAAT
TTTACCTACCCGCTTCCGTTTTTAACCGCCCCCTCAACCGCCTCCTGTTCGAG
GCGGTCGTGGGACCCGCCGCCGTGGCACTGCGATGCCGAAACGTGGACGCCG
TGGCCCGCGCGGCCGCCCACCTGGCGTTTGACGAAAACCACGAGGGCGCCGC

CCTCCCCGCCGACATTACGTTCACGGCCTTCGAAGCCAGCCAGGGTAAGACC

CCGCGGGGTGGGCGCGACGGCGGCGGCAAGGGCCCGGCGGGCGGGTTCGAA

CAGCGCCTGGCCTCCGTCATGGCCGGAGACGCCGCCCTGGCCCTCGAGTCTA

TCGTGTCGATGGCCGTCTTCGACGAGCCGCCCACCGACATCTCCGCGTGGCC

GCTGTGCGAGGGCCAGGACACGGCCGCGGCCCGCGCCAACGCCGTCGGGGC

GTACCTGGCGCGCGCCGCGGGACTCGTGGGGGCCATGGTATTTAGCACCAAC

TCGGCCCTCCATCTCACCGAGGTGGACGACGCCGGTCCGGCGGACCCAAAGG

ACCACAGCAAACCCTCCTTTACCGCTTCTTCCTCGTGCCCGGGACCCACGTG

GCGGCCAACCCACAGGTGGACCGCGAGGGACACGTGGTGCCCGGGTTCGAG

GGTCGGCCCACCGCGCCCCTCGTCGGCGGAACCCAGGAATTTGCCGGCGAGC

ACCTGGCCATGCTGTGTGGGTTTTCCCCGGCGCTGCTGGCCAAGATGCTGTTT

TACCTGGAGCGCTGCGACGGCGGCGTGATCGTCGGGCGCCAGGAGATGGAC

GTGTTTCGATACGTCGCGGACTCCAACCAGACCGACGTGCCCTGCAACCTGT

GCACCTTCGACACGCGCCACGCCTGCGTACACACGACGCTCATGCGCCTCCG

GGCGCGCCATCCCAAGTTCGCCAGCGCCGCCCGCGGAGCCATCGGCGTCTTC

GGGACCATGAACAGCATGTACAGCGACTGCGACGTGCTGGGAAACTACGCC

GCCTTCTCGGCCCTGAAGCGCGCGGACGGATCCGAGACCGCCCGGACCATCA

TGCAGGAGACGTACCGCGCGGCGACCGAGCGCGTCATGGCCGAACTCGAGA

CCCTGCAGTACGTGGACCAGGCGGTCCCCACGGCCATGGGGCGGCTGGAGAC

CATCATCACCAACCGCGAGGCCCTGCATACGGTGGTGAACAACGTCAGGCAG

GTCGTGGACCGCGAGGTGGAGCAGCTGATGCGCAACCTGGTGGAGGGGAGG

AACTTCAAGTTTCGCGACGGTCTGGGCGAGGCCAACCACGCCATGTCCCTGA

CGCTGGACCCGTACGCGTGCGGGCCATGCCCCCTGCTTCAGCTTCTCGGGCG

GCGATCCAACCTCGCCGTGTATCAGGACCTGGCCCTGAGCCAGTGCCACGGG

GTGTTCGCCGGGCAGTCGGTCGAGGGGCGCAACTTTCGCAATCAATTCCAAC

CGGTGCTGCGGCGGCGCGTGATGGACATGTTTAACAACGGGTTTCTGTCGGC

CAAAACGCTGACGGTCGCGCTCTCGGAGGGGGCGGCTATCTGCGCCCCCAGC

CTAACGGCCGGCCAGACGGCCCCCGCCGAGAGCAGCTTCGAGGGCGACGTTG

CCCGCGTGACCCTGGGGTTTCCCAAGGAGCTGCGCGTCAAGAGCCGCGTGTT

GTTCGCGGGCGCGAGCGCCAACGCGTCCGAGGCCGCCAAGGCGCGGGTCGC

CAGCCTCCAGAGCGCCTACCAGAAGCCCGACAAGCGCGTGGACATCCTCCTC

GGACCGCTGGGCTTTCTGCTGAAGCAGTTCCACGCGGCCATCTTCCCCAACG

GCAAGCCCCCGGGGTCCAACCAGCCGAACCCGCAGTGGTTCTGGACGGCCCT

CCAACGCAACCAGCTTCCCGCCCGGCTCCTGTCGCGCGAGGACATCGAGACC

ATCGCGTTCATTAAAAAGTTTTCCCTGGACTACGGCGCGATAAACTTTATTAA

CCTGGCCCCCAACAACGTGAGCGAGCTGGCGATGTACTACATGGCAAACCAG

ATTCTGCGGTACTGCGATCACTCGACATACTTCATCAACACCCTCACGGCCAT

CATCGCGGGGTCCCGCCGTCCCCCCAGCGTGCAGGCGGCGGCCGCGTGGTCC

GCGCAGGGCGGGGCGGGCCTGGAGGCCGGGGCCCGCGCGCTGATGGACGCC

GTGGACGCGCATCCGGGCGCGTGGACGTCCATGTTCGCCAGCTGCAACCTGC

TGCGGCCCGTCATGGCGGCGCGCCCCATGGTCGTGTTGGGGTTGAGCATCAG

CAAATACTACGGCATGGCCGGCAACGACCGTGTGTTTCAGGCCGGGAACTGG

GCCAGCCTGATGGGCGGCAAAAACGCGTGCCCGCTCCTTATTTTTGACCGCA

CCCGCAAGTTCGTCCTGGCCTGTCCCCGGGCCGGGTTTGTGTGCGCGGCCTCG

AACCTCGGCGGCGGAGCGCACGAAAGCTCGCTGTGCGAGCAGCTCCGGGGC

ATTATCTCCGAGGGCGGGGCGGCCGTCGCCAGTAGCGTGTTCGTGGCGACCG

TGAAAAGCCTGGGGCCCCGCACCCAGCAGCTGCAGATCGAGGACTGGCTGGC
GCTCCTGGAGGACGAGTACCTAAGCGAGGAGATGATGGAGCTGACCGCGCGT
GCCCTGGAGCGCGGCAACGGCGAGTGGTCGACGGACGCGGCCCTGGAGGTG
GCGCACGAGGCCGAGGCCCTAGTCAGCCAACTCGGCAACGCCGGGGAGGTG
TTTAACTTTGGGGATTTTGGCTGCGAGGACGACAACGCGACGCCGTTCGGCG
GCCCGGGGGCCCCGGGACCGGCATTTGCCGGCCGCAAACGGGCGTTCCACGG
GGATGACCCGTTTGGGGAGGGGCCCCCCGACAAAAAGGGAGACCTGACGTT
GGATATGCTGGTTTCCGGATCCGGAGCCACGAACTTCTCTCTGTTAAAGCAAG
CAGGAGACGTGGAAGAAAACCCCGGTCCTATGGATAGCACTGAGAACGTCAT
CAAGCCCTTCATGCGCTTCAAGGTGCACATGGAGGGCTCCGTGAACGGCCAC
GAGTTCGAGATCGAGGGCGTGGGCGAGGGCAAGCCCTACGAGGGCACCCAG
ACCGCCAAGCTGCAAGTGACCAAGGGCGGCCCCCTGCCCTTCGCCTGGGACA
TCCTGTCCCCCCAGTTCTTCTACGGCTCCAAGGCGTACATCAAGCACCCCGCC
GACATCCCCGACTACCTCAAGCAGTCCTTCCCCGAGGGCTTCAAGTGGGAGC
GCGTGATGAACTTCGAGGACGGCGGCGTGGTGACCGTGACCCAGGACTCCTC
CCTGCAGGACGGCACCCTCATCTACCACGTGAAGTTCATCGGCGTGAACTTC
CCCTCCGACGGCCCCGTAATGCAGAAGAAGACTCTGGGCTGGGAGCCCTCCA
CTGAGCGCAACTACCCCCGCGACGGCGTGCTGAAGGGCGAGAACCACATGG
CGCTGAAGCTGAAGGGCGGCGGCCACTACCTGTGTGAGTTCAAGTCCATCTA
CATGGCCAAGAAGCCCGTGAAGCTGCCCGGCTACCACTACGTGGACTACAAG
CTCGACATCACCTCCCACAACGAGGACTACACCGTGGTGGAGCAGTACGAGC
GCGCCGAGGCCCGCCACCACCTGTTCCAGTAGAAACGAATTCGCGGCCGCAC
TCGAGATATCTAGACCCAGCTTTCTTGTACAAAGTTGGCATTATAAGAAAGC

ATTGCTTATCAATTTGTTGCAACGAACAGGTCACTATCAGTCAAAATAAAATC
ATTATTTGCCATCCAGCTGCAGCTCTGGCCCGTGTCTCAAAATCTCTGATGTT
ACATTGCACAAGATAAAAATATATCATCATGAACAATAAAACTGTCTGCTTA
CATAAACAGTAATACAAGGGGTGTTATGAGCCATATTCAACGGGAAACGTCG
AGGCCGCGATTAAATTCCAACATGGATGCTGATTTATATGGGTATAAATGGG
CTCGCGATAATGTCGGGCAATCAGGTGCGACAATCTATCGCTTGTATGGGAA
GCCCGATGCGCCAGAGTTGTTTCTGAAACATGGCAAAGGTAGCGTTGCCAAT
GATGTTACAGATGAGATGGTCAGACTAAACTGGCTGACGGAATTTATGCCTC
TTCCGACCATCAAGCATTTTATCCGTACTCCTGATGATGCATGGTTACTCACC
ACTGCGATCCCCGGAAAAACAGCATTCCAGGTATTAGAAGAATATCCTGATT
CAGGTGAAAATATTGTTGATGCGCTGGCAGTGTTCCTGCGCCGGTTGCATTCG
ATTCCTGTTTGTAATTGTCCTTTTAACAGCGATCGCGTATTTCGTCTCGCTCAG
GCGCAATCACGAATGAATAACGGTTTGGTTGATGCGAGTGATTTTGATGACG
AGCGTAATGGCTGGCCTGTTGAACAAGTCTGGAAAGAAATGCATAAACTTTT
GCCATTCTCACCGGATTCAGTCGTCACTCATGGTGATTTCTCACTTGATAACC
TTATTTTTGACGAGGGGAAATTAATAGGTTGTATTGATGTTGGACGAGTCGGA
ATCGCAGACCGATACCAGGATCTTGCCATCCTATGGAACTGCCTCGGTGAGTT
TTCTCCTTCATTACAGAAACGGCTTTTTCAAAAATATGGTATTGATAATCCTG
ATATGAATAAATTGCAGTTTCATTTGATGCTCGATGAGTTTTTCTAATCAGAA
TTGGTTAATTGGTTGTAACATTATTCAGATTGGGCCCCGTTCCACTGAGCGTC
AGACCCCGTAGAAAAGATCAAAGGATCTTCTTGAGATCCTTTTTTTCTGCGCG
TAATCTGCTGCTTGCAAACAAAAAAACCACCGCTACCAGCGGTGGTTTGTTTG
CCGGATCAAGAGCTACCAACTCTTTTTCCGAAGGTAACTGGCTTCAGCAGAG

CGCAGATACCAAATACTGTTCTTCTAGTGTAGCCGTAGTTAGGCCACCACTTC

AAGAACTCTGTAGCACCGCCTACATACCTCGCTCTGCTAATCCTGTTACCAGT

GGCTGCTGCCAGTGGCGATAAGTCGTGTCTTACCGGGTTGGACTCAAGACGA

TAGTTACCGGATAAGGCGCAGCGGTCGGGCTGAACGGGGGGTTCGTGCACAC

AGCCCAGCTTGGAGCGAACGACCTACACCGAACTGAGATACCTACAGCGTGA

GCTATGAGAAAGCGCCACGCTTCCCGAAGGGAGAAAGGCGGACAGGTATCC

GGTAAGCGGCAGGGTCGGAACAGGAGAGCGCACGAGGGAGCTTCCAGGGGG

AAACGCCTGGTATCTTTATAGTCCTGTCGGGTTTCGCCACCTCTGACTTGAGC

GTCGATTTTTGTGATGCTCGTCAGGGGGGCGGAGCCTATGGAAAAACGCCAG

CAACGCGGCCTTTTTACGGTTCCTGGCCTTTTGCTGGCCTTTTGCTCACATGTT

>pSLIK /TREPitt::ICP8-P2A/Crimson

AKA 4-pSLIK-Zeo/TREPitt::ICP8-P2A/Crimson or pSLIK4. Sequence verified from 2410 to 7547 bp.

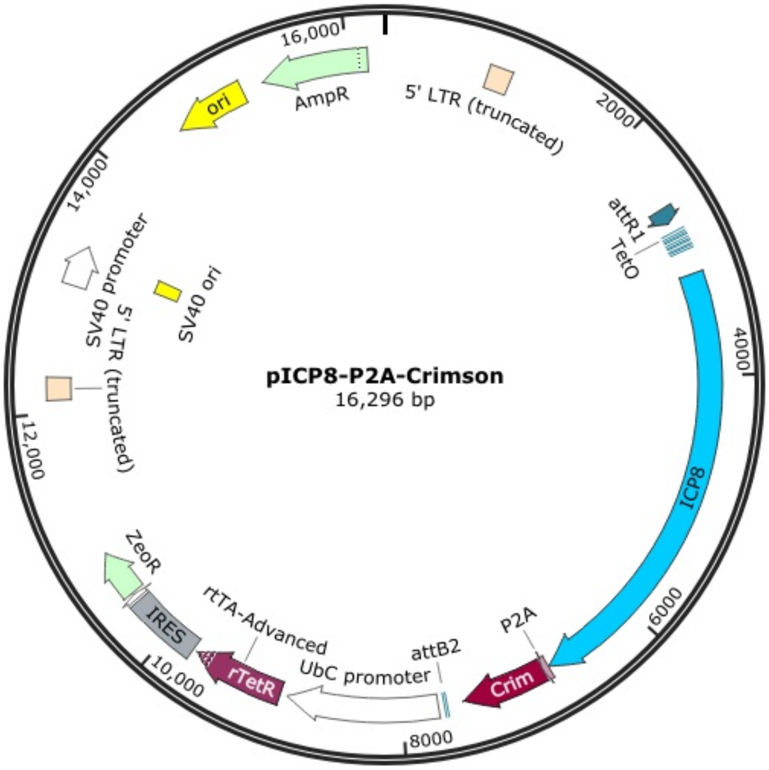

>4-pSLIK-Zeo/TREPitt::ICP8-P2A/Crimson

GTCGACGGATCGGGAGATCTCCCGATCCCCTATGGTGCACTCTCAGTACAAT

CTGCTCTGATGCCGCATAGTTAAGCCAGTATCTGCTCCCTGCTTGTGTGTTGG

AGGTCGCTGAGTAGTGCGCGAGCAAAATTTAAGCTACAACAAGGCAAGGCTT

GACCGACAATTGCATGAAGAATCTGCTTAGGGTTAGGCGTTTTGCGCTGCTTC

GCGATGTACGGGCCAGATATACGCGTTGACATTGATTATTGACTAGTTATTAA

TAGTAATCAATTACGGGGTCATTAGTTCATAGCCCATATATGGAGTTCCGCGT

TACATAACTTACGGTAAATGGCCCGCCTGGCTGACCGCCCAACGACCCCCGC

CCATTGACGTCAATAATGACGTATGTTCCCATAGTAACGCCAATAGGGACTTT

CCATTGACGTCAATGGGTGGAGTATTTACGGTAAACTGCCCACTTGGCAGTA
CATCAAGTGTATCATATGCCAAGTACGCCCCCTATTGACGTCAATGACGGTA
AATGGCCCGCCTGGCATTATGCCCAGTACATGACCTTATGGGACTTTCCTACT
TGGCAGTACATCTACGTATTAGTCATCGCTATTACCATGGTGATGCGGTTTTG
GCAGTACATCAATGGGCGTGGATAGCGGTTTGACTCACGGGGATTTCCAAGT
CTCCACCCCATTGACGTCAATGGGAGTTTGTTTTGGCACCAAAATCAACGGG
ACTTTCCAAAATGTCGTAACAACTCCGCCCCATTGACGCAAATGGGCGGTAG
GCGTGTACGGTGGGAGGTCTATATAAGCAGCGTTTTGCCTGTACTGGGTCT
CTCTGGTTAGACCAGATCTGAGCCTGGGAGCTCTCTGGCTAACTAGGGAACC
CACTGCTTAAGCCTCAATAAAGCTTGCCTTGAGTGCTTCAAGTAGTGTGTGCC
CGTCTGTTGTGTGACTCTGGTAACTAGAGATCCCTCAGACCCTTTTAGTCAGT
GTGGAAAATCTCTAGCAGTGGCGCCCGAACAGGGACTTGAAAGCGAAAGGG
AAACCAGAGGAGCTCTCTCGACGCAGGACTCGGCTTGCTGAAGCGCGCACGG
CAAGAGGCGAGGGGCGGCGACTGGTGAGTACGCCAAAAATTTTGACTAGCG
GAGGCTAGAAGGAGAGAGATGGGTGCGAGAGCGTCAGTATTAAGCGGGGGA
GAATTAGATCGCGATGGGAAAAAATTCGGTTAAGGCCAGGGGGAAAGAAAA
AATATAAATTAAAACATATAGTATGGGCAAGCAGGGAGCTAGAACGATTCGC
AGTTAATCCTGGCCTGTTAGAAACATCAGAAGGCTGTAGACAAATACTGGGA
CAGCTACAACCATCCCTTCAGACAGGATCAGAAGAACTTAGATCATTATATA
ATACAGTAGCAACCCTCTATTGTGTGCATCAAAGGATAGAGATAAAAGACAC
CAAGGAAGCTTTAGACAAGATAGAGGAAGAGCAAAACAAAAGTAAGACCAC
CGCACAGCAAGCGGCCGCTGATCTTCAGACCTGGAGGAGGAGATATGAGGG
ACAATTGGAGAAGTGAATTATATAAATATAAAGTAGTAAAAATTGAACCATT

AGGAGTAGCACCCACCAAGGCAAAGAGAAGAGTGGTGCAGAGAGAAAAAA
GAGCAGTGGGAATAGGAGCTTTGTTCCTTGGGTTCTTGGGAGCAGCAGGAAG
CACTATGGGCGCAGCGTCAATGACGCTGACGGTACAGGCCAGACAATTATTG
TCTGGTATAGTGCAGCAGCAGAACAATTTGCTGAGGGCTATTGAGGCGCAAC
AGCATCTGTTGCAACTCACAGTCTGGGGCATCAAGCAGCTCCAGGCAAGAAT
CCTGGCTGTGGAAAGATACCTAAAGGATCAACAGCTCCTGGGGATTTGGGGT
TGCTCTGGAAAACTCATTTGCACCACTGCTGTGCCTTGGAATGCTAGTTGGAG
TAATAAATCTCTGGAACAGATTTGGAATCACACGACCTGGATGGAGTGGGAC
AGAGAAATTAACAATTACACAAGCTTAATACACTCCTTAATTGAAGAATCGC
AAAACCAGCAAGAAAAGAATGAACAAGAATTATTGGAATTAGATAAATGGG
CAAGTTTGTGGAATTGGTTTAACATAACAAATTGGCTGTGGTATATAAAATTA
TTCATAATGATAGTAGGAGGCTTGGTAGGTTTAAGAATAGTTTTTGCTGTACT
TTCTATAGTGAATAGAGTTAGGCAGGGATATTCACCATTATCGTTTCAGACCC
ACCTCCCAACCCCGAGGGGACCCGACAGGCCCGAAGGAATAGAAGAAGAAG
GTGGAGAGAGAGACAGAGACAGATCCATTCGATTAGTGAACGGATCGGCAC
TGCGTGCGCCAATTCTGCAGACAAATGGCAGTATTCATCCACAATTTTAAAA
GAAAAGGGGGGATTGGGGGGTACAGTGCAGGGGAAAGAATAGTAGACATAA
TAGCAACAGACATACAAACTAAAGAATTACAAAAACAAATTACAAAAATTC
AAAATTTTCGGGTTTATTACAGGGACAGCAGAGATCCAGTTTGGTTAATTCTA
GACAGTTATCAACAAGTTTGTACAAAAAAGCTGAACGAGAAACGTAAAATG
ATATAAATATCAATATATTAAATTAGATTTTGCATAAAAAACAGACTACATA
ATACTGTAAAACACAACATATCCAGTCACTATGGGCGCCGGAACCAATTCAga
tatcAAGTGCCACCTGACGTCTCCCTATCAGTGATAGAGAAGTCGACACGTCTC

GAGCTCCCTATCAGTGATAGAGAAGGTACGTCTAGAACGTCTCCCTATCAGT
GATAGAGAAGTCGACACGTCTCGAGCTCCCTATCAGTGATAGAGAAGGTACG
TCTAGAACGTCTCCCTATCAGTGATAGAGAAGTCGACACGTCTCGAGCTCCCT
ATCAGTGATAGAGAAGGTACGTCTAGAACGTCTCCCTATCAGTGATAGAGAA
GTCGACACGTCTCGAGCTCCCTATCAGTGATAGAGAAGGTACCCCCTATATA
AGCAGAGCTCGTTTAGTGAACCGTCAGATCGCCTGGAGACGCCATCCACGCT
GTTTTGACCTCCATAGAAGACACCGGGACCGATCCAGCCTggatctCGCCATGGA
GACAAAGCCCAAGACGGCAACCACCATCAAGGTCCCCCCCGGCCCCTGGG
ATACGTGTACGCTCGCGCGTGTCCGTCCGAAGGCATCGAGCTTCTGGCGTTAC
TGTCGGCGCGCAGCGGCGATGCCGACGTCGCCGTGGCGCCCCTGGTCGTGGG
CCTGACCGTGGAGAGCGGCTTTGAGGCCAACGTAGCCGTGGTCGTGGGTTCT
CGCACGACGGGGCTCGGGGGTACCGCGGTGTCCCTGAAACTGACGCCATCGC
ACTACAGCTCGTCCGTGTACGTCTTTCACGGCGGCCGGCACCTGGACCCCAG
CACCCAGGCCCCAAACCTGACGCGACTCTGCGAGCGGGCACGCCGCCATTTT
GGCTTTTCGGACTACACCCCCCGGCCCGGCGACCTCAAACACGAGACGACGG
GGGAGGCGCTGTGTGAGCGCCTCGGCCTGGACCCGGACCGCGCCCTCCTGTA
TCTGGTCGTTACCGAGGGCTTCAAGGAGGCCGTGTGCATCAACAACACCTTTC
TGCACCTGGGAGGCTCGGACAAGGTAACCATAGGCGGGGCGGAGGTGCACC
GCATACCCGTGTATCCGTTGCAGCTGTTCATGCCGGATTTTAGCCGGGTCATC
GCCGAGCCGTTCAACGCCAACCACCGATCGATCGGGGAGAATTTTACCTACC
CGCTTCCGTTTTTTAACCGCCCCCTCAACCGCCTCCTGTTCGAGGCGGTCGTG
GGACCCGCCGCCGTGGCACTGCGATGCCGAAACGTGGACGCCGTGGCCCGCG
CGGCCGCCCACCTGGCGTTTGACGAAAACCACGAGGGCGCCGCCCTCCCCGC

CGACATTACGTTCACGGCCTTCGAAGCCAGCCAGGGTAAGACCCCGCGGGGT
GGGCGCGACGGCGGCGGCAAGGGCCCGGCGGGCGGGTTCGAACAGCGCCTG
GCCTCCGTCATGGCCGGAGACGCCGCCCTGGCCCTCGAGTCTATCGTGTCGAT
GGCCGTCTTCGACGAGCCGCCCACCGACATCTCCGCGTGGCCGCTGTGCGAG
GGCCAGGACACGGCCGCGGCCCGCGCCAACGCCGTCGGGGCGTACCTGGCG
CGCGCCGCGGGACTCGTGGGGGCCATGGTATTTAGCACCAACTCGGCCCTCC
ATCTCACCGAGGTGGACGACGCCGGTCCGGCGGACCCAAAGGACCACAGCA
AACCCTCCTTTTACCGCTTCTTCCTCGTGCCCGGGACCCACGTGGCGGCCAAC
CCACAGGTGGACCGCGAGGGACACGTGGTGCCCGGGTTCGAGGGTCGGCCC
ACCGCGCCCCTCGTCGGCGGAACCCAGGAATTTGCCGGCGAGCACCTGGCCA
TGCTGTGTGGGTTTTCCCCGGCGCTGCTGGCCAAGATGCTGTTTTACCTGGAG
CGCTGCGACGGCGGCGTGATCGTCGGGCGCCAGGAGATGGACGTGTTTCGAT
ACGTCGCGGACTCCAACCAGACCGACGTGCCCTGCAACCTGTGCACCTTCGA
CACGCGCCACGCCTGCGTACACACGACGCTCATGCGCCTCCGGGCGCGCCAT
CCCAAGTTCGCCAGCGCCGCCCGCGGAGCCATCGGCGTCTTCGGGACCATGA
ACAGCATGTACAGCGACTGCGACGTGCTGGGAAACTACGCCGCCTTCTCGGC
CCTGAAGCGCGCGGACGGATCCGAGACCGCCCGGACCATCATGCAGGAGAC
GTACCGCGCGGCGACCGAGCGCGTCATGGCCGAACTCGAGACCCTGCAGTAC
GTGGACCAGGCGGTCCCCACGGCCATGGGGCGGCTGGAGACCATCATCACCA
ACCGCGAGGCCCTGCATACGGTGGTGAACAACGTCAGGCAGGTCGTGGACCG
CGAGGTGGAGCAGCTGATGCGCAACCTGGTGGAGGGGAGGAACTTCAAGTTT
CGCGACGGTCTGGGCGAGGCCAACCACGCCATGTCCCTGACGCTGGACCCGT
ACGCGTGCGGGCCATGCCCCCTGCTTCAGCTTCTCGGGCGGCGATCCAACCTC

GCCGTGTATCAGGACCTGGCCCTGAGCCAGTGCCACGGGGTGTTCGCCGGGC
AGTCGGTCGAGGGGCGCAACTTTCGCAATCAATTCCAACCGGTGCTGCGGCG
GCGCGTGATGGACATGTTTAACAACGGGTTTCTGTCGGCCAAAACGCTGACG
GTCGCGCTCTCGGAGGGGGCGGCTATCTGCGCCCCCAGCCTAACGGCCGGCC
AGACGGCCCCCGCCGAGAGCAGCTTCGAGGGCGACGTTGCCCGCGTGACCCT
GGGGTTTCCCAAGGAGCTGCGCGTCAAGAGCCGCGTGTTGTTCGCGGGCGCG
AGCGCCAACGCGTCCGAGGCCGCCAAGGCGCGGGTCGCCAGCCTCCAGAGC
GCCTACCAGAAGCCCGACAAGCGCGTGGACATCCTCCTCGGACCGCTGGGCT
TTCTGCTGAAGCAGTTCCACGCGGCCATCTTCCCCAACGGCAAGCCCCCGGG
GTCCAACCAGCCGAACCCGCAGTGGTTCTGGACGGCCCTCCAACGCAACCAG
CTTCCCGCCCGGCTCCTGTCGCGCGAGGACATCGAGACCATCGCGTTCATTAA
AAAGTTTTCCCTGGACTACGGCGCGATAAACTTTATTAACCTGGCCCCCAACA
ACGTGAGCGAGCTGGCGATGTACTACATGGCAAACCAGATTCTGCGGTACTG
CGATCACTCGACATACTTCATCAACACCCTCACGGCCATCATCGCGGGGTCCC
GCCGTCCCCCCAGCGTGCAGGCGGCGGCCGCGTGGTCCGCGCAGGGCGGGGC
GGGCCTGGAGGCCGGGGCCCGCGCGCTGATGGACGCCGTGGACGCGCATCC
GGGCGCGTGGACGTCCATGTTCGCCAGCTGCAACCTGCTGCGGCCCGTCATG
GCGGCGCGCCCCATGGTCGTGTTGGGGTTGAGCATCAGCAAATACTACGGCA
TGGCCGGCAACGACCGTGTGTTTCAGGCCGGGAACTGGGCCAGCCTGATGGG
CGGCAAAAACGCGTGCCCGCTCCTTATTTTGACCGCACCCGCAAGTTCGTCC
TGGCCTGTCCCCGGGCCGGGTTTGTGTGCGCGGCCTCGAACCTCGGCGGCGG
AGCGCACGAAAGCTCGCTGTGCGAGCAGCTCCGGGGCATTATCTCCGAGGGC
GGGGCGGCCGTCGCCAGTAGCGTGTTCGTGGCGACCGTGAAAAGCCTGGGGC

CCCGCACCCAGCAGCTGCAGATCGAGGACTGGCTGGCGCTCCTGGAGGACGA

GTACCTAAGCGAGGAGATGATGGAGCTGACCGCGCGTGCCCTGGAGCGCGG

CAACGGCGAGTGGTCGACGGACGCGGCCCTGGAGGTGGCGCACGAGGCCGA

GGCCCTAGTCAGCCAACTCGGCAACGCCGGGGAGGTGTTTAACTTTGGGGAT

TTTGGCTGCGAGGACGACAACGCGACGCCGTTCGGCGGCCCGGGGGCCCCGG

GACCGGCATTTGCCGGCCGCAAACGGGCGTTCCACGGGGATGACCCGTTTGG

GGAGGGGCCCCCCGACAAAAAGGGAGACCTGACGTTGGATATGCTGGTTTcc

GGATCCGGAGCCACGAACTTCTCTCTGTTAAAGCAAGCAGGAGACGTGGAAG

AAAACCCCGGTCCTATGGATAGCACTGAGAACGTCATCAAGCCCTTCATGCG

CTTCAAGGTGCACATGGAGGGCTCCGTGAACGGCCACGAGTTCGAGATCGAG

GGCGTGGGCGAGGGCAAGCCCTACGAGGGCACCCAGACCGCCAAGCTGCAA

GTGACCAAGGGCGGCCCCCTGCCCTTCGCCTGGGACATCCTGTCCCCCCAGTT

CTTCTACGGCTCCAAGGCGTACATCAAGCACCCCGCCGACATCCCCGACTAC

CTCAAGCAGTCCTTCCCCGAGGGCTTCAAGTGGGAGCGCGTGATGAACTTCG

AGGACGGCGGCGTGGTGACCGTGACCCAGGACTCCTCCCTGCAGGACGGCAC

CCTCATCTACCACGTGAAGTTCATCGGCGTGAACTTCCCCTCCGACGGCCCCG

TAATGCAGAAGAAGACTCTGGGCTGGGAGCCCTCCACTGAGCGCAACTACCC

CCGCGACGGCGTGCTGAAGGGCGAGAACCACATGGCGCTGAAGCTGAAGGG

CGGCGGCCACTACCTGTGTGAGTTCAAGTCCATCTACATGGCCAAGAAGCCC

GTGAAGCTGCCCGGCTACCACTACGTGGACTACAAGCTCGACATCACCTCCC

ACAACGAGGACTACACCGTGGTGGAGCAGTACGAGCGCGCCGAGGCCCGCC

ACCACCTGTTCCAGTAGAAACGAATTCGCGGCCGCACTCGAGATATCTAGAT

AGTGACTGGATATGTTGTGTTTTACAGTATTATGTAGTCTGTTTTTTATGCAAA

ATCTAATTTAATATATTGATATTTATATCATTTTACGTTTCTCGTTCAGCTTTC

TTGTACAAAGTGGTTGATAACATCTCGAGCCATAAAGATGGTTAATTAACCC

ACCCAAGATCTGGCCTCCGCGCCGGGTTTTGGCGCCTCCCGCGGGCGCCCCC

CTCCTCACGGCGAGCGCTGCCACGTCAGACGAAGGGCGCAGCGAGCGTCCTG

ATCCTTCCGCCCGGACGCTCAGGACAGCGGCCCGCTGCTCATAAGACTCGGC

CTTAGAACCCCAGTATCAGCAGAAGGACATTTTAGGACGGGACTTGGGTGAC

TCTAGGGCACTGGTTTTCTTTCCAGAGAGCGGAACAGGCGAGGAAAAGTAGT

CCCTTCTCGGCGATTCTGCGGAGGGATCTCCGTGGGGCGGTGAACGCCGATG

ATTATATAAGGACGCGCCGGGTGTGGCACAGCTAGTTCCGTCGCAGCCGGGA

TTTGGGTCGCGGTTCTTGTTTGTGGATCGCTGTGATCGTCACTTGGTGAGTAG

CGGGCTGCTGGGCTGGCCGGGGCTTTCGTGGCCGCCGGGCCGCTCGGTGGGA

CGGAAGCGTGTGGAGAGACCGCCAAGGGCTGTAGTCTGGGTCCGCGAGCAA

GGTTGCCCTGAACTGGGGGTTGGGGGGAGCGCAGCAAAATGGCGGCTGTTCC

CGAGTCTTGAATGGAAGACGCTTGTGAGGCGGGCTGTGAGGTCGTTGAAACA

AGGTGGGGGGCATGGTGGGCGGCAAGAACCCAAGGTCTTGAGGCCTTCGCTA

ATGCGGGAAAGCTCTTATTCGGGTGAGATGGGCTGGGGCACCATCTGGGGAC

CCTGACGTGAAGTTTGTCACTGACTGGAGAACTCGGTTTGTCGTCTGTTGCGG

GGGCGGCAGTTATGGCGGTGCCGTTGGGCAGTGCACCCGTACCTTTGGGAGC

GCGCGCCCTCGTCGTGTCGTGACGTCACCCGTTCTGTTGGCTTATAATGCAGG

GTGGGGCCACCTGCCGGTAGGTGTGCGGTAGGCTTTTCTCCGTCGCAGGACG

CAGGGTTCGGGCCTAGGGTAGGCTCTCCTGAATCGACAGGCGCCGGACCTCT

GGTGAGGGGAGGGATAAGTGAGGCGTCAGTTTCTTTGGTCGGTTTTATGTAC

CTATCTTCTTAAGTAGCTGAAGCTCCGGTTTTGAACTATGCGCTCGGGGTTGG

CGAGTGTGTTTTGTGAAGTTTTTAGGCACCTTTTGAAATGTAATCATTTGGG

TCAATATGTAATTTTCAGTGTTAGACTAGTAAATTGTCCGCTAAATTCTGGCC

GTTTTTGGCTTTTTTGTTAGACGAAGCTTGGTACCGAGCTCGGATCTCCACCC

CGTACCGGTCCTGCAGTCGAATTCACCATGTCTAGACTGGACAAGAGCAAAG

TCATAAACGGAGCTCTGGAATTACTCAATGGTGTCGGTATCGAAGGCCTGAC

GACAAGGAAACTCGCTCAAAAGCTGGGAGTTGAGCAGCCTACCCTGTACTGG

CACGTGAAGAACAAGCGGGCCCTGCTCGATGCCCTGCCAATCGAGATGCTGG

ACAGGCATCATACCCACTTCTGCCCCCTGGAAGGCGAGTCATGGCAAGACTT

TCTGCGGAACAACGCCAAGTCATACCGCTGTGCTCTCCTCTCACATCGCGACG

GGGCTAAAGTGCATCTCGGCACCCGCCCAACAGAGAAACAGTACGAAACCCT

GGAAAATCAGCTCGCGTTCCTGTGTCAGCAAGGCTTCTCCCTGGAGAACGCA

CTGTACGCTCTGTCCGCCGTGGGCCACTTTACACTGGGCTGCGTATTGGAGGA

ACAGGAGCATCAAGTAGCAAAAGAGGAAAGAGAGACACCTACCACCGATTC

TATGCCCCCACTTCTGAGACAAGCAATTGAGCTGTTCGACCGGCAGGGAGCC

GAACCTGCCTTCCTTTTCGGCCTGGAACTAATCATATGTGGCCTGGAGAAACA

GCTAAAGTGCGAAAGCGGCGGGCCGACCGACGCCCTTGACGATTTTGACTTA

GACATGCTCCCAGCCGATGCCCTTGACGACTTTGACCTTGATATGCTGCCTGC

TGACGCTCTTGACGATTTTGACCTTGACATGCTCCCCGGGTAACTAAGTAAGG

ATCCGCGGCCGCACTAGAGGAATTCCGCCCCTCTCCCTCCCCCCCCCCTAACG

TTACTGGCCGAAGCCGCTTGGAATAAGGCCGGTGTGTGTTTGTCTATATGTTA

TTTTCCACCATATTGCCGTCTTTTGGCAATGTGAGGGCCCGGAAACCTGGCCC

TGTCTTCTTGACGAGCATTCCTAGGGGTCTTTCCCCTCTCGCCAAAGGAATGC

AAGGTCTGTTGAATGTCGTGAAGGAAGCAGTTCCTCTGGAAGCTTCTTGAAG

ACAAACAACGTCTGTAGCGACCCTTTGCAGGCAGCGGAACCCCCCACCTGGC

GACAGGTGCCTCTGCGGCCAAAAGCCACGTGTATAAGATACACCTGCAAAGG

CGGCACAACCCCAGTGCCACGTTGTGAGTTGGATAGTTGTGGAAAGAGTCAA

ATGGCTCTCCTCAAGCGTAGTCAACAAGGGGCTGAAGGATGCCCAGAAGGTA

CCCCATTGTATGGGAATCTGATCTGGGGCCTCGGTGCACATGCTTTACATGTG

TTTAGTCGAGGTTAAAAAAACGTCTAGGCCCCCCGAACCACGGGGACGTGGT

TTTCCTTTGAAAAACACGATGATAAGCTTACCGGTACGCGTGATGTGTTGACA

ATTAATCATCGGCATAGTATATCGGCATAGTATAATACGACAAGGTGAGGAA

CTAAACCATGGCCAAGTTGACCAGTGCCGTTCCGGTGCTCACCGCGCGCGAC

GTCGCCGGAGCGGTCGAGTTCTGGACCGACCGGCTCGGGTTCTCCCGGGACT

TCGTGGAGGACGACTTCGCCGGTGTGGTCCGGGACGACGTGACCCTGTTCAT

CAGCGCGGTCCAGGACCAGGTGGTGCCGGACAACACCCTGGCCTGGGTGTGG

GTGCGCGGCCTGGACGAGCTGTACGCCGAGTGGTCGGAGGTCGTGTCCACGA

ACTTCCGGGACGCCTCCGGGCCGGCCATGACCGAGATCGGCGAGCAGCCGTG

GGGGCGGGAGTTCGCCCTGCGCGACCCGGCCGGCAACTGCGTGCACTTCGTG

GCCGAGGAGCAGGACTGACACATCTGTACAAGTAAAGCGGCCGCGACTCTA

GATCATAATCAGCCATACCACATTTGTAGAGGTTTTACTTGCTTTAAAAAACC

TCCCACACCTCCCCCTGAACCTGAAACATAAAATGAATGCAATTGTTGTTGTT

TAGTCCCTCCCAATTCGATATCAAGCTTATCGATAATCAACCTCTGGATTACA

AAATTTGTGAAAGATTGACTGGTATTCTTAACTATGTTGCTCCTTTTACGCTAT

GTGGATACGCTGCTTTAATGCCTTTGTATCATGCTATTGCTTCCCGTATGGCTT

TCATTTTCTCCTCCTTGTATAAATCCTGGTTGCTGTCTCTTTATGAGGAGTTGT

GGCCCGTTGTCAGGCAACGTGGCGTGGTGTGCACTGTGTTTGCTGACGCAAC

CCCCACTGGTTGGGGCATTGCCACCACCTGTCAGCTCCTTTCCGGGACTTTCG

CTTTCCCCCTCCCTATTGCCACGGCGGAACTCATCGCCGCCTGCCTTGCCCGC

TGCTGGACAGGGGCTCGGCTGTTGGGCACTGACAATTCCGTGGTGTTGTCGG

GGAAATCATCGTCCTTTCCTTGGCTGCTCGCCTGTGTTGCCACCTGGATTCTG

CGCGGGACGTCCTTCTGCTACGTCCCTTCGGCCCTCAATCCAGCGGACCTTCC

TTCCCGCGGCCTGCTGCCGGCTCTGCGGCCTCTTCCGCGTCTTCGCCTTCGCC

CTCAGACGAGTCGGATCTCCCTTTGGGCCGCCTCCCCGCATCGATACCGTCGA

CCTCGATCGAGACCTAGAAAAACATGGAGCAATCACAAGTAGCAATACAGC

AGCTACCAATGCTGATTGTGCCTGGCTAGAAGCACAAGAGGAGGAGGAGGT

GGGTTTTCCAGTCACACCTCAGGTACCTTTAAGACCAATGACTTACAAGGCA

GCTGTAGATCTTAGCCACTTTTTAAAAGAAAAGGGGGGACTGGAAGGGCTAA

TTCACTCCCAACGAAGACAAGATATCCTTGATCTGTGGATCTACCACACACA

AGGCTACTTCCCTGATTGGCAGAACTACACACCAGGGCCAGGGATCAGATAT

CCACTGACCTTTGGATGGTGCTACAAGCTAGTACCAGTTGAGCAAGAGAAGG

TAGAAGAAGCCAATGAAGGAGAGAACACCCGCTTGTTACACCCTGTGAGCCT

GCATGGGATGGATGACCCGGAGAGAGAAGTATTAGAGTGGAGGTTTGACAG

CCGCCTAGCATTTCATCACATGGCCCGAGAGCTGCATCCGGACTGTACTGGG

TCTCTCTGGTTAGACCAGATCTGAGCCTGGGAGCTCTCTGGCTAACTAGGGAA

CCCACTGCTTAAGCCTCAATAAAGCTTGCCTTGAGTGCTTCAAGTAGTGTGTG

CCCGTCTGTTGTGTGACTCTGGTAACTAGAGATCCCTCAGACCCTTTTAGTCA

GTGTGGAAAATCTCTAGCAGGGCCCGTTTAAACCCGCTGATCAGCCTCGACT

GTGCCTTCTAGTTGCCAGCCATCTGTTGTTTGCCCCTCCCCCGTGCCTTCCTTG

ACCCTGGAAGGTGCCACTCCCACTGTCCTTTCCTAATAAAATGAGGAAATTGC

ATCGCATTGTCTGAGTAGGTGTCATTCTATTCTGGGGGGTGGGGTGGGGCAG
GACAGCAAGGGGGAGGATTGGGAAGACAATAGCAGGCATGCTGGGGATGCG
GTGGGCTCTATGGCTTCTGAGGCGGAAAGAACCAGCTGGGGCTCTAGGGGGT
ATCCCCACGCGCCCTGTAGCGGCGCATTAAGCGCGGCGGGTGTGGTGGTTAC
GCGCAGCGTGACCGCTACACTTGCCAGCGCCCTAGCGCCCGCTCCTTTCGCTT
TCTTCCCTTCCTTTCTCGCCACGTTCGCCGGCTTTCCCCGTCAAGCTCTAAATC
GGGGGCTCCCTTTAGGGTTCCGATTTAGTGCTTTACGGCACCTCGACCCCAAA
AAACTTGATTAGGGTGATGGTTCACGTAGTGGGCCATCGCCCTGATAGACGG
TTTTTCGCCCTTTGACGTTGGAGTCCACGTTCTTTAATAGTGGACTCTTGTTCC
AAACTGGAACAACACTCAACCCTATCTCGGTCTATTCTTTTGATTTATAAGGG
ATTTTGCCGATTTCGGCCTATTGGTTAAAAAATGAGCTGATTTAACAAAAATT
TAACGCGAATTAATTCTGTGGAATGTGTGTCAGTTAGGGTGTGGAAAGTCCC
CAGGCTCCCCAGCAGGCAGAAGTATGCAAAGCATGCATCTCAATTAGTCAGC
AACCAGGTGTGGAAAGTCCCCAGGCTCCCCAGCAGGCAGAAGTATGCAAAG
CATGCATCTCAATTAGTCAGCAACCATAGTCCCGCCCCTAACTCCGCCCATCC
CGCCCCTAACTCCGCCCAGTTCCGCCCATTCTCCGCCCCATGGCTGACTAATT
TTTTTTATTTATGCAGAGGCCGAGGCCGCCTCTGCCTCTGAGCTATTCCAGAA
GTAGTGAGGAGGCTTTTTTGGAGGCCTAGGCTTTTGCAAAAAGCTCCCGGGA
GCTTGTATATCCATTTTCGGATCTGATCAGCACGTGTTGACAATTAATCATCG
GCATAGTATATCGGCATAGTATAATACGACAAGGTGAGGAACTAAACCATGG
CCAAGTTGACCAGTGCCGTTCCGGTGCTCACCGCGCGCGACGTCGCCGGAGC
GGTCGAGTTCTGGACCGACCGGCTCGGGTTCTCCCGGGACTTCGTGGAGGAC
GACTTCGCCGGTGTGGTCCGGGACGACGTGACCCTGTTCATCAGCGCGGTCC

AGGACCAGGTGGTGCCGGACAACACCCTGGCCTGGGTGTGGGTGCGCGGCCT

GGACGAGCTGTACGCCGAGTGGTCGGAGGTCGTGTCCACGAACTTCCGGGAC

GCCTCCGGGCCGGCCATGACCGAGATCGGCGAGCAGCCGTGGGGGCGGGAG

TTCGCCCTGCGCGACCCGGCCGGCAACTGCGTGCACTTCGTGGCCGAGGAGC

AGGACTGACACGTGCTACGAGATTTCGATTCCACCGCCGCCTTCTATGAAAG

GTTGGGCTTCGGAATCGTTTTCCGGGACGCCGGCTGGATGATCCTCCAGCGCG

GGGATCTCATGCTGGAGTTCTTCGCCCACCCCAACTTGTTTATTGCAGCTTAT

AATGGTTACAAATAAAGCAATAGCATCACAAATTTCACAAATAAAGCATTTT

TTTCACTGCATTCTAGTTGTGGTTTGTCCAAACTCATCAATGTATCTTATCATG

TCTGTATACCGTCGACCTCTAGCTAGAGCTTGGCGTAATCATGGTCATAGCTG

TTTCCTGTGTGAAATTGTTATCCGCTCACAATTCCACACAACATACGAGCCGG

AAGCATAAAGTGTAAAGCCTGGGGTGCCTAATGAGTGAGCTAACTCACATTA

ATTGCGTTGCGCTCACTGCCCGCTTTCCAGTCGGGAAACCTGTCGTGCCAGCT

GCATTAATGAATCGGCCAACGCGCGGGGAGAGGCGGTTTGCGTATTGGGCGC

TCTTCCGCTTCCTCGCTCACTGACTCGCTGCGCTCGGTCGTTCGGCTGCGGCG

AGCGGTATCAGCTCACTCAAAGGCGGTAATACGGTTATCCACAGAATCAGGG

GATAACGCAGGAAAGAACATGTGAGCAAAAGGCCAGCAAAAGGCCAGGAAC

CGTAAAAAGGCCGCGTTGCTGGCGTTTTTCCATAGGCTCCGCCCCCCTGACGA

GCATCACAAAAATCGACGCTCAAGTCAGAGGTGGCGAAACCCGACAGGACT

ATAAAGATACCAGGCGTTTCCCCCTGGAAGCTCCCTCGTGCGCTCTCCTGTTC

CGACCCTGCCGCTTACCGGATACCTGTCCGCCTTTCTCCCTTCGGGAAGCGTG

GCGCTTTCTCATAGCTCACGCTGTAGGTATCTCAGTTCGGTGTAGGTCGTTCG

CTCCAAGCTGGGCTGTGTGCACGAACCCCCCGTTCAGCCCGACCGCTGCGCC

TTATCCGGTAACTATCGTCTTGAGTCCAACCCGGTAAGACACGACTTATCGCC
ACTGGCAGCAGCCACTGGTAACAGGATTAGCAGAGCGAGGTATGTAGGCGGT
GCTACAGAGTTCTTGAAGTGGTGGCCTAACTACGGCTACACTAGAAGAACAG
TATTTGGTATCTGCGCTCTGCTGAAGCCAGTTACCTTCGGAAAAAGAGTTGGT
AGCTCTTGATCCGGCAAACAAACCACCGCTGGTAGCGGTGGTTTTTTTGTTTG
CAAGCAGCAGATTACGCGCAGAAAAAAAGGATCTCAAGAAGATCCTTTGATC
TTTTCTACGGGGTCTGACGCTCAGTGGAACGAAAACTCACGTTAAGGGATTTT
GGTCATGAGATTATCAAAAAGGATCTTCACCTAGATCCTTTTAAATTAAAAAT
GAAGTTTTAAATCAATCTAAAGTATATATGAGTAAACTTGGTCTGACAGTTAC
CAATGCTTAATCAGTGAGGCACCTATCTCAGCGATCTGTCTATTTCGTTCATC
CATAGTTGCCTGACTCCCCGTCGTGTAGATAACTACGATACGGGAGGGCTTA
CCATCTGGCCCCAGTGCTGCAATGATACCGCGAGACCCACGCTCACCGGCTC
CAGATTTATCAGCAATAAACCAGCCAGCCGGAAGGGCCGAGCGCAGAAGTG
GTCCTGCAACTTTATCCGCCTCCATCCAGTCTATTAATTGTTGCCGGGAAGCT
AGAGTAAGTAGTTCGCCAGTTAATAGTTTGCGCAACGTTGTTGCCATTGCTAC
AGGCATCGTGGTGTCACGCTCGTCGTTTGGTATGGCTTCATTCAGCTCCGGTT
CCCAACGATCAAGGCGAGTTACATGATCCCCCATGTTGTGCAAAAAAGCGGT
TAGCTCCTTCGGTCCTCCGATCGTTGTCAGAAGTAAGTTGGCCGCAGTGTTAT
CACTCATGGTTATGGCAGCACTGCATAATTCTCTTACTGTCATGCCATCCGTA
AGATGCTTTTCTGTGACTGGTGAGTACTCAACCAAGTCATTCTGAGAATAGTG
TATGCGGCGACCGAGTTGCTCTTGCCCGGCGTCAATACGGGATAATACCGCG
CCACATAGCAGAACTTTAAAAGTGCTCATCATTGGAAAACGTTCTTCGGGGC
GAAAACTCTCAAGGATCTTACCGCTGTTGAGATCCAGTTCGATGTAACCCACT

CGTGCACCCAACTGATCTTCAGCATCTTTTACTTTCACCAGCGTTTCTGGGTG

AGCAAAAACAGGAAGGCAAAATGCCGCAAAAAAGGGAATAAGGGCGACAC

GGAAATGTTGAATACTCATACTCTTCCTTTTTCAATATTATTGAAGCATTTATC

AGGGTTATTGTCTCATGAGCGGATACATATTTGAATGTATTTAGAAAAATAA

ACAAATAGGGGTTCCGCGCACATTTCCCCGAAAAGTGCCACCTGAC

Plasmids used in construction of pSLIK5

>pUC19::NLS/HA-HumBeta-P2A/Crimson

AKA pUC19ΔKpnI-Kan::NLS/HA-HumBeta-P2A/Crimson.

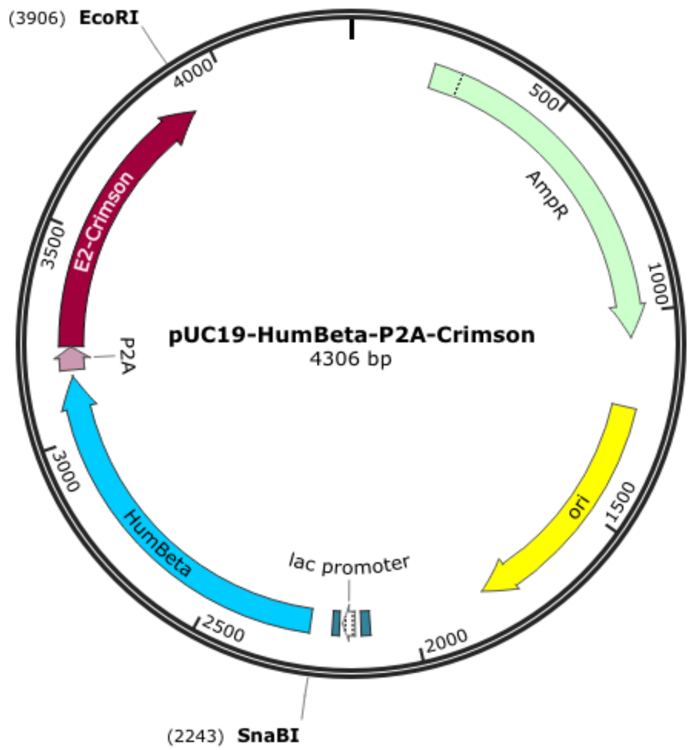

>pUC19::NLS/HA-HumBeta-P2A/Crimson

GACGAAAGGGCCTCGTGATACGCCTATTTTTATAGGTTAATGTCATGATAATA

ATGGTTTCTTAGACGTCAGGTGGCACTTTTCGGGGAAATGTGCGCGGAACCC

CTATTTGTTTATTTTTCTAAATACATTCAAATATGTATCCGCTCATGAGACAAT
AACCCTGATAAATGCTTCAATAATATTGAAAAAGGAAGAGTATGAGTATTCA
ACATTTCCGTGTCGCCCTTATTCCCTTTTTTGCGGCATTTTGCCTTCCTGTTTTT
GCTCACCCAGAAACGCTGGTGAAAGTAAAAGATGCTGAAGATCAGTTGGGTG
CACGAGTGGGTTACATCGAACTGGATCTCAACAGCGGTAAGATCCTTGAGAG
TTTTCGCCCCGAAGAACGTTTTCCAATGATGAGCACTTTTAAAGTTCTGCTAT
GTGGCGCGGTATTATCCCGTATTGACGCCGGGCAAGAGCAACTCGGTCGCCG
CATACACTATTCTCAGAATGACTTGGTTGAGTACTCACCAGTCACAGAAAAG
CATCTTACGGATGGCATGACAGTAAGAGAATTATGCAGTGCTGCCATAACCA
TGAGTGATAACACTGCGGCCAACTTACTTCTGACAACGATCGGAGGACCGAA
GGAGCTAACCGCTTTTTTGCACAACATGGGGGATCATGTAACTCGCCTTGATC
GTTGGGAACCGGAGCTGAATGAAGCCATACCAAACGACGAGCGTGACACCA
CGATGCCTGTAGCAATGGCAACAACGTTGCGCAAACTATTAACTGGCGAACT
ACTTACTCTAGCTTCCCGGCAACAATTAATAGACTGGATGGAGGCGGATAAA
GTTGCAGGACCACTTCTGCGCTCGGCCCTTCCGGCTGGCTGGTTTATTGCTGA
TAAATCTGGAGCCGGTGAGCGTGGGTCTCGCGGTATCATTGCAGCACTGGGG
CCAGATGGTAAGCCCTCCCGTATCGTAGTTATCTACACGACGGGGAGTCAGG
CAACTATGGATGAACGAAATAGACAGATCGCTGAGATAGGTGCCTCACTGAT
TAAGCATTGGTAACTGTCAGACCAAGTTTACTCATATATACTTTAGATTGATT
TAAAACTTCATTTTTAATTTAAAAGGATCTAGGTGAAGATCCTTTTTGATAAT
CTCATGACCAAAATCCCTTAACGTGAGTTTTCGTTCCACTGAGCGTCAGACCC
CGTAGAAAAGATCAAAGGATCTTCTTGAGATCCTTTTTTTCTGCGCGTAATCT
GCTGCTTGCAAACAAAAAAACCACCGCTACCAGCGGTGGTTTGTTTGCCGGA

TCAAGAGCTACCAACTCTTTTTCCGAAGGTAACTGGCTTCAGCAGAGCGCAG

ATACCAAATACTGTTCTTCTAGTGTAGCCGTAGTTAGGCCACCACTTCAAGAA

CTCTGTAGCACCGCCTACATACCTCGCTCTGCTAATCCTGTTACCAGTGGCTG

CTGCCAGTGGCGATAAGTCGTGTCTTACCGGGTTGGACTCAAGACGATAGTT

ACCGGATAAGGCGCAGCGGTCGGGCTGAACGGGGGGTTCGTGCACACAGCC

CAGCTTGGAGCGAACGACCTACACCGAACTGAGATACCTACAGCGTGAGCTA

TGAGAAAGCGCCACGCTTCCCGAAGGGAGAAAGGCGGACAGGTATCCGGTA

AGCGGCAGGGTCGGAACAGGAGAGCGCACGAGGGAGCTTCCAGGGGGAAAC

GCCTGGTATCTTTATAGTCCTGTCGGGTTTCGCCACCTCTGACTTGAGCGTCG

ATTTTTGTGATGCTCGTCAGGGGGGCGGAGCCTATGGAAAAACGCCAGCAAC

GCGGCCTTTTTACGGTTCCTGGCCTTTTGCTGGCCTTTTGCTCACATGTTCTTT

CCTGCGTTATCCCCTGATTCTGTGGATAACCGTATTACCGCCTTTGAGTGAGC

TGATACCGCTCGCCGCAGCCGAACGACCGAGCGCAGCGAGTCAGTGAGCGA

GGAAGCGGAAGAGCGCCCAATACGCAAACCGCCTCTCCCCGCGCGTTGGCCG

ATTCATTAATGCAGCTGGCACGACAGGTTTCCCGACTGGAAAGCGGGCAGTG

AGCGCAACGCAATTAATGTGAGTTAGCTCACTCATTAGGCACCCCAGGCTTT

ACACTTTATGCTTCCGGCTCGTATGTTGTGTGGAATTGTGAGCGGATAACAAT

TTCACACAGGAAACAGCTATGACCATGATTACGCCAAGCTTTACGTACCACC

ATGGTGCCTCCCAAGAAGAAGAGGAAGGTCGAGGACCCCAAGTACCCCTAT

GACGTGCCCGATTACGCTGGCGGAGGCGGAGGCTCCGGCGGAGGAGGAGGA

TCCGGAGGAGGCAGCATGTCCACAGCTCTCGCCACCCTGGCCGGAAAACTGG

CCGAGAGAGTGGGCATGGACTCCGTGGATCCCCAGGAGCTGATCACCACACT

GAGGCAAACCGCCTTCAAGGGAGATGCTTCCGACGCCCAATTCATCGCCCTC

CTCATCGTGGCCAACCAGTACGGCCTGAACCCTTGGACCAAGGAGATTTACG

CCTTCCCCGACAAGCAGAATGGCATTGTCCCTGTGGTCGGCGTGGACGGCTG

GAGCAGAATCATTAACGAGAACCAGCAGTTTGACGGCATGGACTTCGAGCAG

GACAACGAGAGCTGCACATGCAGGATCTACAGGAAGGACAGGAACCATCCT

ATCTGCGTCACCGAGTGGATGGATGAGTGCAGAAGAGAACCCTTCAAGACCA

GAGAGGGCAGAGAGATCACAGGACCCTGGCAGTCCCATCCTAAGAGAATGC

TCAGACACAAGGCCATGATCCAGTGTGCCAGGCTCGCTTTTGGCTTCGCCGG

CATCTATGATAAGGACGAAGCCGAGAGGATCGTGGAAAATACCGCCTACACC

GCTGAAAGGCAGCCCGAAAGGGACATTACACCCGTGAACGACGAGACCATG

CAGGAGATCAATACCCTGCTCATTGCCCTGGACAAAACCTGGGACGATGACC

TCCTCCCCCTCTGCAGCCAAATCTTCAGGAGGGACATCAGGGCCAGCTCCGA

ACTCACACAAGCCGAAGCTGTGAAGGCCCTCGGATTCCTCAAACAGAAGGCC

GCTGAGCAGAAGGTCGCCGCCGTTTCCGGATCCGGAGCCACGAACTTCTCTC

TGTTAAAGCAAGCAGGAGACGTGGAAGAAAACCCCGGTCCTATGGATAGCA

CTGAGAACGTCATCAAGCCCTTCATGCGCTTCAAGGTGCACATGGAGGGCTC

CGTGAACGGCCACGAGTTCGAGATCGAGGGCGTGGGCGAGGGCAAGCCCTA

CGAGGGCACCCAGACCGCCAAGCTGCAAGTGACCAAGGGCGGCCCCCTGCC

CTTCGCCTGGGACATCCTGTCCCCCCAGTTCTTCTACGGCTCCAAGGCGTACA

TCAAGCACCCCGCCGACATCCCCGACTACCTCAAGCAGTCCTTCCCCGAGGG

CTTCAAGTGGGAGCGCGTGATGAACTTCGAGGACGGCGGCGTGGTGACCGTG

ACCCAGGACTCCTCCCTGCAGGACGGCACCCTCATCTACCACGTGAAGTTCA

TCGGCGTGAACTTCCCCTCCGACGGCCCCGTAATGCAGAAGAAGACTCTGGG

CTGGGAGCCCTCCACTGAGCGCAACTACCCCCGCGACGGCGTGCTGAAGGGC

GAGAACCACATGGCGCTGAAGCTGAAGGGCGGCGGCCACTACCTGTGTGAGT

TCAAGTCCATCTACATGGCCAAGAAGCCCGTGAAGCTGCCCGGCTACCACTA

CGTGGACTACAAGCTCGACATCACCTCCCACAACGAGGACTACACCGTGGTG

GAGCAGTACGAGCGCGCCGAGGCCCGCCACCACCTGTTCCAGTAGAAACGA

ATTCACTGGCCGTCGTTTTACAACGTCGTGACTGGGAAAACCCTGGCGTTACC

CAACTTAATCGCCTTGCAGCACATCCCCCTTTCGCCAGCTGGCGTAATAGCGA

AGAGGCCCGCACCGATCGCCCTTCCCAACAGTTGCGCAGCCTGAATGGCGAA

TGGCGCCTGATGCGGTATTTTCTCCTTACGCATCTGTGCGGTATTTCACACCG

CATATGGTGCACTCTCAGTACAATCTGCTCTGATGCCGCATAGTTAAGCCAGC

CCCGACACCCGCCAACACCCGCTGACGCGCCCTGACGGGCTTGTCTGCTCCC

GGCATCCGCTTACAGACAAGCTGTGACCGTCTCCGGGAGCTGCATGTGTCAG

AGGTTTTCACCGTCATCACCGAAACGCGCGA

>pENTR2B/TREPitt::NLS/HA-HumBeta-P2A/Crimson

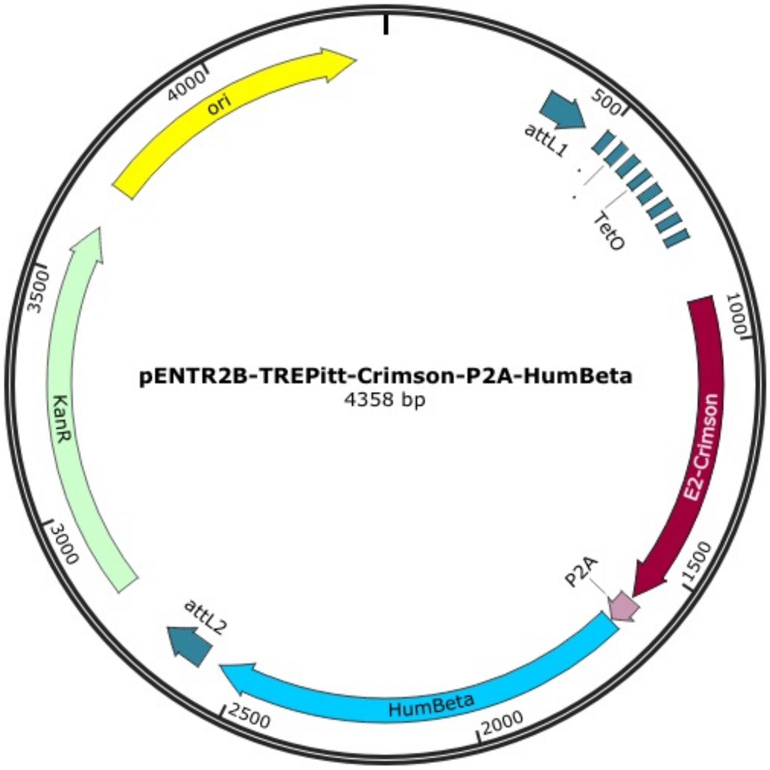

>pENTR2B/TREPitt::NLS/HA-HumBeta-P2A/Crimson

CTTTCCTGCGTTATCCCCTGATTCTGTGGATAACCGTATTACCGCTAGCATGG

ATCTCGGGGACGTCTAACTACTAAGCGAGAGTAGGGAACTGCCAGGCATCAA

ATAAAACGAAAGGCTCAGTCGGAAGACTGGGCCTTTCGTTTTATCTGTTGTTT

GTCGGTGAACGCTCTCCTGAGTAGGACAAATCCGCCGGGAGCGGATTTGAAC

GTTGTGAAGCAACGGCCCGGAGGGTGGCGGGCAGGACGCCCGCCATAAACT

GCCAGGCATCAAACTAAGCAGAAGGCCATCCTGACGGATGGCCTTTTTGCGT

TTCTACAAACTCTTCCTGTTAGTTAGTTACTTAAGCTCGGGCCCCAAATAATG

ATTTTATTTTGACTGATAGTGACCTGTTCGTTGCAACAAATTGATAAGCAATG

CTTTTTTATAATGCCAACTTTGTACAAAAAAGCAGGCTGGCGCCGGAACCAA

TTCAgatatcAAGTGCCACCTGACGTCTCCCTATCAGTGATAGAGAAGTCGACAC

GTCTCGAGCTCCCTATCAGTGATAGAGAAGGTACGTCTAGAACGTCTCCCTAT
CAGTGATAGAGAAGTCGACACGTCTCGAGCTCCCTATCAGTGATAGAGAAGG
TACGTCTAGAACGTCTCCCTATCAGTGATAGAGAAGTCGACACGTCTCGAGC
TCCCTATCAGTGATAGAGAAGGTACGTCTAGAACGTCTCCCTATCAGTGATA
GAGAAGTCGACACGTCTCGAGCTCCCTATCAGTGATAGAGAAGGTACCCCCT
ATATAAGCAGAGCTCGTTTAGTGAACCGTCAGATCGCCTGGAGACGCCATCC
ACGCTGTTTTGACCTCCATAGAAGACACCGGGACCGATCCAGCCTggatctGTAC
CACCATGGTGCCTCCCAAGAAGAAGAGGAAGGTCGAGGACCCCAAGTACCC
CTATGACGTGCCCGATTACGCTGGCGGAGGCGGAGGCTCCGGCGGAGGAGG
AGGATCCGGAGGAGGCAGCATGTCCACAGCTCTCGCCACCCTGGCCGGAAAA
CTGGCCGAGAGAGTGGGCATGGACTCCGTGGATCCCCAGGAGCTGATCACCA
CACTGAGGCAAACCGCCTTCAAGGGAGATGCTTCCGACGCCCAATTCATCGC
CCTCCTCATCGTGGCCAACCAGTACGGCCTGAACCCTTGGACCAAGGAGATT
TACGCCTTCCCCGACAAGCAGAATGGCATTGTCCCTGTGGTCGGCGTGGACG
GCTGGAGCAGAATCATTAACGAGAACCAGCAGTTTGACGGCATGGACTTCGA
GCAGGACAACGAGAGCTGCACATGCAGGATCTACAGGAAGGACAGGAACCA
TCCTATCTGCGTCACCGAGTGGATGGATGAGTGCAGAAGAGAACCCTTCAAG
ACCAGAGAGGGCAGAGAGATCACAGGACCCTGGCAGTCCCATCCTAAGAGA
ATGCTCAGACACAAGGCCATGATCCAGTGTGCCAGGCTCGCTTTTGGCTTCGC
CGGCATCTATGATAAGGACGAAGCCGAGAGGATCGTGGAAAATACCGCCTAC
ACCGCTGAAAGGCAGCCCGAAAGGGACATTACACCCGTGAACGACGAGACC
ATGCAGGAGATCAATACCCTGCTCATTGCCCTGGACAAAACCTGGGACGATG
ACCTCCTCCCCCTCTGCAGCCAAATCTTCAGGAGGGACATCAGGGCCAGCTC

CGAACTCACACAAGCCGAAGCTGTGAAGGCCCTCGGATTCCTCAAACAGAAG

GCCGCTGAGCAGAAGGTCGCCGCCGTTTCCGGATCCGGAGCCACGAACTTCT

CTCTGTTAAAGCAAGCAGGAGACGTGGAAGAAAACCCCGGTCCTATGGATAG

CACTGAGAACGTCATCAAGCCCTTCATGCGCTTCAAGGTGCACATGGAGGGC

TCCGTGAACGGCCACGAGTTCGAGATCGAGGGCGTGGGCGAGGGCAAGCCC

TACGAGGGCACCCAGACCGCCAAGCTGCAAGTGACCAAGGGCGGCCCCCTG

CCCTTCGCCTGGGACATCCTGTCCCCCCAGTTCTTCTACGGCTCCAAGGCGTA

CATCAAGCACCCCGCCGACATCCCCGACTACCTCAAGCAGTCCTTCCCCGAG

GGCTTCAAGTGGGAGCGCGTGATGAACTTCGAGGACGGCGGCGTGGTGACCG

TGACCCAGGACTCCTCCCTGCAGGACGGCACCCTCATCTACCACGTGAAGTT

CATCGGCGTGAACTTCCCCTCCGACGGCCCCGTAATGCAGAAGAAGACTCTG

GGCTGGGAGCCCTCCACTGAGCGCAACTACCCCCGCGACGGCGTGCTGAAGG

GCGAGAACCACATGGCGCTGAAGCTGAAGGGCGGCGGCCACTACCTGTGTG

AGTTCAAGTCCATCTACATGGCCAAGAAGCCCGTGAAGCTGCCCGGCTACCA

CTACGTGGACTACAAGCTCGACATCACCTCCCACAACGAGGACTACACCGTG

GTGGAGCAGTACGAGCGCGCCGAGGCCCGCCACCACCTGTTCCAGTAGAAAC

GAATTCGCGGCCGCACTCGAGATATCTAGACCCAGCTTTCTTGTACAAAGTTG

GCATTATAAGAAAGCATTGCTTATCAATTTGTTGCAACGAACAGGTCACTATC

AGTCAAAATAAAATCATTATTTGCCATCCAGCTGCAGCTCTGGCCCGTGTCTC

AAAATCTCTGATGTTACATTGCACAAGATAAAAATATATCATCATGAACAAT

AAAACTGTCTGCTTACATAAACAGTAATACAAGGGGTGTTATGAGCCATATT

CAACGGGAAACGTCGAGGCCGCGATTAAATTCCAACATGGATGCTGATTTAT

ATGGGTATAAATGGGCTCGCGATAATGTCGGGCAATCAGGTGCGACAATCTA

TCGCTTGTATGGGAAGCCCGATGCGCCAGAGTTGTTTCTGAAACATGGCAAA

GGTAGCGTTGCCAATGATGTTACAGATGAGATGGTCAGACTAAACTGGCTGA

CGGAATTTATGCCTCTTCCGACCATCAAGCATTTTATCCGTACTCCTGATGAT

GCATGGTTACTCACCACTGCGATCCCCGGAAAAACAGCATTCCAGGTATTAG

AAGAATATCCTGATTCAGGTGAAAATATTGTTGATGCGCTGGCAGTGTTCCTG

CGCCGGTTGCATTCGATTCCTGTTTGTAATTGTCCTTTTAACAGCGATCGCGT

ATTTCGTCTCGCTCAGGCGCAATCACGAATGAATAACGGTTTGGTTGATGCGA

GTGATTTTGATGACGAGCGTAATGGCTGGCCTGTTGAACAAGTCTGGAAAGA

AATGCATAAACTTTTGCCATTCTCACCGGATTCAGTCGTCACTCATGGTGATT

TCTCACTTGATAACCTTATTTTTGACGAGGGGAAATTAATAGGTTGTATTGAT

GTTGGACGAGTCGGAATCGCAGACCGATACCAGGATCTTGCCATCCTATGGA

ACTGCCTCGGTGAGTTTTCTCCTTCATTACAGAAACGGCTTTTTCAAAAATAT

GGTATTGATAATCCTGATATGAATAAATTGCAGTTTCATTTGATGCTCGATGA

GTTTTTCTAATCAGAATTGGTTAATTGGTTGTAACATTATTCAGATTGGGCCC

CGTTCCACTGAGCGTCAGACCCCGTAGAAAAGATCAAAGGATCTTCTTGAGA

TCCTTTTTTTCTGCGCGTAATCTGCTGCTTGCAAACAAAAAAACCACCGCTAC

CAGCGGTGGTTTGTTTGCCGGATCAAGAGCTACCAACTCTTTTTCCGAAGGTA

ACTGGCTTCAGCAGAGCGCAGATACCAAATACTGTTCTTCTAGTGTAGCCGTA

GTTAGGCCACCACTTCAAGAACTCTGTAGCACCGCCTACATACCTCGCTCTGC

TAATCCTGTTACCAGTGGCTGCTGCCAGTGGCGATAAGTCGTGTCTTACCGGG

TTGGACTCAAGACGATAGTTACCGGATAAGGCGCAGCGGTCGGGCTGAACGG

GGGGTTCGTGCACACAGCCCAGCTTGGAGCGAACGACCTACACCGAACTGAG

ATACCTACAGCGTGAGCTATGAGAAAGCGCCACGCTTCCCGAAGGGAGAAA

GGCGGACAGGTATCCGGTAAGCGGCAGGGTCGGAACAGGAGAGCGCACGAG

GGAGCTTCCAGGGGGAAACGCCTGGTATCTTTATAGTCCTGTCGGGTTTCGCC

ACCTCTGACTTGAGCGTCGATTTTTGTGATGCTCGTCAGGGGGGCGGAGCCTA

TGGAAAAACGCCAGCAACGCGGCCTTTTTACGGTTCCTGGCCTTTTGCTGGCC

TTTTGCTCACATGTT

>pSLIK/TREPitt::NLS/HA-HumBeta-P2A/Crimson

AKA 5-pSLIK-Zeo/TREPitt::NLS/HA-HumBeta-P2A/Crimson or pSLIK5. Sequence verified from 2738 to 4863 bp.

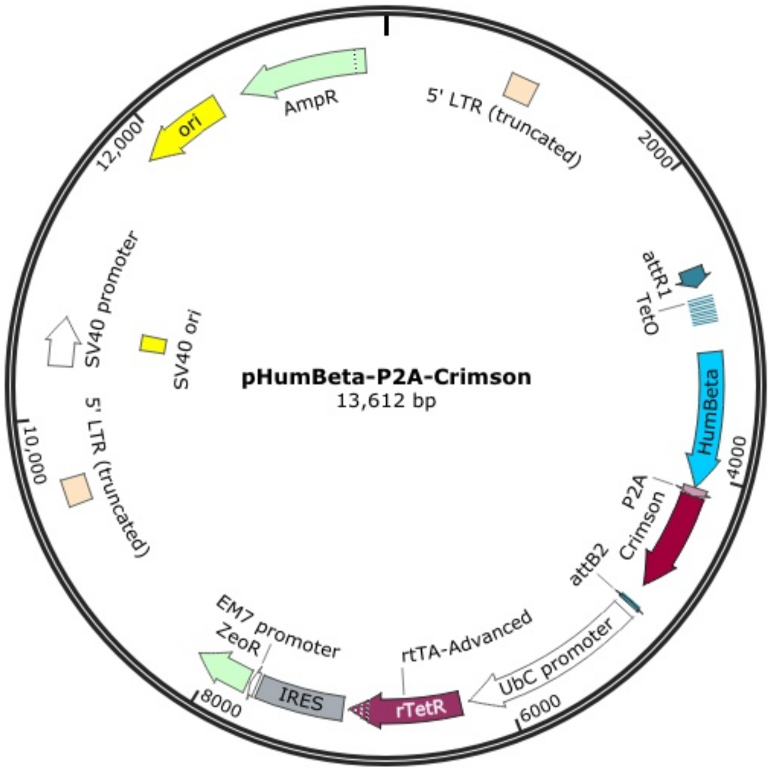

>5-pSLIK-Zeo/TREPitt::NLS/HA-HumBeta-P2A/Crimson

GTCGACGGATCGGGAGATCTCCCGATCCCCTATGGTGCACTCTCAGTACAAT

CTGCTCTGATGCCGCATAGTTAAGCCAGTATCTGCTCCCTGCTTGTGTGTTGG

AGGTCGCTGAGTAGTGCGCGAGCAAAATTTAAGCTACAACAAGGCAAGGCTT

GACCGACAATTGCATGAAGAATCTGCTTAGGGTTAGGCGTTTTGCGCTGCTTC

GCGATGTACGGGCCAGATATACGCGTTGACATTGATTATTGACTAGTTATTAA

TAGTAATCAATTACGGGGTCATTAGTTCATAGCCCATATATGGAGTTCCGCGT

TACATAACTTACGGTAAATGGCCCGCCTGGCTGACCGCCCAACGACCCCCGC

CCATTGACGTCAATAATGACGTATGTTCCCATAGTAACGCCAATAGGGACTTT

CCATTGACGTCAATGGGTGGAGTATTTACGGTAAACTGCCCACTTGGCAGTA

CATCAAGTGTATCATATGCCAAGTACGCCCCCTATTGACGTCAATGACGGTA

AATGGCCCGCCTGGCATTATGCCCAGTACATGACCTTATGGGACTTTCCTACT

TGGCAGTACATCTACGTATTAGTCATCGCTATTACCATGGTGATGCGGTTTTG

GCAGTACATCAATGGGCGTGGATAGCGGTTTGACTCACGGGGATTTCCAAGT

CTCCACCCCATTGACGTCAATGGGAGTTTGTTTTGGCACCAAAATCAACGGG

ACTTTCCAAAATGTCGTAACAACTCCGCCCCATTGACGCAAATGGGCGGTAG

GCGTGTACGGTGGGAGGTCTATATAAGCAGCGCGTTTTGCCTGTACTGGGTCT

CTCTGGTTAGACCAGATCTGAGCCTGGGAGCTCTCTGGCTAACTAGGGAACC

CACTGCTTAAGCCTCAATAAAGCTTGCCTTGAGTGCTTCAAGTAGTGTGTGCC

CGTCTGTTGTGTGACTCTGGTAACTAGAGATCCCTCAGACCCTTTTAGTCAGT

GTGGAAAATCTCTAGCAGTGGCGCCCGAACAGGGACTTGAAAGCGAAAGGG

AAACCAGAGGAGCTCTCTCGACGCAGGACTCGGCTTGCTGAAGCGCGCACGG

CAAGAGGCGAGGGGCGGCGACTGGTGAGTACGCCAAAAATTTTGACTAGCG

GAGGCTAGAAGGAGAGAGATGGGTGCGAGAGCGTCAGTATTAAGCGGGGGA

GAATTAGATCGCGATGGGAAAAAATTCGGTTAAGGCCAGGGGGAAAGAAAA

AATATAAATTAAAACATATAGTATGGGCAAGCAGGGAGCTAGAACGATTCGC

AGTTAATCCTGGCCTGTTAGAAACATCAGAAGGCTGTAGACAAATACTGGGA
CAGCTACAACCATCCCTTCAGACAGGATCAGAAGAACTTAGATCATTATATA
ATACAGTAGCAACCCTCTATTGTGTGCATCAAAGGATAGAGATAAAAGACAC
CAAGGAAGCTTTAGACAAGATAGAGGAAGAGCAAAACAAAAGTAAGACCAC
CGCACAGCAAGCGGCCGCTGATCTTCAGACCTGGAGGAGGAGATATGAGGG
ACAATTGGAGAAGTGAATTATATAAATATAAAGTAGTAAAAATTGAACCATT
AGGAGTAGCACCCACCAAGGCAAAGAGAAGAGTGGTGCAGAGAGAAAAAA
GAGCAGTGGGAATAGGAGCTTTGTTCCTTGGGTTCTTGGGAGCAGCAGGAAG
CACTATGGGCGCAGCGTCAATGACGCTGACGGTACAGGCCAGACAATTATTG
TCTGGTATAGTGCAGCAGCAGAACAATTTGCTGAGGGCTATTGAGGCGCAAC
AGCATCTGTTGCAACTCACAGTCTGGGGCATCAAGCAGCTCCAGGCAAGAAT
CCTGGCTGTGGAAAGATACCTAAAGGATCAACAGCTCCTGGGGATTTGGGGT
TGCTCTGGAAAACTCATTTGCACCACTGCTGTGCCTTGGAATGCTAGTTGGAG
TAATAAATCTCTGGAACAGATTTGGAATCACACGACCTGGATGGAGTGGGAC
AGAGAAATTAACAATTACACAAGCTTAATACACTCCTTAATTGAAGAATCGC
AAAACCAGCAAGAAAAGAATGAACAAGAATTATTGGAATTAGATAAATGGG
CAAGTTTGTGGAATTGGTTTAACATAACAAATTGGCTGTGGTATATAAAATTA
TTCATAATGATAGTAGGAGGCTTGGTAGGTTTAAGAATAGTTTTTGCTGTACT
TTCTATAGTGAATAGAGTTAGGCAGGGATATTCACCATTATCGTTTCAGACCC
ACCTCCCAACCCCGAGGGGACCCGACAGGCCCGAAGGAATAGAAGAAGAAG
GTGGAGAGAGAGACAGAGACAGATCCATTCGATTAGTGAACGGATCGGCAC
TGCGTGCGCCAATTCTGCAGACAAATGGCAGTATTCATCCACAATTTTAAAA
GAAAAGGGGGGATTGGGGGGTACAGTGCAGGGGAAAGAATAGTAGACATAA

TAGCAACAGACATACAAACTAAAGAATTACAAAAACAAATTACAAAAATTC

AAAATTTTCGGGTTTATTACAGGGACAGCAGAGATCCAGTTTGGTTAATTCTA

GACAGTTATCAACAAGTTTGTACAAAAAAGCTGAACGAGAAACGTAAAATG

ATATAAATATCAATATATTAAATTAGATTTTGCATAAAAAACAGACTACATA

ATACTGTAAAACACAACATATCCAGTCACTATGGCGCCGGAACCAATTCAG

ATATCAAGTGCCACCTGACGTCTCCCTATCAGTGATAGAGAAGTCGACACGT

CTCGAGCTCCCTATCAGTGATAGAGAAGGTACGTCTAGAACGTCTCCCTATCA

GTGATAGAGAAGTCGACACGTCTCGAGCTCCCTATCAGTGATAGAGAAGGTA

CGTCTAGAACGTCTCCCTATCAGTGATAGAGAAGTCGACACGTCTCGAGCTC

CCTATCAGTGATAGAGAAGGTACGTCTAGAACGTCTCCCTATCAGTGATAGA

GAAGTCGACACGTCTCGAGCTCCCTATCAGTGATAGAGAAGGTACCCCCTAT

ATAAGCAGAGCTCGTTTAGTGAACCGTCAGATCGCCTGGAGACGCCATCCAC

GCTGTTTTGACCTCCATAGAAGACACCGGGACCGATCCAGCCTGGATCTGTA

CCACCATGGTGCCTCCCAAGAAGAAGAGGAAGGTCGAGGACCCCAAGTACC

CCTATGACGTGCCCGATTACGCTGGCGGAGGCGGAGGCTCCGGCGGAGGAG

GAGGATCCGGAGGAGGCAGCATGTCCACAGCTCTCGCCACCCTGGCCGGAAA

ACTGGCCGAGAGAGTGGGCATGGACTCCGTGGATCCCCAGGAGCTGATCACC

ACACTGAGGCAAACCGCCTTCAAGGGAGATGCTTCCGACGCCCAATTCATCG

CCCTCCTCATCGTGGCCAACCAGTACGGCCTGAACCCTTGGACCAAGGAGAT

TTACGCCTTCCCCGACAAGCAGAATGGCATTGTCCCTGTGGTCGGCGTGGAC

GGCTGGAGCAGAATCATTAACGAGAACCAGCAGTTTGACGGCATGGACTTCG

AGCAGGACAACGAGAGCTGCACATGCAGGATCTACAGGAAGGACAGGAACC

ATCCTATCTGCGTCACCGAGTGGATGGATGAGTGCAGAAGAGAACCCTTCAA

GACCAGAGAGGGCAGAGAGATCACAGGACCCTGGCAGTCCCATCCTAAGAG
AATGCTCAGACACAAGGCCATGATCCAGTGTGCCAGGCTCGCTTTTGGCTTCG
CCGGCATCTATGATAAGGACGAAGCCGAGAGGATCGTGGAAAATACCGCCTA
CACCGCTGAAAGGCAGCCCGAAAGGGACATTACACCCGTGAACGACGAGAC
CATGCAGGAGATCAATACCCTGCTCATTGCCCTGGACAAAACCTGGGACGAT
GACCTCCTCCCCCTCTGCAGCCAAATCTTCAGGAGGGACATCAGGGCCAGCT
CCGAACTCACACAAGCCGAAGCTGTGAAGGCCCTCGGATTCCTCAAACAGAA
GGCCGCTGAGCAGAAGGTCGCCGCCGTTTCCGGATCCGGAGCCACGAACTTC
TCTCTGTTAAAGCAAGCAGGAGACGTGGAAGAAAACCCCGGTCCTATGGATA
GCACTGAGAACGTCATCAAGCCCTTCATGCGCTTCAAGGTGCACATGGAGGG
CTCCGTGAACGGCCACGAGTTCGAGATCGAGGGCGTGGGCGAGGGCAAGCC
CTACGAGGGCACCCAGACCGCCAAGCTGCAAGTGACCAAGGGCGGCCCCCT
GCCCTTCGCCTGGGACATCCTGTCCCCCCAGTTCTTCTACGGCTCCAAGGCGT
ACATCAAGCACCCCGCCGACATCCCCGACTACCTCAAGCAGTCCTTCCCCGA
GGGCTTCAAGTGGGAGCGCGTGATGAACTTCGAGGACGGCGGCGTGGTGACC
GTGACCCAGGACTCCTCCCTGCAGGACGGCACCCTCATCTACCACGTGAAGT
TCATCGGCGTGAACTTCCCCTCCGACGGCCCCGTAATGCAGAAGAAGACTCT
GGGCTGGGAGCCCTCCACTGAGCGCAACTACCCCCGCGACGGCGTGCTGAAG
GGCGAGAACCACATGGCGCTGAAGCTGAAGGGCGGCGGCCACTACCTGTGT
GAGTTCAAGTCCATCTACATGGCCAAGAAGCCCGTGAAGCTGCCCGGCTACC
ACTACGTGGACTACAAGCTCGACATCACCTCCCACAACGAGGACTACACCGT
GGTGGAGCAGTACGAGCGCGCCGAGGCCCGCCACCACCTGTTCCAGTAGAAA
CGAATTCGCGGCCGCACTCGAGATATCTAGATAGTGACTGGATATGTTGTGTT

TTACAGTATTATGTAGTCTGTTTTTATGCAAAATCTAATTTAATATATTGATA

TTTATATCATTTTACGTTTCTCGTTCAGCTTTCTTGTACAAAGTGGTTGATAAC

ATCTCGAGCCATAAAGATGGTTAATTAACCCACCCAAGATCTGGCCTCCGCG

CCGGGTTTTGGCGCCTCCCGCGGGCGCCCCCCTCCTCACGGCGAGCGCTGCC

ACGTCAGACGAAGGGCGCAGCGAGCGTCCTGATCCTTCCGCCCGGACGCTCA

GGACAGCGGCCCGCTGCTCATAAGACTCGGCCTTAGAACCCCAGTATCAGCA

GAAGGACATTTTAGGACGGGACTTGGGTGACTCTAGGGCACTGGTTTTCTTTC

CAGAGAGCGGAACAGGCGAGGAAAAGTAGTCCCTTCTCGGCGATTCTGCGGA

GGGATCTCCGTGGGGCGGTGAACGCCGATGATTATATAAGGACGCGCCGGGT

GTGGCACAGCTAGTTCCGTCGCAGCCGGGATTTGGGTCGCGGTTCTTGTTTGT

GGATCGCTGTGATCGTCACTTGGTGAGTAGCGGGCTGCTGGGCTGGCCGGGG

CTTTCGTGGCCGCCGGGCCGCTCGGTGGGACGGAAGCGTGTGGAGAGACCGC

CAAGGGCTGTAGTCTGGGTCCGCGAGCAAGGTTGCCCTGAACTGGGGGTTGG

GGGGAGCGCAGCAAAATGGCGGCTGTTCCCGAGTCTTGAATGGAAGACGCTT

GTGAGGCGGGCTGTGAGGTCGTTGAAACAAGGTGGGGGGCATGGTGGGCGG

CAAGAACCCAAGGTCTTGAGGCCTTCGCTAATGCGGGAAAGCTCTTATTCGG

GTGAGATGGGCTGGGGCACCATCTGGGGACCCTGACGTGAAGTTTGTCACTG

ACTGGAGAACTCGGTTTGTCGTCTGTTGCGGGGGCGGCAGTTATGGCGGTGC

CGTTGGGCAGTGCACCCGTACCTTTGGGAGCGCGCGCCCTCGTCGTGTCGTG

ACGTCACCCGTTCTGTTGGCTTATAATGCAGGGTGGGGCCACCTGCCGGTAG

GTGTGCGGTAGGCTTTTCTCCGTCGCAGGACGCAGGGTTCGGGCCTAGGGTA

GGCTCTCCTGAATCGACAGGCGCCGGACCTCTGGTGAGGGGAGGGATAAGTG

AGGCGTCAGTTTCTTTGGTCGGTTTTATGTACCTATCTTCTTAAGTAGCTGAA

GCTCCGGTTTTGAACTATGCGCTCGGGGTTGGCGAGTGTGTTTTGTGAAGTTT

TTTAGGCACCTTTTGAAATGTAATCATTTGGGTCAATATGTAATTTTCAGTGTT

AGACTAGTAAATTGTCCGCTAAATTCTGGCCGTTTTTGGCTTTTTTGTTAGAC

GAAGCTTGGTACCGAGCTCGGATCTCCACCCCGTACCGGTCCTGCAGTCGAA

TTCACCATGTCTAGACTGGACAAGAGCAAAGTCATAAACGGAGCTCTGGAAT

TACTCAATGGTGTCGGTATCGAAGGCCTGACGACAAGGAAACTCGCTCAAAA

GCTGGGAGTTGAGCAGCCTACCCTGTACTGGCACGTGAAGAACAAGCGGGCC

CTGCTCGATGCCCTGCCAATCGAGATGCTGGACAGGCATCATACCCACTTCTG

CCCCCTGGAAGGCGAGTCATGGCAAGACTTTCTGCGGAACAACGCCAAGTCA

TACCGCTGTGCTCTCCTCTCACATCGCGACGGGGCTAAAGTGCATCTCGGCAC

CCGCCCAACAGAGAAACAGTACGAAACCCTGGAAAATCAGCTCGCGTTCCTG

TGTCAGCAAGGCTTCTCCCTGGAGAACGCACTGTACGCTCTGTCCGCCGTGG

GCCACTTTACACTGGGCTGCGTATTGGAGGAACAGGAGCATCAAGTAGCAAA

AGAGGAAAGAGAGACACCTACCACCGATTCTATGCCCCCACTTCTGAGACAA

GCAATTGAGCTGTTCGACCGGCAGGGAGCCGAACCTGCCTTCCTTTTCGGCCT

GGAACTAATCATATGTGGCCTGGAGAAACAGCTAAAGTGCGAAAGCGGCGG

GCCGACCGACGCCCTTGACGATTTTGACTTAGACATGCTCCCAGCCGATGCCC

TTGACGACTTTGACCTTGATATGCTGCCTGCTGACGCTCTTGACGATTTTGAC

CTTGACATGCTCCCCGGGTAACTAAGTAAGGATCCGCGGCCGCACTAGAGGA

ATTCCGCCCCTCTCCCTCCCCCCCCCCTAACGTTACTGGCCGAAGCCGCTTGG

AATAAGGCCGGTGTGTGTTTGTCTATATGTTATTTTCCACCATATTGCCGTCTT

TTGGCAATGTGAGGGCCCGGAAACCTGGCCCTGTCTTCTTGACGAGCATTCCT

AGGGGTCTTTCCCCTCTCGCCAAAGGAATGCAAGGTCTGTTGAATGTCGTGA

AGGAAGCAGTTCCTCTGGAAGCTTCTTGAAGACAAACAACGTCTGTAGCGAC
CCTTTGCAGGCAGCGGAACCCCCCACCTGGCGACAGGTGCCTCTGCGGCCAA
AAGCCACGTGTATAAGATACACCTGCAAAGGCGGCACAACCCCAGTGCCACG
TTGTGAGTTGGATAGTTGTGGAAAGAGTCAAATGGCTCTCCTCAAGCGTAGT
CAACAAGGGGCTGAAGGATGCCCAGAAGGTACCCCATTGTATGGGAATCTGA
TCTGGGGCCTCGGTGCACATGCTTTACATGTGTTTAGTCGAGGTTAAAAAAC
GTCTAGGCCCCCCGAACCACGGGGACGTGGTTTTCCTTTGAAAAACACGATG
ATAAGCTTACCGGTACGCGTGATGTGTTGACAATTAATCATCGGCATAGTATA
TCGGCATAGTATAATACGACAAGGTGAGGAACTAAACCATGGCCAAGTTGAC
CAGTGCCGTTCCGGTGCTCACCGCGCGCGACGTCGCCGGAGCGGTCGAGTTC
TGGACCGACCGGCTCGGGTTCTCCCGGGACTTCGTGGAGGACGACTTCGCCG
GTGTGGTCCGGGACGACGTGACCCTGTTCATCAGCGCGGTCCAGGACCAGGT
GGTGCCGGACAACACCCTGGCCTGGGTGTGGGTGCGCGGCCTGGACGAGCTG
TACGCCGAGTGGTCGGAGGTCGTGTCCACGAACTTCCGGGACGCCTCCGGGC
CGGCCATGACCGAGATCGGCGAGCAGCCGTGGGGGCGGGAGTTCGCCCTGC
GCGACCCGGCCGGCAACTGCGTGCACTTCGTGGCCGAGGAGCAGGACTGACA
CATCTGTACAAGTAAAGCGGCCGCGACTCTAGATCATAATCAGCCATACCAC
ATTTGTAGAGGTTTTACTTGCTTTAAAAAACCTCCCACACCTCCCCCTGAACC
TGAAACATAAAATGAATGCAATTGTTGTTGTTTAGTCCCTCCCAATTCGATAT
CAAGCTTATCGATAATCAACCTCTGGATTACAAAATTTGTGAAAGATTGACTG
GTATTCTTAACTATGTTGCTCCTTTTACGCTATGTGGATACGCTGCTTTAATGC
CTTTGTATCATGCTATTGCTTCCCGTATGGCTTTCATTTTCTCCTCCTTGTATA
AATCCTGGTTGCTGTCTCTTTATGAGGAGTTGTGGCCCGTTGTCAGGCAACGT

GGCGTGGTGTGCACTGTGTTTGCTGACGCAACCCCCACTGGTTGGGGCATTGC
CACCACCTGTCAGCTCCTTTCCGGGACTTTCGCTTTCCCCCTCCCTATTGCCAC
GGCGGAACTCATCGCCGCCTGCCTTGCCCGCTGCTGGACAGGGGCTCGGCTG
TTGGGCACTGACAATTCCGTGGTGTTGTCGGGGAAATCATCGTCCTTTCCTTG
GCTGCTCGCCTGTGTTGCCACCTGGATTCTGCGCGGGACGTCCTTCTGCTACG
TCCCTTCGGCCCTCAATCCAGCGGACCTTCCTTCCCGCGGCCTGCTGCCGGCT
CTGCGGCCTCTTCCGCGTCTTCGCCTTCGCCCTCAGACGAGTCGGATCTCCCT
TTGGGCCGCCTCCCCGCATCGATACCGTCGACCTCGATCGAGACCTAGAAAA
ACATGGAGCAATCACAAGTAGCAATACAGCAGCTACCAATGCTGATTGTGCC
TGGCTAGAAGCACAAGAGGAGGAGGAGGTGGGTTTTCCAGTCACACCTCAGG
TACCTTTAAGACCAATGACTTACAAGGCAGCTGTAGATCTTAGCCACTTTTTA
AAAGAAAAGGGGGGACTGGAAGGGCTAATTCACTCCCAACGAAGACAAGAT
ATCCTTGATCTGTGGATCTACCACACACAAGGCTACTTCCCTGATTGGCAGAA
CTACACACCAGGGCCAGGGATCAGATATCCACTGACCTTTGGATGGTGCTAC
AAGCTAGTACCAGTTGAGCAAGAGAAGGTAGAAGAAGCCAATGAAGGAGAG
AACACCCGCTTGTTACACCCTGTGAGCCTGCATGGGATGGATGACCCGGAGA
GAGAAGTATTAGAGTGGAGGTTTGACAGCCGCCTAGCATTTCATCACATGGC
CCGAGAGCTGCATCCGGACTGTACTGGGTCTCTCTGGTTAGACCAGATCTGA
GCCTGGGAGCTCTCTGGCTAACTAGGGAACCCACTGCTTAAGCCTCAATAAA
GCTTGCCTTGAGTGCTTCAAGTAGTGTGTGCCCGTCTGTTGTGTGACTCTGGT
AACTAGAGATCCCTCAGACCCTTTTAGTCAGTGTGGAAAATCTCTAGCAGGG
CCCGTTTAAACCCGCTGATCAGCCTCGACTGTGCCTTCTAGTTGCCAGCCATC
TGTTGTTTGCCCCTCCCCCGTGCCTTCCTTGACCCTGGAAGGTGCCACTCCCA

CTGTCCTTTCCTAATAAAATGAGGAAATTGCATCGCATTGTCTGAGTAGGTGT

CATTCTATTCTGGGGGGTGGGGTGGGGCAGGACAGCAAGGGGGAGGATTGG

GAAGACAATAGCAGGCATGCTGGGGATGCGGTGGGCTCTATGGCTTCTGAGG

CGGAAAGAACCAGCTGGGGCTCTAGGGGGTATCCCCACGCGCCCTGTAGCGG

CGCATTAAGCGCGGCGGGTGTGGTGGTTACGCGCAGCGTGACCGCTACACTT

GCCAGCGCCCTAGCGCCCGCTCCTTTCGCTTTCTTCCCTTCCTTTCTCGCCACG

TTCGCCGGCTTTCCCCGTCAAGCTCTAAATCGGGGGCTCCCTTTAGGGTTCCG

ATTTAGTGCTTTACGGCACCTCGACCCCAAAAAACTTGATTAGGGTGATGGTT

CACGTAGTGGGCCATCGCCCTGATAGACGGTTTTTCGCCCTTTGACGTTGGAG

TCCACGTTCTTTAATAGTGGACTCTTGTTCCAAACTGGAACAACACTCAACCC

TATCTCGGTCTATTCTTTTGATTTATAAGGGATTTTGCCGATTTCGGCCTATTG

GTTAAAAAATGAGCTGATTTAACAAAAATTTAACGCGAATTAATTCTGTGGA

ATGTGTGTCAGTTAGGGTGTGGAAAGTCCCCAGGCTCCCCAGCAGGCAGAAG

TATGCAAAGCATGCATCTCAATTAGTCAGCAACCAGGTGTGGAAAGTCCCCA

GGCTCCCCAGCAGGCAGAAGTATGCAAAGCATGCATCTCAATTAGTCAGCAA

CCATAGTCCCGCCCCTAACTCCGCCCATCCCGCCCCTAACTCCGCCCAGTTCC

GCCCATTCTCCGCCCCATGGCTGACTAATTTTTTTTATTTATGCAGAGGCCGA

GGCCGCCTCTGCCTCTGAGCTATTCCAGAAGTAGTGAGGAGGCTTTTTTGGAG

GCCTAGGCTTTTGCAAAAAGCTCCCGGGAGCTTGTATATCCATTTTCGGATCT

GATCAGCACGTGTTGACAATTAATCATCGGCATAGTATATCGGCATAGTATA

ATACGACAAGGTGAGGAACTAAACCATGGCCAAGTTGACCAGTGCCGTTCCG

GTGCTCACCGCGCGCGACGTCGCCGGAGCGGTCGAGTTCTGGACCGACCGGC

TCGGGTTCTCCCGGGACTTCGTGGAGGACGACTTCGCCGGTGTGGTCCGGGA

CGACGTGACCCTGTTCATCAGCGCGGTCCAGGACCAGGTGGTGCCGGACAAC

ACCCTGGCCTGGGTGTGGGTGCGCGGCCTGGACGAGCTGTACGCCGAGTGGT

CGGAGGTCGTGTCCACGAACTTCCGGGACGCCTCCGGGCCGGCCATGACCGA

GATCGGCGAGCAGCCGTGGGGGCGGGAGTTCGCCCTGCGCGACCCGGCCGG

CAACTGCGTGCACTTCGTGGCCGAGGAGCAGGACTGACACGTGCTACGAGAT

TTCGATTCCACCGCCGCCTTCTATGAAAGGTTGGGCTTCGGAATCGTTTTCCG

GGACGCCGGCTGGATGATCCTCCAGCGCGGGGATCTCATGCTGGAGTTCTTC

GCCCACCCCAACTTGTTTATTGCAGCTTATAATGGTTACAAATAAAGCAATAG

CATCACAAATTTCACAAATAAAGCATTTTTTTCACTGCATTCTAGTTGTGGTTT

GTCCAAACTCATCAATGTATCTTATCATGTCTGTATACCGTCGACCTCTAGCT

AGAGCTTGGCGTAATCATGGTCATAGCTGTTTCCTGTGTGAAATTGTTATCCG

CTCACAATTCCACACAACATACGAGCCGGAAGCATAAAGTGTAAAGCCTGGG

GTGCCTAATGAGTGAGCTAACTCACATTAATTGCGTTGCGCTCACTGCCCGCT

TTCCAGTCGGGAAACCTGTCGTGCCAGCTGCATTAATGAATCGGCCAACGCG

CGGGGAGAGGCGGTTTGCGTATTGGGCGCTCTTCCGCTTCCTCGCTCACTGAC

TCGCTGCGCTCGGTCGTTCGGCTGCGGCGAGCGGTATCAGCTCACTCAAAGG

CGGTAATACGGTTATCCACAGAATCAGGGGATAACGCAGGAAAGAACATGT

GAGCAAAAGGCCAGCAAAAGGCCAGGAACCGTAAAAAGGCCGCGTTGCTGG

CGTTTTTCCATAGGCTCCGCCCCCCTGACGAGCATCACAAAAATCGACGCTCA

AGTCAGAGGTGGCGAAACCCGACAGGACTATAAAGATACCAGGCGTTTCCCC

CTGGAAGCTCCCTCGTGCGCTCTCCTGTTCCGACCCTGCCGCTTACCGGATAC

CTGTCCGCCTTTCTCCCTTCGGGAAGCGTGGCGCTTTCTCATAGCTCACGCTG

TAGGTATCTCAGTTCGGTGTAGGTCGTTCGCTCCAAGCTGGGCTGTGTGCACG

AACCCCCCGTTCAGCCCGACCGCTGCGCCTTATCCGGTAACTATCGTCTTGAG

TCCAACCCGGTAAGACACGACTTATCGCCACTGGCAGCAGCCACTGGTAACA

GGATTAGCAGAGCGAGGTATGTAGGCGGTGCTACAGAGTTCTTGAAGTGGTG

GCCTAACTACGGCTACACTAGAAGAACAGTATTTGGTATCTGCGCTCTGCTGA

AGCCAGTTACCTTCGGAAAAAGAGTTGGTAGCTCTTGATCCGGCAAACAAAC

CACCGCTGGTAGCGGTGGTTTTTTTGTTTGCAAGCAGCAGATTACGCGCAGAA

AAAAAGGATCTCAAGAAGATCCTTTGATCTTTTCTACGGGGTCTGACGCTCAG

TGGAACGAAAACTCACGTTAAGGGATTTTGGTCATGAGATTATCAAAAAGGA

TCTTCACCTAGATCCTTTTAAATTAAAAATGAAGTTTTAAATCAATCTAAAGT

ATATATGAGTAAACTTGGTCTGACAGTTACCAATGCTTAATCAGTGAGGCAC

CTATCTCAGCGATCTGTCTATTTCGTTCATCCATAGTTGCCTGACTCCCCGTCG

TGTAGATAACTACGATACGGGAGGGCTTACCATCTGGCCCCAGTGCTGCAAT

GATACCGCGAGACCCACGCTCACCGGCTCCAGATTTATCAGCAATAAACCAG

CCAGCCGGAAGGGCCGAGCGCAGAAGTGGTCCTGCAACTTTATCCGCCTCCA

TCCAGTCTATTAATTGTTGCCGGGAAGCTAGAGTAAGTAGTTCGCCAGTTAAT

AGTTTGCGCAACGTTGTTGCCATTGCTACAGGCATCGTGGTGTCACGCTCGTC

GTTTGGTATGGCTTCATTCAGCTCCGGTTCCCAACGATCAAGGCGAGTTACAT

GATCCCCCATGTTGTGCAAAAAAGCGGTTAGCTCCTTCGGTCCTCCGATCGTT

GTCAGAAGTAAGTTGGCCGCAGTGTTATCACTCATGGTTATGGCAGCACTGC

ATAATTCTCTTACTGTCATGCCATCCGTAAGATGCTTTTCTGTGACTGGTGAG

TACTCAACCAAGTCATTCTGAGAATAGTGTATGCGGCGACCGAGTTGCTCTTG

CCCGGCGTCAATACGGGATAATACCGCGCCACATAGCAGAACTTTAAAAGTG

CTCATCATTGGAAAACGTTCTTCGGGGCGAAAACTCTCAAGGATCTTACCGCT

GTTGAGATCCAGTTCGATGTAACCCACTCGTGCACCCAACTGATCTTCAGCAT

CTTTTACTTTCACCAGCGTTTCTGGGTGAGCAAAAACAGGAAGGCAAAATGC

CGCAAAAAAGGGAATAAGGGCGACACGGAAATGTTGAATACTCATACTCTTC

CTTTTTCAATATTATTGAAGCATTTATCAGGGTTATTGTCTCATGAGCGGATA

CATATTTGAATGTATTTAGAAAAATAAACAAATAGGGGTTCCGCGCACATTT

CCCCGAAAAGTGCCACCTGAC

Plasmids used in construction of pSLIK6

>pFN24K::P2A/Crimson-control

AKA pFN24K::Product C-Control.

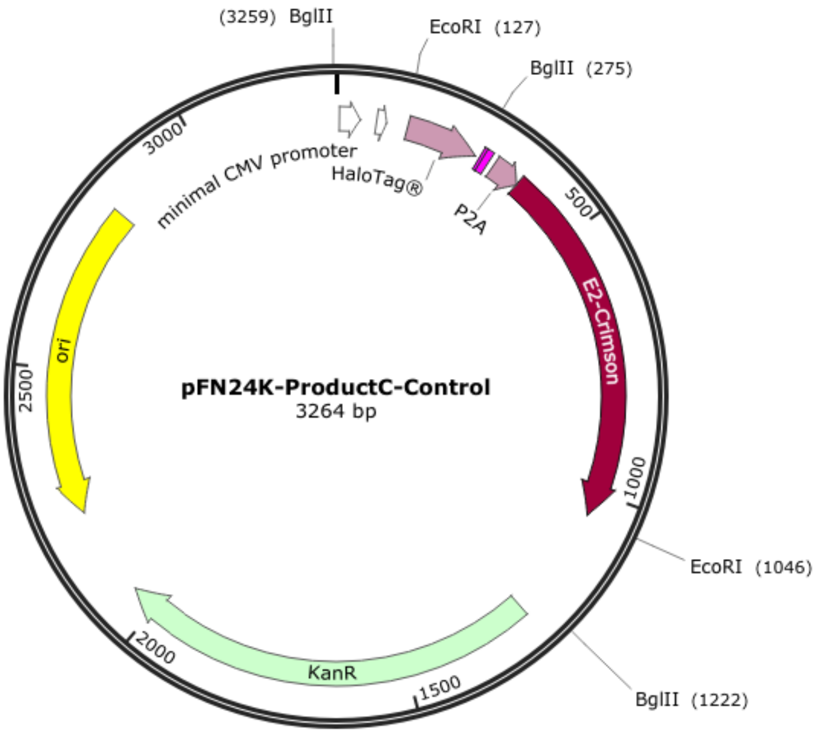

>pFN24K::P2A/Crimson-control

ATGGGCGGTAGGCGTGTACGGTGGGAGGTCTATATAAGCAGAGCTGGTTTAG

TGAACCGTCAGATCACTAGAAGCTTAATACGACTCACTATAGGGTACGATTT

AGGTGACACTATAGAATAAGGAGAATTCATCATGGCAGAAATCGGTACTGGC
TTTCCATTCGACCCCCATTATGTGGAAGTCCTGGGCGAGCGCATGCACTACGT
CGATGTTGGTCCGCGCGATGGCACCCCTGTGCTGTTCCTGCACGGTAACCCGA
CCTCCTCCTACCAGATCTAGGAGGTCCGCCACCATGGCCGGATCCGGAGCCA
CGAACTTCTCTCTGTTAAAGCAAGCAGGAGACGTGGAAGAAAACCCCGGTCC
TATGGATAGCACTGAGAACGTCATCAAGCCCTTCATGCGCTTCAAGGTGCAC
ATGGAGGGCTCCGTGAACGGCCACGAGTTCGAGATCGAGGGCGTGGGCGAG
GGCAAGCCCTACGAGGGCACCCAGACCGCCAAGCTGCAAGTGACCAAGGGC
GGCCCCCTGCCCTTCGCCTGGGACATCCTGTCCCCCCAGTTCTTCTACGGCTC
CAAGGCGTACATCAAGCACCCCGCCGACATCCCCGACTACCTCAAGCAGTCC
TTCCCCGAGGGCTTCAAGTGGGAGCGCGTGATGAACTTCGAGGACGGCGGCG
TGGTGACCGTGACCCAGGACTCCTCCCTGCAGGACGGCACCCTCATCTACCA
CGTGAAGTTCATCGGCGTGAACTTCCCCTCCGACGGCCCCGTAATGCAGAAG
AAGACTCTGGGCTGGGAGCCCTCCACTGAGCGCAACTACCCCCGCGACGGCG
TGCTGAAGGGCGAGAACCACATGGCGCTGAAGCTGAAGGGCGGCGGCCACT
ACCTGTGTGAGTTCAAGTCCATCTACATGGCCAAGAAGCCCGTGAAGCTGCC
CGGCTACCACTACGTGGACTACAAGCTCGACATCACCTCCCACAACGAGGAC
TACACCGTGGTGGAGCAGTACGAGCGCGCCGAGGCCCGCCACCACCTGTTCC
AGTAGGAATTCGCGGCCGCAGTGTTCCGCTTCCTTTAGCAGCCCTTGCGCCCT
GAGTGCTTGCGGCAGCGTGAGCTTCAAAAGAATTGCCAGCTGGGGCGCCCTC
TGGTAAGGTTGGGAAGCCCTGCAAAGTAAACTGGATGGCTTTCTTGCCGCCA
AGGATCTGATGGCGCAGGGGATCAAGATCTGATCAAGAGACAGGATGACGG
TCGTTTCGCATGCTTGAACAAGATGGATTGCACGCAGGTTCTCCGGCCGCTTG

GGTGGAGAGGCTATTCGGCTATGACTGGGCACAACAGACAATCGGCTGCTCT
GATGCCGCCGTGTTCCGGCTGTCAGCGCAGGGGCGCCCGGTTCTTTTTGTCAA
GACCGACCTGTCCGGTGCCCTGAATGAACTGCAGGACGAGGCAGCGCGGCTA
TCGTGGCTGGCCACGACGGGCGTTCCTTGCGCAGCTGTGCTCGACGTTGTCAC
TGAAGCGGGAAGGGACTGGCTGCTATTGGGCGAAGTGCCGGGGCAGGATCTC
CTGTCATCTCACCTTGCTCCTGCCGAGAAAGTATCCATCATGGCTGATGCAAT
GCGGCGGCTGCATACGCTTGATCCGGCTACCTGCCCATTCGACCACCAAGCG
AAACATCGCATCGAGCGAGCACGCACTCGGATGGAAGCCGGTCTTGTCGATC
AGGATGATCTGGACGAAGAGCATCAGGGGCTCGCGCCAGCCGAACTGTTCGC
CAGGCTCAAGGCGCGTATGCCGGATGGTGAGGATCTCGTCGTGACTCATGGC
GATGCCTGCTTGCCGAATATCATGGTGGAAAATGGCCGCTTTTCTGGATTCAT
CGACTGTGGCCGGCTGGGTGTGGCGGACCGCTATCAGGACATAGCGTTGGCT
ACCCGTGATATTGCTGAAGAGCTTGGCGGCGAATGGGCTGACCGCTTCCTCG
TGCTTTACGGTATCGCCGCTCCCGATTCGCAGCGCATCGCCTTCTATCGCCTT
CTTGACGAGTTCTTCTGAGCGGGACTCTGGGGTTCGAAATGACCGACCAAGC
GACGCCCAACCGGTATCAGCTCACTCAAAGGCGGTAATACGGTTATCCACAG
AATCAGGGGATAACGCAGGAAAGAACATGTGAGCAAAAGGCCAGCAAAAGG
CCAGGAACCGTAAAAAGGCCGCGTTGCTGGCGTTTTTCCATAGGCTCCGCCC
CCCTGACGAGCATCACAAAAATCGACGCTCAAGTCAGAGGTGGCGAAACCC
GACAGGACTATAAAGATACCAGGCGTTTCCCCCTGGAAGCTCCCTCGTGCGC
TCTCCTGTTCCGACCCTGCCGCTTACCGGATACCTGTCCGCCTTTCTCCCTTCG
GGAAGCGTGGCGCTTTCTCATAGCTCACGCTGTAGGTATCTCAGTTCGGTGTA
GGTCGTTCGCTCCAAGCTGGGCTGTGTGCACGAACCCCCCGTTCAGCCCGAC

CGCTGCGCCTTATCCGGTAACTATCGTCTTGAGTCCAACCCGGTAAGACACG

ACTTATCGCCACTGGCAGCAGCCACTGGTAACAGGATTAGCAGAGCGAGGTA

TGTAGGCGGTGCTACAGAGTTCTTGAAGTGGTGGCCTAACTACGGCTACACT

AGAAGGACAGTATTTGGTATCTGCGCTCTGCTGAAGCCAGTTACCTTCGGAA

AAAGAGTTGGTAGCTCTTGATCCGGCAAACAAACCACCGCTGGTAGCGGTGG

TTTTTTTGTTTGCAAGCAGCAGATTACGCGCAGAAAAAAAGGATTTCAAGAA

GATCCTTTGATCTTTTCTACGGGGTCTGACGCTCAGTGGAACGAAAACTCACG

TTAAGGGATTTTGGTCATGAGATTATCAAAAAGGATCTTCACCTAGATCCTTT

TATAGTCCGGAAATACAGGAACGCACGCTGGATGGCCCTTCGCTGGGATGGT

GAAACCATGAAAAATGGCAGCTTCAGTGGATTAAGTGGGGGTAATGTGGCCT

GTACCCTCTGGTTGCATAGGTATTCATACGGTTAAAATTTATCAGGCGCGATT

GCGGCAGTTTTTCGGGTGGTTTGTTGCCATTTTTACCTGTCTGCTGCCGTGATC

GCGCTGAACGCGTTTTAGCGGTGCGTACAATTAAGGGATTATGGTAAATCCA

CTTACTGTCTGCCCTCGTAGCCATCGAGATAAACCGCAGTACTCCGGCCACG

ATGCGTCCGGCGTAGAGGATCGAGATCT

>pENTR2B/TREPitt::P2A/Crimson-control

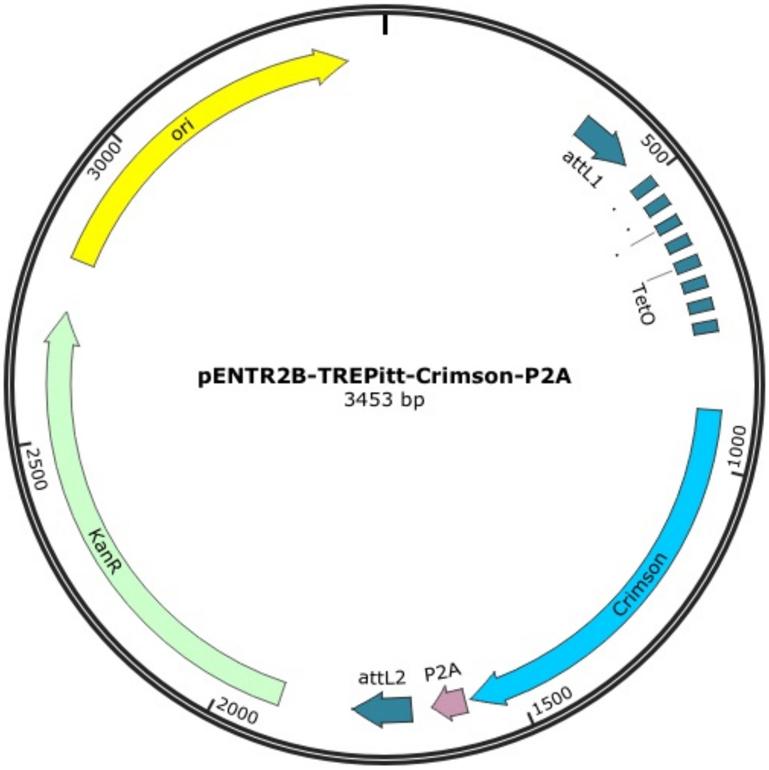

> pENTR2B/TREPitt::P2A/Crimson-control

CTTTCCTGCGTTATCCCCTGATTCTGTGGATAACCGTATTACCGCTAGCATGG

ATCTCGGGGACGTCTAACTACTAAGCGAGAGTAGGGAACTGCCAGGCATCAA

ATAAAACGAAAGGCTCAGTCGGAAGACTGGGCCTTTCGTTTTATCTGTTGTTT

GTCGGTGAACGCTCTCCTGAGTAGGACAAATCCGCCGGGAGCGGATTTGAAC

GTTGTGAAGCAACGGCCCGGAGGGTGGCGGGCAGGACGCCCGCCATAAACT

GCCAGGCATCAAACTAAGCAGAAGGCCATCCTGACGGATGGCCTTTTTGCGT

TTCTACAAACTCTTCCTGTTAGTTAGTTACTTAAGCTCGGGCCCCAAATAATG

ATTTTATTTTGACTGATAGTGACCTGTTCGTTGCAACAAATTGATAAGCAATG

CTTTTTTATAATGCCAACTTTGTACAAAAAAGCAGGCTGGCGCCGGAACCAA

TTCAGATATCAAGTGCCACCTGACGTCTCCCTATCAGTGATAGAGAAGTCGA

CACGTCTCGAGCTCCCTATCAGTGATAGAGAAGGTACGTCTAGAACGTCTCC
CTATCAGTGATAGAGAAGTCGACACGTCTCGAGCTCCCTATCAGTGATAGAG
AAGGTACGTCTAGAACGTCTCCCTATCAGTGATAGAGAAGTCGACACGTCTC
GAGCTCCCTATCAGTGATAGAGAAGGTACGTCTAGAACGTCTCCCTATCAGT
GATAGAGAAGTCGACACGTCTCGAGCTCCCTATCAGTGATAGAGAAGGTACC
CCCTATATAAGCAGAGCTCGTTTAGTGAACCGTCAGATCGCCTGGAGACGCC
ATCCACGCTGTTTTGACCTCCATAGAAGACACCGGGACCGATCCAGCCTGGA
TCTAGGAGGTCCGCCACCATGGCCGGATCCGGAGCCACGAACTTCTCTCTGTT
AAAGCAAGCAGGAGACGTGGAAGAAAACCCCGGTCCTATGGATAGCACTGA
GAACGTCATCAAGCCCTTCATGCGCTTCAAGGTGCACATGGAGGGCTCCGTG
AACGGCCACGAGTTCGAGATCGAGGGCGTGGGCGAGGGCAAGCCCTACGAG
GGCACCCAGACCGCCAAGCTGCAAGTGACCAAGGGCGGCCCCCTGCCCTTCG
CCTGGGACATCCTGTCCCCCCAGTTCTTCTACGGCTCCAAGGCGTACATCAAG
CACCCCGCCGACATCCCCGACTACCTCAAGCAGTCCTTCCCCGAGGGCTTCA
AGTGGGAGCGCGTGATGAACTTCGAGGACGGCGGCGTGGTGACCGTGACCCA
GGACTCCTCCCTGCAGGACGGCACCCTCATCTACCACGTGAAGTTCATCGGC
GTGAACTTCCCCTCCGACGGCCCCGTAATGCAGAAGAAGACTCTGGGCTGGG
AGCCCTCCACTGAGCGCAACTACCCCCGCGACGGCGTGCTGAAGGGCGAGAA
CCACATGGCGCTGAAGCTGAAGGGCGGCGGCCACTACCTGTGTGAGTTCAAG
TCCATCTACATGGCCAAGAAGCCCGTGAAGCTGCCCGGCTACCACTACGTGG
ACTACAAGCTCGACATCACCTCCCACAACGAGGACTACACCGTGGTGGAGCA
GTACGAGCGCGCCGAGGCCCGCCACCACCTGTTCCAGTAGGAATTCGCGGCC
GCACTCGAGATATCTAGACCCAGCTTTCTTGTACAAAGTTGGCATTATAAGAA

AGCATTGCTTATCAATTTGTTGCAACGAACAGGTCACTATCAGTCAAAATAA

AATCATTATTTGCCATCCAGCTGCAGCTCTGGCCCGTGTCTCAAAATCTCTGA

TGTTACATTGCACAAGATAAAAATATATCATCATGAACAATAAAACTGTCTG

CTTACATAAACAGTAATACAAGGGGTGTTATGAGCCATATTCAACGGGAAAC

GTCGAGGCCGCGATTAAATTCCAACATGGATGCTGATTTATATGGGTATAAA

TGGGCTCGCGATAATGTCGGGCAATCAGGTGCGACAATCTATCGCTTGTATG

GGAAGCCCGATGCGCCAGAGTTGTTTCTGAAACATGGCAAAGGTAGCGTTGC

CAATGATGTTACAGATGAGATGGTCAGACTAAACTGGCTGACGGAATTTATG

CCTCTTCCGACCATCAAGCATTTTATCCGTACTCCTGATGATGCATGGTTACT

CACCACTGCGATCCCCGGAAAAACAGCATTCCAGGTATTAGAAGAATATCCT

GATTCAGGTGAAAATATTGTTGATGCGCTGGCAGTGTTCCTGCGCCGGTTGCA

TTCGATTCCTGTTTGTAATTGTCCTTTTAACAGCGATCGCGTATTTCGTCTCGC

TCAGGCGCAATCACGAATGAATAACGGTTTGGTTGATGCGAGTGATTTTGAT

GACGAGCGTAATGGCTGGCCTGTTGAACAAGTCTGGAAAGAAATGCATAAAC

TTTTGCCATTCTCACCGGATTCAGTCGTCACTCATGGTGATTTCTCACTTGATA

ACCTTATTTTTGACGAGGGGAAATTAATAGGTTGTATTGATGTTGGACGAGTC

GGAATCGCAGACCGATACCAGGATCTTGCCATCCTATGGAACTGCCTCGGTG

AGTTTTCTCCTTCATTACAGAAACGGCTTTTTCAAAAATATGGTATTGATAAT

CCTGATATGAATAAATTGCAGTTTCATTTGATGCTCGATGAGTTTTTCTAATC

AGAATTGGTTAATTGGTTGTAACATTATTCAGATTGGGCCCCGTTCCACTGAG

CGTCAGACCCCGTAGAAAAGATCAAAGGATCTTCTTGAGATCCTTTTTTTCTG

CGCGTAATCTGCTGCTTGCAAACAAAAAAACCACCGCTACCAGCGGTGGTTT

GTTTGCCGGATCAAGAGCTACCAACTCTTTTTCCGAAGGTAACTGGCTTCAGC

AGAGCGCAGATACCAAATACTGTTCTTCTAGTGTAGCCGTAGTTAGGCCACC
ACTTCAAGAACTCTGTAGCACCGCCTACATACCTCGCTCTGCTAATCCTGTTA
CCAGTGGCTGCTGCCAGTGGCGATAAGTCGTGTCTTACCGGGTTGGACTCAA
GACGATAGTTACCGGATAAGGCGCAGCGGTCGGGCTGAACGGGGGGTTCGTG
CACACAGCCCAGCTTGGAGCGAACGACCTACACCGAACTGAGATACCTACAG
CGTGAGCTATGAGAAAGCGCCACGCTTCCCGAAGGGAGAAAGGCGGACAGG
TATCCGGTAAGCGGCAGGGTCGGAACAGGAGAGCGCACGAGGGAGCTTCCA
GGGGGAAACGCCTGGTATCTTTATAGTCCTGTCGGGTTTCGCCACCTCTGACT
TGAGCGTCGATTTTTGTGATGCTCGTCAGGGGGGCGGAGCCTATGGAAAAAC
GCCAGCAACGCGGCCTTTTTACGGTTCCTGGCCTTTTGCTGGCCTTTTGCTCAC
ATGTT

>pSLIK/TREPitt::P2A/Crimson

AKA 6-pSLIK-Zeo/TREPitt::P2A/Crimson-Control or pSLIK6. Sequence verified from 2758 to 3966 bp.

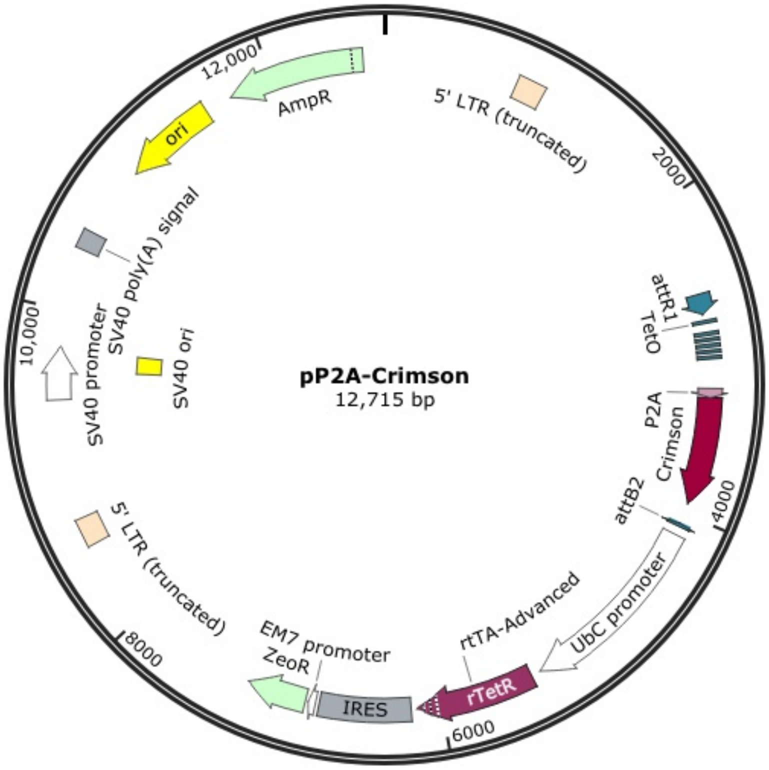

>6-pSLIK-Zeo/TREPitt::P2A/Crimson-Control

GTCGACGGATCGGGAGATCTCCCGATCCCCTATGGTGCACTCTCAGTACAAT

CTGCTCTGATGCCGCATAGTTAAGCCAGTATCTGCTCCCTGCTTGTGTGTTGG

AGGTCGCTGAGTAGTGCGCGAGCAAAATTTAAGCTACAACAAGGCAAGGCTT

GACCGACAATTGCATGAAGAATCTGCTTAGGGTTAGGCGTTTTGCGCTGCTTC

GCGATGTACGGGCCAGATATACGCGTTGACATTGATTATTGACTAGTTATTAA

TAGTAATCAATTACGGGGTCATTAGTTCATAGCCCATATATGGAGTTCCGCGT

TACATAACTTACGGTAAATGGCCCGCCTGGCTGACCGCCCAACGACCCCCGC

CCATTGACGTCAATAATGACGTATGTTCCCATAGTAACGCCAATAGGGACTTT

CCATTGACGTCAATGGGTGGAGTATTTACGGTAAACTGCCCACTTGGCAGTA

CATCAAGTGTATCATATGCCAAGTACGCCCCCTATTGACGTCAATGACGGTA

AATGGCCCGCCTGGCATTATGCCCAGTACATGACCTTATGGGACTTTCCTACT

TGGCAGTACATCTACGTATTAGTCATCGCTATTACCATGGTGATGCGGTTTTG

GCAGTACATCAATGGGCGTGGATAGCGGTTTGACTCACGGGGATTTCCAAGT

CTCCACCCCATTGACGTCAATGGGAGTTTGTTTTGGCACCAAAATCAACGGG

ACTTTCCAAAATGTCGTAACAACTCCGCCCCATTGACGCAAATGGGCGGTAG

GCGTGTACGGTGGGAGGTCTATATAAGCAGCGTTTTGCCTGTACTGGGTCT

CTCTGGTTAGACCAGATCTGAGCCTGGGAGCTCTCTGGCTAACTAGGGAACC

CACTGCTTAAGCCTCAATAAAGCTTGCCTTGAGTGCTTCAAGTAGTGTGTGCC

CGTCTGTTGTGTGACTCTGGTAACTAGAGATCCCTCAGACCCTTTTAGTCAGT

GTGGAAAATCTCTAGCAGTGGCGCCCGAACAGGGACTTGAAAGCGAAAGGG

AAACCAGAGGAGCTCTCTCGACGCAGGACTCGGCTTGCTGAAGCGCGCACGG

CAAGAGGCGAGGGGCGGCGACTGGTGAGTACGCCAAAAATTTTGACTAGCG

GAGGCTAGAAGGAGAGAGATGGGTGCGAGAGCGTCAGTATTAAGCGGGGGA

GAATTAGATCGCGATGGGAAAAAATTCGGTTAAGGCCAGGGGGAAAGAAAA

AATATAAATTAAAACATATAGTATGGGCAAGCAGGGAGCTAGAACGATTCGC

AGTTAATCCTGGCCTGTTAGAAACATCAGAAGGCTGTAGACAAATACTGGGA

CAGCTACAACCATCCCTTCAGACAGGATCAGAAGAACTTAGATCATTATATA

ATACAGTAGCAACCCTCTATTGTGTGCATCAAAGGATAGAGATAAAAGACAC

CAAGGAAGCTTTAGACAAGATAGAGGAAGAGCAAAACAAAAGTAAGACCAC

CGCACAGCAAGCGGCCGCTGATCTTCAGACCTGGAGGAGGAGATATGAGGG

ACAATTGGAGAAGTGAATTATATAAATATAAAGTAGTAAAAATTGAACCATT

AGGAGTAGCACCCACCAAGGCAAAGAGAAGAGTGGTGCAGAGAGAAAAAA

GAGCAGTGGGAATAGGAGCTTTGTTCCTTGGGTTCTTGGGAGCAGCAGGAAG

CACTATGGGCGCAGCGTCAATGACGCTGACGGTACAGGCCAGACAATTATTG

TCTGGTATAGTGCAGCAGCAGAACAATTTGCTGAGGGCTATTGAGGCGCAAC

AGCATCTGTTGCAACTCACAGTCTGGGGCATCAAGCAGCTCCAGGCAAGAAT

CCTGGCTGTGGAAAGATACCTAAAGGATCAACAGCTCCTGGGGATTTGGGGT

TGCTCTGGAAAACTCATTTGCACCACTGCTGTGCCTTGGAATGCTAGTTGGAG

TAATAAATCTCTGGAACAGATTTGGAATCACACGACCTGGATGGAGTGGGAC

AGAGAAATTAACAATTACACAAGCTTAATACACTCCTTAATTGAAGAATCGC

AAAACCAGCAAGAAAAGAATGAACAAGAATTATTGGAATTAGATAAATGGG

CAAGTTTGTGGAATTGGTTTAACATAACAAATTGGCTGTGGTATATAAAATTA

TTCATAATGATAGTAGGAGGCTTGGTAGGTTTAAGAATAGTTTTTGCTGTACT

TTCTATAGTGAATAGAGTTAGGCAGGGATATTCACCATTATCGTTTCAGACCC

ACCTCCCAACCCCGAGGGGACCCGACAGGCCCGAAGGAATAGAAGAAGAAG

GTGGAGAGAGAGACAGAGACAGATCCATTCGATTAGTGAACGGATCGGCAC

TGCGTGCGCCAATTCTGCAGACAAATGGCAGTATTCATCCACAATTTTAAAA

GAAAAGGGGGGATTGGGGGGTACAGTGCAGGGGAAAGAATAGTAGACATAA

TAGCAACAGACATACAAACTAAAGAATTACAAAAACAAATTACAAAAATTC

AAAATTTTCGGGTTTATTACAGGGACAGCAGAGATCCAGTTTGGTTAATTCTA

GACAGTTATCAACAAGTTTGTACAAAAAAGCTGAACGAGAAACGTAAAATG

ATATAAATATCAATATATTAAATTAGATTTTGCATAAAAAACAGACTACATA

ATACTGTAAAACACAACATATCCAGTCACTATGGGCGCCGGAACCAATTCAG

ATATCAAGTGCCACCTGACGTCTCCCTATCAGTGATAGAGAAGTCGACACGT

CTCGAGCTCCCTATCAGTGATAGAGAAGGTACGTCTAGAACGTCTCCCTATCA
GTGATAGAGAAGTCGACACGTCTCGAGCTCCCTATCAGTGATAGAGAAGGTA
CGTCTAGAACGTCTCCCTATCAGTGATAGAGAAGTCGACACGTCTCGAGCTC
CCTATCAGTGATAGAGAAGGTACGTCTAGAACGTCTCCCTATCAGTGATAGA
GAAGTCGACACGTCTCGAGCTCCCTATCAGTGATAGAGAAGGTACCCCCTAT
ATAAGCAGAGCTCGTTTAGTGAACCGTCAGATCGCCTGGAGACGCCATCCAC
GCTGTTTTGACCTCCATAGAAGACACCGGGACCGATCCAGCCTGGATCTAGG
AGGTCCGCCACCATGGCCGGATCCGGAGCCACGAACTTCTCTCTGTTAAAGC
AAGCAGGAGACGTGGAAGAAAACCCCGGTCCTATGGATAGCACTGAGAACG
TCATCAAGCCCTTCATGCGCTTCAAGGTGCACATGGAGGGCTCCGTGAACGG
CCACGAGTTCGAGATCGAGGGCGTGGGCGAGGGCAAGCCCTACGAGGGCAC
CCAGACCGCCAAGCTGCAAGTGACCAAGGGCGGCCCCCTGCCCTTCGCCTGG
GACATCCTGTCCCCCCAGTTCTTCTACGGCTCCAAGGCGTACATCAAGCACCC
CGCCGACATCCCCGACTACCTCAAGCAGTCCTTCCCCGAGGGCTTCAAGTGG
GAGCGCGTGATGAACTTCGAGGACGGCGGCGTGGTGACCGTGACCCAGGACT
CCTCCCTGCAGGACGGCACCCTCATCTACCACGTGAAGTTCATCGGCGTGAA
CTTCCCCTCCGACGGCCCCGTAATGCAGAAGAAGACTCTGGGCTGGGAGCCC
TCCACTGAGCGCAACTACCCCCGCGACGGCGTGCTGAAGGGCGAGAACCACA
TGGCGCTGAAGCTGAAGGGCGGCGGCCACTACCTGTGTGAGTTCAAGTCCAT
CTACATGGCCAAGAAGCCCGTGAAGCTGCCCGGCTACCACTACGTGGACTAC
AAGCTCGACATCACCTCCCACAACGAGGACTACACCGTGGTGGAGCAGTACG
AGCGCGCCGAGGCCCGCCACCACCTGTTCCAGTAGGAATTCGCGGCCGCACT
CGAGATATCTAGATAGTGACTGGATATGTTGTGTTTTACAGTATTATGTAGTC

TGTTTTTTATGCAAAATCTAATTTAATATATTGATATTTATATCATTTTACGTT

TCTCGTTCAGCTTTCTTGTACAAAGTGGTTGATAACATCTCGAGCCATAAAGA

TGGTTAATTAACCCACCCAAGATCTGGCCTCCGCGCCGGGTTTTGGCGCCTCC

CGCGGGCGCCCCCCTCCTCACGGCGAGCGCTGCCACGTCAGACGAAGGGCGC

AGCGAGCGTCCTGATCCTTCCGCCCGGACGCTCAGGACAGCGGCCCGCTGCT

CATAAGACTCGGCCTTAGAACCCCAGTATCAGCAGAAGGACATTTTAGGACG

GGACTTGGGTGACTCTAGGGCACTGGTTTTCTTTCCAGAGAGCGGAACAGGC

GAGGAAAAGTAGTCCCTTCTCGGCGATTCTGCGGAGGGATCTCCGTGGGGCG

GTGAACGCCGATGATTATATAAGGACGCGCCGGGTGTGGCACAGCTAGTTCC

GTCGCAGCCGGGATTTGGGTCGCGGTTCTTGTTTGTGGATCGCTGTGATCGTC

ACTTGGTGAGTAGCGGGCTGCTGGGCTGGCCGGGGCTTTCGTGGCCGCCGGG

CCGCTCGGTGGGACGGAAGCGTGTGGAGAGACCGCCAAGGGCTGTAGTCTGG

GTCCGCGAGCAAGGTTGCCCTGAACTGGGGGTTGGGGGGAGCGCAGCAAAA

TGGCGGCTGTTCCCGAGTCTTGAATGGAAGACGCTTGTGAGGCGGGCTGTGA

GGTCGTTGAAACAAGGTGGGGGGCATGGTGGGCGGCAAGAACCCAAGGTCT

TGAGGCCTTCGCTAATGCGGGAAAGCTCTTATTCGGGTGAGATGGGCTGGGG

CACCATCTGGGGACCCTGACGTGAAGTTTGTCACTGACTGGAGAACTCGGTTT

GTCGTCTGTTGCGGGGGCGGCAGTTATGGCGGTGCCGTTGGGCAGTGCACCC

GTACCTTTGGGAGCGCGCGCCCTCGTCGTGTCGTGACGTCACCCGTTCTGTTG

GCTTATAATGCAGGGTGGGGCCACCTGCCGGTAGGTGTGCGGTAGGCTTTTCT

CCGTCGCAGGACGCAGGGTTCGGGCCTAGGGTAGGCTCTCCTGAATCGACAG

GCGCCGGACCTCTGGTGAGGGGAGGGATAAGTGAGGCGTCAGTTTCTTTGGT

CGGTTTTATGTACCTATCTTCTTAAGTAGCTGAAGCTCCGGTTTTGAACTATG

CGCTCGGGGTTGGCGAGTGTGTTTTGTGAAGTTTTTTAGGCACCTTTTGAAAT

GTAATCATTTGGGTCAATATGTAATTTTCAGTGTTAGACTAGTAAATTGTCCG

CTAAATTCTGGCCGTTTTTGGCTTTTTTGTTAGACGAAGCTTGGTACCGAGCT

CGGATCTCCACCCCGTACCGGTCCTGCAGTCGAATTCACCATGTCTAGACTGG

ACAAGAGCAAAGTCATAAACGGAGCTCTGGAATTACTCAATGGTGTCGGTAT

CGAAGGCCTGACGACAAGGAAACTCGCTCAAAAGCTGGGAGTTGAGCAGCC

TACCCTGTACTGGCACGTGAAGAACAAGCGGGCCCTGCTCGATGCCCTGCCA

ATCGAGATGCTGGACAGGCATCATACCCACTTCTGCCCCCTGGAAGGCGAGT

CATGGCAAGACTTTCTGCGGAACAACGCCAAGTCATACCGCTGTGCTCTCCTC

TCACATCGCGACGGGGCTAAAGTGCATCTCGGCACCCGCCCAACAGAGAAAC

AGTACGAAACCCTGGAAAATCAGCTCGCGTTCCTGTGTCAGCAAGGCTTCTC

CCTGGAGAACGCACTGTACGCTCTGTCCGCCGTGGGCCACTTTACACTGGGCT

GCGTATTGGAGGAACAGGAGCATCAAGTAGCAAAAGAGGAAAGAGAGACAC

CTACCACCGATTCTATGCCCCCACTTCTGAGACAAGCAATTGAGCTGTTCGAC

CGGCAGGGAGCCGAACCTGCCTTCCTTTTCGGCCTGGAACTAATCATATGTGG

CCTGGAGAAACAGCTAAAGTGCGAAAGCGGCGGGCCGACCGACGCCCTTGA

CGATTTTGACTTAGACATGCTCCCAGCCGATGCCCTTGACGACTTTGACCTTG

ATATGCTGCCTGCTGACGCTCTTGACGATTTTGACCTTGACATGCTCCCCGGG

TAACTAAGTAAGGATCCGCGGCCGCACTAGAGGAATTCCGCCCCTCTCCCTC

CCCCCCCCCTAACGTTACTGGCCGAAGCCGCTTGGAATAAGGCCGGTGTGTG

TTTGTCTATATGTTATTTTCCACCATATTGCCGTCTTTTGGCAATGTGAGGGCC

CGGAAACCTGGCCCTGTCTTCTTGACGAGCATTCCTAGGGGTCTTTCCCCTCT

CGCCAAAGGAATGCAAGGTCTGTTGAATGTCGTGAAGGAAGCAGTTCCTCTG

GAAGCTTCTTGAAGACAAACAACGTCTGTAGCGACCCTTTGCAGGCAGCGGA

ACCCCCCACCTGGCGACAGGTGCCTCTGCGGCCAAAAGCCACGTGTATAAGA

TACACCTGCAAAGGCGGCACAACCCCAGTGCCACGTTGTGAGTTGGATAGTT

GTGGAAAGAGTCAAATGGCTCTCCTCAAGCGTAGTCAACAAGGGGCTGAAGG

ATGCCCAGAAGGTACCCCATTGTATGGGAATCTGATCTGGGGCCTCGGTGCA

CATGCTTTACATGTGTTTAGTCGAGGTTAAAAAACGTCTAGGCCCCCCGAAC

CACGGGGACGTGGTTTTCCTTTGAAAAACACGATGATAAGCTTACCGGTACG

CGTGATGTGTTGACAATTAATCATCGGCATAGTATATCGGCATAGTATAATAC

GACAAGGTGAGGAACTAAACCATGGCCAAGTTGACCAGTGCCGTTCCGGTGC

TCACCGCGCGCGACGTCGCCGGAGCGGTCGAGTTCTGGACCGACCGGCTCGG

GTTCTCCCGGGACTTCGTGGAGGACGACTTCGCCGGTGTGGTCCGGGACGAC

GTGACCCTGTTCATCAGCGCGGTCCAGGACCAGGTGGTGCCGGACAACACCC

TGGCCTGGGTGTGGGTGCGCGGCCTGGACGAGCTGTACGCCGAGTGGTCGGA

GGTCGTGTCCACGAACTTCCGGGACGCCTCCGGGCCGGCCATGACCGAGATC

GGCGAGCAGCCGTGGGGGCGGGAGTTCGCCCTGCGCGACCCGGCCGGCAAC

TGCGTGCACTTCGTGGCCGAGGAGCAGGACTGACACATCTGTACAAGTAAAG

CGGCCGCGACTCTAGATCATAATCAGCCATACCACATTTGTAGAGGTTTTACT

TGCTTTAAAAAACCTCCCACACCTCCCCCTGAACCTGAAACATAAAATGAAT

GCAATTGTTGTTGTTTAGTCCCTCCCAATTCGATATCAAGCTTATCGATAATC

AACCTCTGGATTACAAAATTTGTGAAAGATTGACTGGTATTCTTAACTATGTT

GCTCCTTTTACGCTATGTGGATACGCTGCTTTAATGCCTTTGTATCATGCTATT

GCTTCCCGTATGGCTTTCATTTTCTCCTCCTTGTATAAATCCTGGTTGCTGTCT

CTTTATGAGGAGTTGTGGCCCGTTGTCAGGCAACGTGGCGTGGTGTGCACTGT

GTTTGCTGACGCAACCCCCACTGGTTGGGGCATTGCCACCACCTGTCAGCTCC

TTTCCGGGACTTTCGCTTTCCCCCTCCCTATTGCCACGGCGGAACTCATCGCC

GCCTGCCTTGCCCGCTGCTGGACAGGGGCTCGGCTGTTGGGCACTGACAATT

CCGTGGTGTTGTCGGGGAAATCATCGTCCTTTCCTTGGCTGCTCGCCTGTGTT

GCCACCTGGATTCTGCGCGGGACGTCCTTCTGCTACGTCCCTTCGGCCCTCAA

TCCAGCGGACCTTCCTTCCCGCGGCCTGCTGCCGGCTCTGCGGCCTCTTCCGC

GTCTTCGCCTTCGCCCTCAGACGAGTCGGATCTCCCTTTGGGCCGCCTCCCCG

CATCGATACCGTCGACCTCGATCGAGACCTAGAAAAACATGGAGCAATCACA

AGTAGCAATACAGCAGCTACCAATGCTGATTGTGCCTGGCTAGAAGCACAAG

AGGAGGAGGAGGTGGGTTTTCCAGTCACACCTCAGGTACCTTTAAGACCAAT

GACTTACAAGGCAGCTGTAGATCTTAGCCACTTTTTAAAAGAAAAGGGGGGA

CTGGAAGGGCTAATTCACTCCCAACGAAGACAAGATATCCTTGATCTGTGGA

TCTACCACACACAAGGCTACTTCCCTGATTGGCAGAACTACACACCAGGGCC

AGGGATCAGATATCCACTGACCTTTGGATGGTGCTACAAGCTAGTACCAGTT

GAGCAAGAGAAGGTAGAAGAAGCCAATGAAGGAGAGAACACCCGCTTGTTA

CACCCTGTGAGCCTGCATGGGATGGATGACCCGGAGAGAGAAGTATTAGAGT

GGAGGTTTGACAGCCGCCTAGCATTTCATCACATGGCCCGAGAGCTGCATCC

GGACTGTACTGGGTCTCTCTGGTTAGACCAGATCTGAGCCTGGGAGCTCTCTG

GCTAACTAGGGAACCCACTGCTTAAGCCTCAATAAAGCTTGCCTTGAGTGCTT

CAAGTAGTGTGTGCCCGTCTGTTGTGTGACTCTGGTAACTAGAGATCCCTCAG

ACCCTTTTAGTCAGTGTGGAAAATCTCTAGCAGGGCCCGTTTAAACCCGCTGA

TCAGCCTCGACTGTGCCTTCTAGTTGCCAGCCATCTGTTGTTTGCCCCTCCCCC

GTGCCTTCCTTGACCCTGGAAGGTGCCACTCCCACTGTCCTTTCCTAATAAAA

TGAGGAAATTGCATCGCATTGTCTGAGTAGGTGTCATTCTATTCTGGGGGGTG

GGGTGGGGCAGGACAGCAAGGGGGAGGATTGGGAAGACAATAGCAGGCATG

CTGGGGATGCGGTGGGCTCTATGGCTTCTGAGGCGGAAAGAACCAGCTGGGG

CTCTAGGGGGTATCCCCACGCGCCCTGTAGCGGCGCATTAAGCGCGGCGGGT

GTGGTGGTTACGCGCAGCGTGACCGCTACACTTGCCAGCGCCCTAGCGCCCG

CTCCTTTCGCTTTCTTCCCTTCCTTTCTCGCCACGTTCGCCGGCTTTCCCCGTC

AAGCTCTAAATCGGGGGCTCCCTTTAGGGTTCCGATTTAGTGCTTTACGGCAC

CTCGACCCCAAAAAACTTGATTAGGGTGATGGTTCACGTAGTGGGCCATCGC

CCTGATAGACGGTTTTTCGCCCTTTGACGTTGGAGTCCACGTTCTTTAATAGT

GGACTCTTGTTCCAAACTGGAACAACACTCAACCCTATCTCGGTCTATTCTTT

TGATTTATAAGGGATTTTGCCGATTTCGGCCTATTGGTTAAAAAATGAGCTGA

TTTAACAAAAATTTAACGCGAATTAATTCTGTGGAATGTGTGTCAGTTAGGGT

GTGGAAAGTCCCCAGGCTCCCCAGCAGGCAGAAGTATGCAAAGCATGCATCT

CAATTAGTCAGCAACCAGGTGTGGAAAGTCCCCAGGCTCCCCAGCAGGCAGA

AGTATGCAAAGCATGCATCTCAATTAGTCAGCAACCATAGTCCCGCCCCTAA

CTCCGCCCATCCCGCCCCTAACTCCGCCCAGTTCCGCCCATTCTCCGCCCCAT

GGCTGACTAATTTTTTTTATTTATGCAGAGGCCGAGGCCGCCTCTGCCTCTGA

GCTATTCCAGAAGTAGTGAGGAGGCTTTTTTGGAGGCCTAGGCTTTTGCAAA

AAGCTCCCGGGAGCTTGTATATCCATTTTCGGATCTGATCAGCACGTGTTGAC

AATTAATCATCGGCATAGTATATCGGCATAGTATAATACGACAAGGTGAGGA

ACTAAACCATGGCCAAGTTGACCAGTGCCGTTCCGGTGCTCACCGCGCGCGA

CGTCGCCGGAGCGGTCGAGTTCTGGACCGACCGGCTCGGGTTCTCCCGGGAC

TTCGTGGAGGACGACTTCGCCGGTGTGGTCCGGGACGACGTGACCCTGTTCA

TCAGCGCGGTCCAGGACCAGGTGGTGCCGGACAACACCCTGGCCTGGGTGTG
GGTGCGCGGCCTGGACGAGCTGTACGCCGAGTGGTCGGAGGTCGTGTCCACG
AACTTCCGGGACGCCTCCGGGCCGGCCATGACCGAGATCGGCGAGCAGCCGT
GGGGGCGGGAGTTCGCCCTGCGCGACCCGGCCGGCAACTGCGTGCACTTCGT
GGCCGAGGAGCAGGACTGACACGTGCTACGAGATTTCGATTCCACCGCCGCC
TTCTATGAAAGGTTGGGCTTCGGAATCGTTTTCCGGGACGCCGGCTGGATGAT
CCTCCAGCGCGGGGATCTCATGCTGGAGTTCTTCGCCCACCCCAACTTGTTTA
TTGCAGCTTATAATGGTTACAAATAAAGCAATAGCATCACAAATTTCACAAA
TAAAGCATTTTTTTCACTGCATTCTAGTTGTGGTTTGTCCAAACTCATCAATGT
ATCTTATCATGTCTGTATACCGTCGACCTCTAGCTAGAGCTTGGCGTAATCAT
GGTCATAGCTGTTTCCTGTGTGAAATTGTTATCCGCTCACAATTCCACACAAC
ATACGAGCCGGAAGCATAAAGTGTAAAGCCTGGGGTGCCTAATGAGTGAGCT
AACTCACATTAATTGCGTTGCGCTCACTGCCCGCTTTCCAGTCGGGAAACCTG
TCGTGCCAGCTGCATTAATGAATCGGCCAACGCGCGGGGAGAGGCGGTTTGC
GTATTGGGCGCTCTTCCGCTTCCTCGCTCACTGACTCGCTGCGCTCGGTCGTT
CGGCTGCGGCGAGCGGTATCAGCTCACTCAAAGGCGGTAATACGGTTATCCA
CAGAATCAGGGGATAACGCAGGAAAGAACATGTGAGCAAAAGGCCAGCAAA
AGGCCAGGAACCGTAAAAAGGCCGCGTTGCTGGCGTTTTTCCATAGGCTCCG
CCCCCCTGACGAGCATCACAAAAATCGACGCTCAAGTCAGAGGTGGCGAAAC
CCGACAGGACTATAAAGATACCAGGCGTTTCCCCCTGGAAGCTCCCTCGTGC
GCTCTCCTGTTCCGACCCTGCCGCTTACCGGATACCTGTCCGCCTTTCTCCCTT
CGGGAAGCGTGGCGCTTTCTCATAGCTCACGCTGTAGGTATCTCAGTTCGGTG
TAGGTCGTTCGCTCCAAGCTGGGCTGTGTGCACGAACCCCCCGTTCAGCCCG

ACCGCTGCGCCTTATCCGGTAACTATCGTCTTGAGTCCAACCCGGTAAGACAC

GACTTATCGCCACTGGCAGCAGCCACTGGTAACAGGATTAGCAGAGCGAGGT

ATGTAGGCGGTGCTACAGAGTTCTTGAAGTGGTGGCCTAACTACGGCTACAC

TAGAAGAACAGTATTTGGTATCTGCGCTCTGCTGAAGCCAGTTACCTTCGGAA

AAAGAGTTGGTAGCTCTTGATCCGGCAAACAAACCACCGCTGGTAGCGGTGG

TTTTTTTGTTTGCAAGCAGCAGATTACGCGCAGAAAAAAAGGATCTCAAGAA

GATCCTTTGATCTTTTCTACGGGGTCTGACGCTCAGTGGAACGAAAACTCACG

TTAAGGGATTTTGGTCATGAGATTATCAAAAAGGATCTTCACCTAGATCCTTT

TAAATTAAAAATGAAGTTTTAAATCAATCTAAAGTATATATGAGTAAACTTG

GTCTGACAGTTACCAATGCTTAATCAGTGAGGCACCTATCTCAGCGATCTGTC

TATTTCGTTCATCCATAGTTGCCTGACTCCCCGTCGTGTAGATAACTACGATA

CGGGAGGGCTTACCATCTGGCCCCAGTGCTGCAATGATACCGCGAGACCCAC

GCTCACCGGCTCCAGATTTATCAGCAATAAACCAGCCAGCCGGAAGGGCCGA

GCGCAGAAGTGGTCCTGCAACTTTATCCGCCTCCATCCAGTCTATTAATTGTT

GCCGGGAAGCTAGAGTAAGTAGTTCGCCAGTTAATAGTTTGCGCAACGTTGT

TGCCATTGCTACAGGCATCGTGGTGTCACGCTCGTCGTTTGGTATGGCTTCAT

TCAGCTCCGGTTCCCAACGATCAAGGCGAGTTACATGATCCCCCATGTTGTGC

AAAAAAGCGGTTAGCTCCTTCGGTCCTCCGATCGTTGTCAGAAGTAAGTTGG

CCGCAGTGTTATCACTCATGGTTATGGCAGCACTGCATAATTCTCTTACTGTC

ATGCCATCCGTAAGATGCTTTTCTGTGACTGGTGAGTACTCAACCAAGTCATT

CTGAGAATAGTGTATGCGGCGACCGAGTTGCTCTTGCCCGGCGTCAATACGG

GATAATACCGCGCCACATAGCAGAACTTTAAAAGTGCTCATCATTGGAAAAC

GTTCTTCGGGGCGAAAACTCTCAAGGATCTTACCGCTGTTGAGATCCAGTTCG

ATGTAACCCACTCGTGCACCCAACTGATCTTCAGCATCTTTTACTTTCACCAG
CGTTTCTGGGTGAGCAAAAACAGGAAGGCAAAATGCCGCAAAAAAGGGAAT
AAGGGCGACACGGAAATGTTGAATACTCATACTCTTCCTTTTTCAATATTATT
GAAGCATTTATCAGGGTTATTGTCTCATGAGCGGATACATATTTGAATGTATT
TAGAAAAATAAACAAATAGGGGTTCCGCGCACATTTCCCCGAAAAGTGCCAC
CTGAC
